# Building an Immune-Related Genes Model to Predict Treatment, Extracellular Matrix, and Prognosis of Head and Neck Squamous Cell Carcinoma

**DOI:** 10.1155/2023/6680731

**Published:** 2023-07-11

**Authors:** Yushi Yang, Yang Feng, Qin Liu, Ji Yin, Chenglong Cheng, Cheng Fan, Chenhui Xuan, Jun Yang

**Affiliations:** ^1^Department of Otolaryngology and Ophthalmology, Anji County People' s Hospital, Zhejiang, China; ^2^Department of Radiation Oncology, Shanghai Ninth People' s Hospital, Shanghai Jiaotong University School of Medicine, Shanghai, China; ^3^Department of Neurosurgery, Anyue County People' s Hospital, Sichuan, China; ^4^The Affiliated Traditional Chinese Medicine Hospital of Southwest Medical University, Sichuan, China; ^5^Department of Endocrinology, The Affiliated Third Hospital of Chengdu Traditional Chinese Medicine University, Sichuan, China; ^6^Department of Endocrinology, Chengdu Pidu District Hospital of Traditional Chinese Medicine, Sichuan, China; ^7^Department of Cardiology, Anyue County People's Hospital, Sichuan, China

## Abstract

Due to the considerable heterogeneity of head and neck squamous cell carcinoma (HNSCC), individuals with comparable TNM stages who receive the same treatment strategy have varying prognostic outcomes. In HNSCC, immunotherapy is developing quickly and has shown effective. We want to develop an immune-related gene (IRG) prognostic model to forecast the prognosis and response to immunotherapy of patients. In order to analyze differential expression in normal and malignant tissues, we first identified IRGs that were differently expressed. Weighted gene coexpression network analysis (WGCNA) was used to identify modules that were highly related, and univariate and multivariate Cox regression analyses were also used to create a predictive model for IRGs that included nine IRGs. WGCNA identified the four most noteworthy related modules. Patients in the model's low-risk category had a better chance of survival. The IRGs prognostic model was also proved to be an independent prognostic predictor, and the model was also substantially linked with a number of clinical characteristics. The low-risk group was associated with immune-related pathways, a low incidence of gene mutation, a high level of M1 macrophage infiltration, regulatory T cells, CD8 T cells, and B cells, active immunity, and larger benefits from immune checkpoint inhibitors (ICIs) therapy. The high-risk group, on the other hand, had suppressive immunity, high levels of NK and CD4 T-cell infiltration, high gene mutation rates, and decreased benefits from ICI therapy. As a result of our research, a predictive model for IRGs that can reliably predict a patient's prognosis and their response to both conventional and immunotherapy has been created.

## 1. Introduction

Head and neck cancer ranks as the 6th most prevalent malignancy worldwide, with an annual incidence of 930,000 cases and 470,000 deaths [1]. Head and neck squamous cell carcinoma (HNSCC) is the majority of head and neck cancer, and the major risk factors for the development of HNSCC include tobacco, alcohol consumption, and human papillomavirus infection [1]. The main reasons for death in advanced HNSCC patients are local recurrence, remote metastasis, and therapeutic failure owing to resistance to routine chemotherapy [2]. In the last years, immune checkpoint inhibitors (ICIs) are regarded as revolutionary agents in medicinal therapy for malignant tumors, especially for HNSCC [3].

Cancer immunotherapy operates on the basis that the host's immune system may get activated by the cancer cells, which identifies and eliminates them [4]. While immune checkpoints can prevent overwhelming inflammatory responses and the progression of autoimmunity, they can as well be operated as a mechanism of tumor immune evasion [5]. ICIs reactivate immune responses against cancer by blocking immune checkpoint pathways, including antiprogrammed death-1, antiprogrammed death-1 ligand, and anticytotoxic T-lymphocyte-associated protein 4 antibodies [5]. Nevertheless, the main restriction of this treatment is the poor patient response rate. Only a small percentage of HNSCC patients respond to immunotherapy, and the responses seen are usually durable and profound, but many others show widespread resistance to immunotherapy [6]. Therefore, novel therapeutic markers demand to urgent study to identify the ideal subgroup of HNSCC for immunotherapy.

In the research, we attempted to construct a prognostic signature for HNSCC that can predict the efficacy of routine therapy and immunotherapy. First, we assessed immune-related genes (IRGs) of HNSCC and identified survival-associated differentially expressed IRGs in significantly relevant modules by weighted gene coexpression network analysis (WGCNA) to develop an IRGs prognostic model. We then estimated its predictive value among HNSCC patients, examined the immune profile of the prognostic model, and characterized it with gene mutation, N6-methyladenosine (*m*^6^A) mRNA stats, tumor immune dysfunction and exclusion (TIDE) score, tumor inflammation signature (TIS) score, and chemotherapeutic efficacy. Conclusively, the IRGs prognostic model was a prospective prognostic signature for precise prediction of patient prognosis and reaction to traditional treatment and immunotherapy.

## 2. Materials and Methods

### 2.1. Preparation of Data

The RNA-seq data and clinicopathological features of HNSCC samples (The Cancer Genome Atlas (TCGA)-HNSCC and GSE65858) were procured from the TCGA (http://portal.gdc.cancer.gov) and gene expression omnibus (GEO) (http://www.ncbi.nlm.nih.gov/geo/) [7]. The gene transfer format files with gene names and the transcript annotation of the genome available were obtained in the Ensemble database (http://asia.ensembl.org) [8]. A dataset of recognized IRGs was acquired in the InnateDB (http://www.innatedb.com) databases and the ImmPort database (http://www.immport.org) [9]. The somatic mutation data of HNSCC patients were retrieved from the TCGA.

### 2.2. Identification of Significantly Relevant Modules with WGCNA

Differential expression analysis (|log2FC| > 0.585, false discovery rate (FDR) <0.05) was utilized to recognize differentially expressed IRGs. The gene ontology (GO) and Kyoto Encyclopedia of Genes and Genomes (KEGG) analyses were employed to analyze these differentially expressed IRGs (*P* value <0.05) [10].

After that, significantly relevant modules were obtained using WGCNA. First, a similarity matrix that calculates Pearson's correlation coefficient between two genes was constructed in light of expression data. Second, an adjacency matrix with a network type of sign was acquired based on the similarity matrix by selecting five as the soft threshold and further converted to a topological matrix with the topological overlap measure, which was used to depict the degree of association between genes. Then, genes were grouped at a distance of 1-TOM, and gene modules were recognized using the dynamic hybrid tree-cut algorithm. Lastly, nine modules were determined based on a minimal cluster size of 25, a correlation coefficient greater than 0.9, and a merging threshold function of 0.25. These modules (the green, pink, brown, and red modules) were recognized as significantly relevant modules.

Finally, to show as many protein interactions as possible in the different modules, the protein–protein interaction networks (PPI) of these IRGs in significantly relevant modules were retrieved, respectively, from STRING (http://string-db.org) and were visualized separately by Cytoscape 3.8.2 software (minimum required interaction score >0.2) [11]. And these IRGs in significantly relevant modules were analyzed individually by GO and KEGG (*P*-value <0.05).

### 2.3. Development and Evaluation of Prognostic Model

Univariate Cox regression and Kaplan–Meier (KM) analysis were carried out to identify the association of these IRGs in significantly relevant modules with survival, and twenty IRGs with *P* < 0.05 were determined to be survival-associated IRGs. These survival-related IRGs were utilized by multivariate Cox regression analysis to construct an IRGs prognostic model with nine IRGs. The specific risk score for each patient was calculated, and the risk score formula was as follows:

(1) $$\begin{array}{c} \sum_{\text{i}=1}^{\text{k}}1^{\surd }iSi. \end{array}$$

We used KM survival analysis to evaluate the prognostic ability of the model in the TCGA and GEO cohorts. Chi-squared test was applied to investigate the association between the prognostic model and clinical characteristics. Wilcoxon signed-rank test was carried out to compute the risk score differences among distinct groups of clinical features. Univariate and multivariate Cox analyses were utilized to verify that the signature was an independent predictor of clinical prognosis. Finally, decision curve analysis (DCA) was employed to assess the net benefit of five markers for clinical decision-making, and a nomogram integrating prognostic signatures was built to predict the survival rates of patients.

### 2.4. Exploration of Molecular and Immunological Characteristics and ICIs Therapeutics

Gene set enrichment analysis (GSEA) based on the KEGG and HALLMARK genes was applied to identify the signaling pathways in different groups (*P* < 0.05 and FDR <0.25). The gene mutation analysis was used to identify the quantity and quality of gene mutations among the signature subgroups. Wilcoxon signed-rank test was employed to investigate differences in expression levels of *m*^6^A-related genes in different groups.

To analyze the immune characteristics of this model, the relative proportion of immune cells was computed using CIBERSORT (http://cibersort.stanford.edu/) [12]. Single-sample GSEA (ssGSEA) was applied to identify differences of immune function between different groups. Survival status was compared with the immune cell proportions and immune function between different groups. And we carried out Wilcoxon signed-rank test to explore the expression level of ICIs-related molecules among different groups.

TIDE score was obtained from TIDE (http://tide.dfci.harvard.edu/), and TIS score was computed based on the expression of the 18 genes [13, 14]. Then, the time-dependent receiver-operating characteristic (ROC) curve analyses were performed to acquire the area under the curve (AUC) and compare the prognostic value among the model, TIDE, and TIS. To evaluate the sensitivity of chemotherapy in the IRGs prognostic model, Wilcoxon signed-rank test was used to compare the difference in the half inhibitory concentration (IC_50_) among the prognostic model subgroups.

## 3. Results

### 3.1. Identification of Significantly Related Modules

By performing differential expression analysis and intersecting these genes with identified IRGs, 920 differentially expressed IRGs were extracted, of which 726 were upregulated and 194 were downregulated (*Supplementary 1*). In total, 920 IRGs were enriched in various GO and KEGG terms (details in *Supplementary 2*), and the first 30 GO and KEGG terms are presented in *Supplementary 1* (Figures S1B and S1C). Nine significantly related modules were recognized by WGCNA on the basis of the scale-free network (*Supplementary 3*). PPI was constructed separately, and GO and KEGG were conducted respectively on the genes of the green, pink, brown, and red modules (*Supplementary 4*).

### 3.2. Development of IRGs Prognostic Index

**Figure 1:**
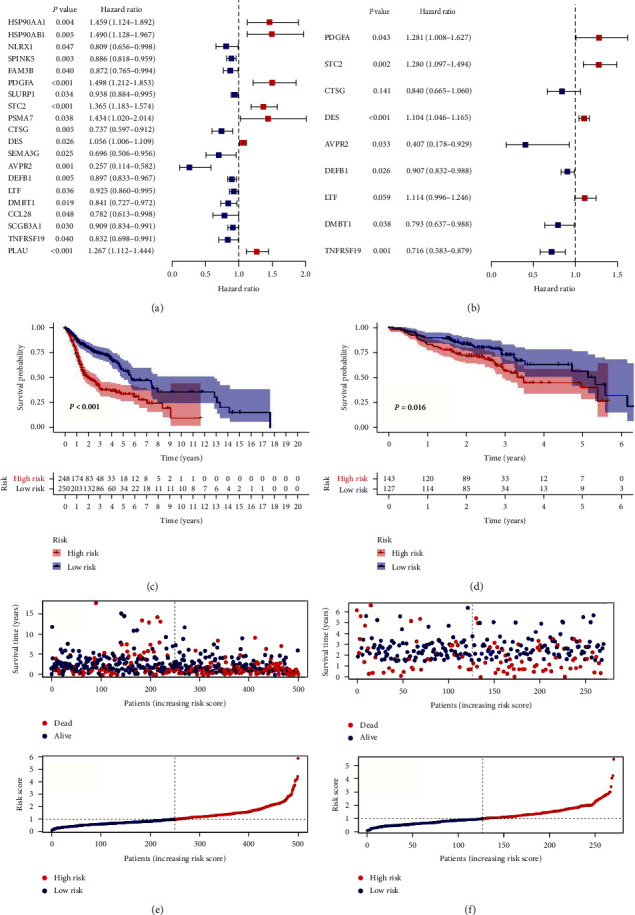
Prognostic analysis of distinct prognostic signature groups: (a) univariate Cox analysis of 20 IRGs; (b) multivariate Cox regression analysis of nine IRGs; (c and d) Kaplan–Meier survival analysis; (e and f) risk scores and survival outcome.

To construct an IRGs prognostic model, 20 survival-associated IRGs were extracted in accordance with these genes of significantly relevant modules (the green, pink, brown, and red modules), shown in Figure 1(a) and *Supplementary 5*. Among 20 survival-associated IRGs, nine IRGs were identified via multivariate Cox regression analysis, thereby establishing the prognostic model (Figure 1(b)). Although some genes had *P*-values greater than 0.05 in the multivariate Cox regression analysis, these genes still had some prognostic value. On the basis of the respective median risk score, 249 patients were assigned to the high-risk group and 250 patients to the low-risk group in TCGA, 143 patients to the high-risk group, and 127 patients to the low-risk group in GEO.

### 3.3. Estimation of the Risk Assessment Signature

**Figure 2:**
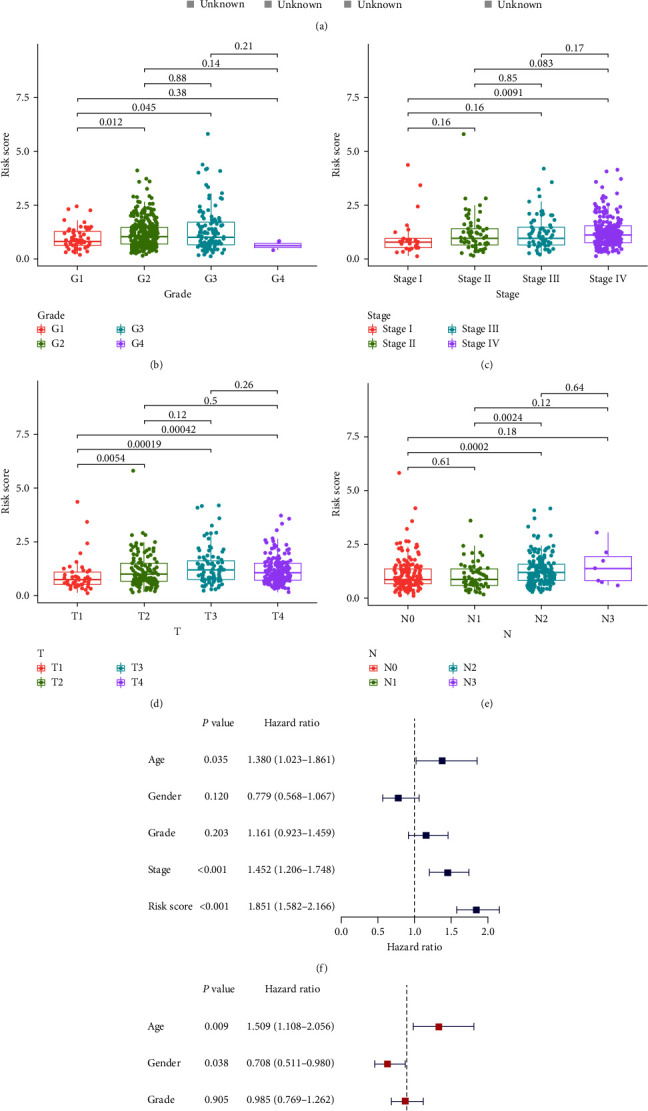
Assessment of clinical characteristics for the model: (a–e) the relationship between the model and traditional clinical characteristics; (f and g) univariate and multivariate Cox analysis of the model and traditional clinical characteristics.  ^*∗*^*P* < 0.05,  ^*∗∗*^*P* < 0.01,  ^*∗∗∗*^*P* < 0.001.

**Figure 3:**
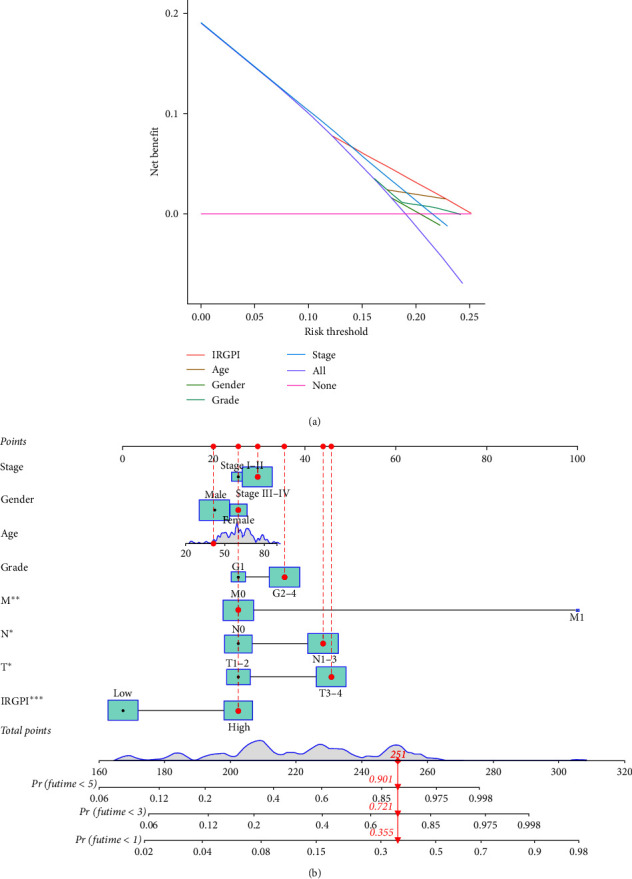
Exploration of the model: (a) the DCA of the risk model; (b) a nomogram based on clinical characteristics and risk groups.  ^*∗*^*P* < 0.05,  ^*∗∗*^*P* < 0.01,  ^*∗∗∗*^*P* < 0.001.

KM analysis demonstrated that survival rates were remarkably lower in high-risk HNSCC patients (Figures 1(c) and 1(d)). Besides, the risk score distribution for HNSCC patients is presented in Figures 1(e) and 1(f), indicating that the clinical prognosis of patients in the high-risk group was generally worse. Based on a set of *χ*^2^ tests, the strip chart (Figure 2(a)) and consequent scatter diagrams show that risk scores are strongly related to clinico-pathological characteristics of HNSCC patients, including pathological stage (Figure 2(b)), clinical stage (Figure 2(c)), T stage (Figure 2(d)) and N stage (Figure 2(e)). Univariate and multivariate Cox regression analysis validated that the model was an independent prognostic risk factor (Figures 2(f) and 2(g), details in *Supplementary 6*). The result of DCA indicated that the prognostic model was more precise than other conventional clinico-pathological characteristics (Figure 3(a)). The nomogram combining the IRGs prognostic model and clinico-pathological characteristics was dependable and sensitive for survival prediction of HNSCC patients (Figure 3(b)).

### 3.4. Investigation of Molecular Characteristics

**Figure 4:**
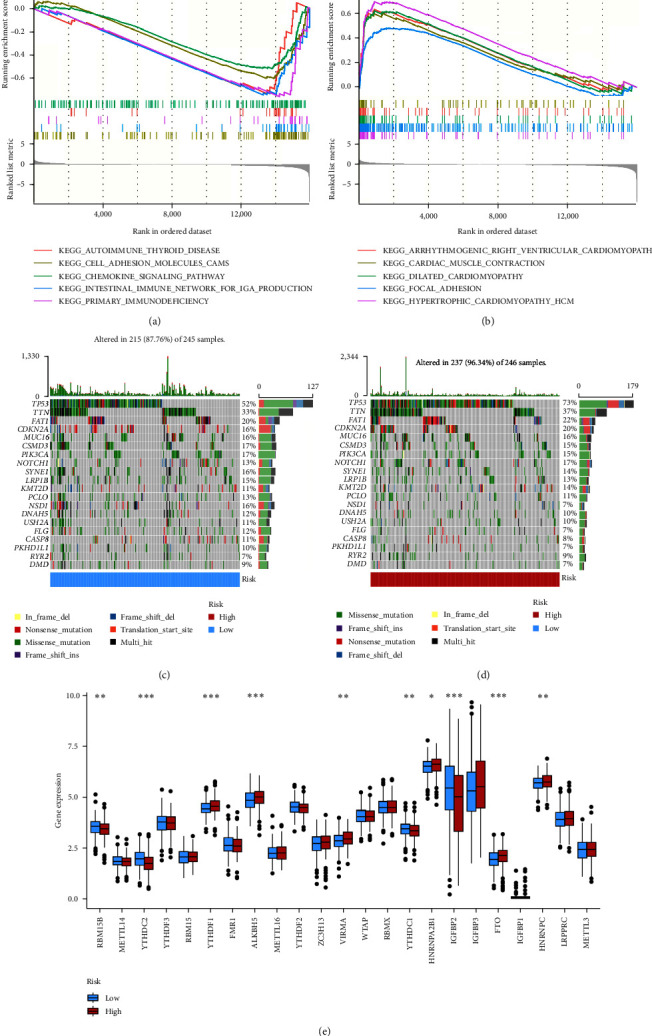
Molecular features of distinct groups: (a and b) genes enriched in distinct groups; (c and d) gene mutation analysis of distinct groups; (e) differences of *m*^6^A-related genes expression among distinct groups.  ^*∗*^*P* < 0.05,  ^*∗∗*^*P* < 0.01,  ^*∗∗∗*^*P* < 0.001.

Genes in the low-risk group were largely enriched in cell adhesion molecules, chemokines, and immune-related pathways, whereas genes in the high-risk group were majorly enriched in focal adhesion (Figures 4(a) and 4(b), details in *Supplementary 7*). The results of gene mutation analysis displayed that more genes were mutated in the high-risk group (Figures 4(c) and 4(d)). The commonest type of mutation was the missense variant mutations, followed by nonsense mutation and multiple mutations of a gene. The top 10 genes having the greatest mutation rate were TP53, TTN, FAT1, CDKN2A, MUC16, CSMD3, PIK3CA, NOTCH1, SYNE1, and LRP1B. Mutations in the TP53, TTN, CDKN2A, and NOTCH1 genes were more frequent among the high-risk group, whereas mutations in the NSD1 and FLG genes were more prevalent among the other group. Comparing *m*^6^A-related mRNAs in different groups revealed that the expression levels of YTHDC2, YTHDF1, ALKBH5, IGFBP2, and FTO (*P* < 0.001), RBM15B, VIRMA, YTHDC1, and HNRNPC (*P* < 0.01), and HNRNPA2B1 (*P* < 0.05) were statistically different (Figure 4(e)).

### 3.5. Evaluation of Immunological Characteristics

**Figure 5:**
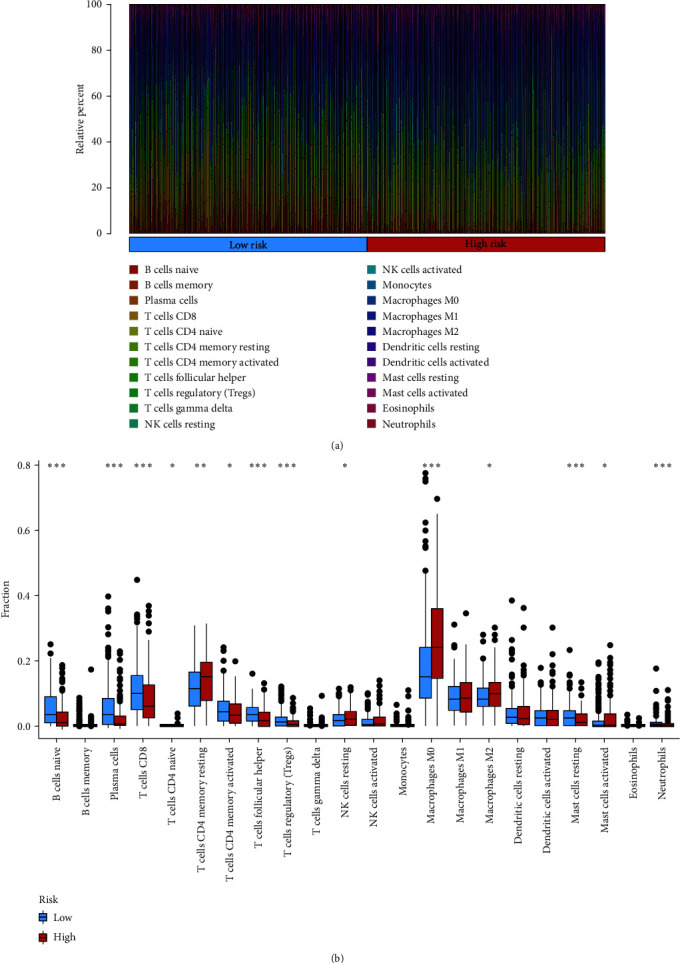
The landscape of the TME in the TCGA-HNSCC set: (a) the proportion of TME cells in the TCGA set; (b) the proportion of each TME cell type in distinct groups.  ^*∗*^*P* < 0.05,  ^*∗∗*^*P* < 0.01,  ^*∗∗∗*^*P* < 0.001.

**Figure 6:**
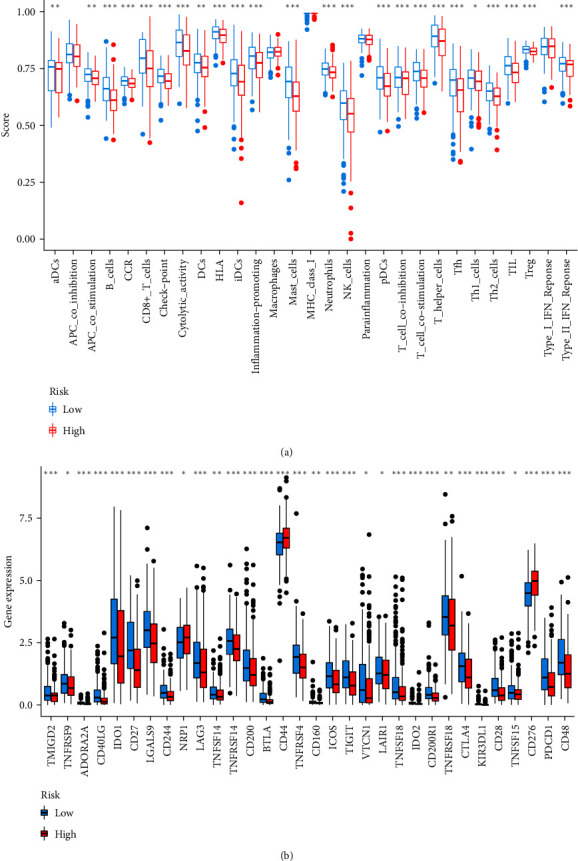
Evaluation of immune-related functions and ICIs-related molecules: (a) immune-related function analyzed by the ssGSEA in distinct groups; (b) differences of checkpoint-related genes expression among diverse groups.  ^*∗*^*P* < 0.05,  ^*∗∗*^*P* < 0.01,  ^*∗∗∗*^*P* < 0.001.

Based on CIBERSORT, we found that NK-cell resting, eosinophil, T-cell CD4^+^ naive, mast cell resting, M0 macrophage were more plentiful in the high-risk group, and T-cell follicular helper, T-cell CD8^+^, mast cell activated, regulatory T-cell, B-cell naive, B-cell plasma, B-cell memory, myeloid dendritic cell resting, monocyte, M1 macrophage were more plentiful in the low-risk group (Figures 5(a) and 5(b), details in *Supplementary 8*). The vast majority of immune functions were statistically different among distinct groups, except for antigen-presenting cell coinhibition, macrophages, major histocompatibility complex class I, response to type I Interferon, and parainflammation (Figure 6(a)). The relationship between immune cell proportions and immune function and survival status was analyzed (*Supplementary 9* and *Supplementary 10*). We also explored whether the model was associated with ICIs and found statistically significant differences in the expression of CTLA-4, PDCD1, LAG3, TIGIT, BTLA, and others in different groups (Figure 6(b)).

### 3.6. Exploration of ICIs Therapeutics

**Figure 7:**
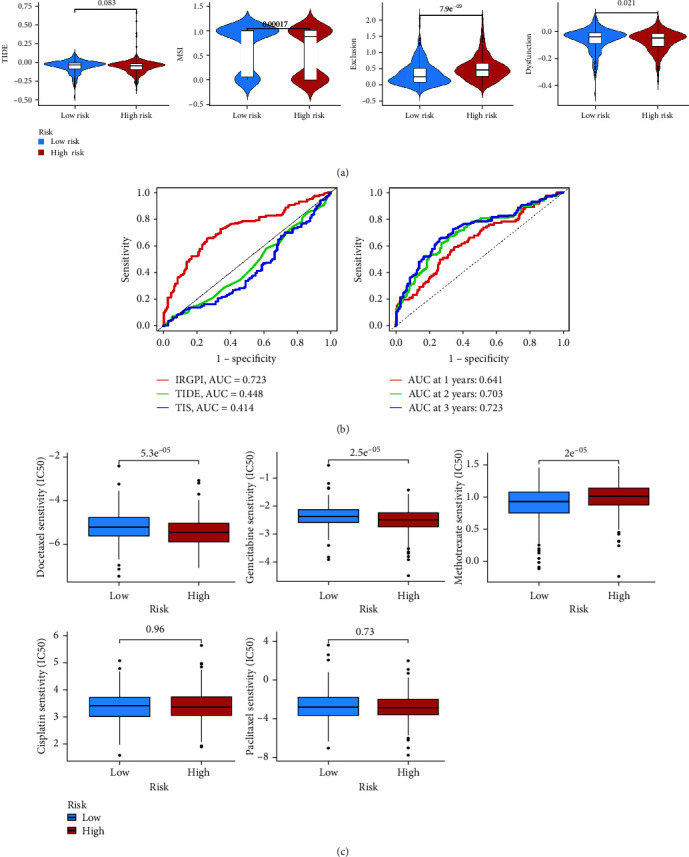
Exploration of ICIs therapeutics in different risk groups: (a) TIDE, MSI, T-cell exclusion, and dysfunction score in distinct groups; (b) ROC analysis of the IRGs prognostic model, TIDE, and TIS on 3-year and ROC analysis of the model in 1-, 2-, and 3-year; (c) differences of IC_50_ of chemotherapeutics among different groups.

To estimate the underlying clinical efficacy of immunotherapy of the prognostic model, TIDE was used to evaluate this, and lower TIDE prediction scores indicated a higher likelihood of patients benefiting from treatment with ICIs, as represented by a lower potential for immune evasion. In our results, no statistical differences in TIDE scores were found between distinct groups, but TIDE scores were low in both groups, indicating that both groups were able to benefit better from treatment with ICIs (Figure 7(a)). In addition, we found that the low-risk group had a higher microsatellite instability (MSI) score and T-cell dysfunction score, whereas the high-risk group had a higher T-cell exclusion score. The AUC of the ROC analysis for 3-year survival prediction showed that the model was more accurate than TIDE and MSI. The AUC values for the 1-, 2-, and 3-year ROC curves in the IRGs prognostic model were all high, indicating that the model had superior sensitivity and specificity for survival prediction (Figure 7(b)). Beyond ICIs blocking therapy, we found that the IC_50_ of docetaxel, gemcitabine, and methotrexate were statistically different among different groups; while the difference in IC_50_ for cisplatin and paclitaxel was minimal (Figure 7(c)).

### 3.7. Role of PDGFA in HNSC Progression

**Figure 8:**
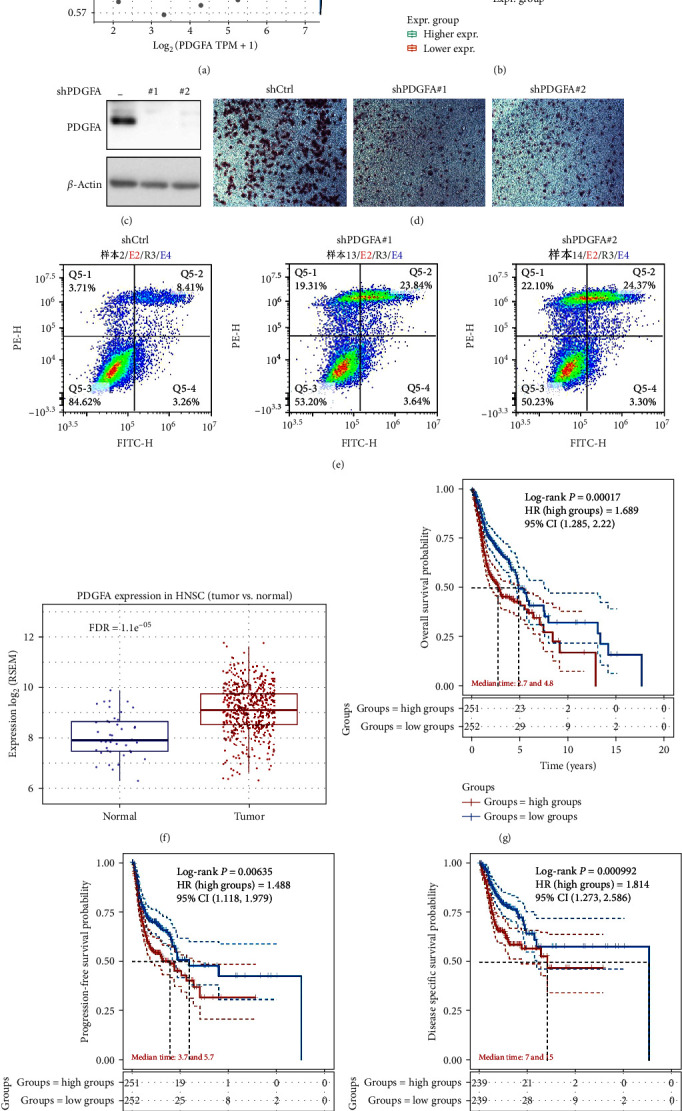
Role of PDGFA in HNSC progression: (a) correlation of PDGFA with TGF-*β* based on TCGA database; (b) PDGFA expression levels in the tissues of HNSC patients; (c) WB analysis validation for PDGFA knockdown in HNSC; (d) PDGFA knockdown inhibit the metastatic potential of HNSC cells in vitro; (e) immunofluorescence detection of the apoptosis rate in control and PDGFA knocked down cells; (f) PDGFA expression levels in HNSC and normal tissues; (g–i) PDGFA expression levels predict the prognosis in HNSC patient.

To clarify the role of PDGFA in HNSC progression, we found by analyzing the TCGA database that: PDGFA expression levels showed a significant positive correlation with TGF-*β* by Figure 8(a). Correspondingly, we found that PDGFA expression levels were significantly higher in the tissues of HNSC patients with higher epithelial-mesenchymal transition (EMT) viability compared to those with lower EMT viability (Figure 8(b)). The above data suggest that the aberrant expression of PDGFA expression levels in HNSC may promote distal metastasis of HNSC by promoting EMT and thereby. Further, we performed a knockdown of PDGFA in HNSC and verified the knockdown efficiency by the western blot (WB) (Figure 8(c)). Based on this, we found by transwell assay that: PDGFA knockdown could significantly inhibit the metastatic potential of HNSC cells in vitro (Figure 8(d)). The current first-line treatment regimen of HNSC is still dominated by radiotherapy, and the activation of EMT is closely associated with chemoresistance, according to which we speculate that the abnormal expression of PDGFA may confer chemoresistant properties to HNSC cells. To test our conjecture, we performed 5-Fu treatment in control and PDGFA cells and detected the apoptosis rate by flow assay, and found that: PDGFA knockdown could significantly promote apoptosis induced by 5-Fu treatment, i.e., PDGFA could enhance HNSC for chemotherapy accordingly (Figure 8(e)). In addition, we found that PDGFA expression levels were significantly elevated in HNSC compared to normal tissues (Figure 8(f)). And the abnormally elevated level of its expression predicted poor prognosis of patients (Figures 8(g)–8(i)).

## 4. Discussion

ICIs therapeutics have been demonstrated to be an accurate and safe therapy for relapsed or refractory HNSCC patients [15, 16]. As the general response rate to treatment with ICIs remains low, it is crucial to ascertain those patients who could profit most from those treatments [17, 18]. Over the past few years, although there have been many evaluations of various prognostic signatures for HNSCC, we remain without identifying a validated biomarker for predicting immunotherapy and immune system response. We emphasize the necessity of identifying the optimal treatment population and prognostic genes for response to immunotherapy.

WGCNA is a virtual approach to finding modules of strongly correlated genes, modules, and external sample characteristics and can help recognize potential IRGs or therapeutic targets [19, 20]. WGCNA was used to identify nine IRGs, and the IRGs prognostic model was developed based on TCGA. The model has been shown to be an effective IRGs for HNSCC, with better survival in patients with the low-risk group.

Various studies have indicated that a variety of immune-related biomarkers are related to the outcome of patients with various malignancies, particularly HNSCC [21–23]. Wang et al. [24] set up a nine IRGs signature to analyze the tumor microenvironment and indicate the prognosis for HNSCC. She et al. [25] identified 27 IRGs and established a signature that offers a thorough overview of the immune microenvironment and prognosis of HNSCC. In this study, some of the IRGs that have been recognized during modeling play an important role in the malignant phenotype of different cancer types, especially HNSCC. Humphries et al. reported that CTSG was highly expressed in HNSCC tissues in contrast to paraneoplastic tissues and affected cancer progression and metastasis by activating and inhibiting a large network of protein hydrolytic interactions [25]. Yang et al. found that STC2 facilitates HNSCC proliferation and metastasis by modulating the G1/S cell cycle transition [25]. Zhang et al. [26] demonstrated that the re-expression of LTF could impair the malignancy of HNSCC cells. In summary, the IRGs prognostic model we developed in the study was a novel model that could recognize new biomarkers to be further studied.

The results of GSEA showed that the low-risk group was enriched in cell adhesion molecules, chemokines, and immune-related pathways, whereas the high-risk group was enriched in focal adhesion, which implied that the low-risk group was characterized by immune activation and suppression of tumor progression. The gene mutations of the high- and low-risk groups showed that the most prevalent type of mutation was the missense variant mutation, next to nonsense mutations and multiple mutations of a gene, as mentioned previously [26]. TP53 mutation was the most frequent mutation between different groups (73% vs. 52%). The majority of HNSCC patients (about 70%) have the TP53 mutation, while the incidence of this genetic change varies according to the head and neck area [27]. Furthermore, TP53 mutation is related to more invasive disease and poorer patient prognosis in HNSCC [27]. Thus, the low-risk group with low TP53 mutations had a better prognosis, in agreement with our results. DNA methylation is a form of chemical modification of DNA that can alter genetic expression without altering the DNA sequence [28, 29]. Methylation-related genes in HNSCC have been extensively studied in recent years [30, 31]. In this study, some *m*^6^A-related genes not only differed significantly different among different groups but also correlated with the prognosis of various malignancies, such as FTO, ALKBH5, YTHDF1, and YTHDC2, in agreement with our results [32–35]. In addition, we found that abnormal expression of PDGFA expression levels in HNSC may promote distal metastasis of HNSC by promoting EMT and thus HNSC.

A comprehensive understanding of the immunological landscape can help find new ways to treat HNSCC. CD4^+^ T cells and NK cells were more prevalent in the high-risk group, whereas CD8^+^ T cells, M1 macrophages, regulatory T cells, and B cells were more common in the low-risk group. A large number of researches have indicated that CD4^+^ T cells are related to poor prognosis [32–35]. Conversely, the high density of CD8^+^ T cells and M1 macrophages are indicative of a good prognosis [36–39]. These research findings are in accordance with ours. However, regulatory T cells and B cells were negatively related to the prognosis of patients in some researches, while others indicated the opposite [40–42]. Similarly, the results of the immune function analysis showed that the low-risk group had more immune activities, which predicted a better prognosis for the low-risk group. Furthermore, our results indicated that the low-risk group was positively associated with the expression of most ICIs, including CTLA-4, PDCD1, LAG3, TIGIT, and BTLA, suggesting that patients in the low-risk group might be able to benefit more from ICI therapy.

TIDE has been developed based on two different mechanisms of tumor immune escape: T-cell dysfunction in cytotoxic T lymphocytes (CTL)-high tumors and T-cell exclusion in CTL-low tumors [43]. In our study, there was no significant difference in TIDE scores between different risk groups, but both their TIDE scores were low. The high-risk group had a higher T-cell exclusion score and lower T-cell dysfunction score, and higher MSI score, which indicated that these patients had higher levels of T-cell exclusion. On the contrary, the low-risk group had a higher T-cell dysfunction score, MSI score, and lower T-cell exclusion score than the high-risk group, which demonstrated that these patients had higher levels of T-cell dysfunction and more MSI. Some researches have demonstrated the prevalence of MSI in HNSCC, and the high mutational burden caused by MSI makes the tumor immunogenic and sensitive to anti-PD1 therapy [44, 45]. TIS, an 18 gene signature developed by NanoString Technologies, has been verified in HNSCC clinical trials (KEYNOTE-012 and KEYNOTE-055) using single-agent pembrolizumab treatment, demonstrating a positive association with response and survival [16, 46]. In the research, the predictive value of the prognostic model was higher than that of TIDE and TIS, and the model consisted of only nine genes and was, therefore, easier to detect than TIDE and TIS. Our findings indicated that the IC_50_ of docetaxel, gemcitabine, and methotrexate were statistically different between different groups, whereas the difference in IC_50_ for cisplatin and paclitaxel was little.

However, the current study has several shortcomings and limitations. First, though external validation has been carried out to verify the predictive power of the model, the exact molecular mechanisms of the nine IRGs have not been explored in the present study. Second, our total sample size is relatively small, and the normal to tumor sample counts are nonproportional. Third, the results may be biased as the majority of samples from TCGA are nonmetastatic. Therefore, in order to further examine and validate our model, we want to recollect more clinical samples, increase the size of our sample, and carefully follow-up on our results.

## 5. Conclusion

In summary, this study demonstrated that a promising IRGs prognostic model might facilitate the differentiation of immune and molecular features, forecast patient prognosis, and aid in distinguishing those who could benefit from antitumor immunotherapy for HNSCC.

## Data Availability

Publicly available datasets were analyzed in this study; these can be found in TCGA (https://portal.gdc.cancer.gov/) and GEO (http://www.ncbi.nlm.nih.gov/geo/).

## Supplementary Materials

Supplementary 1 Determination of differentially expressed IRGs in HNSCC.

Supplementary 2 The details of GO and KEGG enrichment analysis.

Supplementary 3 Identification of modules by the WGCNA analysis.

Supplementary 4 Analysis of genes in significantly relevant modules.

Supplementary 5 Kaplan–Meier survival curves of twenty survival-associated IRGs (*P* < 0.05).

Supplementary 6 The detailed values of univariate and multivariate Cox regression analysis.

Supplementary 7 The GSEA in different groups.

Supplementary 8 The relative proportion of various immune cells by CIBERSORT.

Supplementary 9 Kaplan–Meier survival curves of immune cell proportion (*P* < 0.05).

Supplementary 10 Kaplan–Meier survival curves of immune function (*P* < 0.05).
