# Supplementary figures and images for: Building an Immune-Related Genes Model to Predict Treatment, Extracellular Matrix, and Prognosis of Head and Neck Squamous Cell Carcinoma
Source: Mediators Inflamm. 2023 Jul 11;2023:6680731. doi: 10.1155/2023/6680731 (PMC10353907; doi:10.1155/2023/6680731)

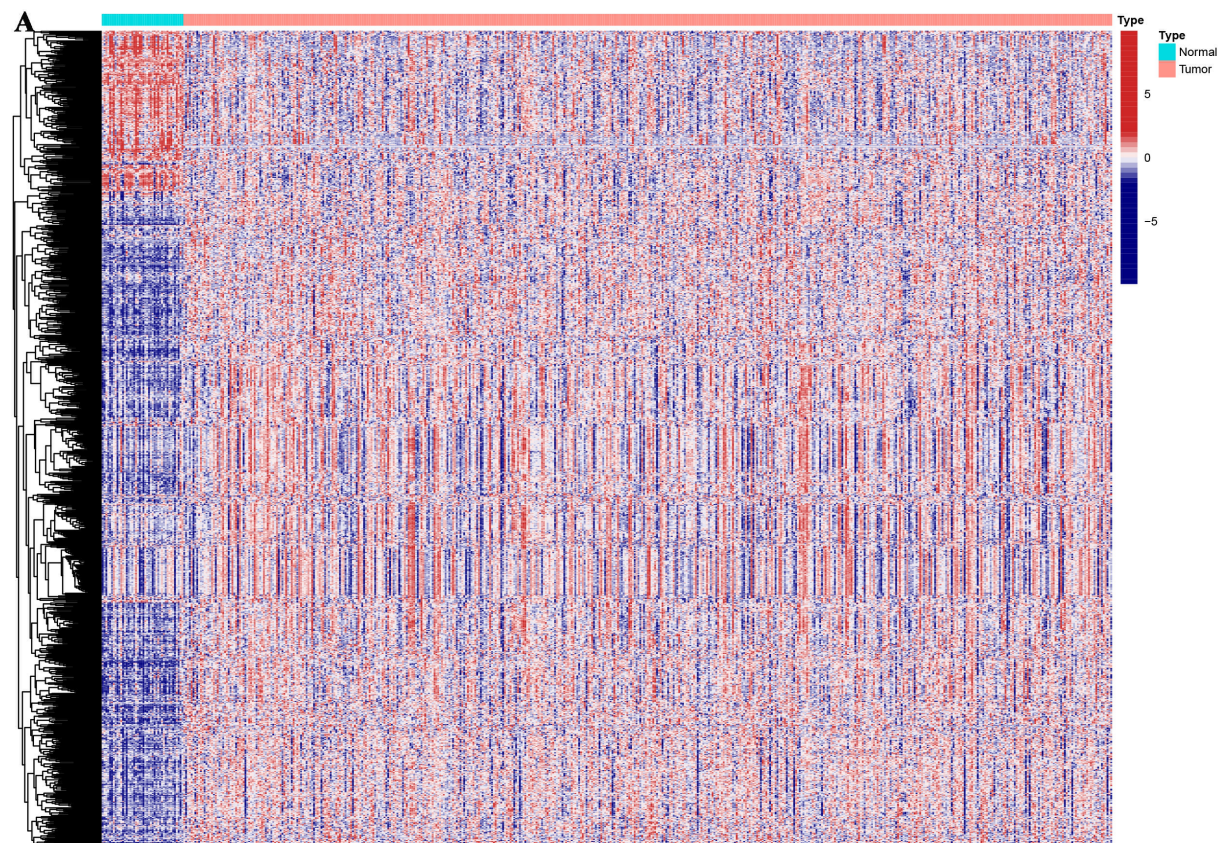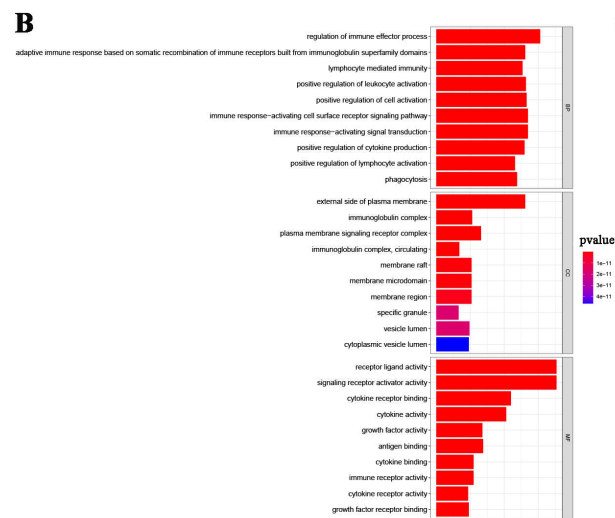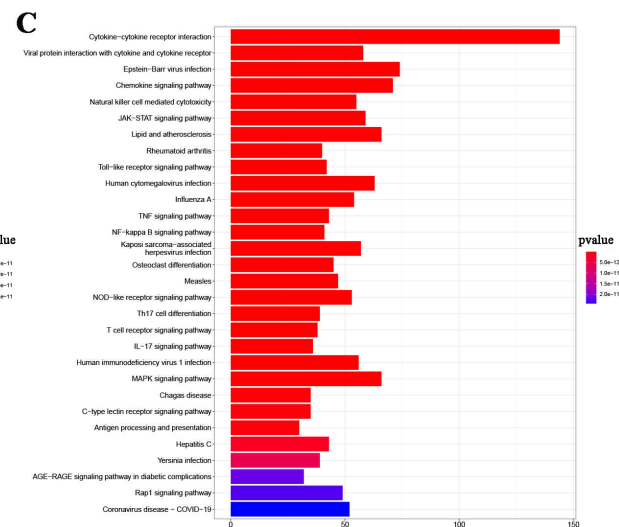

Supplement: Supplementary 1 — Determination of differentially expressed IRGs in HNSCC. [file 6680731.f1.pdf]

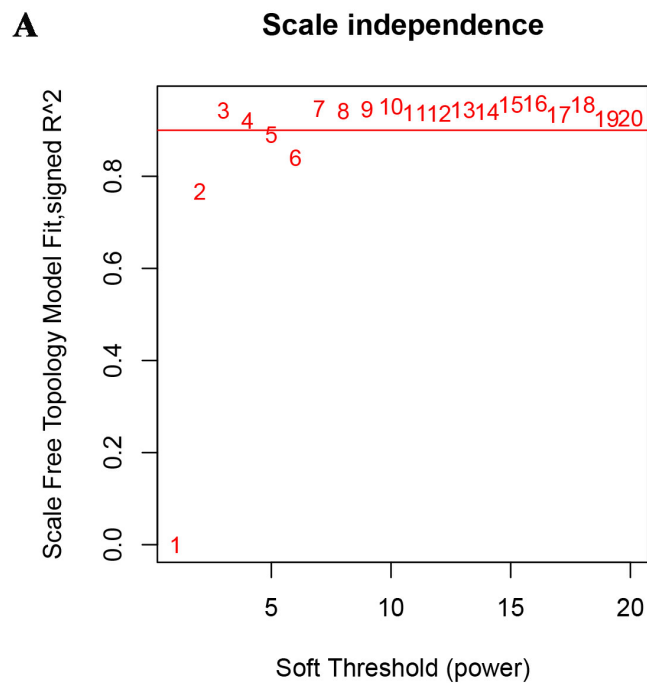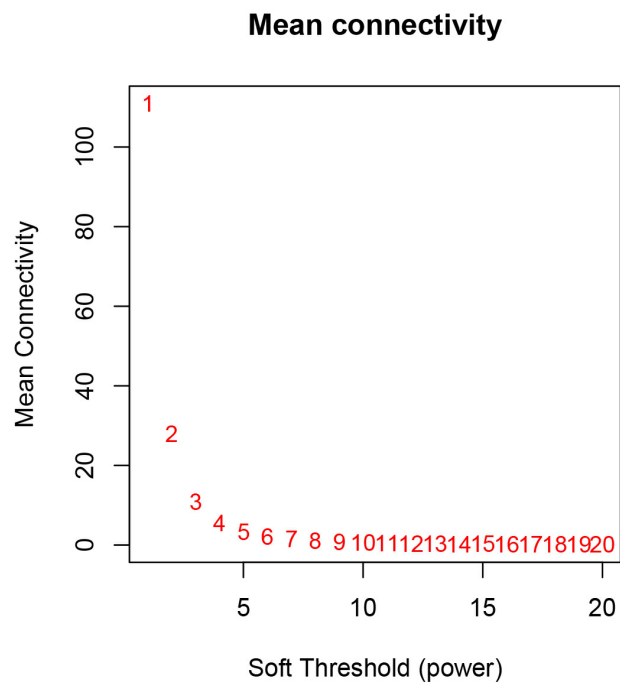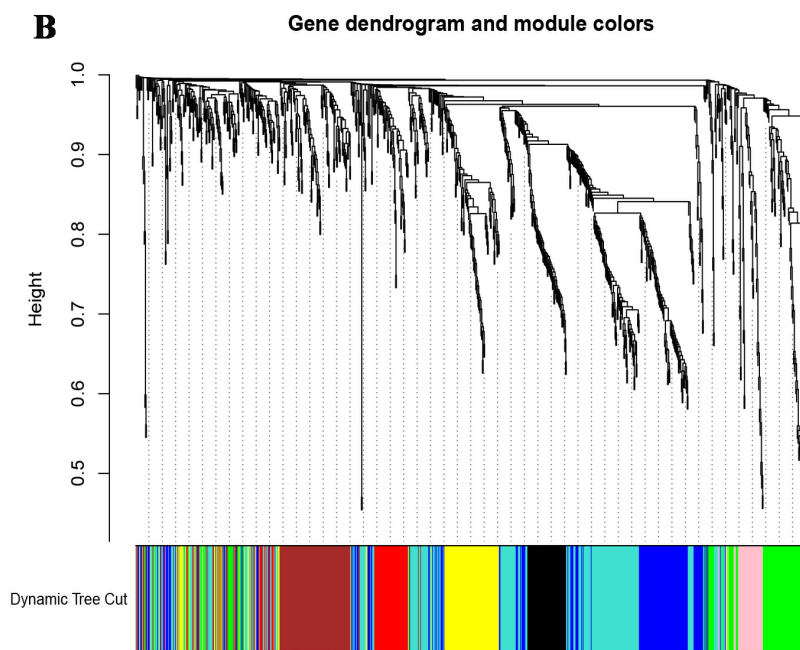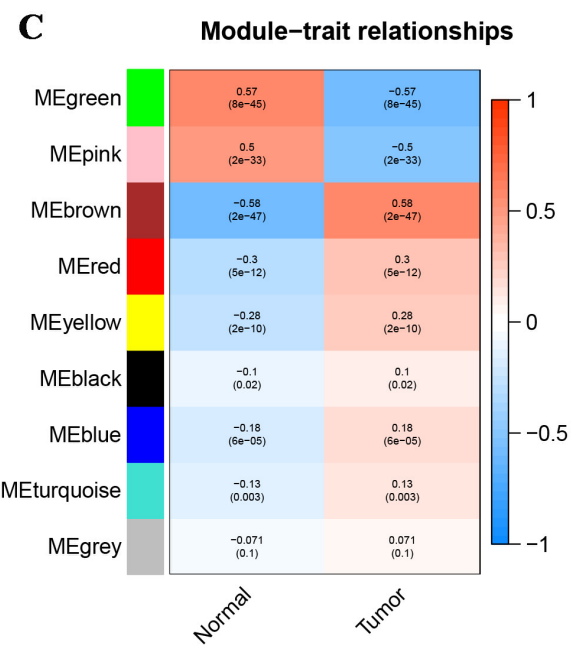

Supplement: Supplementary 3 — Identification of modules by the WGCNA analysis. [file 6680731.f3.pdf]

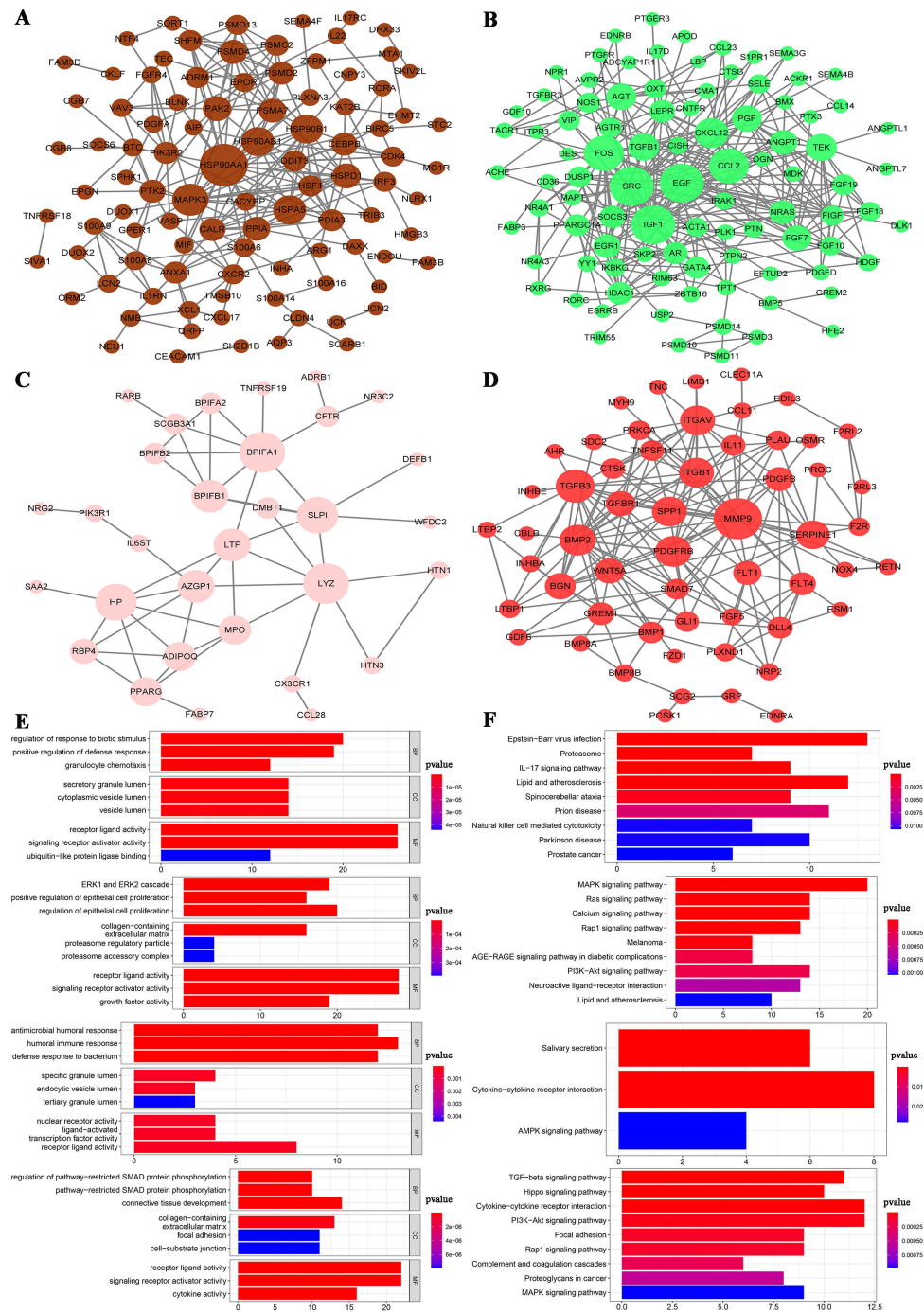

Supplement: Supplementary 4 — Analysis of genes in significantly relevant modules. [file 6680731.f4.pdf]

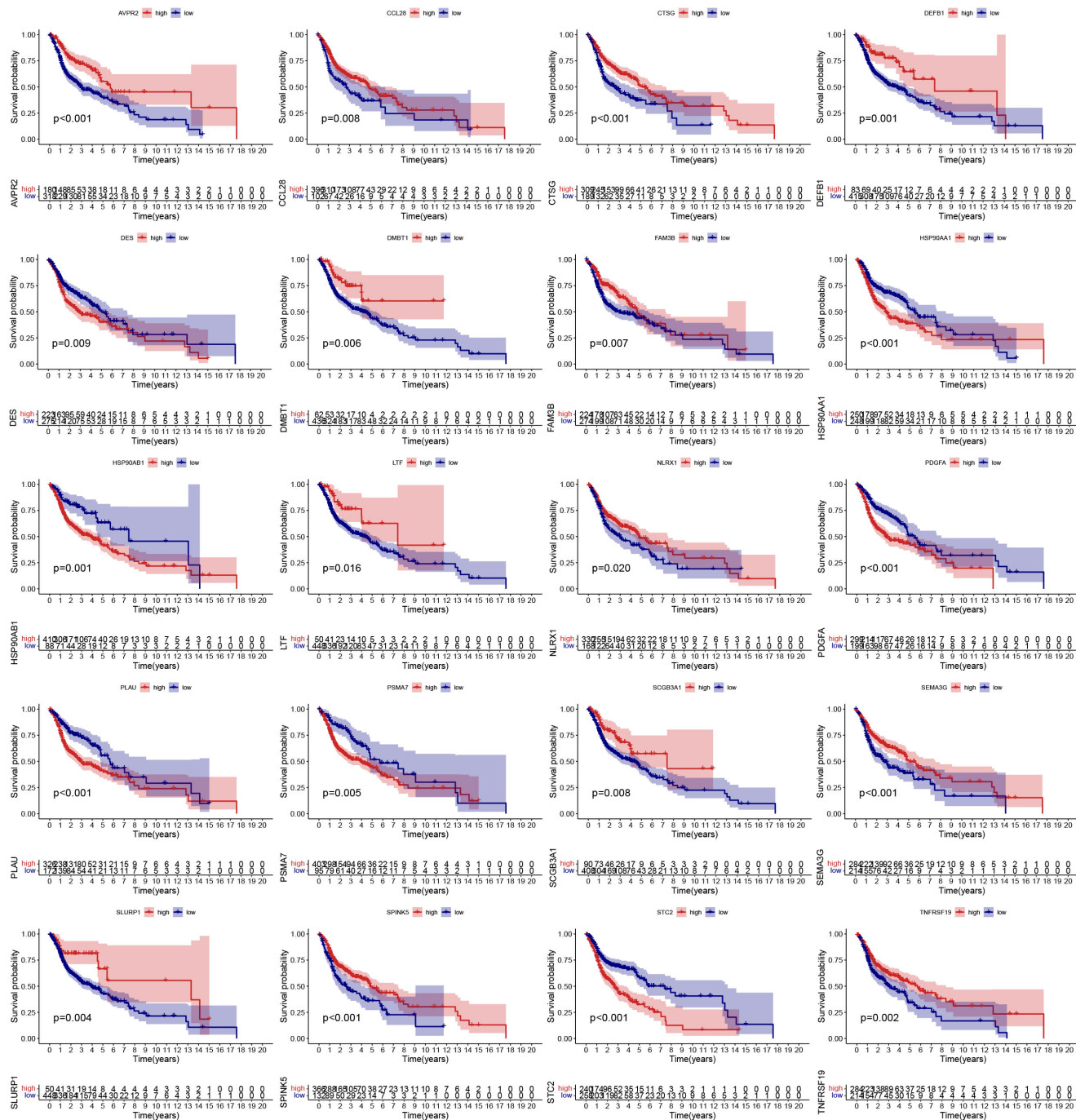

Supplement: Supplementary 5 — Kaplan–Meier survival curves of twenty survival-associated IRGs (P < 0.05). [file 6680731.f5.pdf]

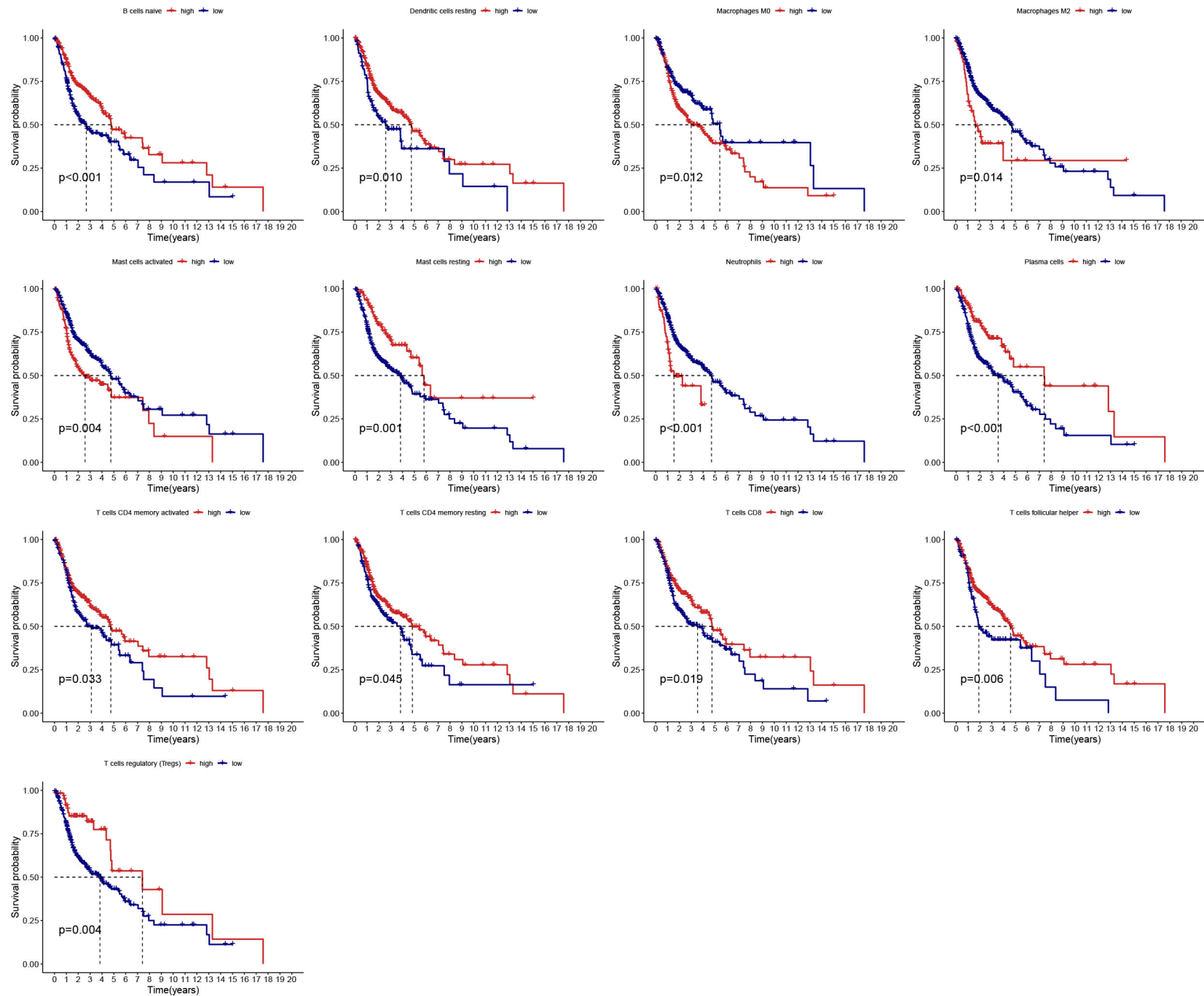

Supplement: Supplementary 9 — Kaplan–Meier survival curves of immune cell proportion (P < 0.05). [file 6680731.f9.pdf]

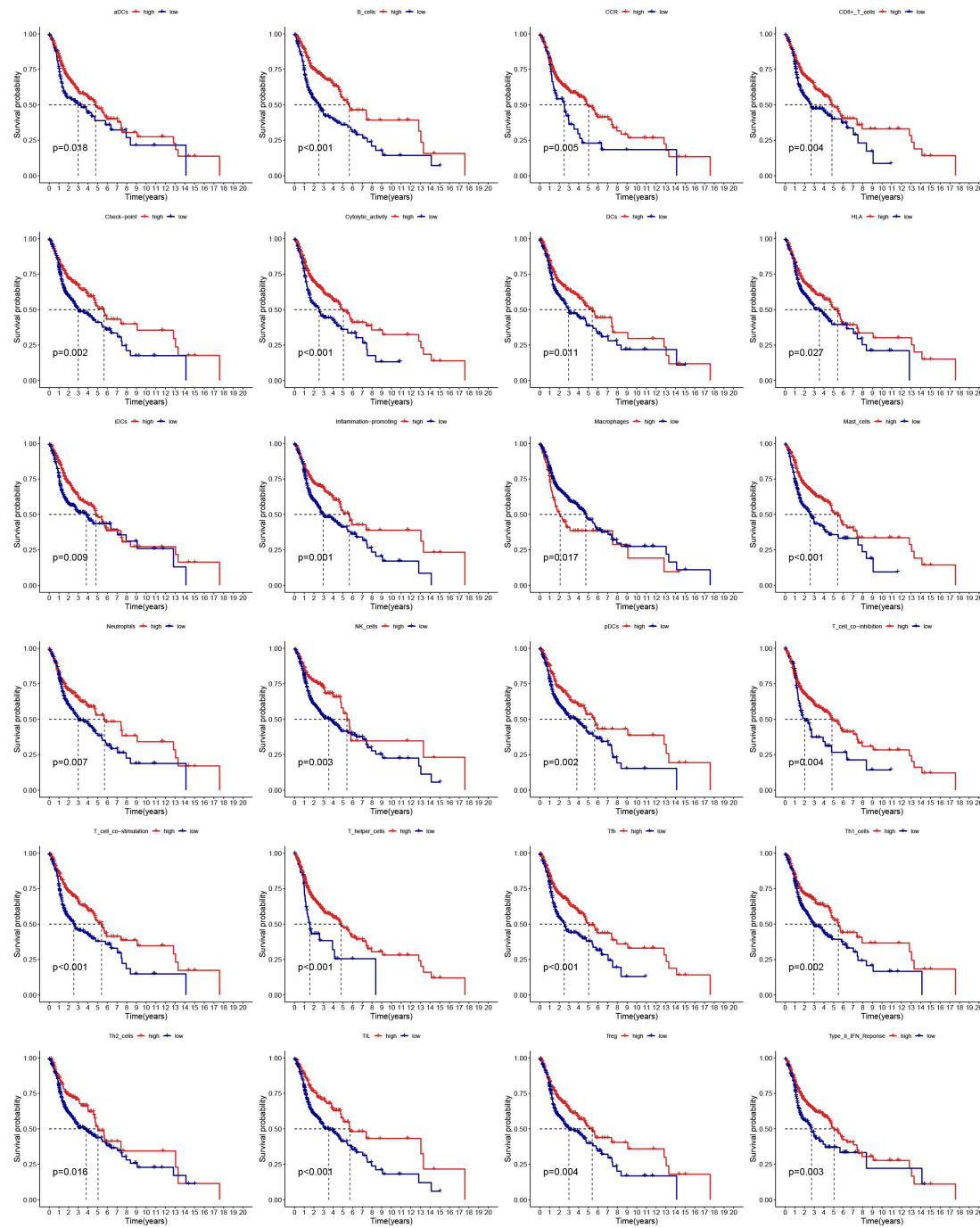

Supplement: Supplementary 10 — Kaplan–Meier survival curves of immune function (P < 0.05). [file 6680731.f10.pdf]
