# Supplementary material for: Building an Immune-Related Genes Model to Predict Treatment, Extracellular Matrix, and Prognosis of Head and Neck Squamous Cell Carcinoma
Source: Mediators Inflamm. 2023 Jul 11;2023:6680731. doi: 10.1155/2023/6680731 (PMC10353907; doi:10.1155/2023/6680731)
Supplement: Supplementary 2 — The details of GO and KEGG enrichment analysis. [file 6680731.f2.pdf]

ONTOLOG\	ID	Descriptior	GeneRatio	BgRatio	pvalue	p.adjust	qvalue	geneID
BP	GO:00026!	regulation	153/905	470/18866	1.84E-86	9.93E-83	5.22E-83	AZGP1/B2I
BP	GO:00024(	adaptive in	131/905	370/18866	6.38E-79	1.73E-75	9.07E-76	AZGP1/B2I
BP	GO:00024	lymphocyt	127/905	366/18866	3.14E-75	5.67E-72	2.98E-72	AZGP1/B2I
BP	GO:00026!	positive re	132/905	406/18866	3.45E-74	4.67E-71	2.45E-71	CD4/FCER1
BP	GO:00508(	positive re	133/905	421/18866	5.51E-73	5.96E-70	3.13E-70	CD4/FCER1
BP	GO:00024	immune re	135/905	481/18866	7.13E-67	5.51E-64	2.89E-64	CD4/FCER1
BP	GO:00027!	immune re	135/905	481/18866	7.13E-67	5.51E-64	2.89E-64	CD4/FCER1
BP	GO:00018	positive re	130/905	447/18866	2.13E-66	1.44E-63	7.56E-64	B2M/CD4/
BP	GO:00512!	positive re	116/905	357/18866	5.31E-65	3.19E-62	1.68E-62	CD4/HLA-A
BP	GO:00069(	phagocyto:	119/905	382/18866	2.19E-64	1.19E-61	6.23E-62	CALR/FCER
BP	GO:00028:	regulation	121/905	409/18866	1.22E-62	5.98E-60	3.14E-60	FCER1G/HI
BP	GO:00603	cell chemo	106/905	311/18866	1.02E-61	4.59E-59	2.41E-59	CALR/FCER
BP	GO:00069!	humoral in	115/905	377/18866	4.40E-61	1.83E-58	9.61E-59	HLA-A/HLA
BP	GO:00421	T cell activ	125/905	483/18866	2.19E-57	8.47E-55	4.45E-55	B2M/CD1C
BP	GO:00022:	response t	108/905	356/18866	4.70E-57	1.69E-54	8.90E-55	B2M/ICAM
BP	GO:00427	defense re	106/905	348/18866	3.49E-56	1.18E-53	6.20E-54	CD4/FCER1
BP	GO:00324!	response t	103/905	334/18866	3.66E-55	1.11E-52	5.86E-53	ICAM1/LTA
BP	GO:00313	positive re	110/905	385/18866	3.71E-55	1.11E-52	5.86E-53	FCER1G/HI
BP	GO:00305!	leukocyte (	85/905	232/18866	2.00E-52	5.70E-50	2.99E-50	CALR/FCER
BP	GO:00096	response t	100/905	349/18866	3.07E-50	8.31E-48	4.37E-48	IFNG/MICA
BP	GO:00508!	antigen re	95/905	325/18866	1.44E-48	3.70E-46	1.95E-46	CD4/HLA-A
BP	GO:00975	myeloid le	79/905	222/18866	1.23E-47	3.03E-45	1.59E-45	FCER1G/C)
BP	GO:00516(	defense re	84/905	258/18866	4.28E-47	1.01E-44	5.28E-45	IFNG/MICA
BP	GO:00380!	Fc recepto	81/905	241/18866	1.25E-46	2.81E-44	1.47E-44	FCER1G/HS
BP	GO:00071!	leukocyte (	98/905	364/18866	1.49E-46	3.23E-44	1.70E-44	CD4/HLA-A
BP	GO:00224(	regulation	106/905	439/18866	1.89E-45	3.94E-43	2.07E-43	CD4/HLA-A
BP	GO:00197	B cell medi	78/905	230/18866	2.99E-45	5.99E-43	3.15E-43	FCER1G/HI
BP	GO:19030:	regulation	92/905	329/18866	3.14E-45	6.06E-43	3.18E-43	CD4/HLA-A
BP	GO:00457	positive re	104/905	428/18866	7.31E-45	1.36E-42	7.16E-43	CALR/CD4/
BP	GO:00509	regulation	77/905	229/18866	2.28E-44	4.12E-42	2.16E-42	CALR/CXCL
BP	GO:00450	regulation	88/905	307/18866	3.10E-44	5.41E-42	2.84E-42	FCER1G/HI
BP	GO:00160(	immunogl	76/905	227/18866	1.21E-43	2.04E-41	1.07E-41	FCER1G/HI
BP	GO:00706(	leukocyte	88/905	313/18866	1.75E-43	2.88E-41	1.51E-41	CD4/HLA-A
BP	GO:00712	cellular res	78/905	246/18866	8.28E-43	1.32E-40	6.92E-41	HSPA5/ICA
BP	GO:00346	response t	88/905	320/18866	1.25E-42	1.93E-40	1.02E-40	ICAM1/LTA
BP	GO:00713!	cellular res	84/905	297/18866	9.65E-42	1.45E-39	7.62E-40	ICAM1/LTA
BP	GO:19037(	regulation	108/905	498/18866	1.46E-41	2.13E-39	1.12E-39	B2M/CD4/
BP	GO:00026	negative re	104/905	463/18866	1.72E-41	2.44E-39	1.28E-39	FCER1G/HI
BP	GO:00508(	regulation	88/905	332/18866	3.18E-41	4.41E-39	2.32E-39	CD4/HLA-A
BP	GO:00224(	positive re	81/905	279/18866	3.32E-41	4.48E-39	2.36E-39	CD4/HLA-A
BP	GO:00507	regulation	99/905	425/18866	5.73E-41	7.56E-39	3.97E-39	FCER1G/IFI
BP	GO:00421	B cell activ	87/905	328/18866	8.74E-41	1.13E-38	5.91E-39	TGFB1/TFR
BP	GO:00712	cellular res	70/905	208/18866	2.02E-40	2.54E-38	1.33E-38	ICAM1/CX(
BP	GO:19030:	positive re	74/905	235/18866	2.08E-40	2.55E-38	1.34E-38	CD4/HLA-A
BP	GO:00712	cellular res	72/905	222/18866	2.58E-40	3.11E-38	1.63E-38	ICAM1/CX(
BP	GO:00380!	Fc-gamma	59/905	142/18866	2.73E-40	3.21E-38	1.69E-38	FCER1G/HS
BP	GO:00182	peptidyl-ty	92/905	377/18866	7.41E-40	8.52E-38	4.48E-38	CD4/ICAM
BP	GO:00706(	regulation	74/905	240/18866	1.07E-39	1.20E-37	6.32E-38	CD4/HLA-A
BP	GO:00024:	Fc recepto	59/905	145/18866	1.16E-39	1.28E-37	6.72E-38	FCER1G/HS

BP	GO:003809	Fc-epsilon	63/905	169/18866	1.52E-39	1.64E-37	8.61E-38	FCER1G/PS
BP	GO:001810	peptidyl-ty	91/905	374/18866	2.58E-39	2.74E-37	1.44E-37	CD4/ICAM1
BP	GO:000283	positive reg	74/905	251/18866	3.34E-38	3.47E-36	1.82E-36	FCER1G/HL
BP	GO:000268	regulation	68/905	212/18866	9.09E-38	9.27E-36	4.87E-36	CALR/ICAM
BP	GO:000245	humoral im	59/905	155/18866	1.07E-37	1.07E-35	5.63E-36	HLA-DQB1,
BP	GO:007162	granulocyte	54/905	127/18866	1.34E-37	1.32E-35	6.93E-36	FCER1G/CX
BP	GO:005092	positive reg	57/905	144/18866	1.47E-37	1.42E-35	7.46E-36	CALR/CXCL
BP	GO:000281	regulation	61/905	168/18866	1.54E-37	1.46E-35	7.69E-36	AZGP1/B2M
BP	GO:000243	immune re	56/905	139/18866	2.05E-37	1.88E-35	9.89E-36	HSP90AA1,
BP	GO:003809	Fc-gamma	56/905	139/18866	2.05E-37	1.88E-35	9.89E-36	HSP90AA1,
BP	GO:004665	lymphocyte	77/905	283/18866	5.30E-37	4.77E-35	2.51E-35	CD4/HLA-A
BP	GO:003294	mononucle	77/905	286/18866	1.19E-36	1.06E-34	5.55E-35	CD4/HLA-A
BP	GO:005087	positive reg	67/905	214/18866	1.76E-36	1.54E-34	8.08E-35	CD4/HLA-A
BP	GO:009753	granulocyte	57/905	150/18866	2.10E-36	1.80E-34	9.47E-35	FCER1G/CX
BP	GO:003434	response to	65/905	202/18866	3.11E-36	2.62E-34	1.38E-34	B2M/HLA-A
BP	GO:000695	complemen	55/905	141/18866	7.12E-36	5.92E-34	3.11E-34	IGHE/IGHG
BP	GO:000695	complemen	61/905	178/18866	7.47E-36	6.12E-34	3.22E-34	IL1B/CD19,
BP	GO:003009	lymphocyte	86/905	368/18866	9.74E-36	7.87E-34	4.13E-34	B2M/CD4/
BP	GO:003320	tumor necr	60/905	173/18866	1.28E-35	1.01E-33	5.33E-34	LTA/PSMB8
BP	GO:005086	regulation	63/905	195/18866	2.90E-35	2.28E-33	1.20E-33	TGFB1/TFR
BP	GO:007037	ERK1 and E	80/905	325/18866	5.27E-35	4.07E-33	2.14E-33	CD4/ICAM1
BP	GO:000292	regulation	53/905	134/18866	5.65E-35	4.31E-33	2.26E-33	LTA/CXCL1
BP	GO:005067	regulation	66/905	219/18866	8.30E-35	6.24E-33	3.28E-33	CD4/HLA-A
BP	GO:007009	chemokine	44/905	88/18866	1.17E-34	8.65E-33	4.54E-33	CXCL8/CXC
BP	GO:003294	regulation	66/905	221/18866	1.55E-34	1.13E-32	5.93E-33	CD4/HLA-A
BP	GO:000244	production	77/905	306/18866	1.99E-34	1.43E-32	7.54E-33	B2M/FCER
BP	GO:007037	positive reg	65/905	215/18866	2.19E-34	1.56E-32	8.20E-33	CD4/ICAM1
BP	GO:006033	type I inter	45/905	95/18866	4.39E-34	3.05E-32	1.60E-32	HLA-A/HLA
BP	GO:007135	cellular res	45/905	95/18866	4.39E-34	3.05E-32	1.60E-32	HLA-A/HLA
BP	GO:000282	regulation	55/905	153/18866	1.12E-33	7.66E-32	4.03E-32	AZGP1/B2M
BP	GO:199086	response to	45/905	97/18866	1.41E-33	9.40E-32	4.94E-32	CXCL8/CXC
BP	GO:199086	cellular res	45/905	97/18866	1.41E-33	9.40E-32	4.94E-32	CXCL8/CXC
BP	GO:007037	regulation	76/905	306/18866	1.43E-33	9.43E-32	4.95E-32	CD4/ICAM1
BP	GO:000268	positive reg	53/905	142/18866	1.86E-33	1.21E-31	6.38E-32	CALR/ICAM
BP	GO:003434	response to	45/905	99/18866	4.35E-33	2.80E-31	1.47E-31	HLA-A/HLA
BP	GO:000270	regulation	63/905	211/18866	5.30E-33	3.37E-31	1.77E-31	AZGP1/B2M
BP	GO:190210	regulation	73/905	290/18866	1.09E-32	6.88E-31	3.62E-31	CD4/HLA-B
BP	GO:000181	negative re	81/905	360/18866	1.94E-32	1.21E-30	6.34E-31	HLA-F/HSP
BP	GO:007134	cellular res	58/905	182/18866	3.56E-32	2.19E-30	1.15E-30	B2M/HLA-A
BP	GO:003044	regulation	47/905	115/18866	6.48E-32	3.89E-30	2.05E-30	IL1B/CD19,
BP	GO:003260	interferon-	47/905	115/18866	6.48E-32	3.89E-30	2.05E-30	HLA-A/LTA,
BP	GO:005085	B cell recep	50/905	135/18866	1.95E-31	1.16E-29	6.09E-30	CD79A/BTK
BP	GO:007055	response to	61/905	211/18866	4.15E-31	2.44E-29	1.28E-29	ICAM1/PSM
BP	GO:007167	mononucle	43/905	97/18866	4.23E-31	2.46E-29	1.29E-29	CXCL10/CX
BP	GO:003059	neutrophil	44/905	103/18866	6.01E-31	3.46E-29	1.82E-29	FCER1G/CX
BP	GO:003210	negative re	87/905	433/18866	6.71E-31	3.82E-29	2.01E-29	HLA-A/HLA
BP	GO:005073	regulation	67/905	263/18866	2.08E-30	1.17E-28	6.17E-29	CD4/ICAM1
BP	GO:000691	phagocytos	47/905	126/18866	8.80E-30	4.91E-28	2.58E-28	FCER1G/F2
BP	GO:000689	receptor-m	74/905	328/18866	8.90E-30	4.91E-28	2.58E-28	B2M/CALR,
BP	GO:000269	positive reg	61/905	223/18866	1.25E-29	6.84E-28	3.59E-28	AZGP1/B2M

BP	GO:199026	neutrophil	46/905	122/18866	2.05E-29	1.10E-27	5.78E-28	FCER1G/CX
BP	GO:004209	T cell prolif	57/905	195/18866	2.05E-29	1.10E-27	5.78E-28	CD4/HLA-A
BP	GO:000270	regulation	51/905	154/18866	2.50E-29	1.33E-27	6.97E-28	AZGP1/B2M
BP	GO:000268	regulation	46/905	124/18866	4.75E-29	2.49E-27	1.31E-27	CALR/CXCL
BP	GO:004508	positive reg	59/905	213/18866	5.18E-29	2.69E-27	1.41E-27	FCER1G/HL
BP	GO:007066	positive reg	50/905	150/18866	6.30E-29	3.24E-27	1.70E-27	CD4/HLA-A
BP	GO:000269	positive reg	41/905	96/18866	6.48E-29	3.31E-27	1.74E-27	CALR/CXCL
BP	GO:000724	I-kappaB ki	68/905	286/18866	6.89E-29	3.48E-27	1.83E-27	CD4/RELB/
BP	GO:005077	negative re	51/905	159/18866	1.42E-28	7.04E-27	3.70E-27	HLA-A/HLA
BP	GO:005109	positive reg	51/905	159/18866	1.42E-28	7.04E-27	3.70E-27	ICAM1/S10
BP	GO:004212	regulation	52/905	167/18866	2.09E-28	1.03E-26	5.39E-27	CD4/HLA-A
BP	GO:009902	plasma me	47/905	135/18866	3.19E-28	1.55E-26	8.17E-27	FCER1G/F2
BP	GO:005189	positive reg	53/905	178/18866	7.31E-28	3.53E-26	1.86E-26	HSP90AA1/
BP	GO:005087	positive reg	49/905	151/18866	9.23E-28	4.42E-26	2.32E-26	TGFB1/TFR
BP	GO:003264	regulation	41/905	104/18866	2.99E-27	1.42E-25	7.44E-26	HLA-A/LTA/
BP	GO:004349	protein kin	65/905	278/18866	3.30E-27	1.55E-25	8.17E-26	HLA-G/HSP
BP	GO:009719	extrinsic ap	59/905	230/18866	4.57E-27	2.13E-25	1.12E-25	PDIA3/ICAM
BP	GO:005067	positive reg	46/905	136/18866	5.07E-27	2.35E-25	1.23E-25	CD4/HLA-A
BP	GO:001032	membrane	47/905	143/18866	5.92E-27	2.71E-25	1.43E-25	FCER1G/F2
BP	GO:005507	calcium ior	85/905	468/18866	6.04E-27	2.74E-25	1.44E-25	CALR/CD4/
BP	GO:003294	positive reg	46/905	137/18866	7.30E-27	3.29E-25	1.73E-25	CD4/HLA-A
BP	GO:000222	pattern rec	55/905	201/18866	7.95E-27	3.55E-25	1.87E-25	CTSB/CTSL/
BP	GO:004312	regulation	61/905	252/18866	1.82E-26	8.08E-25	4.24E-25	CD4/S100B
BP	GO:000687	cellular cal	83/905	456/18866	2.15E-26	9.45E-25	4.97E-25	CALR/CD4/
BP	GO:007134	cellular res	52/905	183/18866	2.79E-26	1.22E-24	6.40E-25	ICAM1/PSM
BP	GO:004340	regulation	71/905	342/18866	2.97E-26	1.28E-24	6.75E-25	TGFB1/IL1B
BP	GO:000245	T cell medi	40/905	104/18866	3.97E-26	1.70E-24	8.96E-25	AZGP1/B2M
BP	GO:007250	cellular div	86/905	492/18866	4.84E-26	2.06E-24	1.08E-24	CALR/CD4/
BP	GO:000691	phagocyto	37/905	88/18866	6.43E-26	2.72E-24	1.43E-24	IGHE/IGHG
BP	GO:000190	cell killing	50/905	172/18866	8.13E-26	3.41E-24	1.79E-24	AZGP1/B2M
BP	GO:005109	regulation	82/905	455/18866	8.79E-26	3.66E-24	1.92E-24	ICAM1/PSM
BP	GO:005073	positive reg	53/905	195/18866	9.77E-26	4.03E-24	2.12E-24	CD4/ICAM1
BP	GO:000222	toll-like rec	47/905	152/18866	1.22E-25	5.01E-24	2.63E-24	CTSB/CTSL/
BP	GO:005085	T cell recep	54/905	206/18866	2.31E-25	9.41E-24	4.95E-24	CD4/HLA-A
BP	GO:003261	interleukin	42/905	121/18866	2.73E-25	1.10E-23	5.79E-24	MR1/IFNG/
BP	GO:005067	epithelial c	81/905	453/18866	3.05E-25	1.22E-23	6.42E-24	B2M/CXCL1
BP	GO:000282	positive reg	40/905	110/18866	4.92E-25	1.96E-23	1.03E-23	AZGP1/B2M
BP	GO:000236	cytokine pr	39/905	104/18866	5.07E-25	2.00E-23	1.05E-23	B2M/FCER
BP	GO:000720	positive reg	67/905	322/18866	6.92E-25	2.71E-23	1.42E-23	CD4/CXCL1
BP	GO:003021	T cell differ	59/905	253/18866	9.98E-25	3.88E-23	2.04E-23	B2M/CD4/
BP	GO:004340	positive reg	60/905	264/18866	1.68E-24	6.51E-23	3.42E-23	TGFB1/IL1B
BP	GO:005067	regulation	74/905	395/18866	2.06E-24	7.89E-23	4.15E-23	B2M/CXCL1
BP	GO:000190	leukocyte	39/905	108/18866	2.61E-24	9.96E-23	5.23E-23	AZGP1/B2M
BP	GO:007099	neuron dea	70/905	360/18866	3.97E-24	1.50E-22	7.90E-23	HLA-F/HSP
BP	GO:005109	positive reg	60/905	270/18866	5.88E-24	2.21E-22	1.16E-22	ICAM1/S10
BP	GO:005189	regulation	58/905	253/18866	6.36E-24	2.37E-22	1.25E-22	HLA-G/HSP
BP	GO:005072	positive reg	46/905	158/18866	7.06E-24	2.61E-22	1.37E-22	FCER1G/IF
BP	GO:000270	regulation	44/905	144/18866	7.63E-24	2.81E-22	1.48E-22	B2M/FCER
BP	GO:000282	positive reg	38/905	105/18866	9.29E-24	3.39E-22	1.78E-22	AZGP1/B2M
BP	GO:000254	monocyte	31/905	67/18866	1.79E-23	6.49E-22	3.41E-22	CXCL10/CX

BP	GO:00421(	positive re{	37/905	101/18866	2.20E-23	7.93E-22	4.17E-22	CD4/HLA-A
BP	GO:00300!	myeloid ce	76/905	431/18866	2.41E-23	8.63E-22	4.54E-22	B2M/CD4/
BP	GO:00607!	regulation	50/905	196/18866	5.42E-23	1.93E-21	1.01E-21	HSP90AB1/
BP	GO:00425!	antigen prc	33/905	80/18866	6.20E-23	2.18E-21	1.15E-21	B2M/CALR/
BP	GO:00514!	regulation	68/905	357/18866	6.21E-23	2.18E-21	1.15E-21	CD4/CXCL1
BP	GO:00313	regulation	35/905	92/18866	7.82E-23	2.73E-21	1.43E-21	AZGP1/B2M
BP	GO:00326	regulation	45/905	159/18866	7.97E-23	2.76E-21	1.45E-21	FCER1G/HL
BP	GO:00027(	positive re{	42/905	138/18866	9.65E-23	3.32E-21	1.75E-21	AZGP1/B2M
BP	GO:00024	antigen prc	36/905	99/18866	1.17E-22	4.00E-21	2.10E-21	AZGP1/B2M
BP	GO:00326	interleukin	37/905	106/18866	1.58E-22	5.36E-21	2.82E-21	MR1/IFNG/
BP	GO:00326	interleukin	46/905	170/18866	2.09E-22	7.06E-21	3.71E-21	FCER1G/HL
BP	GO:00025	myeloid leı	51/905	210/18866	2.22E-22	7.47E-21	3.92E-21	CD4/FCER1
BP	GO:00704!	response t	71/905	396/18866	2.66E-22	8.88E-21	4.67E-21	ICAM1/LTA
BP	GO:00016(	response t	67/905	359/18866	4.26E-22	1.41E-20	7.43E-21	ICAM1/LTA
BP	GO:19012	regulation	63/905	321/18866	4.88E-22	1.61E-20	8.46E-21	HLA-F/HSP
BP	GO:00362!	response t	68/905	371/18866	5.92E-22	1.94E-20	1.02E-20	ICAM1/LTA
BP	GO:00603	interferon-	34/905	91/18866	6.43E-22	2.09E-20	1.10E-20	B2M/HLA-/
BP	GO:00019!	regulation	47/905	183/18866	8.07E-22	2.61E-20	1.37E-20	HSP90AB1/
BP	GO:00485	negative re	36/905	104/18866	8.19E-22	2.64E-20	1.39E-20	SLPI/APOB
BP	GO:00199	second-me	76/905	456/18866	8.51E-22	2.72E-20	1.43E-20	CD4/CXCL8
BP	GO:00026!	negative re	39/905	124/18866	8.66E-22	2.75E-20	1.45E-20	HLA-A/HLA
BP	GO:00719(	positive re{	65/905	345/18866	1.07E-21	3.37E-20	1.77E-20	HSP90AB1/
BP	GO:00019	regulation	31/905	75/18866	1.18E-21	3.68E-20	1.93E-20	AZGP1/B2M
BP	GO:00024	antigen prc	31/905	75/18866	1.18E-21	3.68E-20	1.93E-20	B2M/CALR/
BP	GO:00324	regulation	39/905	125/18866	1.20E-21	3.73E-20	1.96E-20	RELB/TLR2/
BP	GO:00512!	negative re	43/905	154/18866	1.30E-21	4.02E-20	2.11E-20	HLA-F/HLA
BP	GO:00506	positive re{	50/905	211/18866	1.86E-21	5.70E-20	3.00E-20	CXCL12/MI
BP	GO:19035!	regulation	37/905	113/18866	2.02E-21	6.16E-20	3.24E-20	FCER1G/IFI
BP	GO:00022!	lymphocyt	47/905	187/18866	2.16E-21	6.57E-20	3.45E-20	CD1C/FCEF
BP	GO:00326(	type I inter	39/905	127/18866	2.29E-21	6.92E-20	3.64E-20	RELB/TLR2/
BP	GO:19037(	positive re{	49/905	204/18866	2.51E-21	7.56E-20	3.97E-20	CD4/HLA-G
BP	GO:00431	positive re{	47/905	188/18866	2.75E-21	8.22E-20	4.32E-20	CD4/S100E
BP	GO:00027	regulation	32/905	83/18866	3.37E-21	1.00E-19	5.27E-20	B2M/FCER
BP	GO:00439(	regulation	52/905	231/18866	3.49E-21	1.03E-19	5.42E-20	CD4/SLPI/C
BP	GO:00456	regulation	46/905	181/18866	3.54E-21	1.04E-19	5.46E-20	HLA-G/IFN(
BP	GO:00313	negative re	56/905	269/18866	5.49E-21	1.61E-19	8.44E-20	HLA-A/HLA
BP	GO:00197	antimicrob	40/905	137/18866	5.60E-21	1.63E-19	8.55E-20	HLA-A/CXC
BP	GO:00026!	negative re	46/905	184/18866	7.35E-21	2.13E-19	1.12E-19	HLA-F/HLA
BP	GO:00726	lymphocyt	37/905	117/18866	7.87E-21	2.26E-19	1.19E-19	ICAM1/CX(
BP	GO:00326!	regulation	36/905	111/18866	1.02E-20	2.91E-19	1.53E-19	IFNG/ORM
BP	GO:00071(	negative re	59/905	301/18866	1.12E-20	3.17E-19	1.67E-19	HLA-G/CXC
BP	GO:20012	regulation	43/905	162/18866	1.13E-20	3.21E-19	1.69E-19	PDIA3/ICAI
BP	GO:19039(	negative re	32/905	86/18866	1.21E-20	3.42E-19	1.79E-19	SLPI/APOB
BP	GO:00321	activation (	62/905	331/18866	1.31E-20	3.66E-19	1.92E-19	IL1B/TNF/T
BP	GO:20012	regulation	70/905	413/18866	1.43E-20	3.99E-19	2.10E-19	PDIA3/ICAI
BP	GO:00717(	tumor necı	37/905	119/18866	1.52E-20	4.21E-19	2.21E-19	FCER1G/IFI
BP	GO:00027(	positive re{	35/905	106/18866	1.80E-20	4.98E-19	2.61E-19	AZGP1/B2M
BP	GO:00140(	positive re{	32/905	88/18866	2.75E-20	7.51E-19	3.95E-19	TNF/F2R/C
BP	GO:00327!	positive re{	32/905	88/18866	2.75E-20	7.51E-19	3.95E-19	FCER1G/IFI
BP	GO:00508(	negative re	48/905	207/18866	3.19E-20	8.66E-19	4.55E-19	HLA-F/HLA

BP	GO:003265	regulation	26/905	55/18866	3.37E-20	9.12E-19	4.79E-19	HLA-B/HLA
BP	GO:003268	regulation	35/905	110/18866	7.13E-20	1.92E-18	1.01E-18	FCER1G/TL
BP	GO:005076	regulation	33/905	97/18866	7.77E-20	2.08E-18	1.09E-18	CALR/FCER
BP	GO:000270	regulation	29/905	73/18866	9.14E-20	2.43E-18	1.28E-18	AZGP1/B2M
BP	GO:200011	regulation	51/905	239/18866	1.03E-19	2.73E-18	1.44E-18	S100A9/S1
BP	GO:003261	interleukin	26/905	57/18866	1.06E-19	2.79E-18	1.46E-18	HLA-B/HLA
BP	GO:001988	antigen pro	50/905	232/18866	1.51E-19	3.97E-18	2.09E-18	AZGP1/B2M
BP	GO:004331	neutrophil	75/905	487/18866	1.90E-19	4.96E-18	2.61E-18	B2M/CTSB/
BP	GO:009769	receptor si	43/905	174/18866	2.25E-19	5.84E-18	3.07E-18	IFNG/IFNL1
BP	GO:000228	neutrophil	75/905	490/18866	2.73E-19	7.06E-18	3.71E-18	B2M/CTSB/
BP	GO:190210	positive re	41/905	159/18866	2.89E-19	7.45E-18	3.91E-18	CD4/HLA-G
BP	GO:001905	viral life cy	61/905	341/18866	2.96E-19	7.58E-18	3.99E-18	CD4/CTSB/
BP	GO:003264	tumor necr	35/905	116/18866	4.94E-19	1.26E-17	6.63E-18	FCER1G/TL
BP	GO:001972	calcium-me	48/905	222/18866	7.10E-19	1.80E-17	9.47E-18	CD4/CXCL8
BP	GO:004507	negative re	26/905	61/18866	8.76E-19	2.21E-17	1.16E-17	SLPI/APOB
BP	GO:190134	regulation	70/905	444/18866	8.78E-19	2.21E-17	1.16E-17	HLA-G/ERA
BP	GO:000725	receptor si	41/905	164/18866	9.89E-19	2.48E-17	1.30E-17	IFNG/IFNL1
BP	GO:190390	regulation	40/905	157/18866	1.27E-18	3.15E-17	1.66E-17	CD4/SLPI/C
BP	GO:190401	positive re	49/905	235/18866	1.52E-18	3.77E-17	1.98E-17	ERAP1/CXC
BP	GO:001406	regulation	36/905	127/18866	1.53E-18	3.77E-17	1.98E-17	TNF/F2R/C
BP	GO:005079	regulation	47/905	217/18866	1.54E-18	3.77E-17	1.98E-17	CD4/SLPI/C
BP	GO:005123	maintenan	58/905	324/18866	2.22E-18	5.44E-17	2.86E-17	CALR/CD4/
BP	GO:003129	lymphocyte	25/905	58/18866	2.95E-18	7.18E-17	3.77E-17	SRC/CD40L
BP	GO:004586	positive re	62/905	370/18866	4.36E-18	1.06E-16	5.55E-17	IFNG/PSMC
BP	GO:000166	ameboidal-	72/905	481/18866	5.18E-18	1.25E-16	6.57E-17	CALR/IFNG
BP	GO:004824	lymphocyte	26/905	65/18866	6.00E-18	1.44E-16	7.58E-17	CXCL14/CX
BP	GO:003265	regulation	31/905	97/18866	8.11E-18	1.94E-16	1.02E-16	IFNG/ORM
BP	GO:000252	acute infla	33/905	111/18866	8.38E-18	2.00E-16	1.05E-16	FCER1G/IC
BP	GO:000193	endothelia	44/905	199/18866	8.46E-18	2.01E-16	1.05E-16	CXCL12/TN
BP	GO:004860	reproducti	68/905	443/18866	1.19E-17	2.82E-16	1.48E-16	CTSB/HSPA
BP	GO:000237	immunoglo	45/905	210/18866	1.29E-17	3.03E-16	1.59E-16	HLA-DQB1/
BP	GO:003134	positive re	25/905	61/18866	1.29E-17	3.03E-16	1.59E-16	AZGP1/B2M
BP	GO:000193	regulation	42/905	184/18866	1.38E-17	3.21E-16	1.69E-16	CXCL12/TN
BP	GO:003806	NIK/NF-kap	42/905	184/18866	1.38E-17	3.21E-16	1.69E-16	CALR/PSM
BP	GO:005091	positive ch	26/905	67/18866	1.48E-17	3.41E-16	1.79E-16	CXCL8/CXC
BP	GO:000193	positive re	33/905	113/18866	1.53E-17	3.52E-16	1.85E-16	CXCL12/VE
BP	GO:003129	T cell costi	24/905	56/18866	1.65E-17	3.78E-16	1.98E-16	SRC/CD40L
BP	GO:004800	antigen pro	43/905	194/18866	1.82E-17	4.16E-16	2.18E-16	AZGP1/B2M
BP	GO:006145	reproducti	68/905	447/18866	1.93E-17	4.38E-16	2.30E-16	CTSB/HSPA
BP	GO:004576	regulation	64/905	403/18866	2.00E-17	4.52E-16	2.38E-16	HLA-G/ERA
BP	GO:190489	positive re	30/905	93/18866	2.05E-17	4.62E-16	2.43E-16	IFNG/IFNL1
BP	GO:004506	regulation	31/905	100/18866	2.18E-17	4.89E-16	2.57E-16	SLPI/CXCL8
BP	GO:007188	leukocyte a	32/905	108/18866	2.95E-17	6.59E-16	3.46E-16	CTSL/FCER
BP	GO:001406	phosphatic	38/905	154/18866	3.00E-17	6.67E-16	3.51E-16	TNF/F2R/C
BP	GO:004328	regulation	45/905	215/18866	3.37E-17	7.46E-16	3.92E-16	S100A9/S1
BP	GO:003260	chemokine	27/905	75/18866	3.39E-17	7.47E-16	3.93E-16	S100A9/S1
BP	GO:004563	regulation	50/905	263/18866	3.97E-17	8.74E-16	4.59E-16	B2M/CD4/
BP	GO:004576	positive re	44/905	208/18866	4.98E-17	1.09E-15	5.74E-16	ERAP1/CXC
BP	GO:003272	positive re	25/905	65/18866	7.98E-17	1.74E-15	9.15E-16	HLA-A/LTA
BP	GO:005104	positive re	57/905	340/18866	9.89E-17	2.15E-15	1.13E-15	FCER1G/HL

BP	GO:190489	regulation	37/905	151/18866	1.01E-16	2.18E-15	1.14E-15	IFNG/IFNL1
BP	GO:000283	negative re	30/905	98/18866	1.06E-16	2.29E-15	1.20E-15	HLA-A/HLA
BP	GO:000221	activation (	36/905	144/18866	1.31E-16	2.82E-15	1.48E-15	FCER1G/PS
BP	GO:004877	tissue remo	40/905	178/18866	1.47E-16	3.14E-15	1.65E-15	TGFB1/NO:
BP	GO:200123	negative re	46/905	233/18866	1.63E-16	3.46E-15	1.82E-15	ICAM1/PSM
BP	GO:005254	regulation	68/905	466/18866	1.73E-16	3.67E-15	1.93E-15	PSMB8/PSI
BP	GO:004211	macrophag	30/905	101/18866	2.70E-16	5.70E-15	2.99E-15	IFNG/TLR2,
BP	GO:005076	positive re	25/905	68/18866	2.82E-16	5.94E-15	3.12E-15	CALR/FCER
BP	GO:007167	regulation	22/905	51/18866	3.10E-16	6.49E-15	3.41E-15	CXCL10/CX
BP	GO:002240	negative re	41/905	191/18866	3.42E-16	7.13E-15	3.75E-15	HLA-G/CXC
BP	GO:001095	positive re	42/905	201/18866	4.12E-16	8.56E-15	4.50E-15	PSME2/PSI
BP	GO:007259	reactive ox	51/905	288/18866	4.19E-16	8.68E-15	4.56E-15	HSP90AA1,
BP	GO:000191	positive re	22/905	52/18866	5.13E-16	1.06E-14	5.56E-15	AZGP1/B2I
BP	GO:004642	regulation	35/905	142/18866	5.61E-16	1.15E-14	6.06E-15	IFNG/IFNL1
BP	GO:004249	response t	61/905	397/18866	5.73E-16	1.17E-14	6.17E-15	B2M/CALR,
BP	GO:001988	antigen pro	40/905	185/18866	6.06E-16	1.24E-14	6.50E-15	B2M/CALR,
BP	GO:004642	positive re	28/905	90/18866	6.99E-16	1.42E-14	7.47E-15	IFNG/IFNL1
BP	GO:001907	viral genon	33/905	127/18866	7.14E-16	1.45E-14	7.60E-15	SLPI/CXCL8
BP	GO:190353	positive re	53/905	313/18866	7.87E-16	1.59E-14	8.34E-15	FCER1G/HL
BP	GO:005254	regulation	64/905	434/18866	8.29E-16	1.67E-14	8.76E-15	PSMB8/PSI
BP	GO:004854	response t	56/905	346/18866	9.20E-16	1.84E-14	9.68E-15	CALR/ICAM
BP	GO:000803	cell recogn	44/905	226/18866	1.28E-15	2.55E-14	1.34E-14	PAEP/CCR7
BP	GO:001095	positive re	39/905	181/18866	1.59E-15	3.16E-14	1.66E-14	PSME2/PSI
BP	GO:000270	positive re	29/905	100/18866	1.72E-15	3.41E-14	1.79E-14	B2M/FCER
BP	GO:200010	regulation	27/905	86/18866	1.79E-15	3.52E-14	1.85E-14	FCER1G/CX
BP	GO:004866	regulation	38/905	173/18866	1.88E-15	3.71E-14	1.95E-14	IFNG/MMF
BP	GO:000191	T cell medi	20/905	44/18866	2.00E-15	3.91E-14	2.05E-14	AZGP1/B2I
BP	GO:004208	T-helper 1	20/905	44/18866	2.00E-15	3.91E-14	2.05E-14	RELB/XCL1,
BP	GO:003109	stress-activ	51/905	300/18866	2.37E-15	4.61E-14	2.42E-14	IL1B/TNF/T
BP	GO:000228	T cell activ	30/905	109/18866	2.72E-15	5.27E-14	2.77E-14	CD1C/FCEF
BP	GO:004865	smooth mu	38/905	175/18866	2.81E-15	5.43E-14	2.85E-14	IFNG/MMF
BP	GO:000271	positive re	21/905	50/18866	2.85E-15	5.49E-14	2.89E-14	AZGP1/B2I
BP	GO:190121	negative re	42/905	212/18866	3.01E-15	5.77E-14	3.03E-14	HLA-F/HSP
BP	GO:000191	regulation	18/905	35/18866	3.22E-15	6.12E-14	3.22E-14	AZGP1/B2I
BP	GO:003273	positive re	18/905	35/18866	3.22E-15	6.12E-14	3.22E-14	HLA-G/IFN
BP	GO:003264	regulation	24/905	68/18866	3.28E-15	6.23E-14	3.27E-14	TLR2/IL1B/
BP	GO:000150	ossificatior	61/905	412/18866	3.34E-15	6.32E-14	3.32E-14	PSMC2/TG
BP	GO:004558	regulation	35/905	150/18866	3.45E-15	6.51E-14	3.42E-14	HLA-G/IFN
BP	GO:004582	negative re	23/905	62/18866	3.50E-15	6.58E-14	3.46E-14	HLA-A/HLA
BP	GO:004343	response t	64/905	447/18866	3.52E-15	6.58E-14	3.46E-14	ICAM1/CX
BP	GO:200105	positive re	35/905	151/18866	4.29E-15	8.00E-14	4.21E-14	S100A9/S1
BP	GO:000279	regulation	58/905	381/18866	4.67E-15	8.68E-14	4.56E-14	IFNG/S100
BP	GO:000282	regulation	16/905	27/18866	5.48E-15	1.01E-13	5.33E-14	XCL1/IL1B/
BP	GO:005140	neuron apo	45/905	245/18866	5.80E-15	1.07E-13	5.62E-14	HSPA5/HSF
BP	GO:003287	positive re	37/905	170/18866	6.05E-15	1.11E-13	5.84E-14	IL1B/TNF/T
BP	GO:005140	stress-activ	49/905	286/18866	6.17E-15	1.13E-13	5.94E-14	IL1B/TNF/T
BP	GO:003287	regulation	44/905	237/18866	7.80E-15	1.42E-13	7.48E-14	IL1B/TNF/T
BP	GO:190303	negative re	33/905	137/18866	7.92E-15	1.44E-13	7.58E-14	HLA-G/CXC
BP	GO:004801	vascular er	28/905	98/18866	8.07E-15	1.46E-13	7.69E-14	HSP90AA1,
BP	GO:015007	neuroinflar	25/905	77/18866	8.19E-15	1.48E-13	7.79E-14	IFNG/MMF

BP	GO:00703(	positive re{	37/905	172/18866	8.97E-15	1.62E-13	8.49E-14	IL1B/TNF/T
BP	GO:00466:	alpha-beta	34/905	146/18866	9.13E-15	1.64E-13	8.62E-14	CTSL/HLA-/
BP	GO:00302:	macrophag	20/905	47/18866	9.67E-15	1.73E-13	9.10E-14	CD4/IFNG/
BP	GO:00301(	natural kill	26/905	85/18866	1.20E-14	2.15E-13	1.13E-13	HLA-F/MIC
BP	GO:00316(	lipopolysac	22/905	59/18866	1.23E-14	2.19E-13	1.15E-13	TGFB1/TLR
BP	GO:00703(	regulation	44/905	240/18866	1.25E-14	2.22E-13	1.17E-13	IL1B/TNF/T
BP	GO:00480:	phosphatic	39/905	192/18866	1.26E-14	2.22E-13	1.17E-13	TNF/F2R/C
BP	GO:00326	regulation	20/905	48/18866	1.58E-14	2.79E-13	1.47E-13	RELB/TLR2,
BP	GO:00022:	natural kill	23/905	66/18866	1.70E-14	2.99E-13	1.57E-13	HLA-A/HLA
BP	GO:00027!	innate imn	30/905	117/18866	2.20E-14	3.84E-13	2.02E-13	FCER1G/PS
BP	GO:00488(	stem cell d	46/905	264/18866	2.22E-14	3.88E-13	2.04E-13	PSMB8/PSI
BP	GO:00330(	muscle cell	44/905	244/18866	2.32E-14	4.03E-13	2.12E-13	IFNG/MMF
BP	GO:00024:	antigen prc	37/905	177/18866	2.34E-14	4.05E-13	2.13E-13	B2M/CALR,
BP	GO:00726:	T cell migra	23/905	67/18866	2.48E-14	4.28E-13	2.25E-13	ICAM1/CX(
BP	GO:00480:	inositol lipi	39/905	196/18866	2.56E-14	4.41E-13	2.32E-13	TNF/F2R/C
BP	GO:00305:	intracellula	46/905	265/18866	2.57E-14	4.41E-13	2.32E-13	CALR/DDX!
BP	GO:00508(	negative re	30/905	118/18866	2.81E-14	4.81E-13	2.53E-13	HLA-G/XCL
BP	GO:00027:	positive re{	21/905	55/18866	2.84E-14	4.84E-13	2.54E-13	B2M/FCER
BP	GO:00901:	tissue migr	56/905	374/18866	2.89E-14	4.91E-13	2.58E-13	CALR/IFNG
BP	GO:00516!	maintenan	41/905	216/18866	2.98E-14	5.05E-13	2.66E-13	CALR/CD4/
BP	GO:00106:	epithelial c	55/905	365/18866	3.76E-14	6.36E-13	3.34E-13	CALR/IFNG
BP	GO:00716!	protein loc	64/905	470/18866	3.89E-14	6.55E-13	3.44E-13	IFNG/TGFB
BP	GO:00326(	interferon-	20/905	50/18866	4.07E-14	6.84E-13	3.59E-13	RELB/TLR2,
BP	GO:00072(	tyrosine ph	26/905	89/18866	4.10E-14	6.87E-13	3.61E-13	IFNG/IFNL1
BP	GO:00421(	B cell prolit	27/905	97/18866	4.95E-14	8.26E-13	4.34E-13	TFRC/CD4(
BP	GO:20003:	regulation	39/905	200/18866	5.11E-14	8.50E-13	4.46E-13	HSP90AA1,
BP	GO:00901:	epithelium	55/905	368/18866	5.34E-14	8.86E-13	4.66E-13	CALR/IFNG
BP	GO:00456:	positive re{	28/905	105/18866	5.53E-14	9.15E-13	4.81E-13	HLA-G/IFN(
BP	GO:00093(	protein sec	63/905	462/18866	5.80E-14	9.56E-13	5.02E-13	IFNG/TGFB
BP	GO:00022:	stimulatory	29/905	113/18866	5.82E-14	9.56E-13	5.02E-13	FCER1G/PS
BP	GO:00715:	semaphori	18/905	40/18866	6.40E-14	1.05E-12	5.51E-13	SEMA3A/S
BP	GO:00355!	establishm	63/905	463/18866	6.42E-14	1.05E-12	5.51E-13	IFNG/TGFB
BP	GO:00432:	positive re{	47/905	284/18866	8.47E-14	1.38E-12	7.25E-13	CD4/HSPA:
BP	GO:00027(	regulation	30/905	123/18866	9.29E-14	1.50E-12	7.91E-13	CD4/IFNG/
BP	GO:20012:	negative re	28/905	107/18866	9.29E-14	1.50E-12	7.91E-13	ICAM1/IL1I
BP	GO:00506!	regulation	24/905	78/18866	1.09E-13	1.76E-12	9.26E-13	MICB/MMI
BP	GO:00425!	superoxide	17/905	36/18866	1.18E-13	1.90E-12	1.00E-12	TGFB1/NO:
BP	GO:00022:	innate imn	29/905	116/18866	1.21E-13	1.94E-12	1.02E-12	FCER1G/PS
BP	GO:00003(	response t	42/905	235/18866	1.25E-13	1.99E-12	1.05E-12	LCN2/MMI
BP	GO:00425(	regulation	25/905	86/18866	1.44E-13	2.29E-12	1.21E-12	IFNG/IFNL1
BP	GO:00331:	positive re{	28/905	109/18866	1.54E-13	2.45E-12	1.29E-12	HSP90AA1,
BP	GO:00521:	movement	35/905	169/18866	1.58E-13	2.51E-12	1.32E-12	CD4/CTSB/
BP	GO:00509:	negative re	22/905	66/18866	1.82E-13	2.88E-12	1.51E-12	CXCL13/WI
BP	GO:19012:	positive re{	24/905	80/18866	2.04E-13	3.22E-12	1.69E-12	CALR/TLR2
BP	GO:00619(	glial cell ac	21/905	60/18866	2.14E-13	3.37E-12	1.77E-12	IFNG/TLR2,
BP	GO:00160	cell growth	64/905	490/18866	2.72E-13	4.26E-12	2.24E-12	HSP90AA1,
BP	GO:00324{	positive re{	23/905	74/18866	2.80E-13	4.37E-12	2.30E-12	TLR2/DDX5
BP	GO:19034(	reactive ox	30/905	128/18866	2.88E-13	4.48E-12	2.36E-12	HSP90AA1,
BP	GO:00019:	positive re{	15/905	28/18866	3.05E-13	4.74E-12	2.49E-12	AZGP1/B2I
BP	GO:00620:	regulation	61/905	456/18866	3.49E-13	5.41E-12	2.84E-12	IFNG/NFYA

BP	GO:00517(	interaction	39/905	212/18866	3.63E-13	5.60E-12	2.94E-12	CD4/CTSB/
BP	GO:00198	antigen prc	14/905	24/18866	3.88E-13	5.97E-12	3.14E-12	AZGP1/B2M
BP	GO:00463	positive re	31/905	138/18866	3.93E-13	6.03E-12	3.17E-12	IL1B/TNF/T
BP	GO:19021(	negative re	27/905	105/18866	4.08E-13	6.25E-12	3.28E-12	HLA-G/PGL
BP	GO:00420	gliogenesis	48/905	307/18866	4.09E-13	6.26E-12	3.29E-12	IFNG/LTA/S
BP	GO:00444(	entry into I	32/905	147/18866	4.12E-13	6.28E-12	3.30E-12	CD4/CTSB/
BP	GO:00507(	regulation	52/905	352/18866	4.23E-13	6.40E-12	3.37E-12	IFNG/TGFB
BP	GO:00069	response t	61/905	458/18866	4.24E-13	6.40E-12	3.37E-12	MICB/LCN2
BP	GO:00072	JNK cascad	39/905	213/18866	4.24E-13	6.40E-12	3.37E-12	IL1B/TNF/T
BP	GO:00463	regulation	36/905	184/18866	4.34E-13	6.53E-12	3.43E-12	IL1B/TNF/T
BP	GO:00481	regulation	24/905	83/18866	5.03E-13	7.56E-12	3.97E-12	LTA/S100A
BP	GO:20003	positive re	27/905	106/18866	5.23E-13	7.83E-12	4.11E-12	HSP90AA1,
BP	GO:00017	microglial	19/905	50/18866	5.35E-13	7.97E-12	4.19E-12	IFNG/TLR2,
BP	GO:00022	leukocyte a	19/905	50/18866	5.35E-13	7.97E-12	4.19E-12	IFNG/TLR2,
BP	GO:00181(	peptidyl-se	48/905	310/18866	5.92E-13	8.79E-12	4.62E-12	HSP90AA1,
BP	GO:00507	negative re	37/905	196/18866	6.46E-13	9.54E-12	5.02E-12	PGLYRP1/A
BP	GO:00326	interleukin	21/905	63/18866	6.46E-13	9.54E-12	5.02E-12	FCER1G/XC
BP	GO:00622(	regulation	26/905	99/18866	6.50E-13	9.58E-12	5.03E-12	TLR2/LBP/L
BP	GO:00481	fibroblast	24/905	84/18866	6.73E-13	9.89E-12	5.20E-12	LTA/S100A
BP	GO:00024	antigen prc	12/905	17/18866	6.85E-13	1.00E-11	5.26E-12	AZGP1/B2M
BP	GO:00198	antigen prc	12/905	17/18866	6.85E-13	1.00E-11	5.26E-12	AZGP1/B2M
BP	GO:19035	negative re	20/905	57/18866	7.59E-13	1.10E-11	5.80E-12	LBP/LTF/AI
BP	GO:00226	gland morp	29/905	124/18866	7.60E-13	1.10E-11	5.80E-12	TGFB1/TNF
BP	GO:00481	positive re	19/905	51/18866	8.14E-13	1.18E-11	6.21E-12	S100A6/PN
BP	GO:00326	regulation	14/905	25/18866	8.42E-13	1.22E-11	6.40E-12	DDX58/IRF
BP	GO:00431	receptor m	37/905	198/18866	8.92E-13	1.29E-11	6.76E-12	FCER1G/IFI
BP	GO:00509	induction c	11/905	14/18866	9.30E-13	1.34E-11	7.03E-12	CXCL8/CXC
BP	GO:00432	positive re	30/905	134/18866	1.03E-12	1.48E-11	7.78E-12	S100A9/S1
BP	GO:00192	regulation	58/905	431/18866	1.05E-12	1.51E-11	7.91E-12	IFNG/NFYA
BP	GO:00435	regulation	20/905	58/18866	1.11E-12	1.58E-11	8.30E-12	TGFB1/SOC
BP	GO:00015	regulation	57/905	420/18866	1.16E-12	1.66E-11	8.71E-12	PSMD10/C
BP	GO:00027(	negative re	19/905	52/18866	1.23E-12	1.74E-11	9.15E-12	HLA-A/HLA
BP	GO:00400	negative re	55/905	397/18866	1.27E-12	1.80E-11	9.44E-12	CALR/CXCL
BP	GO:00331	regulation	31/905	145/18866	1.60E-12	2.25E-11	1.18E-11	HSP90AA1,
BP	GO:00381	ERBB signa	31/905	145/18866	1.60E-12	2.25E-11	1.18E-11	HSP90AA1,
BP	GO:00519	regulation	42/905	253/18866	1.62E-12	2.28E-11	1.20E-11	CD4/HSPA
BP	GO:00435	endothelia	45/905	286/18866	1.79E-12	2.51E-11	1.32E-11	CALR/CXCL
BP	GO:00324	negative re	18/905	47/18866	1.88E-12	2.61E-11	1.37E-11	RELB/DDX5
BP	GO:00509	negative cl	18/905	47/18866	1.88E-12	2.61E-11	1.37E-11	ITGAV/WN
BP	GO:00027	positive re	36/905	193/18866	1.92E-12	2.67E-11	1.40E-11	IFNG/S100
BP	GO:00016	temperatu	34/905	174/18866	2.02E-12	2.80E-11	1.47E-11	LCN2/IL1B/
BP	GO:00435	regulation	38/905	214/18866	2.27E-12	3.14E-11	1.65E-11	HSP90AB1,
BP	GO:00327	positive re	20/905	60/18866	2.29E-12	3.16E-11	1.66E-11	IFNG/ORM
BP	GO:00182(	peptidyl-se	49/905	333/18866	2.36E-12	3.24E-11	1.71E-11	HSP90AA1,
BP	GO:00714	cellular res	40/905	235/18866	2.44E-12	3.34E-11	1.76E-11	ICAM1/PSM
BP	GO:00341	regulation	22/905	74/18866	2.53E-12	3.46E-11	1.82E-11	TLR2/LBP/L
BP	GO:00357	CD4-positiv	25/905	97/18866	2.86E-12	3.89E-11	2.04E-11	CTSL/IFNG,
BP	GO:00466	regulation	25/905	97/18866	2.86E-12	3.89E-11	2.04E-11	HLA-A/IFN(
BP	GO:00075	female pre	36/905	196/18866	3.09E-12	4.19E-11	2.20E-11	CALR/CTSB
BP	GO:00422	natural kill	20/905	61/18866	3.26E-12	4.42E-11	2.32E-11	HLA-A/HLA

BP	GO:003016	platelet act	32/905	158/18866	3.29E-12	4.45E-11	2.34E-11	FCER1G/CD
BP	GO:004884	axon exten	16/905	37/18866	3.44E-12	4.63E-11	2.43E-11	CXCL12/VE
BP	GO:190228	neuron prc	16/905	37/18866	3.44E-12	4.63E-11	2.43E-11	CXCL12/VE
BP	GO:003260	interferon-	14/905	27/18866	3.46E-12	4.65E-11	2.44E-11	DDX58/IRF
BP	GO:006219	cellular res	51/905	360/18866	3.63E-12	4.86E-11	2.55E-11	RELB/LCN2
BP	GO:004884	regulation	15/905	32/18866	3.85E-12	5.14E-11	2.70E-11	CXCL12/VE
BP	GO:007400	axonogene	61/905	482/18866	3.96E-12	5.27E-11	2.77E-11	HSP90AA1/
BP	GO:003629	cellular res	38/905	218/18866	4.09E-12	5.43E-11	2.85E-11	ICAM1/PSM
BP	GO:007145	cellular res	37/905	208/18866	4.19E-12	5.55E-11	2.92E-11	ICAM1/PSM
BP	GO:003162	receptor in	27/905	115/18866	4.25E-12	5.63E-11	2.96E-11	FCER1G/CX
BP	GO:200010	negative re	18/905	49/18866	4.33E-12	5.71E-11	3.00E-11	FCER1G/CX
BP	GO:004269	ovulation c	21/905	69/18866	4.80E-12	6.31E-11	3.32E-11	HSPA5/SRC
BP	GO:000961	response t	37/905	209/18866	4.86E-12	6.38E-11	3.35E-11	CXCL10/CX
BP	GO:003293	positive re	12/905	19/18866	5.10E-12	6.68E-11	3.51E-11	TGFB1/MA
BP	GO:004620	nitric oxide	23/905	84/18866	5.42E-12	7.07E-11	3.72E-11	HSP90AA1/
BP	GO:200040	positive re	16/905	38/18866	5.68E-12	7.40E-11	3.89E-11	CXCL14/CX
BP	GO:005127	negative re	54/905	400/18866	5.73E-12	7.44E-11	3.91E-11	CALR/CXCL
BP	GO:005122	positive re	50/905	354/18866	6.64E-12	8.61E-11	4.52E-11	HSP90AB1/
BP	GO:000681	calcium ior	56/905	426/18866	6.78E-12	8.78E-11	4.61E-11	CD4/HSPA2
BP	GO:005109	regulation	51/905	367/18866	7.56E-12	9.76E-11	5.13E-11	B2M/HSP9
BP	GO:200002	regulation	41/905	254/18866	7.59E-12	9.77E-11	5.13E-11	PSMB8/PSI
BP	GO:004253	positive re	21/905	71/18866	8.88E-12	1.14E-10	5.99E-11	IFNG/IFNL1
BP	GO:190303	regulation	34/905	183/18866	8.89E-12	1.14E-10	5.99E-11	FCER1G/PL
BP	GO:200040	regulation	20/905	64/18866	8.94E-12	1.14E-10	6.01E-11	CXCL14/CX
BP	GO:007066	negative re	23/905	86/18866	9.25E-12	1.18E-10	6.20E-11	HLA-G/XCL
BP	GO:004887	homeostas	41/905	256/18866	9.82E-12	1.25E-10	6.57E-11	B2M/FCER
BP	GO:009858	detection c	13/905	24/18866	9.99E-12	1.27E-10	6.67E-11	HLA-A/HLA
BP	GO:190122	regulation	27/905	119/18866	1.01E-11	1.27E-10	6.70E-11	CALR/TMSI
BP	GO:004544	fat cell diff	39/905	235/18866	1.03E-11	1.30E-10	6.84E-11	PSMB8/ERA
BP	GO:000680	nitric oxide	22/905	79/18866	1.08E-11	1.36E-10	7.17E-11	HSP90AA1/
BP	GO:200105	reactive nit	23/905	87/18866	1.20E-11	1.51E-10	7.94E-11	HSP90AA1/
BP	GO:000018	activation c	31/905	156/18866	1.21E-11	1.51E-10	7.95E-11	IL1B/TNF/N
BP	GO:007049	interleukin	25/905	103/18866	1.21E-11	1.51E-10	7.95E-11	PSMB8/PSI
BP	GO:000254	chronic infl	12/905	20/18866	1.22E-11	1.52E-10	8.01E-11	LTA/CXCL1
BP	GO:006038	pathway-re	20/905	65/18866	1.23E-11	1.54E-10	8.08E-11	TGFB1/BM
BP	GO:004677	protein aut	39/905	237/18866	1.35E-11	1.67E-10	8.80E-11	ADIPOQ/VI
BP	GO:001063	positive re	33/905	176/18866	1.39E-11	1.72E-10	9.06E-11	CALR/IFNG
BP	GO:007162	regulation	18/905	52/18866	1.40E-11	1.72E-10	9.06E-11	CXCL8/XCL
BP	GO:200123	positive re	18/905	52/18866	1.40E-11	1.72E-10	9.06E-11	PDIA3/TNF
BP	GO:006113	morphoger	34/905	187/18866	1.66E-11	2.05E-10	1.08E-10	TNF/PML/S
BP	GO:006040	calcium ior	31/905	158/18866	1.71E-11	2.09E-10	1.10E-10	CD4/CXCL1
BP	GO:190370	negative re	31/905	158/18866	1.71E-11	2.09E-10	1.10E-10	HLA-G/PGL
BP	GO:004851	rhythmic p	45/905	305/18866	1.72E-11	2.10E-10	1.10E-10	HSPA5/NFY
BP	GO:002260	regulation	61/905	499/18866	1.73E-11	2.11E-10	1.11E-10	CALR/ICAM
BP	GO:004352	negative re	30/905	149/18866	1.83E-11	2.23E-10	1.17E-10	HSP90AB1/
BP	GO:005192	positive re	27/905	122/18866	1.87E-11	2.27E-10	1.19E-10	CD4/HSPA2
BP	GO:005092	positive re	13/905	25/18866	1.99E-11	2.41E-10	1.27E-10	CXCL8/CXC
BP	GO:003273	positive re	18/905	53/18866	2.02E-11	2.44E-10	1.28E-10	IFNG/ORM
BP	GO:000680	superoxide	21/905	74/18866	2.14E-11	2.59E-10	1.36E-10	TGFB1/NO
BP	GO:000756	aging	46/905	319/18866	2.28E-11	2.74E-10	1.44E-10	B2M/CALR/

BP	GO:003265	regulation	19/905	60/18866	2.28E-11	2.74E-10	1.44E-10	FCER1G/XC
BP	GO:000717	epidermal	27/905	123/18866	2.29E-11	2.74E-10	1.44E-10	TGFB1/MM
BP	GO:000695	acute-phas	17/905	47/18866	2.29E-11	2.74E-10	1.44E-10	IL1B/LBP/C
BP	GO:004355	regulation	20/905	67/18866	2.30E-11	2.75E-10	1.45E-10	TGFB1/SO(
BP	GO:004339	regulation	36/905	211/18866	2.85E-11	3.40E-10	1.79E-10	B2M/HSP9
BP	GO:000696	cellular def	18/905	54/18866	2.89E-11	3.42E-10	1.80E-10	HLA-G/KIR
BP	GO:000962	response t	18/905	54/18866	2.89E-11	3.42E-10	1.80E-10	S100A9/S1
BP	GO:003262	interleukin	18/905	54/18866	2.89E-11	3.42E-10	1.80E-10	CD4/FCER1
BP	GO:003275	positive re	18/905	54/18866	2.89E-11	3.42E-10	1.80E-10	TLR2/IL1B/
BP	GO:000176	morphoger	35/905	201/18866	2.98E-11	3.51E-10	1.84E-10	TNF/PML/S
BP	GO:003459	cellular res	45/905	310/18866	3.00E-11	3.53E-10	1.86E-10	LCN2/MMI
BP	GO:000282	negative re	17/905	48/18866	3.39E-11	3.97E-10	2.09E-10	HLA-F/HLA
BP	GO:004671	viral entry	28/905	134/18866	3.41E-11	3.99E-10	2.10E-10	CD4/CTSB/
BP	GO:190495	positive re	50/905	370/18866	3.42E-11	3.99E-10	2.10E-10	HSP90AB1/
BP	GO:000270	negative re	16/905	42/18866	3.55E-11	4.13E-10	2.17E-10	HLA-A/HLA
BP	GO:004824	macrophag	16/905	42/18866	3.55E-11	4.13E-10	2.17E-10	CCL5/MAP
BP	GO:009730	response t	38/905	234/18866	3.70E-11	4.30E-10	2.26E-10	ICAM1/PSN
BP	GO:004884	negative re	13/905	26/18866	3.81E-11	4.40E-10	2.31E-10	WNT5A/SE
BP	GO:005092	regulation	13/905	26/18866	3.81E-11	4.40E-10	2.31E-10	CXCL8/CXC
BP	GO:001063	regulation	44/905	301/18866	3.95E-11	4.55E-10	2.39E-10	CALR/IFNG
BP	GO:003272	negative re	18/905	55/18866	4.11E-11	4.73E-10	2.49E-10	LBP/ADIPO
BP	GO:006048	mesenchyr	43/905	290/18866	4.12E-11	4.73E-10	2.49E-10	TGFB1/IL1I
BP	GO:200123	positive re	33/905	183/18866	4.16E-11	4.76E-10	2.50E-10	PDIA3/S10
BP	GO:004558	positive re	23/905	92/18866	4.18E-11	4.78E-10	2.51E-10	HLA-G/IFN
BP	GO:006039	regulation	19/905	62/18866	4.36E-11	4.97E-10	2.61E-10	TGFB1/BM
BP	GO:005076	positive re	59/905	485/18866	4.58E-11	5.21E-10	2.74E-10	HSPA5/IFN
BP	GO:200073	regulation	26/905	118/18866	4.90E-11	5.57E-10	2.93E-10	PSMB8/PSI
BP	GO:001401	positive re	21/905	77/18866	4.93E-11	5.59E-10	2.94E-10	LTA/TGFB1
BP	GO:004470	multi-multi	37/905	226/18866	5.26E-11	5.95E-10	3.13E-10	CALR/CTSB
BP	GO:003267	regulation	23/905	93/18866	5.30E-11	5.99E-10	3.15E-10	TMSB4X/TI
BP	GO:000236	T cell cytok	16/905	43/18866	5.40E-11	6.07E-10	3.19E-10	B2M/HLA-/
BP	GO:000282	negative re	16/905	43/18866	5.40E-11	6.07E-10	3.19E-10	HLA-F/HLA
BP	GO:003292	regulation	12/905	22/18866	5.73E-11	6.43E-10	3.38E-10	TGFB1/MA
BP	GO:000691	activation	22/905	86/18866	6.80E-11	7.61E-10	4.00E-10	S100A9/S1
BP	GO:190162	regulation	13/905	27/18866	7.02E-11	7.84E-10	4.12E-10	CXCL14/CX
BP	GO:000741	axon guida	42/905	284/18866	7.57E-11	8.44E-10	4.44E-10	CXCL12/VE
BP	GO:004876	mesenchyr	37/905	229/18866	7.80E-11	8.68E-10	4.56E-10	TGFB1/IL1I
BP	GO:003268	negative re	15/905	38/18866	8.02E-11	8.91E-10	4.68E-10	XCL1/PGLY
BP	GO:006076	positive re	18/905	57/18866	8.09E-11	8.94E-10	4.70E-10	MMP12/TL
BP	GO:190551	macrophag	18/905	57/18866	8.09E-11	8.94E-10	4.70E-10	CCL5/MAP
BP	GO:000243	inflammato	19/905	64/18866	8.11E-11	8.95E-10	4.70E-10	FCER1G/IC
BP	GO:190372	regulation	23/905	95/18866	8.46E-11	9.31E-10	4.90E-10	TGFB1/FAE
BP	GO:009748	neuron prc	42/905	285/18866	8.48E-11	9.32E-10	4.90E-10	CXCL12/VE
BP	GO:001000	glial cell dif	37/905	230/18866	8.88E-11	9.74E-10	5.12E-10	IFNG/S100
BP	GO:004873	gland deve	55/905	443/18866	9.76E-11	1.07E-09	5.61E-10	TGFB1/TNI
BP	GO:001056	regulation	33/905	189/18866	1.02E-10	1.11E-09	5.83E-10	PSMB8/PSI
BP	GO:003461	cellular res	31/905	170/18866	1.21E-10	1.31E-09	6.91E-10	LCN2/MMI
BP	GO:007083	divalent m	57/905	471/18866	1.21E-10	1.31E-09	6.91E-10	CD4/HSPA
BP	GO:190266	negative re	13/905	28/18866	1.25E-10	1.36E-09	7.15E-10	WNT5A/SE
BP	GO:001095	regulation	51/905	396/18866	1.28E-10	1.38E-09	7.27E-10	B2M/CD4/

BP	GO:00610[illegible]	regulation	29/905	151/18866	1.32E-10	1.42E-09	7.49E-10	FCER1G/PL
BP	GO:00075[illegible]	hemostasis	47/905	348/18866	1.37E-10	1.48E-09	7.76E-10	FCER1G/PF
BP	GO:19053[illegible]	regulation	32/905	181/18866	1.40E-10	1.50E-09	7.91E-10	PSMB8/PSI
BP	GO:00316[illegible]	response t	57/905	473/18866	1.42E-10	1.53E-09	8.05E-10	CD4/HSPA5
BP	GO:19035[illegible]	positive re	17/905	52/18866	1.46E-10	1.57E-09	8.24E-10	FCER1G/IFI
BP	GO:00512[illegible]	sequesterin	26/905	124/18866	1.58E-10	1.69E-09	8.89E-10	CALR/CXCL
BP	GO:00458[illegible]	positive re	29/905	153/18866	1.83E-10	1.96E-09	1.03E-09	IFNG/TGFB
BP	GO:00506[illegible]	regulation	15/905	40/18866	1.91E-10	2.03E-09	1.07E-09	MICB/MMI
BP	GO:007251	divalent in	57/905	478/18866	2.14E-10	2.28E-09	1.20E-09	CD4/HSPA2
BP	GO:00618[illegible]	antimicrob	20/905	75/18866	2.22E-10	2.36E-09	1.24E-09	CXCL14/CX
BP	GO:00468[illegible]	bone remo	22/905	91/18866	2.24E-10	2.37E-09	1.25E-09	TGFB1/NO
BP	GO:00458[illegible]	positive re	23/905	100/18866	2.56E-10	2.71E-09	1.43E-09	B2M/VEGF
BP	GO:00454[illegible]	regulation	19/905	68/18866	2.60E-10	2.74E-09	1.44E-09	HSP90AA1,
BP	GO:00075[illegible]	blood coag	46/905	343/18866	2.73E-10	2.88E-09	1.51E-09	FCER1G/PF
BP	GO:00380[illegible]	peptidyl-ty	15/905	41/18866	2.87E-10	3.02E-09	1.59E-09	VEGFA/MA
BP	GO:00301[illegible]	B cell diffe	27/905	137/18866	3.10E-10	3.26E-09	1.71E-09	CD40LG/PT
BP	GO:00326[illegible]	interleukin	23/905	101/18866	3.17E-10	3.32E-09	1.74E-09	TMSB4X/TI
BP	GO:00456[illegible]	positive re	23/905	101/18866	3.17E-10	3.32E-09	1.74E-09	CD4/IFNG/
BP	GO:00327[illegible]	positive re	11/905	20/18866	3.29E-10	3.43E-09	1.80E-09	DDX58/IRF
BP	GO:00466[illegible]	female sex	25/905	119/18866	3.40E-10	3.54E-09	1.86E-09	HSPA5/ICA
BP	GO:00610[illegible]	regulation	22/905	93/18866	3.52E-10	3.66E-09	1.92E-09	NOX4/CCL5
BP	GO:00108[illegible]	regulation	18/905	62/18866	3.84E-10	3.98E-09	2.09E-09	TNF/ADIPC
BP	GO:00308[illegible]	regulation	18/905	62/18866	3.84E-10	3.98E-09	2.09E-09	TFRC/CD40
BP	GO:00604[illegible]	cytosolic c	31/905	178/18866	4.00E-10	4.13E-09	2.17E-09	CD4/CXCL1
BP	GO:00027[illegible]	negative re	14/905	36/18866	4.37E-10	4.51E-09	2.37E-09	HLA-F/XCL1
BP	GO:00095[illegible]	detection c	14/905	36/18866	4.37E-10	4.51E-09	2.37E-09	HLA-A/HLA
BP	GO:001491	regulation	21/905	86/18866	4.70E-10	4.83E-09	2.54E-09	PLAU/NOX
BP	GO:19016[illegible]	response t	33/905	200/18866	4.71E-10	4.83E-09	2.54E-09	CALR/ICAN
BP	GO:00433[illegible]	CD4-positiv	20/905	78/18866	4.77E-10	4.88E-09	2.57E-09	CTSL/IFNG,
BP	GO:005081	coagulatio	46/905	349/18866	4.87E-10	4.98E-09	2.62E-09	FCER1G/PF
BP	GO:000301	vascular pr	32/905	190/18866	5.05E-10	5.15E-09	2.71E-09	ICAM1/TGI
BP	GO:00026[illegible]	regulation	16/905	49/18866	5.20E-10	5.29E-09	2.78E-09	FCER1G/IL1
BP	GO:00468[illegible]	hormone s	43/905	314/18866	5.43E-10	5.52E-09	2.90E-09	IFNG/IL1B/
BP	GO:00302[illegible]	regulation	34/905	212/18866	5.60E-10	5.68E-09	2.98E-09	TGFB1/LTF
BP	GO:00022[illegible]	T cell diffe	19/905	71/18866	5.86E-10	5.93E-09	3.12E-09	FCER1G/IFI
BP	GO:00323[illegible]	response t	27/905	141/18866	6.11E-10	6.17E-09	3.24E-09	CALR/CD4/
BP	GO:00075[illegible]	sex differe	40/905	280/18866	6.16E-10	6.22E-09	3.27E-09	HSPA5/ICA
BP	GO:01401[illegible]	positive re	11/905	21/18866	6.61E-10	6.66E-09	3.50E-09	CXCL14/CX
BP	GO:00105[illegible]	positive re	26/905	132/18866	6.69E-10	6.72E-09	3.53E-09	CALR/TMSI
BP	GO:00903[illegible]	regulation	14/905	37/18866	6.73E-10	6.75E-09	3.55E-09	TGFB1/TNI
BP	GO:00486[illegible]	axon exten	25/905	123/18866	7.12E-10	7.13E-09	3.75E-09	HSP90AA1,
BP	GO:19055[illegible]	positive re	12/905	26/18866	7.16E-10	7.16E-09	3.76E-09	CCL5/MAP
BP	GO:00327[illegible]	positive re	16/905	50/18866	7.30E-10	7.29E-09	3.83E-09	FCER1G/TL
BP	GO:005071	positive re	30/905	172/18866	7.37E-10	7.34E-09	3.86E-09	IFNG/TGFB
BP	GO:00316[illegible]	heat gener	10/905	17/18866	8.79E-10	8.74E-09	4.59E-09	IL1B/TNF/T
BP	GO:00466[illegible]	alpha-beta	23/905	106/18866	8.81E-10	8.74E-09	4.59E-09	CTSL/IFNG,
BP	GO:19026[illegible]	regulation	15/905	44/18866	9.10E-10	8.99E-09	4.73E-09	CXCL12/VE
BP	GO:19055[illegible]	regulation	15/905	44/18866	9.10E-10	8.99E-09	4.73E-09	CCL5/MAP
BP	GO:00319[illegible]	response t	29/905	164/18866	1.01E-09	1.00E-08	5.26E-09	ICAM1/S10
BP	GO:00107[illegible]	macrophag	14/905	38/18866	1.02E-09	1.00E-08	5.26E-09	TGFB1/CET

BP	GO:009007	foam cell d	14/905	38/18866	1.02E-09	1.00E-08	5.26E-09	TGFB1/CET
BP	GO:190342	regulation	23/905	107/18866	1.07E-09	1.05E-08	5.53E-09	HSP90AA1,
BP	GO:000166	fever gene	8/905	10/18866	1.12E-09	1.10E-08	5.79E-09	IL1B/TNF/T
BP	GO:001076	regulation	42/905	310/18866	1.20E-09	1.17E-08	6.17E-09	CALR/CXCL
BP	GO:005083	defense re:	22/905	99/18866	1.26E-09	1.23E-08	6.48E-09	HLA-A/MR:
BP	GO:000271	regulation	15/905	45/18866	1.30E-09	1.27E-08	6.68E-09	HLA-A/HLA
BP	GO:000991	hormone t	43/905	323/18866	1.32E-09	1.28E-08	6.75E-09	IFNG/IL1B/
BP	GO:000189	placenta de	28/905	156/18866	1.38E-09	1.34E-08	7.06E-09	CTSB/HSP9
BP	GO:003410	regulation	21/905	91/18866	1.44E-09	1.39E-08	7.33E-09	TGFB1/TFR
BP	GO:003302	myeloid ce	13/905	33/18866	1.53E-09	1.48E-08	7.77E-09	CTSL/FCER
BP	GO:004355	positive re	13/905	33/18866	1.53E-09	1.48E-08	7.77E-09	TGFB1/SRC
BP	GO:005120	release of :	24/905	118/18866	1.53E-09	1.48E-08	7.77E-09	CXCL10/CX
BP	GO:004512	cellular ext	18/905	67/18866	1.55E-09	1.49E-08	7.83E-09	ICAM1/CX(
BP	GO:004592	negative re	37/905	254/18866	1.57E-09	1.51E-08	7.93E-09	TGFB1/RBF
BP	GO:004361	regulation	23/905	109/18866	1.57E-09	1.51E-08	7.94E-09	HSPA5/PSN
BP	GO:007149	cellular res	43/905	326/18866	1.76E-09	1.68E-08	8.84E-09	HSPA5/ICA
BP	GO:009719	intrinsic ap	40/905	290/18866	1.76E-09	1.68E-08	8.84E-09	CXCL12/S1
BP	GO:006109	positive re	17/905	60/18866	1.79E-09	1.71E-08	8.99E-09	NOX4/CCL!
BP	GO:005128	negative re	24/905	119/18866	1.84E-09	1.75E-08	9.18E-09	CXCL10/CX
BP	GO:005079	activated T	15/905	46/18866	1.85E-09	1.76E-08	9.24E-09	AGER/EPO,
BP	GO:001057	positive re	12/905	28/18866	2.06E-09	1.95E-08	1.03E-08	TGFB1/IL1I
BP	GO:001081	T cell chem	12/905	28/18866	2.06E-09	1.95E-08	1.03E-08	CXCL10/CX
BP	GO:001490	smooth mu	21/905	93/18866	2.20E-09	2.07E-08	1.09E-08	PLAU/NOX
BP	GO:003208	negative re	21/905	93/18866	2.20E-09	2.07E-08	1.09E-08	PSMD10/T
BP	GO:005105	negative re	55/905	483/18866	2.44E-09	2.30E-08	1.21E-08	HLA-F/ICAI
BP	GO:011011	positive re	20/905	85/18866	2.46E-09	2.31E-08	1.21E-08	VEGFA/TNI
BP	GO:009755	calcium ior	26/905	140/18866	2.51E-09	2.36E-08	1.24E-08	CXCL10/CX
BP	GO:000165	urogenital	43/905	330/18866	2.57E-09	2.41E-08	1.26E-08	MMP9/RBI
BP	GO:005128	regulation	24/905	121/18866	2.61E-09	2.44E-08	1.28E-08	CXCL10/CX
BP	GO:006056	developme	35/905	236/18866	2.68E-09	2.51E-08	1.32E-08	HSP90AA1,
BP	GO:000758	response to	29/905	171/18866	2.77E-09	2.58E-08	1.36E-08	CD4/LTA/C
BP	GO:004866	positive re	22/905	103/18866	2.80E-09	2.60E-08	1.37E-08	MMP9/TNI
BP	GO:004226	regulation	14/905	41/18866	3.24E-09	3.01E-08	1.58E-08	HLA-A/HLA
BP	GO:005083	defense re:	14/905	41/18866	3.24E-09	3.01E-08	1.58E-08	S100A9/S1
BP	GO:007162	positive re	12/905	29/18866	3.37E-09	3.11E-08	1.63E-08	CXCL8/XCL
BP	GO:001059	regulation	35/905	238/18866	3.37E-09	3.11E-08	1.63E-08	CALR/CXCL
BP	GO:003253	regulation	47/905	383/18866	3.37E-09	3.11E-08	1.63E-08	HSP90AB1,
BP	GO:004562	negative re	16/905	55/18866	3.52E-09	3.24E-08	1.70E-08	PGLYRP1/P
BP	GO:009880	regulation	13/905	35/18866	3.61E-09	3.32E-08	1.74E-08	ADIPOQ/F2
BP	GO:001086	positive re	15/905	48/18866	3.61E-09	3.32E-08	1.74E-08	TGFB1/BM
BP	GO:006108	positive re	10/905	19/18866	3.82E-09	3.50E-08	1.84E-08	FCER1G/HL
BP	GO:003574	natural kill	8/905	11/18866	3.95E-09	3.61E-08	1.90E-08	CXCL14/XC
BP	GO:000240	dendritic c	11/905	24/18866	4.09E-09	3.72E-08	1.96E-08	CALR/CCL5
BP	GO:003134	negative re	11/905	24/18866	4.09E-09	3.72E-08	1.96E-08	HLA-A/HLA
BP	GO:004663	positive re	17/905	63/18866	4.10E-09	3.72E-08	1.96E-08	HLA-A/IFN(
BP	GO:200051	regulation	17/905	63/18866	4.10E-09	3.72E-08	1.96E-08	IFNG/XCL1,
BP	GO:005067	negative re	19/905	79/18866	4.15E-09	3.77E-08	1.98E-08	HLA-G/XCL
BP	GO:004362	regulation	23/905	115/18866	4.72E-09	4.27E-08	2.25E-08	HSPA5/PSN
BP	GO:004864	negative re	23/905	115/18866	4.72E-09	4.27E-08	2.25E-08	RBP4/WNT
BP	GO:001481	muscle cell	22/905	106/18866	4.94E-09	4.46E-08	2.34E-08	PLAU/NOX

BP	GO:003266	regulation	15/905	49/18866	4.98E-09	4.48E-08	2.36E-08	CD4/XCL1/
BP	GO:005077	regulation	30/905	186/18866	5.02E-09	4.52E-08	2.38E-08	CXCL12/VE
BP	GO:001403	neural cres	19/905	80/18866	5.20E-09	4.67E-08	2.45E-08	MAPK3/ED
BP	GO:003294	negative re	19/905	80/18866	5.20E-09	4.67E-08	2.45E-08	HLA-G/XCL
BP	GO:001075	regulation	12/905	30/18866	5.36E-09	4.78E-08	2.51E-08	CCL5/MAP
BP	GO:003272	positive re	12/905	30/18866	5.36E-09	4.78E-08	2.51E-08	TLR2/DDX5
BP	GO:003566	TRIF-deper	12/905	30/18866	5.36E-09	4.78E-08	2.51E-08	IRF3/TLR3/
BP	GO:006108	myeloid le	12/905	30/18866	5.36E-09	4.78E-08	2.51E-08	FCER1G/HL
BP	GO:000275	MyD88-de	13/905	36/18866	5.40E-09	4.80E-08	2.52E-08	TLR2/IRF1/
BP	GO:004663	alpha-beta	13/905	36/18866	5.40E-09	4.80E-08	2.52E-08	HLA-A/XCL
BP	GO:005066	cytokine se	18/905	72/18866	5.46E-09	4.84E-08	2.55E-08	IFNG/TLR2/
BP	GO:000862	extrinsic ap	20/905	89/18866	5.78E-09	5.12E-08	2.69E-08	ICAM1/TNI
BP	GO:004688	regulation	37/905	267/18866	6.30E-09	5.57E-08	2.93E-08	IFNG/IL1B/
BP	GO:000196	positive re	15/905	50/18866	6.80E-09	5.99E-08	3.15E-08	MMP12/IR
BP	GO:007022	lymphocyt	18/905	73/18866	6.93E-09	6.09E-08	3.20E-08	IDO1/CCL5
BP	GO:190203	regulation	18/905	73/18866	6.93E-09	6.09E-08	3.20E-08	PSMB8/PSI
BP	GO:000209	positive re	11/905	25/18866	7.00E-09	6.12E-08	3.22E-08	VEGFA/ARI
BP	GO:004669	decidualiza	11/905	25/18866	7.00E-09	6.12E-08	3.22E-08	CTSB/MAP
BP	GO:007167	positive re	11/905	25/18866	7.00E-09	6.12E-08	3.22E-08	CXCL10/CX
BP	GO:004232	negative re	54/905	484/18866	7.00E-09	6.12E-08	3.22E-08	IFNG/PSMI
BP	GO:007054	response t	20/905	90/18866	7.11E-09	6.20E-08	3.26E-08	TLR2/FABP
BP	GO:000722	integrin-m	22/905	108/18866	7.13E-09	6.21E-08	3.26E-08	FCER1G/IS
BP	GO:000191	negative re	10/905	20/18866	7.32E-09	6.35E-08	3.34E-08	HLA-A/HLA
BP	GO:000282	positive re	10/905	20/18866	7.32E-09	6.35E-08	3.34E-08	XCL1/IL1B/
BP	GO:009021	positive re	13/905	37/18866	7.97E-09	6.91E-08	3.63E-08	TGFB1/SRC
BP	GO:003505	cardiocyte	27/905	158/18866	8.21E-09	7.10E-08	3.73E-08	CALR/TGFE
BP	GO:003030	negative re	30/905	190/18866	8.36E-09	7.21E-08	3.79E-08	TGFB1/PM
BP	GO:005104	negative re	30/905	190/18866	8.36E-09	7.21E-08	3.79E-08	HLA-F/IL1B
BP	GO:000267	positive re	12/905	31/18866	8.37E-09	7.21E-08	3.79E-08	FCER1G/IL1
BP	GO:190289	regulation	15/905	51/18866	9.20E-09	7.91E-08	4.16E-08	TGFB1/TNI
BP	GO:009702	dendritic c	14/905	44/18866	9.25E-09	7.92E-08	4.16E-08	HLA-B/HLA
BP	GO:200040	regulation	14/905	44/18866	9.25E-09	7.92E-08	4.16E-08	CXCL10/CX
BP	GO:004870	astrocyte d	19/905	83/18866	1.00E-08	8.57E-08	4.50E-08	IFNG/S100
BP	GO:004325	regulation	51/905	449/18866	1.02E-08	8.72E-08	4.58E-08	HSPA5/HSI
BP	GO:004858	developme	34/905	237/18866	1.07E-08	9.13E-08	4.80E-08	HSP90AA1,
BP	GO:004595	negative re	9/905	16/18866	1.09E-08	9.29E-08	4.88E-08	HLA-A/HLA
BP	GO:000717	transmemb	44/905	359/18866	1.11E-08	9.45E-08	4.97E-08	HSPA5/HSI
BP	GO:003158	cell-substr	44/905	359/18866	1.11E-08	9.45E-08	4.97E-08	CALR/MMI
BP	GO:003273	positive re	13/905	38/18866	1.16E-08	9.80E-08	5.15E-08	FCER1G/X
BP	GO:009002	positive re	11/905	26/18866	1.16E-08	9.80E-08	5.15E-08	CXCL8/XCL
BP	GO:009002	regulation	11/905	26/18866	1.16E-08	9.80E-08	5.15E-08	CXCL10/CX
BP	GO:006138	regulation	22/905	111/18866	1.21E-08	1.02E-07	5.38E-08	CXCL12/VE
BP	GO:001403	mesenchyr	19/905	84/18866	1.24E-08	1.04E-07	5.47E-08	MAPK3/ED
BP	GO:004886	stem cell d	19/905	84/18866	1.24E-08	1.04E-07	5.47E-08	MAPK3/ED
BP	GO:000272	regulation	12/905	32/18866	1.28E-08	1.07E-07	5.64E-08	B2M/HLA-
BP	GO:001074	regulation	12/905	32/18866	1.28E-08	1.07E-07	5.64E-08	CETP/ADIP
BP	GO:003272	positive re	14/905	45/18866	1.28E-08	1.07E-07	5.64E-08	TLR2/IL1B/
BP	GO:004542	positive re	14/905	45/18866	1.28E-08	1.07E-07	5.64E-08	HSP90AA1,
BP	GO:000253	production	20/905	93/18866	1.29E-08	1.08E-07	5.67E-08	FCER1G/LB
BP	GO:003545	response t	10/905	21/18866	1.34E-08	1.11E-07	5.85E-08	EIF2AK2/B

BP	GO:00483(	immunoglo	10/905	21/18866	1.34E-08	1.11E-07	5.85E-08	RBP4/TNF/
BP	GO:00026:	regulation	17/905	68/18866	1.45E-08	1.20E-07	6.33E-08	XCL1/TGFB
BP	GO:006161	pri-miRNA	15/905	53/18866	1.65E-08	1.37E-07	7.19E-08	TGFB1/TNI
BP	GO:00420(	type 2 imm	13/905	39/18866	1.66E-08	1.38E-07	7.23E-08	XCL1/IFNL1
BP	GO:006141	regulation	18/905	77/18866	1.72E-08	1.42E-07	7.47E-08	PSMB8/PSI
BP	GO:19044(	positive re	14/905	46/18866	1.76E-08	1.46E-07	7.65E-08	HSP90AA1,
BP	GO:00486(	negative re	17/905	69/18866	1.84E-08	1.52E-07	7.97E-08	IFNG/APOI
BP	GO:00309(	forebrain d	46/905	391/18866	1.86E-08	1.53E-07	8.04E-08	B2M/CXCL
BP	GO:19015:	regulation	19/905	86/18866	1.87E-08	1.53E-07	8.06E-08	PSMB8/PSI
BP	GO:00019:	negative re	50/905	444/18866	1.89E-08	1.55E-07	8.14E-08	IFNG/PSMI
BP	GO:00486:	regulation	43/905	353/18866	1.96E-08	1.61E-07	8.44E-08	CXCL12/RB
BP	GO:00022(	myeloid ce	26/905	154/18866	2.00E-08	1.64E-07	8.61E-08	B2M/FCER
BP	GO:006201	positive re	26/905	154/18866	2.00E-08	1.64E-07	8.61E-08	IFNG/TMSI
BP	GO:00603(	SMAD prot	18/905	78/18866	2.13E-08	1.74E-07	9.15E-08	TGFB1/FOS
BP	GO:000271	negative re	9/905	17/18866	2.22E-08	1.81E-07	9.50E-08	HLA-A/HLA
BP	GO:00069:	inflammat	10/905	22/18866	2.35E-08	1.91E-07	1.00E-07	CTSL/IRF3/
BP	GO:19028(	positive re	13/905	40/18866	2.35E-08	1.91E-07	1.00E-07	TGFB1/TNI
BP	GO:00313:	positive re	35/905	257/18866	2.53E-08	2.05E-07	1.08E-07	HSP90AA1,
BP	GO:00712(	cellular res	18/905	79/18866	2.64E-08	2.14E-07	1.12E-07	IL1B/TLR3/
BP	GO:003051	regulation	20/905	97/18866	2.76E-08	2.23E-07	1.17E-07	CXCL12/VE
BP	GO:190121	positive re	20/905	97/18866	2.76E-08	2.23E-07	1.17E-07	IFNG/TNF/
BP	GO:00097!	hormone-r	30/905	200/18866	2.80E-08	2.25E-07	1.18E-07	CALR/SRC/
BP	GO:00425<	response t	25/905	146/18866	2.80E-08	2.25E-07	1.18E-07	LCN2/STAT
BP	GO:00316!	regulation	8/905	13/18866	2.82E-08	2.27E-07	1.19E-07	IL1B/TNF/T
BP	GO:00027!	MyD88-ind	12/905	34/18866	2.85E-08	2.28E-07	1.20E-07	IRF3/TLR3/
BP	GO:00702:	regulation	12/905	34/18866	2.85E-08	2.28E-07	1.20E-07	IDO1/CCL5
BP	GO:190401	epithelial c	22/905	117/18866	3.32E-08	2.66E-07	1.40E-07	HLA-G/ICA
BP	GO:00140:	neural cres	19/905	89/18866	3.38E-08	2.70E-07	1.42E-07	MAPK3/ED
BP	GO:00421{	cellular ket	35/905	260/18866	3.41E-08	2.72E-07	1.43E-07	PSMB8/PSI
BP	GO:00017!	neural cres	15/905	56/18866	3.75E-08	2.97E-07	1.56E-07	EDN3/SEM
BP	GO:000271	regulation	15/905	56/18866	3.75E-08	2.97E-07	1.56E-07	FCER1G/LT
BP	GO:00028{	regulation	15/905	56/18866	3.75E-08	2.97E-07	1.56E-07	FCER1G/LT
BP	GO:00702:	regulation	15/905	56/18866	3.75E-08	2.97E-07	1.56E-07	IDO1/CCL5
BP	GO:00421:	negative re	16/905	64/18866	3.85E-08	3.05E-07	1.60E-07	HLA-G/XCL
BP	GO:001401	regulation	23/905	128/18866	3.96E-08	3.13E-07	1.64E-07	LTA/TGFB1
BP	GO:00456<	regulation	10/905	23/18866	3.97E-08	3.13E-07	1.65E-07	TGFB1/ADI
BP	GO:00726;	eosinophil	10/905	23/18866	3.97E-08	3.13E-07	1.65E-07	CCL13/CCL
BP	GO:00508:	defense re:	18/905	81/18866	4.00E-08	3.15E-07	1.66E-07	CD4/MR1/
BP	GO:00420!	regulation	19/905	90/18866	4.09E-08	3.22E-07	1.69E-07	MMP9/FAS
BP	GO:00083(	regulation	28/905	181/18866	4.11E-08	3.23E-07	1.70E-07	HSP90AB1,
BP	GO:00603:	regulation	12/905	35/18866	4.14E-08	3.24E-07	1.70E-07	HSP90AB1,
BP	GO:00706;	response t	12/905	35/18866	4.14E-08	3.24E-07	1.70E-07	HSPA5/HSI
BP	GO:00614<	connective	35/905	262/18866	4.15E-08	3.24E-07	1.70E-07	TGFB1/MA
BP	GO:00140;	response t	25/905	149/18866	4.25E-08	3.31E-07	1.74E-07	HSPA5/IL1I
BP	GO:00510:	regulation	9/905	18/18866	4.25E-08	3.31E-07	1.74E-07	RBP4/TNF/
BP	GO:00702:	T cell apop	14/905	49/18866	4.34E-08	3.38E-07	1.77E-07	IDO1/CCL5
BP	GO:00017;	myeloid de	11/905	29/18866	4.55E-08	3.52E-07	1.85E-07	RELB/IL10/
BP	GO:00330:	regulation	11/905	29/18866	4.55E-08	3.52E-07	1.85E-07	FCER1G/AI
BP	GO:00363:	dendritic c	11/905	29/18866	4.55E-08	3.52E-07	1.85E-07	CALR/CCL5
BP	GO:00430:	positive re	11/905	29/18866	4.55E-08	3.52E-07	1.85E-07	LBP/WNT5

BP	GO:00460(	regulation	13/905	42/18866	4.56E-08	3.53E-07	1.85E-07	AGER/EPO/
BP	GO:003327	response t	19/905	91/18866	4.94E-08	3.81E-07	2.00E-07	CD4/CXCL1
BP	GO:001081	regulation	31/905	217/18866	5.23E-08	4.03E-07	2.12E-07	CALR/MMI
BP	GO:003017	positive re	28/905	183/18866	5.24E-08	4.03E-07	2.12E-07	PSMB8/PSI
BP	GO:199013	neuron prc	27/905	172/18866	5.24E-08	4.03E-07	2.12E-07	HSP90AA1/
BP	GO:004513	developme	32/905	229/18866	5.43E-08	4.17E-07	2.19E-07	HSPA5/ICA
BP	GO:007067	response t	14/905	50/18866	5.76E-08	4.41E-07	2.32E-07	IFNG/PSMI
BP	GO:190372	positive re	14/905	50/18866	5.76E-08	4.41E-07	2.32E-07	TGFB1/FAE
BP	GO:004574	positive re	12/905	36/18866	5.95E-08	4.54E-07	2.39E-07	MMP9/FAS
BP	GO:19052(	regulation	16/905	66/18866	6.16E-08	4.70E-07	2.47E-07	TGFB1/SEN
BP	GO:003051	negative re	13/905	43/18866	6.26E-08	4.77E-07	2.50E-07	WNT5A/SE
BP	GO:000247	antigen prc	8/905	14/18866	6.31E-08	4.80E-07	2.52E-07	AZGP1/B2I
BP	GO:000271	negative re	10/905	24/18866	6.52E-08	4.95E-07	2.60E-07	HLA-F/XCL
BP	GO:004873	cardiac mu	32/905	231/18866	6.68E-08	5.07E-07	2.66E-07	CALR/TGFE
BP	GO:001072	negative re	41/905	343/18866	7.11E-08	5.38E-07	2.83E-07	B2M/CALR/
BP	GO:200011	negative re	19/905	93/18866	7.15E-08	5.41E-07	2.84E-07	MMP9/LTF
BP	GO:004327	apoptotic c	14/905	51/18866	7.60E-08	5.74E-07	3.02E-07	ITGAV/CCL
BP	GO:001075	positive re	9/905	19/18866	7.73E-08	5.80E-07	3.05E-07	CCL5/MAP
BP	GO:004824	eosinophil	9/905	19/18866	7.73E-08	5.80E-07	3.05E-07	CCL13/CCL
BP	GO:007023	negative re	9/905	19/18866	7.73E-08	5.80E-07	3.05E-07	IDO1/CCL5
BP	GO:009854	detection c	9/905	19/18866	7.73E-08	5.80E-07	3.05E-07	HLA-A/HLA
BP	GO:015014	cell junctio	9/905	19/18866	7.73E-08	5.80E-07	3.05E-07	CX3CR1/DI
BP	GO:004303	regulation	15/905	59/18866	8.03E-08	6.02E-07	3.16E-07	LBP/WNT5
BP	GO:000821	regulation	28/905	187/18866	8.39E-08	6.28E-07	3.30E-07	ERAP1/CTS
BP	GO:004568	positive re	13/905	44/18866	8.49E-08	6.34E-07	3.33E-07	TGFB1/TLR
BP	GO:00301(	regulation	30/905	210/18866	8.57E-08	6.39E-07	3.36E-07	B2M/VEGF
BP	GO:190165	cellular res	45/905	398/18866	8.67E-08	6.46E-07	3.39E-07	ICAM1/IL1
BP	GO:001657	protein de	36/905	283/18866	9.41E-08	7.00E-07	3.68E-07	PSMB8/PSI
BP	GO:004875	branching	25/905	155/18866	9.46E-08	7.03E-07	3.69E-07	TNF/PML/
BP	GO:00084(	gonad dev	31/905	223/18866	9.87E-08	7.32E-07	3.85E-07	HSPA5/ICA
BP	GO:004826	positive re	14/905	52/18866	9.94E-08	7.36E-07	3.87E-07	B2M/VEGF
BP	GO:000282	regulation	11/905	31/18866	1.02E-07	7.52E-07	3.95E-07	XCL1/IFNL1
BP	GO:190262	positive re	11/905	31/18866	1.02E-07	7.52E-07	3.95E-07	CXCL8/XCL
BP	GO:20004(	positive re	11/905	31/18866	1.02E-07	7.52E-07	3.95E-07	CXCL10/CX
BP	GO:190342	positive re	15/905	60/18866	1.02E-07	7.54E-07	3.97E-07	HSP90AA1/
BP	GO:003109	regenerati	29/905	201/18866	1.13E-07	8.30E-07	4.36E-07	CXCL12/AP
BP	GO:00140(	astrocyte d	13/905	45/18866	1.14E-07	8.39E-07	4.41E-07	IFNG/S100
BP	GO:190118	positive re	12/905	38/18866	1.18E-07	8.63E-07	4.54E-07	MMP9/FAS
BP	GO:003033	negative re	41/905	350/18866	1.24E-07	9.10E-07	4.78E-07	CALR/CXCL
BP	GO:005178	positive re	18/905	87/18866	1.29E-07	9.39E-07	4.94E-07	TGFB1/IL1
BP	GO:006021	hematopoi	18/905	87/18866	1.29E-07	9.39E-07	4.94E-07	PSMB8/PSI
BP	GO:004333	response t	14/905	53/18866	1.29E-07	9.41E-07	4.95E-07	DDX58/IRF
BP	GO:004559	negative re	14/905	53/18866	1.29E-07	9.41E-07	4.95E-07	TGFB1/TNI
BP	GO:002303	CD40 signa	8/905	15/18866	1.30E-07	9.43E-07	4.95E-07	CD40/TNF
BP	GO:005138	response t	24/905	147/18866	1.34E-07	9.76E-07	5.13E-07	ICAM1/S10
BP	GO:190382	positive re	40/905	338/18866	1.34E-07	9.76E-07	5.13E-07	FCER1G/HS
BP	GO:000241	immune re	9/905	20/18866	1.35E-07	9.76E-07	5.13E-07	HLA-A/MR
BP	GO:007064	protein mc	37/905	300/18866	1.39E-07	1.01E-06	5.29E-07	PSMB8/PSI
BP	GO:200014	negative re	42/905	365/18866	1.44E-07	1.04E-06	5.46E-07	CALR/CXCL
BP	GO:012016	positive re	19/905	97/18866	1.45E-07	1.04E-06	5.49E-07	LCN2/FABI

BP	GO:190118	regulation	19/905	97/18866	1.45E-07	1.04E-06	5.49E-07	MMP9/FAS
BP	GO:003267	regulation	11/905	32/18866	1.49E-07	1.07E-06	5.60E-07	FCER1G/CD
BP	GO:003812	ERBB2 sign	11/905	32/18866	1.49E-07	1.07E-06	5.60E-07	HSP90AA1,
BP	GO:004558	regulation	11/905	32/18866	1.49E-07	1.07E-06	5.60E-07	HLA-G/IFN(
BP	GO:007135	cellular res	11/905	32/18866	1.49E-07	1.07E-06	5.60E-07	HSPA5/HSP
BP	GO:004333	response t	13/905	46/18866	1.52E-07	1.09E-06	5.73E-07	DDX58/IRF
BP	GO:190547	positive re	22/905	127/18866	1.53E-07	1.09E-06	5.74E-07	IFNG/TNF/
BP	GO:005134	regulation	21/905	117/18866	1.56E-07	1.12E-06	5.86E-07	HSP90AA1,
BP	GO:003165	positive re	7/905	11/18866	1.59E-07	1.13E-06	5.95E-07	IL1B/TNF/T
BP	GO:007075	interleukin	7/905	11/18866	1.59E-07	1.13E-06	5.95E-07	STAT1/EBI3
BP	GO:009888	synapse pr	7/905	11/18866	1.59E-07	1.13E-06	5.95E-07	CX3CR1/DK
BP	GO:003274	positive re	10/905	26/18866	1.62E-07	1.15E-06	6.03E-07	CD4/IL1B/I
BP	GO:004210	positive re	10/905	26/18866	1.62E-07	1.15E-06	6.03E-07	AGER/EPO,
BP	GO:000229	CD4-positiv	15/905	62/18866	1.64E-07	1.16E-06	6.10E-07	IFNG/RELB,
BP	GO:003031	osteoclast	19/905	98/18866	1.72E-07	1.22E-06	6.39E-07	FCER1G/IFI
BP	GO:190595	regulation	26/905	171/18866	1.77E-07	1.25E-06	6.58E-07	IL1B/FABP3
BP	GO:004343	negative re	27/905	183/18866	1.94E-07	1.37E-06	7.18E-07	PSMD10/X
BP	GO:000224	hematopoi	26/905	172/18866	1.99E-07	1.41E-06	7.39E-07	PSMB8/PSI
BP	GO:002260	ovulation c	13/905	47/18866	2.01E-07	1.42E-06	7.45E-07	HSPA5/SRC
BP	GO:000228	alpha-beta	15/905	63/18866	2.05E-07	1.44E-06	7.58E-07	IFNG/RELB,
BP	GO:000229	alpha-beta	15/905	63/18866	2.05E-07	1.44E-06	7.58E-07	IFNG/RELB,
BP	GO:007200	renal syste	36/905	292/18866	2.07E-07	1.45E-06	7.63E-07	MMP9/RBI
BP	GO:004664	regulation	11/905	33/18866	2.13E-07	1.49E-06	7.84E-07	HLA-A/XCL
BP	GO:009002	regulation	11/905	33/18866	2.13E-07	1.49E-06	7.84E-07	CXCL8/XCL
BP	GO:006056	epithelial t	39/905	331/18866	2.16E-07	1.51E-06	7.94E-07	CXCL10/TG
BP	GO:014035	lipid export	12/905	40/18866	2.22E-07	1.55E-06	8.15E-07	IL1B/TNFSF
BP	GO:004814	astrocyte a	10/905	27/18866	2.46E-07	1.71E-06	9.00E-07	IFNG/IL1B/
BP	GO:000252	leukocyte r	8/905	16/18866	2.48E-07	1.73E-06	9.08E-07	S100A9/S1
BP	GO:005109	positive re	26/905	174/18866	2.52E-07	1.75E-06	9.20E-07	B2M/HSP9
BP	GO:004663	regulation	15/905	64/18866	2.57E-07	1.78E-06	9.35E-07	IFNG/SOCS
BP	GO:006013	maternal p	15/905	64/18866	2.57E-07	1.78E-06	9.35E-07	CTSB/MAP
BP	GO:001491	positive re	13/905	48/18866	2.64E-07	1.83E-06	9.60E-07	NOX4/CCL5
BP	GO:004337	regulation	13/905	48/18866	2.64E-07	1.83E-06	9.60E-07	IFNG/SOCS
BP	GO:006068	regulation	14/905	56/18866	2.73E-07	1.88E-06	9.90E-07	TNF/VEGFA
BP	GO:001052	regulation	19/905	101/18866	2.82E-07	1.95E-06	1.02E-06	CD4/CXCL1
BP	GO:005068	negative re	26/905	175/18866	2.83E-07	1.95E-06	1.02E-06	B2M/TGFB
BP	GO:003087	mammary	23/905	142/18866	2.83E-07	1.95E-06	1.02E-06	PML/VEGFA
BP	GO:005134	negative re	36/905	296/18866	2.90E-07	1.99E-06	1.05E-06	IFNG/IL1B/
BP	GO:190204	negative re	12/905	41/18866	3.00E-07	2.06E-06	1.08E-06	ICAM1/TNI
BP	GO:003281	regulation	11/905	34/18866	3.02E-07	2.07E-06	1.09E-06	HLA-F/MIC
BP	GO:003529	regulation	23/905	143/18866	3.22E-07	2.20E-06	1.16E-06	ICAM1/F2R
BP	GO:004559	regulation	23/905	143/18866	3.22E-07	2.20E-06	1.16E-06	TGFB1/TNF
BP	GO:009774	regulation	23/905	143/18866	3.22E-07	2.20E-06	1.16E-06	ICAM1/F2R
BP	GO:000268	negative re	13/905	49/18866	3.44E-07	2.34E-06	1.23E-06	CXCL12/AP
BP	GO:004505	T cell selec	13/905	49/18866	3.44E-07	2.34E-06	1.23E-06	CD4/CTSL/
BP	GO:007134	cellular res	13/905	49/18866	3.44E-07	2.34E-06	1.23E-06	IFNG/PSMI
BP	GO:005105	positive re	28/905	200/18866	3.51E-07	2.39E-06	1.25E-06	HSP90AA1,
BP	GO:000255	mast cell cl	7/905	12/18866	3.66E-07	2.47E-06	1.30E-06	VEGFA/CCL
BP	GO:003249	detection c	7/905	12/18866	3.66E-07	2.47E-06	1.30E-06	TLR2/LBP/I
BP	GO:003811	interleukin	7/905	12/18866	3.66E-07	2.47E-06	1.30E-06	PTK2B/IL2R

BP	GO:190353	negative re	25/905	166/18866	3.66E-07	2.47E-06	1.30E-06	HLA-F/IL1B
BP	GO:003515	regulation	23/905	144/18866	3.67E-07	2.47E-06	1.30E-06	ICAM1/F2F
BP	GO:010610	cold-induce	23/905	144/18866	3.67E-07	2.47E-06	1.30E-06	LCN2/FABF
BP	GO:012016	regulation	23/905	144/18866	3.67E-07	2.47E-06	1.30E-06	LCN2/FABF
BP	GO:000189	tissue hom	33/905	261/18866	3.69E-07	2.49E-06	1.31E-06	AZGP1/B2M
BP	GO:199084	adaptive th	24/905	155/18866	3.71E-07	2.49E-06	1.31E-06	LCN2/FABF
BP	GO:003297	regulation	44/905	405/18866	3.73E-07	2.50E-06	1.32E-06	ICAM1/CXC
BP	GO:000636	transcriptio	27/905	189/18866	3.76E-07	2.52E-06	1.32E-06	PSMC2/PSI
BP	GO:004668	response to	22/905	134/18866	4.02E-07	2.69E-06	1.41E-06	HSPA5/IL1I
BP	GO:190262	regulation	12/905	42/18866	4.02E-07	2.69E-06	1.41E-06	CXCL8/XCL
BP	GO:003110	animal orga	16/905	75/18866	4.11E-07	2.74E-06	1.44E-06	CXCL12/IL1
BP	GO:000836	regulation	24/905	156/18866	4.19E-07	2.79E-06	1.47E-06	ICAM1/CCL
BP	GO:000743	salivary gla	11/905	35/18866	4.21E-07	2.80E-06	1.47E-06	TGFB1/TNF
BP	GO:003263	interleukin	11/905	35/18866	4.21E-07	2.80E-06	1.47E-06	FCER1G/CD
BP	GO:004506	regulatory	11/905	35/18866	4.21E-07	2.80E-06	1.47E-06	HLA-G/IFN(
BP	GO:000276	positive reg	14/905	58/18866	4.36E-07	2.89E-06	1.52E-06	CD4/IFNG/
BP	GO:005159	response to	18/905	94/18866	4.38E-07	2.90E-06	1.52E-06	HSPA5/NO
BP	GO:190403	regulation	18/905	94/18866	4.38E-07	2.90E-06	1.52E-06	HLA-G/ICAI
BP	GO:004685	regulation	13/905	50/18866	4.44E-07	2.94E-06	1.54E-06	TFRC/TNFA
BP	GO:001604	detection c	8/905	17/18866	4.49E-07	2.96E-06	1.56E-06	HLA-A/HLA
BP	GO:003269	negative re	8/905	17/18866	4.49E-07	2.96E-06	1.56E-06	TLR8/CCR7
BP	GO:001077	positive reg	24/905	157/18866	4.72E-07	3.11E-06	1.64E-06	CALR/CXCL
BP	GO:004315	negative re	17/905	85/18866	4.78E-07	3.14E-06	1.65E-06	MMP9/TNI
BP	GO:004567	regulation	15/905	67/18866	4.86E-07	3.19E-06	1.68E-06	IFNG/LTF/T
BP	GO:000195	regulation	21/905	125/18866	4.95E-07	3.25E-06	1.71E-06	MMP12/PL
BP	GO:003307	T cell differ	16/905	76/18866	4.97E-07	3.26E-06	1.71E-06	B2M/MR1/
BP	GO:200010	positive reg	10/905	29/18866	5.34E-07	3.50E-06	1.84E-06	IDO1/CCL5
BP	GO:005070	regulation	14/905	59/18866	5.46E-07	3.57E-06	1.88E-06	IFNG/TLR2/
BP	GO:000276	negative re	13/905	51/18866	5.70E-07	3.72E-06	1.96E-06	LTF/TLR3/A
BP	GO:004871	positive reg	9/905	23/18866	5.75E-07	3.75E-06	1.97E-06	TLR2/HDAC
BP	GO:000189	maternal p	11/905	36/18866	5.80E-07	3.77E-06	1.98E-06	CTSB/MAP
BP	GO:003328	response to	11/905	36/18866	5.80E-07	3.77E-06	1.98E-06	CD4/CXCL1
BP	GO:003440	response to	11/905	36/18866	5.80E-07	3.77E-06	1.98E-06	SRC/PTGS2
BP	GO:000275	cytoplasmi	15/905	68/18866	5.96E-07	3.87E-06	2.03E-06	DDX58/IRF
BP	GO:007257	endothelia	15/905	68/18866	5.96E-07	3.87E-06	2.03E-06	HLA-G/ICAI
BP	GO:009009	regulation	32/905	254/18866	6.00E-07	3.89E-06	2.04E-06	HSPA5/HSF
BP	GO:003209	negative re	19/905	106/18866	6.17E-07	3.99E-06	2.10E-06	B2M/SLPI/
BP	GO:004209	T-helper ce	14/905	60/18866	6.81E-07	4.40E-06	2.31E-06	RELB/IL27/
BP	GO:003262	interleukin	12/905	44/18866	7.02E-07	4.53E-06	2.38E-06	MR1/IFNG/
BP	GO:190547	regulation	27/905	195/18866	7.08E-07	4.56E-06	2.40E-06	IFNG/TGFB
BP	GO:003295	regulation	40/905	360/18866	7.09E-07	4.57E-06	2.40E-06	ICAM1/CXC
BP	GO:004689	regulation	28/905	207/18866	7.17E-07	4.61E-06	2.42E-06	IFNG/NFYA
BP	GO:005105	positive reg	16/905	78/18866	7.21E-07	4.63E-06	2.44E-06	F2R/F2RL1/
BP	GO:003572	interleukin	7/905	13/18866	7.60E-07	4.85E-06	2.55E-06	CD4/IL15/I
BP	GO:006034	positive reg	7/905	13/18866	7.60E-07	4.85E-06	2.55E-06	MMP12/IR
BP	GO:007135	cellular res	7/905	13/18866	7.60E-07	4.85E-06	2.55E-06	CD4/IL15/I
BP	GO:007135	cellular res	7/905	13/18866	7.60E-07	4.85E-06	2.55E-06	PTK2B/IL2F
BP	GO:009753	mast cell m	7/905	13/18866	7.60E-07	4.85E-06	2.55E-06	VEGFA/CCL
BP	GO:190228	semaphorin	7/905	13/18866	7.60E-07	4.85E-06	2.55E-06	SEMA3A/P
BP	GO:009009	negative re	26/905	184/18866	7.62E-07	4.86E-06	2.55E-06	PSMB8/PSI

BP	GO:006082	regulation	35/905	295/18866	7.64E-07	4.86E-06	2.55E-06	PSMB8/PSI
BP	GO:009026	positive re	23/905	150/18866	7.71E-07	4.90E-06	2.58E-06	PSMB8/PSI
BP	GO:004573	respiratory	11/905	37/18866	7.90E-07	5.00E-06	2.63E-06	LBP/CYBB/
BP	GO:006220	positive re	11/905	37/18866	7.90E-07	5.00E-06	2.63E-06	TLR2/LBP/L
BP	GO:007154	dopaminer	11/905	37/18866	7.90E-07	5.00E-06	2.63E-06	VEGFA/WN
BP	GO:003286	response t	34/905	283/18866	8.21E-07	5.19E-06	2.73E-06	ICAM1/TLR
BP	GO:000926	response t	30/905	233/18866	8.45E-07	5.34E-06	2.80E-06	HSPA2/HSP
BP	GO:190204	regulation	14/905	61/18866	8.45E-07	5.34E-06	2.80E-06	ICAM1/TNI
BP	GO:007155	response t	32/905	258/18866	8.51E-07	5.36E-06	2.82E-06	HSPA5/HSP
BP	GO:004353	positive re	16/905	79/18866	8.65E-07	5.44E-06	2.86E-06	TMSB4X/T
BP	GO:000175	organ indu	9/905	24/18866	8.81E-07	5.52E-06	2.90E-06	WNT5A/FG
BP	GO:003275	positive re	9/905	24/18866	8.81E-07	5.52E-06	2.90E-06	FCER1G/CD
BP	GO:003412	positive re	9/905	24/18866	8.81E-07	5.52E-06	2.90E-06	TLR2/LBP/L
BP	GO:005077	negative re	15/905	70/18866	8.86E-07	5.55E-06	2.92E-06	WNT5A/PT
BP	GO:005237	modulation	12/905	45/18866	9.16E-07	5.73E-06	3.01E-06	CD4/CXCL8
BP	GO:003476	positive re	28/905	210/18866	9.62E-07	6.01E-06	3.16E-06	HSPA2/IFN
BP	GO:005105	regulation	40/905	365/18866	1.01E-06	6.31E-06	3.32E-06	HSP90AA1/
BP	GO:001051	positive re	14/905	62/18866	1.04E-06	6.51E-06	3.42E-06	CCL5/PDGF
BP	GO:200035	regulation	14/905	62/18866	1.04E-06	6.51E-06	3.42E-06	HLA-G/ICA
BP	GO:003139	regulation	28/905	211/18866	1.06E-06	6.59E-06	3.46E-06	HSPA5/HSP
BP	GO:007022	negative re	10/905	31/18866	1.08E-06	6.74E-06	3.54E-06	IDO1/CCL5
BP	GO:007030	cellular res	18/905	100/18866	1.14E-06	7.06E-06	3.71E-06	LCN2/TNFA
BP	GO:001086	positive re	12/905	46/18866	1.19E-06	7.35E-06	3.86E-06	PDGFRB/CI
BP	GO:003323	regulation	16/905	81/18866	1.23E-06	7.63E-06	4.01E-06	PSMB8/PSI
BP	GO:000271	negative re	8/905	19/18866	1.28E-06	7.92E-06	4.16E-06	HLA-F/HLA
BP	GO:009002	positive re	8/905	19/18866	1.28E-06	7.92E-06	4.16E-06	CXCL10/CX
BP	GO:004353	blood vess	26/905	189/18866	1.28E-06	7.92E-06	4.16E-06	TMSB4X/T
BP	GO:006103	regulation	15/905	72/18866	1.30E-06	8.00E-06	4.21E-06	WNT5A/BN
BP	GO:006033	regulation	9/905	25/18866	1.32E-06	8.11E-06	4.26E-06	HSP90AB1/
BP	GO:006033	regulation	9/905	25/18866	1.32E-06	8.11E-06	4.26E-06	HSP90AB1/
BP	GO:003050	BMP signal	23/905	155/18866	1.39E-06	8.52E-06	4.48E-06	TGFB1/HJV
BP	GO:000743	salivary gla	11/905	39/18866	1.42E-06	8.70E-06	4.57E-06	TGFB1/TNF
BP	GO:005135	positive re	13/905	55/18866	1.45E-06	8.89E-06	4.67E-06	IFNG/S100
BP	GO:001093	regulation	7/905	14/18866	1.46E-06	8.89E-06	4.67E-06	HLA-G/TGF
BP	GO:003231	prostaglan	7/905	14/18866	1.46E-06	8.89E-06	4.67E-06	IL1B/TNFSF
BP	GO:003268	negative re	7/905	14/18866	1.46E-06	8.89E-06	4.67E-06	RELB/NLRX
BP	GO:007066	response t	7/905	14/18866	1.46E-06	8.89E-06	4.67E-06	PTK2B/IL2F
BP	GO:007067	response t	7/905	14/18866	1.46E-06	8.89E-06	4.67E-06	CD4/IL15/I
BP	GO:007156	cellular res	31/905	252/18866	1.50E-06	9.14E-06	4.81E-06	HSPA5/HSP
BP	GO:003469	response t	10/905	32/18866	1.51E-06	9.16E-06	4.81E-06	TNFSF4/CC
BP	GO:003545	response t	10/905	32/18866	1.51E-06	9.16E-06	4.81E-06	TLR3/STAT
BP	GO:006103	positive re	10/905	32/18866	1.51E-06	9.16E-06	4.81E-06	WNT5A/BN
BP	GO:003572	interleukin	12/905	47/18866	1.52E-06	9.22E-06	4.85E-06	IFNG/PSMI
BP	GO:004663	positive re	12/905	47/18866	1.52E-06	9.22E-06	4.85E-06	IFNG/SOCS
BP	GO:001051	regulation	15/905	73/18866	1.56E-06	9.45E-06	4.96E-06	CCL5/PDGF
BP	GO:004545	bone resor	14/905	64/18866	1.57E-06	9.50E-06	4.99E-06	NOX4/TFR
BP	GO:005196	negative re	36/905	319/18866	1.76E-06	1.06E-05	5.59E-06	B2M/CALR
BP	GO:004518	maintenan	17/905	93/18866	1.80E-06	1.08E-05	5.70E-06	CD4/HSPA5
BP	GO:009018	regulation	13/905	56/18866	1.81E-06	1.09E-05	5.73E-06	MMP9/AD
BP	GO:190464	response t	13/905	56/18866	1.81E-06	1.09E-05	5.73E-06	ICAM1/MN

BP	GO:000271	positive re	11/905	40/18866	1.87E-06	1.12E-05	5.89E-06	FCER1G/LT
BP	GO:000289	positive re	11/905	40/18866	1.87E-06	1.12E-05	5.89E-06	FCER1G/LT
BP	GO:003414	toll-like rec	11/905	40/18866	1.87E-06	1.12E-05	5.89E-06	S100A14/L
BP	GO:009018	positive re	11/905	40/18866	1.87E-06	1.12E-05	5.89E-06	ADIPOQ/VI
BP	GO:190332	regulation	30/905	242/18866	1.88E-06	1.12E-05	5.91E-06	HSPA5/HSP
BP	GO:003164	killing of ce	14/905	65/18866	1.92E-06	1.14E-05	6.02E-06	IFNG/CCL1
BP	GO:000234	response to	9/905	26/18866	1.93E-06	1.15E-05	6.05E-06	HLA-A/MR1
BP	GO:000250	tolerance i	9/905	26/18866	1.93E-06	1.15E-05	6.05E-06	HLA-B/HLA
BP	GO:190027	regulation	12/905	48/18866	1.94E-06	1.16E-05	6.08E-06	PDGFRB/CI
BP	GO:003367	negative re	32/905	268/18866	1.96E-06	1.17E-05	6.14E-06	IFNG/IL1B/
BP	GO:003011	regulation	40/905	375/18866	2.00E-06	1.19E-05	6.25E-06	PSMB8/PSI
BP	GO:000272	positive re	8/905	20/18866	2.05E-06	1.21E-05	6.35E-06	B2M/HLA-A
BP	GO:003413	toll-like rec	8/905	20/18866	2.05E-06	1.21E-05	6.35E-06	TLR3/TNFA
BP	GO:005176	nitric-oxide	8/905	20/18866	2.05E-06	1.21E-05	6.35E-06	IFNG/TLR2/
BP	GO:005176	regulation	8/905	20/18866	2.05E-06	1.21E-05	6.35E-06	IFNG/TLR2/
BP	GO:005088	endocrine p	16/905	84/18866	2.05E-06	1.21E-05	6.35E-06	IL1B/CTSG/
BP	GO:000720	phospholip	18/905	104/18866	2.06E-06	1.21E-05	6.38E-06	F2R/F2RL1/
BP	GO:000269	regulation	10/905	33/18866	2.07E-06	1.22E-05	6.42E-06	ICAM1/CXC
BP	GO:007138	cellular res	27/905	206/18866	2.09E-06	1.23E-05	6.48E-06	CALR/ICAM
BP	GO:190595	positive re	17/905	94/18866	2.10E-06	1.24E-05	6.49E-06	IL1B/FABP3
BP	GO:005076	negative re	34/905	295/18866	2.12E-06	1.25E-05	6.55E-06	B2M/CALR/
BP	GO:000267	regulation	6/905	10/18866	2.13E-06	1.25E-05	6.57E-06	LTA/TNF/ID
BP	GO:003230	positive re	6/905	10/18866	2.13E-06	1.25E-05	6.57E-06	IL1B/TNFSF
BP	GO:000164	osteoblast	29/905	231/18866	2.15E-06	1.26E-05	6.63E-06	PSMC2/LTF
BP	GO:004568	regulation	15/905	75/18866	2.24E-06	1.31E-05	6.89E-06	TGFB1/TLR
BP	GO:000154	ovarian foll	13/905	57/18866	2.24E-06	1.31E-05	6.89E-06	ICAM1/VEG
BP	GO:003007	peptide ho	31/905	257/18866	2.28E-06	1.33E-05	7.01E-06	IFNG/IL1B/
BP	GO:000182	kidney dev	33/905	283/18866	2.31E-06	1.35E-05	7.08E-06	MMP9/AD
BP	GO:005500	cardiac mu	20/905	126/18866	2.31E-06	1.35E-05	7.08E-06	CALR/TGFB
BP	GO:004654	developme	18/905	105/18866	2.37E-06	1.38E-05	7.26E-06	HSPA5/ICA
BP	GO:004563	negative re	17/905	95/18866	2.44E-06	1.42E-05	7.46E-06	LTF/TLR3/A
BP	GO:003089	positive re	11/905	41/18866	2.45E-06	1.42E-05	7.46E-06	TFRC/CD40
BP	GO:190520	positive re	11/905	41/18866	2.45E-06	1.42E-05	7.46E-06	TGFB1/SEM
BP	GO:003413	toll-like rec	7/905	15/18866	2.62E-06	1.52E-05	7.97E-06	TLR2/TNFA
BP	GO:009059	inflammato	7/905	15/18866	2.62E-06	1.52E-05	7.97E-06	TGFB1/F2R
BP	GO:000646	negative re	30/905	246/18866	2.64E-06	1.53E-05	8.03E-06	IFNG/IL1B/
BP	GO:003476	positive re	23/905	161/18866	2.70E-06	1.56E-05	8.21E-06	HSPA2/IFN
BP	GO:001003	response to	39/905	366/18866	2.75E-06	1.59E-05	8.33E-06	B2M/CALR/
BP	GO:000756	embryo im	13/905	58/18866	2.77E-06	1.59E-05	8.38E-06	CALR/MMP
BP	GO:006007	canonical W	37/905	339/18866	2.81E-06	1.62E-05	8.52E-06	PSMB8/PSI
BP	GO:005121	cartilage de	26/905	197/18866	2.82E-06	1.62E-05	8.54E-06	TGFB1/MA
BP	GO:000177	leukocyte h	16/905	86/18866	2.83E-06	1.63E-05	8.55E-06	FCER1G/TN
BP	GO:004308	regulation	47/905	481/18866	3.08E-06	1.77E-05	9.30E-06	ICAM1/CXC
BP	GO:004564	regulation	12/905	50/18866	3.10E-06	1.78E-05	9.33E-06	B2M/STAT
BP	GO:009730	programm	12/905	50/18866	3.10E-06	1.78E-05	9.33E-06	IRF3/TNF/T
BP	GO:004664	positive re	8/905	21/18866	3.16E-06	1.81E-05	9.50E-06	HLA-A/XCL
BP	GO:009028	positive re	8/905	21/18866	3.16E-06	1.81E-05	9.50E-06	CXCL12/PD
BP	GO:009027	regulation	11/905	42/18866	3.17E-06	1.81E-05	9.50E-06	CXCL12/TR
BP	GO:009876	response to	11/905	42/18866	3.17E-06	1.81E-05	9.50E-06	PDIA3/SOC
BP	GO:009876	cellular res	11/905	42/18866	3.17E-06	1.81E-05	9.50E-06	PDIA3/SOC

BP	GO:19047(	regulation	16/905	87/18866	3.31E-06	1.89E-05	9.91E-06	MMP9/TNI
BP	GO:199087	vascular as	16/905	87/18866	3.31E-06	1.89E-05	9.91E-06	MMP9/TNI
BP	GO:00341(	erythrocyt	20/905	129/18866	3.36E-06	1.91E-05	1.00E-05	B2M/STAT
BP	GO:001973	antibacteri	13/905	59/18866	3.39E-06	1.92E-05	1.01E-05	HLA-A/SLPI
BP	GO:00480(	platelet-de	13/905	59/18866	3.39E-06	1.92E-05	1.01E-05	APOD/ADII
BP	GO:001993	cyclic-nucl	28/905	224/18866	3.49E-06	1.98E-05	1.04E-05	CXCL10/CX
BP	GO:00071٤	G protein-c	31/905	263/18866	3.71E-06	2.10E-05	1.10E-05	CXCL10/CX
BP	GO:007137	cellular res	36/905	330/18866	3.87E-06	2.18E-05	1.15E-05	IL1B/ADIP(
BP	GO:00328(	receptor bi	9/905	28/18866	3.91E-06	2.21E-05	1.16E-05	IFNG/TNF/
BP	GO:015007	regulation	11/905	43/18866	4.08E-06	2.30E-05	1.21E-05	MMP9/IL1I
BP	GO:009775	positive re	13/905	60/18866	4.14E-06	2.33E-05	1.23E-05	F2RL1/NOS
BP	GO:00032(	cardiac cha	20/905	131/18866	4.28E-06	2.40E-05	1.26E-05	TGFB1/RBI
BP	GO:004688	positive re	20/905	131/18866	4.28E-06	2.40E-05	1.26E-05	RBP4/TFR2
BP	GO:001093	macrophag	7/905	16/18866	4.46E-06	2.50E-05	1.31E-05	HLA-G/TGF
BP	GO:003303	negative re	7/905	16/18866	4.46E-06	2.50E-05	1.31E-05	FCER1G/C(
BP	GO:005177	positive re	7/905	16/18866	4.46E-06	2.50E-05	1.31E-05	IFNG/TLR2
BP	GO:00323(	regulation	6/905	11/18866	4.50E-06	2.52E-05	1.32E-05	IL1B/TNFSI
BP	GO:00701(	interleukin	6/905	11/18866	4.50E-06	2.52E-05	1.32E-05	STAT1/IL27
BP	GO:00435(	regulation	16/905	89/18866	4.52E-06	2.52E-05	1.33E-05	TNF/WNT5
BP	GO:200124	regulation	23/905	166/18866	4.58E-06	2.56E-05	1.34E-05	CXCL12/S1
BP	GO:00901(	positive re	18/905	110/18866	4.71E-06	2.63E-05	1.38E-05	HSP90AB1
BP	GO:000177	natural kill	8/905	22/18866	4.76E-06	2.65E-05	1.39E-05	PGLYRP1/P
BP	GO:00001٤	activation	12/905	52/18866	4.82E-06	2.68E-05	1.41E-05	F2R/EIF2AI
BP	GO:003021	erythrocyt	19/905	121/18866	4.84E-06	2.69E-05	1.41E-05	B2M/STAT
BP	GO:003237	positive re	14/905	70/18866	4.85E-06	2.70E-05	1.42E-05	IL1B/FABP3
BP	GO:200051	positive re	10/905	36/18866	4.99E-06	2.77E-05	1.46E-05	IFNG/XCL1
BP	GO:00023٤	immunoglo	13/905	61/18866	5.04E-06	2.79E-05	1.47E-05	HLA-DQB1
BP	GO:00085٤	female gor	17/905	100/18866	5.06E-06	2.80E-05	1.47E-05	HSPA5/ICA
BP	GO:005084	regulation	17/905	100/18866	5.06E-06	2.80E-05	1.47E-05	CD4/TNF/N
BP	GO:001082	regulation	25/905	191/18866	5.07E-06	2.80E-05	1.47E-05	PSMD10/M
BP	GO:00440(	regulation	11/905	44/18866	5.20E-06	2.87E-05	1.51E-05	IL1B/F2R/F
BP	GO:000231	B cell activ	15/905	80/18866	5.21E-06	2.87E-05	1.51E-05	TGFB1/TFR
BP	GO:000223	positive re	9/905	29/18866	5.43E-06	2.98E-05	1.57E-05	APOBEC3G
BP	GO:001983	cytolysis	9/905	29/18866	5.43E-06	2.98E-05	1.57E-05	MICA/MICI
BP	GO:004557	regulation	9/905	29/18866	5.43E-06	2.98E-05	1.57E-05	BTK/CARD1
BP	GO:00506(	regulation	9/905	29/18866	5.43E-06	2.98E-05	1.57E-05	B2M/CD4/
BP	GO:00217٤	glial cell de	19/905	122/18866	5.48E-06	3.00E-05	1.58E-05	IFNG/S100
BP	GO:004547	response to	19/905	122/18866	5.48E-06	3.00E-05	1.58E-05	ICAM1/PSN
BP	GO:007177	response to	23/905	168/18866	5.62E-06	3.08E-05	1.62E-05	TGFB1/HJV
BP	GO:007177	cellular res	23/905	168/18866	5.62E-06	3.08E-05	1.62E-05	TGFB1/HJV
BP	GO:001482	response to	14/905	71/18866	5.78E-06	3.15E-05	1.66E-05	ADIPOQ/C)
BP	GO:003362	cell adhesi	14/905	71/18866	5.78E-06	3.15E-05	1.66E-05	ICAM1/CX(
BP	GO:00356(	cellular res	14/905	71/18866	5.78E-06	3.15E-05	1.66E-05	HSPA5/HSI
BP	GO:004657	positive re	14/905	71/18866	5.78E-06	3.15E-05	1.66E-05	F2R/F2RL1
BP	GO:00071(	cell-matrix	28/905	230/18866	5.84E-06	3.18E-05	1.67E-05	MMP12/PL
BP	GO:004312	negative re	12/905	53/18866	5.96E-06	3.24E-05	1.70E-05	ADIPOQ/ST
BP	GO:006175	leukocyte a	12/905	53/18866	5.96E-06	3.24E-05	1.70E-05	ICAM1/CX(
BP	GO:00511(	negative re	23/905	169/18866	6.22E-06	3.37E-05	1.77E-05	B2M/SLPI/
BP	GO:009724	amyloid-be	10/905	37/18866	6.55E-06	3.55E-05	1.86E-05	IFNG/TNF/
BP	GO:19049(	regulation	10/905	37/18866	6.55E-06	3.55E-05	1.86E-05	ICAM1/CX(

BP	GO:003412	negative re	11/905	45/18866	6.59E-06	3.56E-05	1.87E-05	BPIFB1/TLR
BP	GO:190464	cellular res	11/905	45/18866	6.59E-06	3.56E-05	1.87E-05	ICAM1/AG
BP	GO:005502	regulation	17/905	102/18866	6.67E-06	3.60E-05	1.89E-05	TGFB1/RBP
BP	GO:003017	negative re	27/905	219/18866	6.73E-06	3.63E-05	1.91E-05	PSMB8/PSI
BP	GO:003236	regulation	20/905	135/18866	6.83E-06	3.68E-05	1.93E-05	IL1B/FABP3
BP	GO:004259	glucose ho	29/905	245/18866	6.97E-06	3.75E-05	1.97E-05	ICAM1/RBI
BP	GO:003269	negative re	8/905	23/18866	7.00E-06	3.75E-05	1.97E-05	IDO1/AGEF
BP	GO:003574	CD4-positiv	8/905	23/18866	7.00E-06	3.75E-05	1.97E-05	XCL1/IL1B/
BP	GO:006044	branching i	8/905	23/18866	7.00E-06	3.75E-05	1.97E-05	TNF/FGF10
BP	GO:002264	regulation	7/905	17/18866	7.27E-06	3.88E-05	2.04E-05	HLA-B/HLA
BP	GO:001081	regulation	7/905	17/18866	7.27E-06	3.88E-05	2.04E-05	CXCL10/CX
BP	GO:001573	prostaglan	7/905	17/18866	7.27E-06	3.88E-05	2.04E-05	IL1B/TNFSF
BP	GO:003054	female gen	7/905	17/18866	7.27E-06	3.88E-05	2.04E-05	RBP4/WNT
BP	GO:004454	NLRP3 infla	7/905	17/18866	7.27E-06	3.88E-05	2.04E-05	EIF2AK2/D
BP	GO:004476	multi-orga	7/905	17/18866	7.27E-06	3.88E-05	2.04E-05	CD4/CTSL/
BP	GO:004853	lymph nod	7/905	17/18866	7.27E-06	3.88E-05	2.04E-05	LTA/TGFB1
BP	GO:001052	positive re	12/905	54/18866	7.34E-06	3.90E-05	2.05E-05	CD4/CXCL1
BP	GO:000652	regulation	13/905	63/18866	7.35E-06	3.91E-05	2.05E-05	PSMB8/PSI
BP	GO:004337	positive re	9/905	30/18866	7.43E-06	3.95E-05	2.07E-05	IFNG/SOCS
BP	GO:000974	response t	28/905	233/18866	7.49E-06	3.97E-05	2.09E-05	ICAM1/IL1I
BP	GO:003350	carbohydra	29/905	246/18866	7.55E-06	4.00E-05	2.10E-05	ICAM1/RBI
BP	GO:000863	intrinsic ap	17/905	103/18866	7.64E-06	4.05E-05	2.13E-05	CXCL12/TN
BP	GO:000989	positive re	44/905	454/18866	7.81E-06	4.13E-05	2.17E-05	HSP90AA1,
BP	GO:000301	renal syste	19/905	125/18866	7.88E-06	4.16E-05	2.19E-05	ADIPOQ/F2
BP	GO:000863	apoptotic r	19/905	125/18866	7.88E-06	4.16E-05	2.19E-05	PSMD10/M
BP	GO:003269	negative re	11/905	46/18866	8.29E-06	4.37E-05	2.30E-05	TNFAIP3/IL
BP	GO:004558	negative re	11/905	46/18866	8.29E-06	4.37E-05	2.30E-05	IFNL1/SOC
BP	GO:003022	monocyte	10/905	38/18866	8.51E-06	4.48E-05	2.35E-05	CD4/VEGF
BP	GO:190313	mononucle	10/905	38/18866	8.51E-06	4.48E-05	2.35E-05	CD4/VEGF
BP	GO:000286	positive re	6/905	12/18866	8.63E-06	4.53E-05	2.38E-05	FCER1G/LT
BP	GO:007188	macrophag	6/905	12/18866	8.63E-06	4.53E-05	2.38E-05	CTSL/IRF3/
BP	GO:190228	semaphori	6/905	12/18866	8.63E-06	4.53E-05	2.38E-05	SEMA3A/P
BP	GO:007026	necrotic ce	13/905	64/18866	8.82E-06	4.62E-05	2.43E-05	IRF3/TNF/T
BP	GO:004577	positive re	16/905	94/18866	9.38E-06	4.91E-05	2.58E-05	TGFB1/LTF
BP	GO:004350	positive re	14/905	74/18866	9.58E-06	5.01E-05	2.63E-05	TNF/WNT5
BP	GO:003277	positive re	9/905	31/18866	1.00E-05	5.23E-05	2.75E-05	IFNG/S100
BP	GO:003952	cytoplasmi	9/905	31/18866	1.00E-05	5.23E-05	2.75E-05	DDX58/IRF
BP	GO:001046	regulation	23/905	174/18866	1.02E-05	5.30E-05	2.78E-05	IFNG/PLAU
BP	GO:001057	vascular er	13/905	65/18866	1.05E-05	5.49E-05	2.88E-05	TGFB1/IL1I
BP	GO:009858	cellular res	13/905	65/18866	1.05E-05	5.49E-05	2.88E-05	MMP12/DI
BP	GO:004340	steroid hor	20/905	139/18866	1.07E-05	5.56E-05	2.92E-05	CALR/SRC/
BP	GO:003266	regulation	10/905	39/18866	1.10E-05	5.69E-05	2.99E-05	IFNG/TGFB
BP	GO:004566	negative re	27/905	225/18866	1.11E-05	5.77E-05	3.03E-05	B2M/CALR
BP	GO:003803	signal trans	14/905	75/18866	1.13E-05	5.84E-05	3.07E-05	LCN2/IL1B/
BP	GO:009719	extrinsic ap	14/905	75/18866	1.13E-05	5.84E-05	3.07E-05	LCN2/IL1B/
BP	GO:001088	regulation	7/905	18/18866	1.14E-05	5.89E-05	3.10E-05	MSR1/PPA
BP	GO:004688	regulation	7/905	18/18866	1.14E-05	5.89E-05	3.10E-05	ADM/BMP
BP	GO:200118	regulation	7/905	18/18866	1.14E-05	5.89E-05	3.10E-05	HLA-A/XCL
BP	GO:003133	positive re	39/905	390/18866	1.24E-05	6.40E-05	3.37E-05	HSP90AA1,
BP	GO:000256	somatic div	13/905	66/18866	1.26E-05	6.46E-05	3.40E-05	TGFB1/TFR

BP	GO:00164	somatic ce	13/905	66/18866	1.26E-05	6.46E-05	3.40E-05	TGFB1/TFR
BP	GO:00327	regulation	13/905	66/18866	1.26E-05	6.46E-05	3.40E-05	HSP90AA1,
BP	GO:00094	response t	11/905	48/18866	1.29E-05	6.61E-05	3.47E-05	HSPA2/HSF
BP	GO:00507	positive re	15/905	86/18866	1.31E-05	6.71E-05	3.53E-05	CXCL12/VE
BP	GO:00601	positive re	14/905	76/18866	1.32E-05	6.78E-05	3.56E-05	CCL5/PDGF
BP	GO:00020	regulation	12/905	57/18866	1.32E-05	6.78E-05	3.57E-05	VEGFA/ARI
BP	GO:00458	positive re	12/905	57/18866	1.32E-05	6.78E-05	3.57E-05	IL1B/IL1A/I
BP	GO:00508	negative re	9/905	32/18866	1.34E-05	6.83E-05	3.59E-05	TNFAIP3/IL
BP	GO:19018	negative re	9/905	32/18866	1.34E-05	6.83E-05	3.59E-05	TLR2/IL1B/
BP	GO:00466	negative re	10/905	40/18866	1.40E-05	7.13E-05	3.75E-05	XCL1/SOCS
BP	GO:00487	regulation	10/905	40/18866	1.40E-05	7.13E-05	3.75E-05	TLR2/HDAC
BP	GO:00605	morphoger	8/905	25/18866	1.42E-05	7.22E-05	3.80E-05	WNT5A/FG
BP	GO:00108	positive re	18/905	119/18866	1.45E-05	7.40E-05	3.89E-05	MMP9/TNI
BP	GO:00325	response t	17/905	108/18866	1.46E-05	7.43E-05	3.90E-05	MICB/RBP4
BP	GO:00600	Wnt signali	17/905	108/18866	1.46E-05	7.43E-05	3.90E-05	PSMB8/PSI
BP	GO:00026	respiratory	6/905	13/18866	1.54E-05	7.79E-05	4.09E-05	LBP/MPO/I
BP	GO:00107	negative re	6/905	13/18866	1.54E-05	7.79E-05	4.09E-05	CETP/ADIP
BP	GO:00432	proteasom	6/905	13/18866	1.54E-05	7.79E-05	4.09E-05	PSMD4/PS
BP	GO:19029	regulation	6/905	13/18866	1.54E-05	7.79E-05	4.09E-05	HSP90AA1,
BP	GO:00022	somatic div	14/905	77/18866	1.54E-05	7.81E-05	4.10E-05	TGFB1/TFR
BP	GO:00604	cardiac sep	14/905	77/18866	1.54E-05	7.81E-05	4.10E-05	WNT5A/SE
BP	GO:00313	positive re	39/905	394/18866	1.57E-05	7.93E-05	4.17E-05	HSPA5/CXC
BP	GO:19037	positive re	12/905	58/18866	1.60E-05	8.06E-05	4.24E-05	IL1B/FABP3
BP	GO:00450	T-helper 1	7/905	19/18866	1.73E-05	8.71E-05	4.58E-05	RELB/IL27/
BP	GO:00607	mammary	7/905	19/18866	1.73E-05	8.71E-05	4.58E-05	VEGFA/TNI
BP	GO:00613	mammary	7/905	19/18866	1.73E-05	8.71E-05	4.58E-05	VEGFA/TNI
BP	GO:00508	regulation	15/905	88/18866	1.74E-05	8.75E-05	4.60E-05	FCER1G/PF
BP	GO:00028	regulation	9/905	33/18866	1.76E-05	8.83E-05	4.64E-05	FCER1G/LT
BP	GO:00457	positive re	39/905	396/18866	1.76E-05	8.84E-05	4.65E-05	CALR/HSPA
BP	GO:00423	vasoconstr	14/905	78/18866	1.80E-05	9.02E-05	4.74E-05	ICAM1/F2F
BP	GO:00313	positive re	18/905	121/18866	1.84E-05	9.19E-05	4.83E-05	HSPA5/PSN
BP	GO:00901	regulation	17/905	110/18866	1.87E-05	9.37E-05	4.92E-05	PSMB8/PSI
BP	GO:00140	glial cell pr	11/905	50/18866	1.95E-05	9.72E-05	5.11E-05	LTA/IL1B/T
BP	GO:20007	regulation	11/905	50/18866	1.95E-05	9.72E-05	5.11E-05	TGFB1/BM
BP	GO:00316	regulation	8/905	26/18866	1.96E-05	9.78E-05	5.14E-05	LTF/TNFAIF
BP	GO:00108	positive re	18/905	122/18866	2.06E-05	0.000103	5.39E-05	CALR/VEGF
BP	GO:20002	regulation	22/905	170/18866	2.20E-05	0.00011	5.76E-05	CALR/DEFE
BP	GO:19040	positive re	10/905	42/18866	2.22E-05	0.000111	5.82E-05	HLA-G/CD4
BP	GO:00028	positive re	9/905	34/18866	2.29E-05	0.000114	5.98E-05	FCER1G/DI
BP	GO:00072	activation	9/905	34/18866	2.29E-05	0.000114	5.98E-05	CD86/C5AF
BP	GO:00192	regulation	12/905	60/18866	2.30E-05	0.000114	5.99E-05	ICAM1/F2F
BP	GO:00352	exocrine sy	11/905	51/18866	2.38E-05	0.000118	6.19E-05	TGFB1/TNF
BP	GO:00601	regulation	16/905	101/18866	2.39E-05	0.000118	6.21E-05	CCL5/PDGF
BP	GO:00020	sprouting a	24/905	196/18866	2.41E-05	0.000119	6.27E-05	S100A1/VE
BP	GO:00303	positive re	22/905	171/18866	2.41E-05	0.000119	6.27E-05	PSMD10/C
BP	GO:00031	outflow tra	14/905	80/18866	2.43E-05	0.00012	6.30E-05	VEGFA/WN
BP	GO:00025	cytokine pr	13/905	70/18866	2.44E-05	0.00012	6.33E-05	APOD/TNF,
BP	GO:00716	anatomical	27/905	235/18866	2.45E-05	0.000121	6.35E-05	DEFB1/PAE
BP	GO:19040	positive re	20/905	147/18866	2.47E-05	0.000122	6.40E-05	HSPA2/IFN
BP	GO:00108	cholesterol	7/905	20/18866	2.55E-05	0.000125	6.59E-05	MSR1/PPA

BP	GO:003281	positive reg	7/905	20/18866	2.55E-05	0.000125	6.59E-05	HLA-F/IL15
BP	GO:200019	positive reg	7/905	20/18866	2.55E-05	0.000125	6.59E-05	IL1B/FABP3
BP	GO:000173	establishm	18/905	124/18866	2.58E-05	0.000126	6.64E-05	PSMB8/PSI
BP	GO:007161	establishm	18/905	124/18866	2.58E-05	0.000126	6.64E-05	PSMB8/PSI
BP	GO:007048	leukocyte a	6/905	14/18866	2.58E-05	0.000126	6.64E-05	S100A9/S1
BP	GO:007221	positive reg	6/905	14/18866	2.58E-05	0.000126	6.64E-05	ADIPOQ/PI
BP	GO:000635	DNA-templ	28/905	249/18866	2.59E-05	0.000127	6.66E-05	PSMC2/PSI
BP	GO:001097	positive reg	31/905	290/18866	2.66E-05	0.00013	6.84E-05	HSPA5/CXC
BP	GO:004567	positive reg	8/905	27/18866	2.67E-05	0.00013	6.85E-05	IFNG/TNF/
BP	GO:001470	striated mu	38/905	389/18866	2.70E-05	0.000131	6.91E-05	CALR/S100
BP	GO:001057	regulation	12/905	61/18866	2.73E-05	0.000133	6.99E-05	TGFB1/IL1B
BP	GO:004557	mast cell a	12/905	61/18866	2.73E-05	0.000133	6.99E-05	FCER1G/NI
BP	GO:004688	negative re	12/905	61/18866	2.73E-05	0.000133	6.99E-05	IL1B/ADIPC
BP	GO:004512	regulation	10/905	43/18866	2.77E-05	0.000135	7.09E-05	TFRC/TNFA
BP	GO:000196	negative re	14/905	81/18866	2.81E-05	0.000136	7.16E-05	MMP12/AI
BP	GO:004269	muscle cell	38/905	390/18866	2.85E-05	0.000138	7.27E-05	CALR/CXCL
BP	GO:005178	positive reg	13/905	71/18866	2.86E-05	0.000139	7.28E-05	IL1B/WNT5
BP	GO:003233	regulation	11/905	52/18866	2.88E-05	0.00014	7.34E-05	FGF18/GDI
BP	GO:001993	cAMP-med	24/905	199/18866	3.10E-05	0.00015	7.88E-05	CXCL10/CX
BP	GO:004573	positive reg	26/905	225/18866	3.12E-05	0.000151	7.92E-05	HSP90AA1/
BP	GO:000320	cardiac cha	22/905	174/18866	3.17E-05	0.000153	8.04E-05	TGFB1/RBF
BP	GO:003019	regulation	14/905	82/18866	3.24E-05	0.000156	8.20E-05	FCER1G/PL
BP	GO:003411	heterotypi	12/905	62/18866	3.24E-05	0.000156	8.22E-05	IL1B/TNF/A
BP	GO:000686	lipid transp	38/905	393/18866	3.38E-05	0.000163	8.54E-05	FABP6/IL1B
BP	GO:009027	regulation	25/905	213/18866	3.39E-05	0.000163	8.57E-05	IFNG/IL1B/
BP	GO:000301	muscle sys	43/905	467/18866	3.41E-05	0.000164	8.62E-05	IL1B/IL15/F
BP	GO:199077	response to	8/905	28/18866	3.58E-05	0.000172	9.04E-05	CYBB/SRC/
BP	GO:006053	muscle tiss	39/905	409/18866	3.64E-05	0.000175	9.19E-05	CALR/S100
BP	GO:007257	hepatocyte	7/905	21/18866	3.67E-05	0.000176	9.25E-05	TNFAIP3/M
BP	GO:007257	epithelial c	7/905	21/18866	3.67E-05	0.000176	9.25E-05	TNFAIP3/M
BP	GO:003114	anaphase-p	14/905	83/18866	3.72E-05	0.000178	9.36E-05	PSMB8/PSI
BP	GO:190004	regulation	14/905	83/18866	3.72E-05	0.000178	9.36E-05	FCER1G/PL
BP	GO:003019	extracellula	38/905	395/18866	3.77E-05	0.00018	9.43E-05	CTSL/ICAM
BP	GO:001009	specificatio	9/905	36/18866	3.77E-05	0.00018	9.43E-05	WNT5A/FG
BP	GO:003112	developme	9/905	36/18866	3.77E-05	0.00018	9.43E-05	WNT5A/FG
BP	GO:004562	regulation	9/905	36/18866	3.77E-05	0.00018	9.43E-05	IL27/TNFSF
BP	GO:190533	positive reg	9/905	36/18866	3.77E-05	0.00018	9.43E-05	VEGFA/AG
BP	GO:003153	actin cytos	16/905	105/18866	3.91E-05	0.000186	9.77E-05	PTK2B/HCK
BP	GO:004666	male sex di	21/905	164/18866	3.94E-05	0.000187	9.83E-05	ICAM1/TLR
BP	GO:001087	lipid localiz	41/905	440/18866	3.94E-05	0.000187	9.83E-05	FABP6/IL1B
BP	GO:004306	extracellula	38/905	396/18866	3.98E-05	0.000189	9.93E-05	CTSL/ICAM
BP	GO:000228	macrophag	6/905	15/18866	4.13E-05	0.000195	0.000102	IFNG/LBP/I
BP	GO:003563	entry of ba	6/905	15/18866	4.13E-05	0.000195	0.000102	CXCL8/ITGA
BP	GO:004297	activation c	6/905	15/18866	4.13E-05	0.000195	0.000102	SOCS1/CCL
BP	GO:006009	regulation	6/905	15/18866	4.13E-05	0.000195	0.000102	F2RL1/PPA
BP	GO:009720	activation c	6/905	15/18866	4.13E-05	0.000195	0.000102	IFI16/RIPK2
BP	GO:190022	regulation	6/905	15/18866	4.13E-05	0.000195	0.000102	EIF2AK2/D
BP	GO:200034	regulation	6/905	15/18866	4.13E-05	0.000195	0.000102	TNFAIP3/M
BP	GO:000197	blood vess	10/905	45/18866	4.23E-05	0.000199	0.000104	TGFB1/DLL
BP	GO:000263	positive reg	10/905	45/18866	4.23E-05	0.000199	0.000104	XCL1/TGFB

BP	GO:004311	regulation	10/905	45/18866	4.23E-05	0.000199	0.000104	TGFB1/VEC
BP	GO:00604	mammary	10/905	45/18866	4.23E-05	0.000199	0.000104	PML/WNT5
BP	GO:00702	necroptoti	10/905	45/18866	4.23E-05	0.000199	0.000104	TNF/TLR3/
BP	GO:00302	negative re	14/905	84/18866	4.27E-05	0.0002	0.000105	TNF/CCL3/
BP	GO:00192	sensory pe	16/905	106/18866	4.40E-05	0.000206	0.000108	CXCL12/FA
BP	GO:19030	positive re	13/905	74/18866	4.51E-05	0.000211	0.000111	DUOX1/DU
BP	GO:00325	maintenan	12/905	64/18866	4.52E-05	0.000211	0.000111	CD4/HSPA5
BP	GO:00027	positive re	8/905	29/18866	4.74E-05	0.000222	0.000116	HLA-F/HLA
BP	GO:20002	positive re	9/905	37/18866	4.77E-05	0.000223	0.000117	IFNG/CCL2
BP	GO:00320	positive re	14/905	85/18866	4.89E-05	0.000228	0.00012	B2M/HSP9
BP	GO:00030	heart morp	28/905	258/18866	4.92E-05	0.000229	0.00012	TGFB1/RBF
BP	GO:00108	negative re	7/905	22/18866	5.17E-05	0.000238	0.000125	TNF/ITGAV
BP	GO:00361	cellular res	7/905	22/18866	5.17E-05	0.000238	0.000125	SRC/PDGFF
BP	GO:00450	thymic T ce	7/905	22/18866	5.17E-05	0.000238	0.000125	CCR7/CARI
BP	GO:00711	negative re	7/905	22/18866	5.17E-05	0.000238	0.000125	CALR/HSP9
BP	GO:00725	liver morpl	7/905	22/18866	5.17E-05	0.000238	0.000125	TNFAIP3/N
BP	GO:00031	heart indu	5/905	10/18866	5.17E-05	0.000238	0.000125	WNT5A/RC
BP	GO:00328	negative re	5/905	10/18866	5.17E-05	0.000238	0.000125	HLA-F/MIC
BP	GO:00349	protein fol	5/905	10/18866	5.17E-05	0.000238	0.000125	CALR/PDIA
BP	GO:00456	regulation	5/905	10/18866	5.17E-05	0.000238	0.000125	IL27/TNFSF
BP	GO:00600	vagina dev	5/905	10/18866	5.17E-05	0.000238	0.000125	RBP4/WNT
BP	GO:00606	dichotomo	5/905	10/18866	5.17E-05	0.000238	0.000125	SEMA3A/S
BP	GO:00703	response t	5/905	10/18866	5.17E-05	0.000238	0.000125	TLR2/LBP/
BP	GO:00712	cellular res	5/905	10/18866	5.17E-05	0.000238	0.000125	TLR2/LBP/
BP	GO:00900	positive re	5/905	10/18866	5.17E-05	0.000238	0.000125	CD40/VEGI
BP	GO:00323	icosanoid s	10/905	46/18866	5.18E-05	0.000238	0.000125	IL1B/PLA2C
BP	GO:00359	cellular res	13/905	75/18866	5.22E-05	0.00024	0.000126	VEGFA/DLL
BP	GO:00486	animal org	12/905	65/18866	5.30E-05	0.000244	0.000128	MAPK3/WI
BP	GO:19049	negative re	20/905	155/18866	5.35E-05	0.000246	0.000129	IL1B/APOD
BP	GO:00192	regulation	18/905	131/18866	5.44E-05	0.000249	0.000131	IFNG/NFYA
BP	GO:00456	regulation	18/905	131/18866	5.44E-05	0.000249	0.000131	LTF/TNF/P
BP	GO:00977	negative re	14/905	86/18866	5.59E-05	0.000256	0.000135	ICAM1/F2F
BP	GO:00076	learning or	28/905	260/18866	5.64E-05	0.000258	0.000136	B2M/S100I
BP	GO:00071	adenylate	26/905	233/18866	5.67E-05	0.000259	0.000136	CXCL10/CX
BP	GO:19016	cellular res	15/905	97/18866	5.68E-05	0.000259	0.000136	ICAM1/GN
BP	GO:00109	negative re	35/905	359/18866	5.76E-05	0.000263	0.000138	CALR/HLA-
BP	GO:00342	response t	24/905	207/18866	5.88E-05	0.000268	0.000141	ICAM1/NO
BP	GO:00164	somatic re	11/905	56/18866	5.97E-05	0.000272	0.000143	TGFB1/TFR
BP	GO:00031	regulation	9/905	38/18866	5.99E-05	0.000272	0.000143	WNT5A/FG
BP	GO:00465	regulation	9/905	38/18866	5.99E-05	0.000272	0.000143	CD4/TRIM
BP	GO:19035	positive re	13/905	76/18866	6.02E-05	0.000274	0.000144	ICAM1/F2F
BP	GO:00070	cell cycle a	26/905	234/18866	6.09E-05	0.000277	0.000145	CALR/HSP9
BP	GO:00071	regulation	8/905	30/18866	6.19E-05	0.000281	0.000148	AREG/BTC/
BP	GO:00327	negative re	12/905	66/18866	6.20E-05	0.000281	0.000148	TNF/NLRX1
BP	GO:20002	regulation	16/905	109/18866	6.23E-05	0.000282	0.000148	HSP90AA1/
BP	GO:00903	positive re	22/905	182/18866	6.32E-05	0.000285	0.00015	HSP90AB1/
BP	GO:00028	positive re	6/905	16/18866	6.34E-05	0.000285	0.00015	XCL1/IDO1
BP	GO:00028	regulation	6/905	16/18866	6.34E-05	0.000285	0.00015	MR1/IL12A
BP	GO:00028	regulation	6/905	16/18866	6.34E-05	0.000285	0.00015	MR1/IL12A
BP	GO:00108	positive re	6/905	16/18866	6.34E-05	0.000285	0.00015	CXCL13/XC

BP	GO:003437	low-densit	6/905	16/18866	6.34E-05	0.000285	0.00015	CETP/PLA2
BP	GO:004801	hepatocyte	6/905	16/18866	6.34E-05	0.000285	0.00015	ESM1/MET
BP	GO:190515	regulation	6/905	16/18866	6.34E-05	0.000285	0.00015	F2RL1/PPA
BP	GO:006076	negative re	14/905	87/18866	6.37E-05	0.000286	0.000151	MMP12/AI
BP	GO:001077	negative re	15/905	98/18866	6.41E-05	0.000288	0.000151	WNT5A/PT
BP	GO:003050	regulation	13/905	77/18866	6.93E-05	0.000311	0.000164	TGFB1/LTF
BP	GO:003611	response t	7/905	23/18866	7.12E-05	0.000319	0.000167	SRC/PDGFI
BP	GO:007221	regulation	7/905	23/18866	7.12E-05	0.000319	0.000167	ADIPOQ/ST
BP	GO:190499	positive re	7/905	23/18866	7.12E-05	0.000319	0.000167	ICAM1/TNI
BP	GO:190001	regulation	12/905	67/18866	7.23E-05	0.000323	0.00017	APOD/TNF
BP	GO:004325	regulation	15/905	99/18866	7.23E-05	0.000323	0.00017	TGFB1/ADI
BP	GO:005130	regulation	21/905	171/18866	7.31E-05	0.000326	0.000172	TGFB1/IL1I
BP	GO:003315	regulation	28/905	264/18866	7.38E-05	0.000329	0.000173	HSP90AB1/
BP	GO:004516	cell fate co	29/905	278/18866	7.45E-05	0.000332	0.000174	CTSL/PML/
BP	GO:011005	regulation	29/905	278/18866	7.45E-05	0.000332	0.000174	ICAM1/CX(
BP	GO:003269	negative re	9/905	39/18866	7.46E-05	0.000332	0.000174	TNFAIP3/C
BP	GO:003195	regulation	10/905	48/18866	7.62E-05	0.000338	0.000178	ADIPOQ/VI
BP	GO:003362	regulation	10/905	48/18866	7.62E-05	0.000338	0.000178	CXCL13/PL
BP	GO:006024	anatomical	42/905	469/18866	7.90E-05	0.000351	0.000184	AZGP1/B2I
BP	GO:004390	regulation	13/905	78/18866	7.96E-05	0.000353	0.000186	CALR/PAEF
BP	GO:004594	positive re	8/905	31/18866	8.00E-05	0.000354	0.000186	IFNG/IL1B/
BP	GO:200051	negative re	8/905	31/18866	8.00E-05	0.000354	0.000186	XCL1/ARG2
BP	GO:000315	endotheliu	18/905	135/18866	8.11E-05	0.000359	0.000189	ICAM1/CX(
BP	GO:004566	positive re	36/905	380/18866	8.20E-05	0.000363	0.000191	HSPA5/CX(
BP	GO:000288	regulation	11/905	58/18866	8.36E-05	0.000369	0.000194	FCER1G/DI
BP	GO:005114	striated mu	30/905	295/18866	8.98E-05	0.000396	0.000208	CALR/CXCL
BP	GO:000309	regulation	5/905	11/18866	9.11E-05	0.000401	0.000211	F2R/F2RL1
BP	GO:004480	multi-orga	5/905	11/18866	9.11E-05	0.000401	0.000211	CD4/CTSL/
BP	GO:005113	NK T cell ac	5/905	11/18866	9.11E-05	0.000401	0.000211	IL15/IL12A
BP	GO:005171	positive re	5/905	11/18866	9.11E-05	0.000401	0.000211	IFNG/F2RL
BP	GO:001590	fatty acid t	15/905	101/18866	9.14E-05	0.000402	0.000211	IL1B/FABP!
BP	GO:009019	regulation	10/905	49/18866	9.16E-05	0.000402	0.000212	PSMD10/M
BP	GO:000199	regulation	9/905	40/18866	9.22E-05	0.000404	0.000212	CTSG/F2R/
BP	GO:003289	positive re	9/905	40/18866	9.22E-05	0.000404	0.000212	IL1B/FABP3
BP	GO:005128	positive re	9/905	40/18866	9.22E-05	0.000404	0.000212	CXCL10/CX
BP	GO:000725	activation	6/905	17/18866	9.40E-05	0.000411	0.000216	TLR3/TNFR
BP	GO:003230	positive re	6/905	17/18866	9.40E-05	0.000411	0.000216	IL1B/TNFSI
BP	GO:003268	negative re	6/905	17/18866	9.40E-05	0.000411	0.000216	APOD/ARG
BP	GO:007136	cellular res	6/905	17/18866	9.40E-05	0.000411	0.000216	DDX58/IRF
BP	GO:000243	acute infla	7/905	24/18866	9.64E-05	0.00042	0.00022	FCER1G/IC
BP	GO:003600	positive re	7/905	24/18866	9.64E-05	0.00042	0.00022	HSPA5/VE(
BP	GO:004406	regulation	7/905	24/18866	9.64E-05	0.00042	0.00022	EDNRB/AG
BP	GO:006044	branching i	7/905	24/18866	9.64E-05	0.00042	0.00022	PML/WNT!
BP	GO:003296	collagen m	16/905	113/18866	9.67E-05	0.000421	0.000221	CTSB/CTSL
BP	GO:200057	positive re	12/905	69/18866	9.73E-05	0.000423	0.000222	HSP90AA1/
BP	GO:004230	regulation	11/905	59/18866	9.84E-05	0.000427	0.000225	HSP90AB1/
BP	GO:003527	endocrine	17/905	125/18866	9.89E-05	0.000429	0.000225	MAPK3/WI
BP	GO:004350	muscle ada	17/905	125/18866	9.89E-05	0.000429	0.000225	IL1B/IL15/
BP	GO:000020	protein pol	33/905	340/18866	0.000101	0.000438	0.00023	PSMB8/PSI
BP	GO:006060	mammary	8/905	32/18866	0.000102	0.000442	0.000233	PML/WNT!

BP	GO:00313	negative re	22/905	188/18866	0.000103	0.000444	0.000234	B2M/WNT
BP	GO:00300	regionaliza	34/905	355/18866	0.000104	0.000448	0.000236	WNT5A/DL
BP	GO:00973	cellular res	14/905	91/18866	0.000105	0.000455	0.000239	CYBB/GNA
BP	GO:00032	cardiac sep	16/905	114/18866	0.000108	0.000464	0.000244	WNT5A/LT
BP	GO:00312	biomineral	20/905	163/18866	0.000109	0.000469	0.000247	HTN3/HTN
BP	GO:11001	biomineral	20/905	163/18866	0.000109	0.000469	0.000247	HTN3/HTN
BP	GO:00030	regulation	10/905	50/18866	0.00011	0.000471	0.000248	CTSG/F2R/
BP	GO:00717	icosanoid t	10/905	50/18866	0.00011	0.000471	0.000248	IL1B/PLA2
BP	GO:19015	fatty acid d	10/905	50/18866	0.00011	0.000471	0.000248	IL1B/PLA2
BP	GO:19047	positive re	10/905	50/18866	0.00011	0.000471	0.000248	MMP9/TNI
BP	GO:00097	response t	23/905	202/18866	0.00011	0.000471	0.000248	ICAM1/NO
BP	GO:00507	regulation	12/905	70/18866	0.000112	0.000482	0.000253	PTGDS/CCL
BP	GO:00335	response t	9/905	41/18866	0.000113	0.000485	0.000255	CALR/EPO/
BP	GO:00512	negative re	19/905	151/18866	0.000115	0.000491	0.000258	IL1B/APOD
BP	GO:00018	mesonephr	15/905	103/18866	0.000115	0.000491	0.000258	VEGFA/AR
BP	GO:00018	release of	11/905	60/18866	0.000115	0.000492	0.000259	PSMD10/M
BP	GO:00433	positive re	11/905	60/18866	0.000115	0.000492	0.000259	IFNG/TGFB
BP	GO:00435	regulation	20/905	164/18866	0.000119	0.000506	0.000266	TMSB4X/T
BP	GO:00199	lipid storag	13/905	81/18866	0.000119	0.000506	0.000266	IL1B/TNF/I
BP	GO:00512	regulation	13/905	81/18866	0.000119	0.000506	0.000266	CXCL10/CX
BP	GO:00030	renal syste	7/905	25/18866	0.000128	0.000546	0.000287	F2R/F2RL1
BP	GO:00972	renal filtrat	7/905	25/18866	0.000128	0.000546	0.000287	F2R/F2RL1
BP	GO:19058	negative re	7/905	25/18866	0.000128	0.000546	0.000287	TLR2/WNT
BP	GO:00483	retinoic aci	8/905	33/18866	0.000129	0.000549	0.000289	CALR/PML
BP	GO:00611	mammary	12/905	71/18866	0.000129	0.000549	0.000289	PML/WNT
BP	GO:00022	somatic re	10/905	51/18866	0.000131	0.000553	0.000291	TGFB1/TFR
BP	GO:00022	somatic div	10/905	51/18866	0.000131	0.000553	0.000291	TGFB1/TFR
BP	GO:00451	isotype swi	10/905	51/18866	0.000131	0.000553	0.000291	TGFB1/TFR
BP	GO:00720	stem cell p	17/905	128/18866	0.000133	0.000563	0.000296	VEGFA/EIF
BP	GO:00311	SCF-depen	14/905	93/18866	0.000134	0.000566	0.000297	PSMB8/PS
BP	GO:19030	negative re	14/905	93/18866	0.000134	0.000566	0.000297	PLAU/TNF/
BP	GO:00903	positive re	11/905	61/18866	0.000135	0.000568	0.000299	DUOX1/DU
BP	GO:00219	central ner	13/905	82/18866	0.000135	0.000569	0.000299	HSP90AA1
BP	GO:00702	positive re	6/905	18/18866	0.000135	0.000569	0.000299	IDO1/CCL5
BP	GO:00716	monocyte	6/905	18/18866	0.000135	0.000569	0.000299	IL1B/APOD
BP	GO:00716	regulation	6/905	18/18866	0.000135	0.000569	0.000299	IL1B/APOD
BP	GO:01500	positive re	6/905	18/18866	0.000135	0.000569	0.000299	IL1B/TNF/
BP	GO:00508	cognition	30/905	302/18866	0.000137	0.000575	0.000302	B2M/S100
BP	GO:00712	cellular res	32/905	331/18866	0.000138	0.000578	0.000304	HSPA5/REL
BP	GO:01040	cellular res	32/905	331/18866	0.000138	0.000578	0.000304	HSPA5/REL
BP	GO:00459	positive re	28/905	274/18866	0.00014	0.000587	0.000309	PSMD10/C
BP	GO:00094	response t	20/905	166/18866	0.00014	0.000587	0.000309	HSPA2/HS
BP	GO:00085	male gona	18/905	141/18866	0.000143	0.000597	0.000314	ICAM1/TLR
BP	GO:19033	positive re	18/905	141/18866	0.000143	0.000597	0.000314	HSPA5/PSN
BP	GO:00440	regulation	15/905	105/18866	0.000143	0.000597	0.000314	IL1B/FABP
BP	GO:00482	regulation	15/905	105/18866	0.000143	0.000597	0.000314	B2M/VEGF
BP	GO:00302	bone mine	16/905	117/18866	0.000147	0.000613	0.000322	TGFB1/LTF
BP	GO:19044	positive re	12/905	72/18866	0.000149	0.00062	0.000326	HSPA2/CX
BP	GO:00109	negative re	19/905	154/18866	0.000149	0.00062	0.000326	B2M/WNT
BP	GO:06135	neural pre	19/905	154/18866	0.000149	0.00062	0.000326	FABP7/VE

BP	GO:000251	T cell toler	5/905	12/18866	0.00015	0.000622	0.000327	HLA-B/HLA
BP	GO:000273	regulation	5/905	12/18866	0.00015	0.000622	0.000327	DDX58/TLF
BP	GO:003249	response t	5/905	12/18866	0.00015	0.000622	0.000327	IRF5/C5AR
BP	GO:006151	macrophag	5/905	12/18866	0.00015	0.000622	0.000327	MAPK3/PT
BP	GO:007049	thrombin-a	5/905	12/18866	0.00015	0.000622	0.000327	F2R/F2RL1,
BP	GO:200024	positive re	13/905	83/18866	0.000153	0.000634	0.000333	DEFB1/VEG
BP	GO:001088	regulation	10/905	52/18866	0.000155	0.000641	0.000337	TNF/ITGAV
BP	GO:005099	regulation	10/905	52/18866	0.000155	0.000641	0.000337	HSP90AA1,
BP	GO:004654	developme	18/905	142/18866	0.000156	0.000646	0.00034	ICAM1/TLF
BP	GO:190458	regulation	11/905	62/18866	0.000157	0.000647	0.00034	HSP90AB1,
BP	GO:003214	activation	8/905	34/18866	0.000162	0.000667	0.000351	WNT5A/SR
BP	GO:004564	positive re	8/905	34/18866	0.000162	0.000667	0.000351	STAT1/ISG1
BP	GO:005071	positive re	8/905	34/18866	0.000162	0.000667	0.000351	IFNG/IRF3/
BP	GO:006138	heart trabe	8/905	34/18866	0.000162	0.000667	0.000351	RBP4/DLL4
BP	GO:000756	cell aging	16/905	118/18866	0.000163	0.000669	0.000352	B2M/CALR,
BP	GO:004593	positive re	20/905	168/18866	0.000165	0.000679	0.000357	HSPA2/IL1
BP	GO:004316	proteasom	38/905	424/18866	0.000168	0.000689	0.000362	HSPA5/HSF
BP	GO:000308	regulation	7/905	26/18866	0.000168	0.00069	0.000363	CTSG/F2R/
BP	GO:003411	regulation	7/905	26/18866	0.000168	0.00069	0.000363	IL1B/TNF/
BP	GO:190073	regulation	7/905	26/18866	0.000168	0.00069	0.000363	GZMB/BID,
BP	GO:190074	positive re	7/905	26/18866	0.000168	0.00069	0.000363	GZMB/BID,
BP	GO:000307	regulation	14/905	95/18866	0.000169	0.00069	0.000363	CTSG/F2R/
BP	GO:007016	regulation	14/905	95/18866	0.000169	0.00069	0.000363	TGFB1/LTF,
BP	GO:011014	regulation	14/905	95/18866	0.000169	0.00069	0.000363	TGFB1/LTF,
BP	GO:004591	positive re	13/905	84/18866	0.000173	0.000708	0.000372	IFNG/SRC/
BP	GO:004348	regulation	22/905	195/18866	0.000175	0.000716	0.000376	PSMB8/PSI
BP	GO:190186	regulation	19/905	156/18866	0.000177	0.000721	0.000379	S100B/TGF
BP	GO:004410	cellular am	17/905	131/18866	0.000177	0.000722	0.000379	PSMB8/PSI
BP	GO:005115	regulation	16/905	119/18866	0.00018	0.000731	0.000384	CXCL10/CX
BP	GO:005160	protein ma	29/905	293/18866	0.000187	0.000761	0.0004	CALR/CTSL,
BP	GO:001648	protein pro	24/905	223/18866	0.000188	0.000766	0.000403	CTSL/PLAU
BP	GO:001097	negative re	14/905	96/18866	0.000189	0.000768	0.000404	PSMB8/PSI
BP	GO:001074	positive re	6/905	19/18866	0.00019	0.000768	0.000404	PLA2G2A/N
BP	GO:004509	regulation	6/905	19/18866	0.00019	0.000768	0.000404	CXCL8/APC
BP	GO:004578	positive re	6/905	19/18866	0.00019	0.000768	0.000404	TFRC/TNFS
BP	GO:004685	positive re	6/905	19/18866	0.00019	0.000768	0.000404	TFRC/TNFS
BP	GO:190042	regulation	6/905	19/18866	0.00019	0.000768	0.000404	F2RL1/SPIN
BP	GO:005134	negative re	41/905	473/18866	0.000193	0.00078	0.00041	SLPI/WFDC
BP	GO:004217	regulation	36/905	397/18866	0.000196	0.000793	0.000417	HSP90AA1,
BP	GO:000610	regulation	23/905	210/18866	0.000197	0.000794	0.000417	IFNG/TGFB
BP	GO:000206	chondrocyt	15/905	108/18866	0.000197	0.000794	0.000417	TGFB1/BM
BP	GO:000859	regulation	15/905	108/18866	0.000197	0.000794	0.000417	SRC/DLL4/
BP	GO:190275	negative re	15/905	108/18866	0.000197	0.000794	0.000417	PSMB8/PSI
BP	GO:003631	response t	8/905	35/18866	0.000201	0.00081	0.000426	TGFB1/CCL
BP	GO:190103	positive re	8/905	35/18866	0.000201	0.00081	0.000426	GSK3B/GZI
BP	GO:000974	response t	22/905	197/18866	0.000203	0.000817	0.000429	ICAM1/NO
BP	GO:000173	morphogei	18/905	145/18866	0.000204	0.000819	0.000431	PSMB8/PSI
BP	GO:003085	regulation	20/905	171/18866	0.00021	0.000843	0.000443	CTSL/IFNG,
BP	GO:200017	positive re	10/905	54/18866	0.000215	0.000861	0.000453	VEGFA/DLL
BP	GO:009028	regulation	30/905	310/18866	0.000216	0.000868	0.000456	HSPA5/HSF

BP	GO:003195	positive re	7/905	27/18866	0.000218	0.000871	0.000458	VEGFA/SRC
BP	GO:003289	negative re	7/905	27/18866	0.000218	0.000871	0.000458	CCL5/CCL4
BP	GO:003603	CD8-positiv	7/905	27/18866	0.000218	0.000871	0.000458	HLA-A/XCL
BP	GO:200035	positive re	7/905	27/18866	0.000218	0.000871	0.000458	HLA-G/CD4
BP	GO:000206	columnar/c	16/905	121/18866	0.000218	0.000872	0.000458	WNT5A/SR
BP	GO:003166	cellular res	26/905	253/18866	0.000219	0.000873	0.000459	HSPA5/ICA
BP	GO:004544	myoblast d	13/905	86/18866	0.000221	0.00088	0.000462	CXCL14/CX
BP	GO:000237	dendritic c	5/905	13/18866	0.000234	0.000927	0.000487	DDX58/TLF
BP	GO:003065	regulation	5/905	13/18866	0.000234	0.000927	0.000487	IFNG/IL1B/
BP	GO:003272	positive re	5/905	13/18866	0.000234	0.000927	0.000487	IL1B/PAEP/
BP	GO:003282	regulation	5/905	13/18866	0.000234	0.000927	0.000487	PGLYRP1/P
BP	GO:003800	netrin-activ	5/905	13/18866	0.000234	0.000927	0.000487	PTK2/TUBE
BP	GO:004574	positive re	5/905	13/18866	0.000234	0.000927	0.000487	AREG/BTC/
BP	GO:006010	positive re	5/905	13/18866	0.000234	0.000927	0.000487	F2RL1/PPA
BP	GO:007268	T cell extra	5/905	13/18866	0.000234	0.000927	0.000487	ICAM1/CCL
BP	GO:190515	positive re	5/905	13/18866	0.000234	0.000927	0.000487	F2RL1/PPA
BP	GO:200019	regulation	5/905	13/18866	0.000234	0.000927	0.000487	SRC/AMH/
BP	GO:005081	regulation	14/905	98/18866	0.000236	0.000933	0.00049	IFNG/NFYA
BP	GO:200124	negative re	14/905	98/18866	0.000236	0.000933	0.00049	CXCL12/MI
BP	GO:003410	positive re	9/905	45/18866	0.000241	0.000952	0.0005	TFRC/IL15/
BP	GO:004348	regulation	21/905	186/18866	0.000242	0.000954	0.000501	PSMB8/PSI
BP	GO:007058	calcium ior	30/905	312/18866	0.000242	0.000954	0.000501	HSPA2/CXC
BP	GO:003007	insulin secr	23/905	213/18866	0.000242	0.000954	0.000502	IFNG/IL1B/
BP	GO:000718	adenylate c	18/905	147/18866	0.000242	0.000955	0.000502	CXCL10/CX
BP	GO:007140	cellular res	8/905	36/18866	0.000248	0.000975	0.000512	FCER1G/PF
BP	GO:190109	negative re	8/905	36/18866	0.000248	0.000975	0.000512	IL1B/TNF/I
BP	GO:200124	negative re	8/905	36/18866	0.000248	0.000975	0.000512	IL1B/TNF/I
BP	GO:000194	hair follicle	13/905	87/18866	0.000248	0.000975	0.000512	TNF/WNT5
BP	GO:000195	positive re	10/905	55/18866	0.000251	0.000986	0.000518	VEGFA/LIM
BP	GO:007074	response to	10/905	55/18866	0.000251	0.000986	0.000518	ICAM1/STA
BP	GO:003355	multicellula	12/905	76/18866	0.000252	0.000986	0.000518	IDO1/NOS1
BP	GO:005080	synapse or	38/905	433/18866	0.000256	0.001001	0.000526	TLR2/TNF/
BP	GO:000257	regulation	6/905	20/18866	0.00026	0.001013	0.000533	HLA-DOB/S
BP	GO:000725	I-kappaB pl	6/905	20/18866	0.00026	0.001013	0.000533	TLR2/TNF/T
BP	GO:003230	regulation	6/905	20/18866	0.00026	0.001013	0.000533	IL1B/TNFSF
BP	GO:003233	positive re	6/905	20/18866	0.00026	0.001013	0.000533	FGF18/GDI
BP	GO:003249	response to	6/905	20/18866	0.00026	0.001013	0.000533	TNFAIP3/IF
BP	GO:003969	single strar	6/905	20/18866	0.00026	0.001013	0.000533	CXCL8/APC
BP	GO:190593	regulation	6/905	20/18866	0.00026	0.001013	0.000533	SRC/SEMA3
BP	GO:009027	positive re	14/905	99/18866	0.000263	0.001024	0.000538	RBP4/TFR2
BP	GO:009025	regulation	26/905	256/18866	0.000263	0.001025	0.000539	F2R/PTGS2
BP	GO:001067	regulation	18/905	148/18866	0.000264	0.001026	0.000539	ADIPOQ/SF
BP	GO:000701	actin filam	38/905	434/18866	0.000268	0.00104	0.000547	ICAM1/CXC
BP	GO:001644	somatic div	11/905	66/18866	0.000278	0.001079	0.000567	TGFB1/TFR
BP	GO:004390	negative re	7/905	28/18866	0.000279	0.00108	0.000567	CALR/PAEF
BP	GO:009020	positive re	7/905	28/18866	0.000279	0.00108	0.000567	MMP9/TNI
BP	GO:200019	regulation	7/905	28/18866	0.000279	0.00108	0.000567	IL1B/FABP3
BP	GO:003257	response to	9/905	46/18866	0.000287	0.001111	0.000584	TGFB1/TLR
BP	GO:005110	regulation	16/905	124/18866	0.00029	0.001123	0.00059	IFNG/TMSE
BP	GO:001033	response to	10/905	56/18866	0.000293	0.001132	0.000595	HSPA5/CXC

BP	GO:00104€	negative re	27/905	272/18866	0.000293	0.001132	0.000595	SLPI/WFDC
BP	GO:004682	regulation	15/905	112/18866	0.000295	0.001141	0.000599	HSP90AB1/
BP	GO:001095	negative re	26/905	258/18866	0.000298	0.001148	0.000603	SLPI/WFDC
BP	GO:00104S	proteasom	41/905	483/18866	0.000299	0.001151	0.000605	HSPA5/HSF
BP	GO:00514S	positive re	24/905	230/18866	0.0003	0.001155	0.000607	ICAM1/NO
BP	GO:004354	positive re	36/905	406/18866	0.000302	0.001164	0.000612	ICAM1/CX(
BP	GO:00451S	regulation	8/905	37/18866	0.000303	0.001166	0.000613	TGFB1/TFR
BP	GO:003227	positive re	17/905	137/18866	0.000305	0.001171	0.000615	HSP90AA1/
BP	GO:00018S	embryonic	13/905	89/18866	0.000312	0.001198	0.000629	SOCS3/IL1(
BP	GO:00224(	molting cy	13/905	89/18866	0.000312	0.001198	0.000629	TNF/WNT5
BP	GO:00224(	hair cycle p	13/905	89/18866	0.000312	0.001198	0.000629	TNF/WNT5
BP	GO:00105(	regulation	32/905	347/18866	0.000324	0.00124	0.000652	IFNG/MAP
BP	GO:001921	regulation	14/905	101/18866	0.000325	0.001244	0.000654	IL1B/FABP!
BP	GO:003582	modulatior	15/905	113/18866	0.000326	0.001247	0.000656	CD4/SLPI/S
BP	GO:003032	lung devel	20/905	177/18866	0.000333	0.001273	0.000669	RBP4/TNF/
BP	GO:00355€	non-canon	18/905	151/18866	0.000338	0.001293	0.00068	PSMB8/PSI
BP	GO:000762	circadian rl	23/905	218/18866	0.000339	0.001295	0.000681	RELB/PTGD
BP	GO:00488(	genitalia de	9/905	47/18866	0.00034	0.001296	0.000681	RBP4/WNT
BP	GO:00609ε	endocrine	9/905	47/18866	0.00034	0.001296	0.000681	IL1B/AGT/
BP	GO:009917	regulation	39/905	455/18866	0.000343	0.001307	0.000687	S100B/IL1E
BP	GO:009877	skin epider	13/905	90/18866	0.000349	0.001323	0.000695	TNF/WNT5
BP	GO:00026S	positive re	6/905	21/18866	0.000349	0.001323	0.000695	ICAM1/AG
BP	GO:00108S	positive re	6/905	21/18866	0.000349	0.001323	0.000695	IFNG/IL1B/
BP	GO:003581	regulation	6/905	21/18866	0.000349	0.001323	0.000695	EDNRB/AG
BP	GO:007211	cell prolife	6/905	21/18866	0.000349	0.001323	0.000695	STAT1/PDG
BP	GO:000282	negative re	5/905	14/18866	0.00035	0.001323	0.000695	IFNL1/ARG
BP	GO:001083	positive re	5/905	14/18866	0.00035	0.001323	0.000695	FGF10/ARE
BP	GO:003461	response t	5/905	14/18866	0.00035	0.001323	0.000695	TGFB3/AB(
BP	GO:00357S	platelet-de	5/905	14/18866	0.00035	0.001323	0.000695	SRC/PDGFF
BP	GO:00517(	regulation	5/905	14/18866	0.00035	0.001323	0.000695	IFNG/F2RL
BP	GO:007023	positive re	5/905	14/18866	0.00035	0.001323	0.000695	IDO1/CCL5
BP	GO:00509(	leukocyte t	7/905	29/18866	0.000352	0.00133	0.000699	CXCL12/TN
BP	GO:200072	positive re	7/905	29/18866	0.000352	0.00133	0.000699	TGFB1/GRE
BP	GO:004244	hormone b	11/905	68/18866	0.000364	0.001372	0.000721	DUOX1/DL
BP	GO:000183	epithelial t	18/905	152/18866	0.000367	0.001384	0.000727	TGFB1/IL1I
BP	GO:004592	positive re	8/905	38/18866	0.000368	0.001386	0.000728	IL1B/ADIP(
BP	GO:00713S	cellular res	8/905	38/18866	0.000368	0.001386	0.000728	IL10/EGFR/
BP	GO:190018	regulation	16/905	127/18866	0.000382	0.001436	0.000755	HSP90AB1/
BP	GO:200017	regulation	13/905	91/18866	0.00039	0.001466	0.000771	VEGFA/WN
BP	GO:003163	zymogen a	10/905	58/18866	0.000394	0.00148	0.000778	CTSL/PLAU
BP	GO:00093(	amine met	17/905	140/18866	0.000394	0.00148	0.000778	PSMB8/PSI
BP	GO:00162(	regulation	18/905	153/18866	0.000398	0.001492	0.000784	S100B/TGF
BP	GO:007177	response t	18/905	153/18866	0.000398	0.001492	0.000784	CXCL8/CXC
BP	GO:004222	response t	9/905	48/18866	0.000401	0.001501	0.000789	HSPA5/HSF
BP	GO:200123	regulation	9/905	48/18866	0.000401	0.001501	0.000789	IL1B/TNF/I
BP	GO:005085	regulation	11/905	69/18866	0.000414	0.00155	0.000815	CCR7/CARI
BP	GO:000151	prostaglan	7/905	30/18866	0.000441	0.001643	0.000864	PTGDS/IL1I
BP	GO:000184	protein ins	7/905	30/18866	0.000441	0.001643	0.000864	GZMB/BID/
BP	GO:00350€	positive re	7/905	30/18866	0.000441	0.001643	0.000864	IL1B/MAPK
BP	GO:004645	prostanoid	7/905	30/18866	0.000441	0.001643	0.000864	PTGDS/IL1I

BP	GO:006014	regulation	7/905	30/18866	0.000441	0.001643	0.000864	CXCL10/CX
BP	GO:004327	response to	14/905	104/18866	0.000441	0.001643	0.000864	HSPA5/HSP
BP	GO:003032	respiratory	20/905	181/18866	0.000446	0.001661	0.000873	RBP4/TNF/
BP	GO:004545	cell redox h	10/905	59/18866	0.000454	0.00169	0.000888	PDIA3/PDIA
BP	GO:000257	platelet de	16/905	129/18866	0.000455	0.001694	0.00089	FCER1G/TN
BP	GO:000721	Notch signa	21/905	195/18866	0.000459	0.001707	0.000897	STAT1/SRC
BP	GO:003580	regulation	6/905	22/18866	0.000461	0.00171	0.000899	EDNRB/AD
BP	GO:004562	positive reg	6/905	22/18866	0.000461	0.00171	0.000899	TNFSF4/CD
BP	GO:007135	cellular res	6/905	22/18866	0.000461	0.00171	0.000899	DDX58/IRF
BP	GO:007207	kidney epit	17/905	142/18866	0.000465	0.001725	0.000907	ADIPOQ/ST
BP	GO:001071	regulation	9/905	49/18866	0.00047	0.00174	0.000914	TGFB1/F2R
BP	GO:004205	negative re	9/905	49/18866	0.00047	0.00174	0.000914	AREG/BTC/
BP	GO:006138	trabecula r	9/905	49/18866	0.00047	0.00174	0.000914	RBP4/DLL4
BP	GO:009028	negative re	20/905	182/18866	0.000479	0.00177	0.00093	HSPA5/CXC
BP	GO:001076	positive reg	5/905	15/18866	0.000504	0.001856	0.000975	TGFB1/PTK
BP	GO:003264	regulation	5/905	15/18866	0.000504	0.001856	0.000975	IL1B/PAEP/
BP	GO:004440	adhesion o	5/905	15/18866	0.000504	0.001856	0.000975	HSP90AB1/
BP	GO:004484	estrous cyc	5/905	15/18866	0.000504	0.001856	0.000975	GNRH1/MD
BP	GO:004565	positive reg	5/905	15/18866	0.000504	0.001856	0.000975	TGFB1/IL34
BP	GO:190218	negative re	5/905	15/18866	0.000504	0.001856	0.000975	PML/TRIM2
BP	GO:007200	nephron de	17/905	143/18866	0.000505	0.001858	0.000977	ADIPOQ/ST
BP	GO:003444	substrate a	14/905	106/18866	0.000537	0.001972	0.001036	CALR/ITGA
BP	GO:001066	regulation	13/905	94/18866	0.000537	0.001972	0.001036	IFNG/ARRE
BP	GO:000323	cardiac ver	16/905	131/18866	0.000541	0.001987	0.001044	TGFB1/WN
BP	GO:004863	regulation	18/905	157/18866	0.000544	0.001996	0.001049	S100B/TGF
BP	GO:003436	protein-lipi	7/905	31/18866	0.000546	0.001998	0.00105	CETP/PLA2
BP	GO:003436	plasma lipo	7/905	31/18866	0.000546	0.001998	0.00105	CETP/PLA2
BP	GO:007253	T-helper 17	7/905	31/18866	0.000546	0.001998	0.00105	IL23A/IL12
BP	GO:004361	keratinocyt	9/905	50/18866	0.00055	0.002011	0.001057	VDR/FGF10
BP	GO:003328	cellular res	23/905	226/18866	0.000565	0.002068	0.001087	IL1B/ADIPO
BP	GO:007132	cellular res	17/905	145/18866	0.000593	0.002168	0.00114	ICAM1/NO
BP	GO:003581	renal sodiu	6/905	23/18866	0.000599	0.002182	0.001147	EDNRB/AG
BP	GO:006054	negative re	6/905	23/18866	0.000599	0.002182	0.001147	UCN/FADD
BP	GO:007140	cellular res	6/905	23/18866	0.000599	0.002182	0.001147	FCER1G/PF
BP	GO:190001	positive reg	6/905	23/18866	0.000599	0.002182	0.001147	TNF/IL17D/
BP	GO:200082	regulation	8/905	41/18866	0.000633	0.002306	0.001212	WNT5A/SE
BP	GO:003808	vascular en	9/905	51/18866	0.000639	0.002322	0.00122	VEGFA/PD(
BP	GO:006097	coronary va	9/905	51/18866	0.000639	0.002322	0.00122	VEGFA/PD(
BP	GO:007135	cellular res	9/905	51/18866	0.000639	0.002322	0.00122	ICAM1/STA
BP	GO:007182	plasma lipo	9/905	51/18866	0.000639	0.002322	0.00122	CETP/PLA2
BP	GO:005080	modulation	38/905	454/18866	0.00064	0.002323	0.001221	S100B/IL1B
BP	GO:009010	negative re	16/905	133/18866	0.00064	0.002323	0.001221	HSPA5/TGF
BP	GO:003462	cellular res	17/905	146/18866	0.000642	0.002327	0.001223	CALR/HSPA
BP	GO:004434	cellular res	17/905	146/18866	0.000642	0.002327	0.001223	CXCL8/CXC
BP	GO:000600	glucose me	22/905	214/18866	0.000644	0.002331	0.001225	RBP4/FABP
BP	GO:000317	aortic valve	7/905	32/18866	0.00067	0.00242	0.001272	TGFB1/DLL
BP	GO:003235	regulation	7/905	32/18866	0.00067	0.00242	0.001272	ADM/BMP
BP	GO:003436	protein-cor	7/905	32/18866	0.00067	0.00242	0.001272	CETP/PLA2
BP	GO:004340	negative re	20/905	187/18866	0.000678	0.002446	0.001286	PSMD10/IL
BP	GO:002170	developme	27/905	287/18866	0.000679	0.002446	0.001286	DEFB1/PAE

BP	GO:001061	regulation	11/905	73/18866	0.000679	0.002446	0.001286	PTK2/AGT/
BP	GO:005115	positive re	11/905	73/18866	0.000679	0.002446	0.001286	CXCL9/TGF
BP	GO:001090	regulation	15/905	121/18866	0.000682	0.002454	0.00129	ADIPOQ/SI
BP	GO:000226	lymphocyt	10/905	62/18866	0.000682	0.002454	0.00129	TNFAIP3/M
BP	GO:000740	neuroblast	10/905	62/18866	0.000682	0.002454	0.00129	VEGFA/CX3
BP	GO:000749	mesoderm	16/905	134/18866	0.000696	0.0025	0.001314	VEGFA/WN
BP	GO:000178	neutrophil	5/905	16/18866	0.000704	0.002521	0.001325	PIK3CD/CX
BP	GO:000197	renal syste	5/905	16/18866	0.000704	0.002521	0.001325	F2R/F2RL1
BP	GO:003260	granulocyt	5/905	16/18866	0.000704	0.002521	0.001325	IL1B/PAEP/
BP	GO:003411	positive re	5/905	16/18866	0.000704	0.002521	0.001325	IL1B/TNF/A
BP	GO:004586	negative re	5/905	16/18866	0.000704	0.002521	0.001325	APOBEC3G
BP	GO:006057	morphoger	5/905	16/18866	0.000704	0.002521	0.001325	WNT5A/FG
BP	GO:004368	post-transl	32/905	363/18866	0.000706	0.002524	0.001327	PSMB8/PSI
BP	GO:000693	smooth mu	14/905	109/18866	0.000713	0.002548	0.001339	F2R/PTGS2
BP	GO:007050	calcium ior	12/905	85/18866	0.00072	0.00257	0.001351	CXCL12/TR
BP	GO:190352	regulation	28/905	303/18866	0.000721	0.002575	0.001353	ICAM1/S10
BP	GO:002153	telencepha	25/905	259/18866	0.000729	0.002601	0.001367	CXCL12/WI
BP	GO:000717	transformi	21/905	202/18866	0.000732	0.002608	0.001371	HSPA5/HSI
BP	GO:199008	response t	9/905	52/18866	0.000741	0.002639	0.001387	HSPA5/MA
BP	GO:190406	regulation	31/905	349/18866	0.000749	0.002667	0.001402	HSPA2/IFN
BP	GO:000650	membrane	8/905	42/18866	0.00075	0.002668	0.001402	IFNG/ERAP
BP	GO:000309	glomerular	6/905	24/18866	0.000766	0.002715	0.001427	F2R/F2RL1
BP	GO:001064	regulation	6/905	24/18866	0.000766	0.002715	0.001427	APOD/ADII
BP	GO:001080	negative re	6/905	24/18866	0.000766	0.002715	0.001427	ADIPOQ/F2
BP	GO:001086	regulation	6/905	24/18866	0.000766	0.002715	0.001427	IFNG/ADIP
BP	GO:003469	response t	6/905	24/18866	0.000766	0.002715	0.001427	TNFSF4/CC
BP	GO:006200	secondary	6/905	24/18866	0.000766	0.002715	0.001427	WNT5A/JA
BP	GO:005500	cardiac mu	10/905	63/18866	0.000777	0.00275	0.001445	TGFB1/WN
BP	GO:007005	intrinsic ap	10/905	63/18866	0.000777	0.00275	0.001445	PML/TNFR
BP	GO:000165	ureteric bu	13/905	98/18866	0.000803	0.002839	0.001492	VEGFA/AR
BP	GO:001065	muscle cell	13/905	98/18866	0.000803	0.002839	0.001492	IFNG/ARRE
BP	GO:190437	regulation	15/905	123/18866	0.00081	0.00286	0.001503	IFNG/TGFB
BP	GO:190336	positive re	17/905	149/18866	0.00081	0.00286	0.001503	HSP90AA1/
BP	GO:004801	neurotroph	7/905	33/18866	0.000816	0.002872	0.001509	SRC/AGT/N
BP	GO:006131	cell surface	7/905	33/18866	0.000816	0.002872	0.001509	TGFB1/WN
BP	GO:012025	olefinic cor	7/905	33/18866	0.000816	0.002872	0.001509	ADM/BMP
BP	GO:200075	positive re	7/905	33/18866	0.000816	0.002872	0.001509	IL1B/MAPK
BP	GO:000806	regulation	20/905	190/18866	0.000829	0.002916	0.001532	ICAM1/CX
BP	GO:003296	collagen bi	9/905	53/18866	0.000855	0.003005	0.001579	TGFB1/F2R
BP	GO:004230	molting cyc	14/905	111/18866	0.000856	0.003005	0.001579	TNF/WNT5
BP	GO:004263	hair cycle	14/905	111/18866	0.000856	0.003005	0.001579	TNF/WNT5
BP	GO:000252	hypersensi	4/905	10/18866	0.000875	0.003041	0.001598	FCER1G/CC
BP	GO:000266	regulation	4/905	10/18866	0.000875	0.003041	0.001598	HLA-B/HLA
BP	GO:001088	negative re	4/905	10/18866	0.000875	0.003041	0.001598	PPARG/PP/
BP	GO:003308	regulation	4/905	10/18866	0.000875	0.003041	0.001598	IL1B/IL1A/
BP	GO:003413	regulation	4/905	10/18866	0.000875	0.003041	0.001598	TNFAIP3/F
BP	GO:003650	ATF6-medi	4/905	10/18866	0.000875	0.003041	0.001598	CALR/HSPA
BP	GO:003953	MDA-5 sigr	4/905	10/18866	0.000875	0.003041	0.001598	IRF3/IFIH1/
BP	GO:004254	hemoglobi	4/905	10/18866	0.000875	0.003041	0.001598	EPO/INHA/
BP	GO:004275	drinking be	4/905	10/18866	0.000875	0.003041	0.001598	AGT/APLN/

BP	GO:004298	sequesterir	4/905	10/18866	0.000875	0.003041	0.001598	TMSB10/TI
BP	GO:004338	regulation	4/905	10/18866	0.000875	0.003041	0.001598	IFNL1/TNFS
BP	GO:006051	prostatic b	4/905	10/18866	0.000875	0.003041	0.001598	WNT5A/FG
BP	GO:007038	DNA cytosi	4/905	10/18866	0.000875	0.003041	0.001598	APOBEC3G
BP	GO:007167	smooth mu	4/905	10/18866	0.000875	0.003041	0.001598	PDGFRB/M
BP	GO:190042	positive reg	4/905	10/18866	0.000875	0.003041	0.001598	F2RL1/HAV
BP	GO:200010	regulation	4/905	10/18866	0.000875	0.003041	0.001598	CCL5/IRF7/
BP	GO:200042	regulation	4/905	10/18866	0.000875	0.003041	0.001598	CCL2/C2/C
BP	GO:000195	negative re	8/905	43/18866	0.000883	0.00306	0.001608	MMP12/AI
BP	GO:005085	positive reg	8/905	43/18866	0.000883	0.00306	0.001608	CD4/TNF/C
BP	GO:005085	regulation	8/905	43/18866	0.000883	0.00306	0.001608	CCR7/CARD
BP	GO:007216	mesonephr	13/905	99/18866	0.000885	0.00306	0.001608	VEGFA/AR(
BP	GO:007216	mesonephr	13/905	99/18866	0.000885	0.00306	0.001608	VEGFA/AR(
BP	GO:003083	regulation	20/905	191/18866	0.000885	0.00306	0.001608	ICAM1/CX(
BP	GO:003526	organ grow	20/905	191/18866	0.000885	0.00306	0.001608	RBP4/PDGI
BP	GO:004586	negative re	32/905	369/18866	0.000929	0.00321	0.001687	HSP90AB1/
BP	GO:002195	central ner	20/905	192/18866	0.000944	0.003261	0.001714	B2M/HSP9
BP	GO:000312	heart field	5/905	17/18866	0.000959	0.003291	0.00173	WNT5A/RC
BP	GO:001056	regulation	5/905	17/18866	0.000959	0.003291	0.00173	ADM/BMP
BP	GO:003088	negative re	5/905	17/18866	0.000959	0.003291	0.00173	IL10/BTK/T
BP	GO:004311	positive reg	5/905	17/18866	0.000959	0.003291	0.00173	TGFB1/VEC
BP	GO:005066	hydrogen p	5/905	17/18866	0.000959	0.003291	0.00173	DUOX1/CY
BP	GO:006026	regulation	5/905	17/18866	0.000959	0.003291	0.00173	LBP/RAC2/
BP	GO:190494	midbrain d	5/905	17/18866	0.000959	0.003291	0.00173	WNT5A/DK
BP	GO:200081	negative re	5/905	17/18866	0.000959	0.003291	0.00173	SRC/PTK2/
BP	GO:001474	regulation	11/905	76/18866	0.000959	0.003291	0.00173	PTK2/AGT/
BP	GO:006041	muscle tiss	11/905	76/18866	0.000959	0.003291	0.00173	TGFB1/WN
BP	GO:003952	RIG-I signal	6/905	25/18866	0.000968	0.003312	0.001741	DDX58/NLI
BP	GO:004566	negative re	6/905	25/18866	0.000968	0.003312	0.001741	CXCL14/CX
BP	GO:004663	negative re	6/905	25/18866	0.000968	0.003312	0.001741	SOCS1/TNI
BP	GO:190438	cellular res	6/905	25/18866	0.000968	0.003312	0.001741	SRC/AGT/A
BP	GO:003497	response to	27/905	294/18866	0.000977	0.003341	0.001756	CALR/PDIA
BP	GO:190118	negative re	9/905	54/18866	0.000983	0.003355	0.001763	AREG/BTC/
BP	GO:003085	granulocyt	7/905	34/18866	0.000985	0.003355	0.001763	ADIPOQ/CS
BP	GO:004327	anoikis	7/905	34/18866	0.000985	0.003355	0.001763	SRC/PTK2/
BP	GO:004590	positive reg	7/905	34/18866	0.000985	0.003355	0.001763	ICAM1/F2F
BP	GO:005148	positive reg	7/905	34/18866	0.000985	0.003355	0.001763	F2R/F2RL1/
BP	GO:005509	response to	7/905	34/18866	0.000985	0.003355	0.001763	FCER1G/PF
BP	GO:007010	interleukin	7/905	34/18866	0.000985	0.003355	0.001763	STAT1/SOC
BP	GO:000206	epithelial c	22/905	221/18866	0.000989	0.003365	0.001768	ICAM1/IL1I
BP	GO:004244	hormone m	22/905	221/18866	0.000989	0.003365	0.001768	CTSB/CTSL/
BP	GO:190316	regulation	17/905	152/18866	0.001014	0.003448	0.001812	HSPA2/CX(
BP	GO:003296	regulation	8/905	44/18866	0.001034	0.003514	0.001847	TGFB1/F2R
BP	GO:003238	positive reg	22/905	222/18866	0.00105	0.003564	0.001873	HSP90AB1/
BP	GO:190199	negative re	24/905	251/18866	0.001051	0.003567	0.001875	PSMB8/PSI
BP	GO:006101	regulation	21/905	208/18866	0.001067	0.003614	0.001899	PSMB8/PSI
BP	GO:190290	positive reg	21/905	208/18866	0.001067	0.003614	0.001899	ICAM1/NO
BP	GO:003083	positive reg	13/905	101/18866	0.001069	0.00362	0.001903	ICAM1/TRI
BP	GO:004657	regulation	20/905	194/18866	0.001074	0.003634	0.00191	F2R/F2RL1/
BP	GO:000167	cellular glu	17/905	153/18866	0.001091	0.003691	0.00194	ICAM1/NO

BP	GO:00432(	response t(	14/905	114/18866	0.001115	0.003768	0.00198	ICAM1/TNI
BP	GO:00029	response t(	9/905	55/18866	0.001126	0.003799	0.001997	CX3CR1/PF
BP	GO:00718	protein-lipi	9/905	55/18866	0.001126	0.003799	0.001997	CETP/PLA2
BP	GO:00329	circadian r(	10/905	66/18866	0.001127	0.003799	0.001997	RELB/PML/
BP	GO:00343	protein loc	10/905	66/18866	0.001127	0.003799	0.001997	FCER1G/HS
BP	GO:19030	positive re	15/905	127/18866	0.001129	0.003805	0.002	PSMC2/PSI
BP	GO:00323	regulation	31/905	358/18866	0.001135	0.00382	0.002008	HSP90AB1/
BP	GO:00030	heart proc(	27/905	297/18866	0.001136	0.003823	0.002009	S100A1/NO
BP	GO:00507	regulation	19/905	181/18866	0.001142	0.003842	0.002019	IFNG/IL1B/
BP	GO:00435(	regulation	13/905	102/18866	0.001173	0.003941	0.002071	PTK2/AGT/
BP	GO:00343	smooth mu	7/905	35/18866	0.001181	0.00396	0.002081	IFNG/ARRE
BP	GO:00343	regulation	7/905	35/18866	0.001181	0.00396	0.002081	IFNG/ARRE
BP	GO:00483	mesoderm	7/905	35/18866	0.001181	0.00396	0.002081	DKK1/INHE
BP	GO:00987	bone cell d	7/905	35/18866	0.001181	0.00396	0.002081	LTF/SRC/TI
BP	GO:00158	organic hy(	25/905	268/18866	0.001188	0.003981	0.002092	FCER1G/CX
BP	GO:00610	negative re	11/905	78/18866	0.001194	0.004	0.002102	PLAU/TNF/
BP	GO:00512(	protein ins	8/905	45/18866	0.001206	0.004028	0.002117	HSP90AA1/
BP	GO:19010	regulation	8/905	45/18866	0.001206	0.004028	0.002117	GSK3B/GZI
BP	GO:00067(	C21-steroi(	6/905	26/18866	0.001207	0.004028	0.002117	ADM/BMP
BP	GO:00329	positive re	6/905	26/18866	0.001207	0.004028	0.002117	TGFB1/F2R
BP	GO:00428	defense re	6/905	26/18866	0.001207	0.004028	0.002117	CD40/SLC1
BP	GO:00720	glomerulus	6/905	26/18866	0.001207	0.004028	0.002117	PDGFRB/PI
BP	GO:00073	pattern spe	37/905	455/18866	0.001263	0.004211	0.002213	WNT5A/DL
BP	GO:00328	regulation	10/905	67/18866	0.001269	0.004228	0.002222	IL1B/FABP
BP	GO:00327	positive re	5/905	18/18866	0.001276	0.004237	0.002227	TGFB1/IL1
BP	GO:00360	osteoclast	5/905	18/18866	0.001276	0.004237	0.002227	LTF/SRC/TI
BP	GO:00430	myeloid de	5/905	18/18866	0.001276	0.004237	0.002227	RELB/CSF2/
BP	GO:00900	regulation	5/905	18/18866	0.001276	0.004237	0.002227	CD40/VEGI
BP	GO:00901	positive re	5/905	18/18866	0.001276	0.004237	0.002227	VEGFA/AG
BP	GO:00487(	oligodendr	13/905	103/18866	0.001284	0.004264	0.002241	TLR2/EED/
BP	GO:00507	negative re	9/905	56/18866	0.001285	0.004265	0.002241	SOCS3/SO(
BP	GO:00069	response t(	19/905	183/18866	0.001301	0.004315	0.002268	CALR/HSPA
BP	GO:00512	protein pol	27/905	300/18866	0.001317	0.004357	0.00229	HSP90AA1/
BP	GO:00017	natural kill	4/905	11/18866	0.001324	0.004357	0.00229	IL15/FLT3L
BP	GO:00327	positive re	4/905	11/18866	0.001324	0.004357	0.00229	IL33/CRLF2
BP	GO:00330	immature	4/905	11/18866	0.001324	0.004357	0.00229	IL1B/IL1A/
BP	GO:00338	regulation	4/905	11/18866	0.001324	0.004357	0.00229	AGT/AGTR
BP	GO:00433	memory T	4/905	11/18866	0.001324	0.004357	0.00229	IFNL1/TNF
BP	GO:00510	positive re	4/905	11/18866	0.001324	0.004357	0.00229	RBP4/TNFS
BP	GO:00704	uterine sm	4/905	11/18866	0.001324	0.004357	0.00229	AGT/OXT/
BP	GO:00706	response t(	4/905	11/18866	0.001324	0.004357	0.00229	PDGFB/IL1
BP	GO:00721	glomerular	4/905	11/18866	0.001324	0.004357	0.00229	PDGFRB/PI
BP	GO:00901	positive re	4/905	11/18866	0.001324	0.004357	0.00229	ADIPOQ/PI
BP	GO:00907	immunolog	4/905	11/18866	0.001324	0.004357	0.00229	IFNL1/TNF
BP	GO:00016	metanephr	12/905	91/18866	0.001328	0.004367	0.002295	ADIPOQ/ST
BP	GO:19007	regulation	8/905	46/18866	0.0014	0.004601	0.002418	IL1B/VEGF
BP	GO:00433	positive T	7/905	36/18866	0.001406	0.004617	0.002427	CTSL/IL23A
BP	GO:00716	regulation	7/905	36/18866	0.001406	0.004617	0.002427	HSP90AB1/
BP	GO:19043	positive re	10/905	68/18866	0.001425	0.004676	0.002457	IFNG/TNF/
BP	GO:00181(	peptidyl-th	14/905	117/18866	0.001437	0.004709	0.002475	TGFB1/WN

BP	GO:004544	endothelia	14/905	117/18866	0.001437	0.004709	0.002475	ICAM1/IL1I
BP	GO:004274	hydrogen p	9/905	57/18866	0.001463	0.004783	0.002513	DUOX1/CY
BP	GO:004574	positive re	9/905	57/18866	0.001463	0.004783	0.002513	SRC/DLL4/I
BP	GO:004801	inositol ph	9/905	57/18866	0.001463	0.004783	0.002513	TNF/CHP1/
BP	GO:007139	cellular res	9/905	57/18866	0.001463	0.004783	0.002513	SRC/TNFSF
BP	GO:190018	positive re	11/905	80/18866	0.001475	0.00482	0.002533	HSP90AB1/
BP	GO:000156	response t	6/905	27/18866	0.00149	0.004852	0.00255	CD40/SLC1
BP	GO:001071	positive re	6/905	27/18866	0.00149	0.004852	0.00255	TGFB1/F2R
BP	GO:003469	response t	6/905	27/18866	0.00149	0.004852	0.00255	ICAM1/INH
BP	GO:006054	regulation	6/905	27/18866	0.00149	0.004852	0.00255	ZBP1/FADD
BP	GO:006209	regulation	6/905	27/18866	0.00149	0.004852	0.00255	ZBP1/FADD
BP	GO:190520	negative re	6/905	27/18866	0.00149	0.004852	0.00255	BMP2/DKK
BP	GO:000763	feeding bel	13/905	105/18866	0.001535	0.004991	0.002623	FOS/AGT/A
BP	GO:009700	regulation	13/905	105/18866	0.001535	0.004991	0.002623	CETP/ADIP
BP	GO:001050	positive re	15/905	131/18866	0.001548	0.005032	0.002644	IFNG/MAP
BP	GO:190188	regulation	20/905	200/18866	0.001557	0.005058	0.002658	TLR2/IL1B/
BP	GO:005114	regulation	19/905	186/18866	0.001575	0.005113	0.002687	CXCL10/CX
BP	GO:005170	interaction	12/905	93/18866	0.001606	0.005211	0.002739	LTF/CTSG/
BP	GO:004330	regulation	8/905	47/18866	0.001617	0.005242	0.002755	FCER1G/HL
BP	GO:000193	negative re	11/905	81/18866	0.001635	0.005296	0.002783	TNF/STAT1
BP	GO:000076	syncytium	9/905	58/18866	0.001659	0.005358	0.002816	CXCL10/CX
BP	GO:005105	negative re	9/905	58/18866	0.001659	0.005358	0.002816	BMP2/BMI
BP	GO:014025	cell-cell fus	9/905	58/18866	0.001659	0.005358	0.002816	CXCL10/CX
BP	GO:003267	regulation	5/905	19/18866	0.001663	0.005358	0.002816	IFNL1/IL33
BP	GO:003318	response t	5/905	19/18866	0.001663	0.005358	0.002816	PPARG/EP
BP	GO:003439	positive re	5/905	19/18866	0.001663	0.005358	0.002816	IFNG/ARRE
BP	GO:003953	regulation	5/905	19/18866	0.001663	0.005358	0.002816	NLRX1/DH
BP	GO:200001	positive re	5/905	19/18866	0.001663	0.005358	0.002816	FCER1G/HS
BP	GO:007042	nucleotide-	7/905	37/18866	0.001664	0.005358	0.002816	TNFAIP3/IK
BP	GO:190531	semi-lunar	7/905	37/18866	0.001664	0.005358	0.002816	TGFB1/DLL
BP	GO:000110	response t	15/905	132/18866	0.001671	0.00538	0.002827	ICAM1/TNI
BP	GO:003003	contractile	13/905	106/18866	0.001674	0.005383	0.002829	NOX4/SRC/
BP	GO:004314	stress fiber	13/905	106/18866	0.001674	0.005383	0.002829	NOX4/SRC/
BP	GO:000170	in utero en	32/905	383/18866	0.001703	0.005472	0.002876	PSMC4/SO
BP	GO:003227	regulation	22/905	231/18866	0.001753	0.005629	0.002958	HSP90AA1/
BP	GO:003083	regulation	18/905	174/18866	0.001795	0.00576	0.003027	ICAM1/TM
BP	GO:002261	extracellula	11/905	82/18866	0.001808	0.005795	0.003046	CTSL/MMP
BP	GO:190293	regulation	11/905	82/18866	0.001808	0.005795	0.003046	IFNG/NFYA
BP	GO:000318	aortic valve	6/905	28/18866	0.001821	0.005812	0.003055	TGFB1/DLL
BP	GO:004204	fluid transp	6/905	28/18866	0.001821	0.005812	0.003055	AQP9/EDN
BP	GO:004583	positive re	6/905	28/18866	0.001821	0.005812	0.003055	TGFB1/TFR
BP	GO:006014	positive re	6/905	28/18866	0.001821	0.005812	0.003055	CXCL9/FLT
BP	GO:006143	renal syste	6/905	28/18866	0.001821	0.005812	0.003055	PDGFRB/PI
BP	GO:006144	kidney vaso	6/905	28/18866	0.001821	0.005812	0.003055	PDGFRB/PI
BP	GO:190293	positive re	6/905	28/18866	0.001821	0.005812	0.003055	IFNG/IL1B/
BP	GO:007016	positive re	8/905	48/18866	0.00186	0.005931	0.003117	TGFB1/LTF
BP	GO:011015	positive re	8/905	48/18866	0.00186	0.005931	0.003117	TGFB1/LTF
BP	GO:000292	regulation	4/905	12/18866	0.001911	0.006042	0.003175	LTA/TNF/P
BP	GO:000621	cytidine ca	4/905	12/18866	0.001911	0.006042	0.003175	APOBEC3G
BP	GO:000997	cytidine de	4/905	12/18866	0.001911	0.006042	0.003175	APOBEC3G

BP	GO:00165!	cytidine to	4/905	12/18866	0.001911	0.006042	0.003175	APOBEC3G
BP	GO:00313!	regulation	4/905	12/18866	0.001911	0.006042	0.003175	IL1B/FABP!
BP	GO:00356:	bone mine	4/905	12/18866	0.001911	0.006042	0.003175	LTF/BMP2/
BP	GO:00381{	nerve grow	4/905	12/18866	0.001911	0.006042	0.003175	NGF/NTF3/
BP	GO:00423(	vitamin D k	4/905	12/18866	0.001911	0.006042	0.003175	IFNG/IL1B/
BP	GO:00460{	cytidine m	4/905	12/18866	0.001911	0.006042	0.003175	APOBEC3G
BP	GO:00603!	negative re	4/905	12/18866	0.001911	0.006042	0.003175	DKK1/GREI
BP	GO:00702	regulation	4/905	12/18866	0.001911	0.006042	0.003175	WNT5A/HI
BP	GO:00716:	positive re	4/905	12/18866	0.001911	0.006042	0.003175	IL1B/ADIPC
BP	GO:19059	positive re	4/905	12/18866	0.001911	0.006042	0.003175	SRC/SEMA:
BP	GO:20012(	regulation	4/905	12/18866	0.001911	0.006042	0.003175	LTF/TNFSF:
BP	GO:00075{	body fluid	12/905	95/18866	0.001932	0.006102	0.003207	VEGFA/VDI
BP	GO:00507(	negative re	12/905	95/18866	0.001932	0.006102	0.003207	IL1B/F2R/F
BP	GO:00713:	cellular res	15/905	134/18866	0.001942	0.00613	0.003222	ICAM1/NO
BP	GO:00023{	mucosal im	7/905	38/18866	0.001956	0.00615	0.003232	XCL1/DEFB
BP	GO:00106(	positive re	7/905	38/18866	0.001956	0.00615	0.003232	IFNG/ARRE
BP	GO:00358	nucleotide-	7/905	38/18866	0.001956	0.00615	0.003232	TNFAIP3/IK
BP	GO:00480(	insulin-like	7/905	38/18866	0.001956	0.00615	0.003232	PIK3R1/BM
BP	GO:00519:	regulation	7/905	38/18866	0.001956	0.00615	0.003232	FABP5/F2R
BP	GO:00712	cellular res	7/905	38/18866	0.001956	0.00615	0.003232	MMP9/CYE
BP	GO:00716(	transformi	7/905	38/18866	0.001956	0.00615	0.003232	HSP90AB1/
BP	GO:00605	respiratory	20/905	204/18866	0.001973	0.006201	0.003259	RBP4/TNF/
BP	GO:00486	muscle org	11/905	83/18866	0.001996	0.006267	0.003294	TGFB1/WN
BP	GO:00485(	digestive tr	15/905	135/18866	0.00209	0.00656	0.003448	CXCL8/TNF
BP	GO:00069	syncytium	9/905	60/18866	0.002115	0.006628	0.003483	CXCL10/CX
BP	GO:20002	negative re	9/905	60/18866	0.002115	0.006628	0.003483	CALR/PAEF
BP	GO:00026{	negative re	5/905	20/18866	0.002131	0.006628	0.003483	CCL2/MIF/
BP	GO:00100(	cardioblast	5/905	20/18866	0.002131	0.006628	0.003483	GREM1/NF
BP	GO:00326:	interleukin	5/905	20/18866	0.002131	0.006628	0.003483	IFNL1/IL33
BP	GO:00439!	positive re	5/905	20/18866	0.002131	0.006628	0.003483	NOS1/UCN
BP	GO:00605	negative re	5/905	20/18866	0.002131	0.006628	0.003483	FADD/CASI
BP	GO:00620!	negative re	5/905	20/18866	0.002131	0.006628	0.003483	FADD/CASI
BP	GO:00977(	connective	5/905	20/18866	0.002131	0.006628	0.003483	TGFB1/F2R
BP	GO:20007	positive re	5/905	20/18866	0.002131	0.006628	0.003483	B2M/HLA-(
BP	GO:00020(	glandular e	8/905	49/18866	0.002131	0.006628	0.003483	WNT5A/GS
BP	GO:00024	mast cell m	8/905	49/18866	0.002131	0.006628	0.003483	FCER1G/BT
BP	GO:00066!	prostanoid	8/905	49/18866	0.002131	0.006628	0.003483	PTGDS/IL1I
BP	GO:00066!	prostaglan	8/905	49/18866	0.002131	0.006628	0.003483	PTGDS/IL1I
BP	GO:00901!	establishm	8/905	49/18866	0.002131	0.006628	0.003483	HSP90AA1/
BP	GO:19900!	cellular res	8/905	49/18866	0.002131	0.006628	0.003483	HSPA5/MA
BP	GO:00148!	muscle hyp	13/905	109/18866	0.002157	0.006704	0.003523	PTK2/AGT/
BP	GO:19029(	regulation	31/905	373/18866	0.002164	0.006721	0.003532	ICAM1/CX(
BP	GO:00615	myeloid ce	10/905	72/18866	0.002212	0.006868	0.003609	TLR2/LTF/S
BP	GO:00713:	cellular res	15/905	136/18866	0.002248	0.006974	0.003665	ICAM1/NO
BP	GO:00081!	actin polyn	21/905	221/18866	0.002265	0.007018	0.003688	ICAM1/CX(
BP	GO:00316(	cellular res	21/905	221/18866	0.002265	0.007018	0.003688	HSPA5/ICA
BP	GO:00108:	regulation	7/905	39/18866	0.002287	0.007067	0.003714	VDR/FGF1(
BP	GO:00381	neurotropl	7/905	39/18866	0.002287	0.007067	0.003714	SRC/AGT/N
BP	GO:00519:	regulation	7/905	39/18866	0.002287	0.007067	0.003714	FABP5/F2R
BP	GO:19047(	negative re	7/905	39/18866	0.002287	0.007067	0.003714	ADIPOQ/IL

BP	GO:200024	regulation	7/905	39/18866	0.002287	0.007067	0.003714	PTK2B/HCK
BP	GO:000660	protein im	16/905	150/18866	0.002304	0.007117	0.00374	HSP90AB1/
BP	GO:190134	negative re	20/905	207/18866	0.002344	0.007235	0.003802	HLA-G/CXC
BP	GO:000317	heart valve	9/905	61/18866	0.002378	0.007314	0.003844	TGFB1/DLL
BP	GO:002187	forebrain g	9/905	61/18866	0.002378	0.007314	0.003844	B2M/WNT
BP	GO:003361	membrane	9/905	61/18866	0.002378	0.007314	0.003844	IFNG/ERAP
BP	GO:004584	negative re	9/905	61/18866	0.002378	0.007314	0.003844	S100B/TGF
BP	GO:009034	regulation	9/905	61/18866	0.002378	0.007314	0.003844	B2M/HLA-
BP	GO:190307	positive re	9/905	61/18866	0.002378	0.007314	0.003844	IFNG/TNF/
BP	GO:003004	actin filam	19/905	193/18866	0.002409	0.007407	0.003892	ICAM1/TM
BP	GO:007132	cellular res	15/905	137/18866	0.002415	0.007421	0.0039	ICAM1/NO
BP	GO:004598	positive re	8/905	50/18866	0.002433	0.007458	0.003919	IFNG/TMS
BP	GO:010102	vascular er	8/905	50/18866	0.002433	0.007458	0.003919	CCL2/PPAR
BP	GO:190054	positive re	8/905	50/18866	0.002433	0.007458	0.003919	IFNG/TMS
BP	GO:190556	regulation	8/905	50/18866	0.002433	0.007458	0.003919	CCL2/PPAR
BP	GO:004846	cell matura	18/905	179/18866	0.002455	0.007516	0.00395	DEFB1/PAE
BP	GO:000320	cardiac ver	10/905	73/18866	0.002456	0.007516	0.00395	TGFB1/DLL
BP	GO:190374	regulation	10/905	73/18866	0.002456	0.007516	0.00395	MAPT/RAC
BP	GO:001633	morphoger	16/905	151/18866	0.002466	0.007543	0.003964	TGFB1/WN
BP	GO:004583	negative re	12/905	98/18866	0.002519	0.007702	0.004048	IL1B/APOD
BP	GO:000726	Ras protein	29/905	346/18866	0.002614	0.007963	0.004185	F2R/F2RL1
BP	GO:003596	cellular res	17/905	166/18866	0.002639	0.007963	0.004185	CALR/HSPA
BP	GO:000340	axis elonga	6/905	30/18866	0.002644	0.007963	0.004185	WNT5A/FG
BP	GO:001080	positive re	6/905	30/18866	0.002644	0.007963	0.004185	TGFB1/WN
BP	GO:003411	regulation	6/905	30/18866	0.002644	0.007963	0.004185	CCL5/TNFS
BP	GO:003811	interleukin	6/905	30/18866	0.002644	0.007963	0.004185	SOCS1/IL7
BP	GO:006005	angiogenes	6/905	30/18866	0.002644	0.007963	0.004185	TNF/VEGF
BP	GO:007016	negative re	6/905	30/18866	0.002644	0.007963	0.004185	TGFB1/CCL
BP	GO:007072	response t	6/905	30/18866	0.002644	0.007963	0.004185	TGFB1/CCL
BP	GO:011015	negative re	6/905	30/18866	0.002644	0.007963	0.004185	TGFB1/CCL
BP	GO:000243	compleme	4/905	13/18866	0.002656	0.007963	0.004185	C5AR1/CM
BP	GO:000283	positive re	4/905	13/18866	0.002656	0.007963	0.004185	MR1/IL12A
BP	GO:000283	positive re	4/905	13/18866	0.002656	0.007963	0.004185	MR1/IL12A
BP	GO:000717	activation	4/905	13/18866	0.002656	0.007963	0.004185	EGF/GREM
BP	GO:000822	opsonizatio	4/905	13/18866	0.002656	0.007963	0.004185	LBP/PTX3/
BP	GO:001062	programm	4/905	13/18866	0.002656	0.007963	0.004185	IL1B/IL1A/
BP	GO:001626	gap junctio	4/905	13/18866	0.002656	0.007963	0.004185	IL1B/AGT/
BP	GO:003235	positive re	4/905	13/18866	0.002656	0.007963	0.004185	ADM/PPAF
BP	GO:003249	response t	4/905	13/18866	0.002656	0.007963	0.004185	TLR2/DMB
BP	GO:003312	regulation	4/905	13/18866	0.002656	0.007963	0.004185	IL1B/MAPK
BP	GO:003360	positive re	4/905	13/18866	0.002656	0.007963	0.004185	CXCL12/GD
BP	GO:004230	negative re	4/905	13/18866	0.002656	0.007963	0.004185	APOD/CHP
BP	GO:005509	phospholip	4/905	13/18866	0.002656	0.007963	0.004185	FABP3/CET
BP	GO:006060	lateral spro	4/905	13/18866	0.002656	0.007963	0.004185	WNT5A/FG
BP	GO:006104	regulation	4/905	13/18866	0.002656	0.007963	0.004185	TNF/TNFAI
BP	GO:190027	negative re	4/905	13/18866	0.002656	0.007963	0.004185	AGER/CX3
BP	GO:190256	regulation	4/905	13/18866	0.002656	0.007963	0.004185	PLA2G2A/I
BP	GO:190323	regulation	4/905	13/18866	0.002656	0.007963	0.004185	CXCL12/CC
BP	GO:190397	positive re	4/905	13/18866	0.002656	0.007963	0.004185	CCL3/CX3C
BP	GO:190459	negative re	4/905	13/18866	0.002656	0.007963	0.004185	APOD/CHP

BP	GO:200119	regulation	4/905	13/18866	0.002656	0.007963	0.004185	HLA-B/HLA
BP	GO:001061	positive re	7/905	40/18866	0.002659	0.007963	0.004185	PTK2/AGT/
BP	GO:001093	regulation	7/905	40/18866	0.002659	0.007963	0.004185	UCN/ZBP1/
BP	GO:001657	histone ph	7/905	40/18866	0.002659	0.007963	0.004185	IL1B/MAPK
BP	GO:003050	positive re	7/905	40/18866	0.002659	0.007963	0.004185	TGFB1/LTF
BP	GO:004230	positive re	7/905	40/18866	0.002659	0.007963	0.004185	HSP90AB1/
BP	GO:007221	metanephr	7/905	40/18866	0.002659	0.007963	0.004185	ADIPOQ/ST
BP	GO:001067	positive re	9/905	62/18866	0.002665	0.007964	0.004185	SRC/IGF1/
BP	GO:004863	negative re	9/905	62/18866	0.002665	0.007964	0.004185	S100B/TGF
BP	GO:005502	positive re	9/905	62/18866	0.002665	0.007964	0.004185	TGFB1/GRE
BP	GO:200124	positive re	9/905	62/18866	0.002665	0.007964	0.004185	S100A9/S1
BP	GO:000202	desensitiza	5/905	21/18866	0.002688	0.007978	0.004193	ARRB1/AD
BP	GO:001482	phasic smo	5/905	21/18866	0.002688	0.007978	0.004193	EDN3/EDN
BP	GO:001655	base conve	5/905	21/18866	0.002688	0.007978	0.004193	APOBEC3G
BP	GO:002240	negative ad	5/905	21/18866	0.002688	0.007978	0.004193	ARRB1/AD
BP	GO:003050	negative re	5/905	21/18866	0.002688	0.007978	0.004193	CCL3/CCR1
BP	GO:003363	positive re	5/905	21/18866	0.002688	0.007978	0.004193	CXCL13/CC
BP	GO:003649	PERK-medi	5/905	21/18866	0.002688	0.007978	0.004193	HSPA5/CXC
BP	GO:004337	negative re	5/905	21/18866	0.002688	0.007978	0.004193	TNFSF4/JA
BP	GO:006021	definitive h	5/905	21/18866	0.002688	0.007978	0.004193	ADAR/TEK/
BP	GO:006104	vascular w	5/905	21/18866	0.002688	0.007978	0.004193	TNF/VEGF/
BP	GO:006121	positive re	5/905	21/18866	0.002688	0.007978	0.004193	VEGFA/AG
BP	GO:014046	integrated	5/905	21/18866	0.002688	0.007978	0.004193	HSPA5/CXC
BP	GO:001071	regulation	12/905	99/18866	0.002745	0.008138	0.004277	TGFB1/IL1
BP	GO:005181	modulation	12/905	99/18866	0.002745	0.008138	0.004277	CD4/LTF/C
BP	GO:000698	ER-nucleus	8/905	51/18866	0.002766	0.008197	0.004308	CALR/HSPA
BP	GO:190198	negative re	24/905	270/18866	0.002792	0.008269	0.004346	PSMB8/PS
BP	GO:005080	regulation	22/905	240/18866	0.002822	0.008355	0.004391	TLR2/MX1/
BP	GO:007189	DNA biosyn	19/905	196/18866	0.002866	0.008479	0.004456	HSP90AA1/
BP	GO:001821	peptidyl-th	14/905	126/18866	0.002898	0.00857	0.004504	TGFB1/WN
BP	GO:000225	organ or tis	7/905	41/18866	0.003076	0.009081	0.004773	XCL1/DEFB
BP	GO:001474	positive re	7/905	41/18866	0.003076	0.009081	0.004773	PTK2/AGT/
BP	GO:200000	regulation	7/905	41/18866	0.003076	0.009081	0.004773	FCER1G/HS
BP	GO:000317	heart valve	8/905	52/18866	0.003135	0.009238	0.004855	TGFB1/DLL
BP	GO:003051	negative re	8/905	52/18866	0.003135	0.009238	0.004855	HJV/WNT5
BP	GO:003806	p38MAPK c	8/905	52/18866	0.003135	0.009238	0.004855	IL1B/VEGF
BP	GO:005085	negative re	6/905	31/18866	0.003148	0.009258	0.004865	CD22/PTPN
BP	GO:006103	negative re	6/905	31/18866	0.003148	0.009258	0.004865	GDF5/GRE
BP	GO:190074	positive re	6/905	31/18866	0.003148	0.009258	0.004865	IL1B/VEGF
BP	GO:190223	negative re	6/905	31/18866	0.003148	0.009258	0.004865	CXCL12/TP
BP	GO:006102	membrane	17/905	169/18866	0.003182	0.009352	0.004915	CALR/CD4/
BP	GO:004688	positive re	11/905	88/18866	0.003188	0.009363	0.004921	IFNG/IL1B/
BP	GO:000188	liver develo	15/905	141/18866	0.003191	0.009365	0.004922	TNFAIP3/IL
BP	GO:003001	establishm	15/905	141/18866	0.003191	0.009365	0.004922	HSP90AA1/
BP	GO:000279	negative re	12/905	101/18866	0.003245	0.009517	0.005001	IL1B/F2R/R
BP	GO:000328	ventricular	10/905	76/18866	0.003317	0.009712	0.005104	WNT5A/LT
BP	GO:007122	cellular res	10/905	76/18866	0.003317	0.009712	0.005104	TNF/CYBB/
BP	GO:007209	regulation	10/905	76/18866	0.003317	0.009712	0.005104	VEGFA/EIF
BP	GO:004668	response to	9/905	64/18866	0.003323	0.009719	0.005108	B2M/MMP
BP	GO:190186	negative re	9/905	64/18866	0.003323	0.009719	0.005108	S100B/TGF

BP	GO:00230!	adaptation	5/905	22/18866	0.003343	0.009719	0.005108	ARRB1/ADI
BP	GO:003206	regulation	5/905	22/18866	0.003343	0.009719	0.005108	OASL/OAS1
BP	GO:00327(	negative re	5/905	22/18866	0.003343	0.009719	0.005108	IFNG/TNFS
BP	GO:00395:	regulation	5/905	22/18866	0.003343	0.009719	0.005108	NLRX1/DH)
BP	GO:00507€	negative re	5/905	22/18866	0.003343	0.009719	0.005108	TLR2/ADIP(
BP	GO:00510(	positive re{	5/905	22/18866	0.003343	0.009719	0.005108	S100A1/TN
BP	GO:00550!	response t(	5/905	22/18866	0.003343	0.009719	0.005108	PDGFRB/PI
BP	GO:00713;	cellular res	5/905	22/18866	0.003343	0.009719	0.005108	TNFSF4/PP
BP	GO:00901{	regulation	5/905	22/18866	0.003343	0.009719	0.005108	VEGFA/AG'
BP	GO:19017:	regulation	5/905	22/18866	0.003343	0.009719	0.005108	CXCL10/CX
BP	GO:20003	regulation	5/905	22/18866	0.003343	0.009719	0.005108	IL23A/IL12
BP	GO:00313:	negative re	15/905	142/18866	0.003415	0.009923	0.005215	HSPA5/TM
BP	GO:19030;	regulation	12/905	102/18866	0.003521	0.010222	0.005372	IFNG/TGFB
BP	GO:20000€	positive re{	12/905	102/18866	0.003521	0.010222	0.005372	PSMD10/P'
BP	GO:00456€	regulation	8/905	53/18866	0.00354	0.010253	0.005389	CXCL14/CX
BP	GO:00457⁄	negative re	8/905	53/18866	0.00354	0.010253	0.005389	CXCL8/CCL
BP	GO:20007;	regulation	8/905	53/18866	0.00354	0.010253	0.005389	B2M/HLA-(
BP	GO:00109(	positive re{	7/905	42/18866	0.003542	0.010253	0.005389	SRC/IGF1/E
BP	GO:19045!	positive re{	7/905	42/18866	0.003542	0.010253	0.005389	HSP90AB1/
BP	GO:00033(	Wnt signali	4/905	14/18866	0.003578	0.010287	0.005406	WNT5A/BN
BP	GO:00302	hyaluronar	4/905	14/18866	0.003578	0.010287	0.005406	TGFB1/IL1E
BP	GO:00351	genitalia m	4/905	14/18866	0.003578	0.010287	0.005406	RBP4/FGF1
BP	GO:00358	positive re{	4/905	14/18866	0.003578	0.010287	0.005406	EDNRB/AG
BP	GO:00426!	regulation	4/905	14/18866	0.003578	0.010287	0.005406	WNT5A/DK
BP	GO:00439;	negative re	4/905	14/18866	0.003578	0.010287	0.005406	CCL5/CCL4,
BP	GO:00450(	DNA deam	4/905	14/18866	0.003578	0.010287	0.005406	APOBEC3G
BP	GO:00455!	positive re{	4/905	14/18866	0.003578	0.010287	0.005406	HLA-G/IFN(
BP	GO:00461:	pyrimidine	4/905	14/18866	0.003578	0.010287	0.005406	APOBEC3G
BP	GO:00461:	pyrimidine	4/905	14/18866	0.003578	0.010287	0.005406	APOBEC3G
BP	GO:00482!	isotype swi	4/905	14/18866	0.003578	0.010287	0.005406	CD40/IL27I
BP	GO:00907	immunolo{	4/905	14/18866	0.003578	0.010287	0.005406	IFNL1/TNF!
BP	GO:20012;	regulation	4/905	14/18866	0.003578	0.010287	0.005406	IL1B/FABP!
BP	GO:00082{	insulin rec€	15/905	143/18866	0.00365	0.01049	0.005513	IL1B/SOCS:
BP	GO:00075{	excretion	9/905	65/18866	0.003697	0.010613	0.005577	AQP9/EDN
BP	GO:00512(	protein ins	9/905	65/18866	0.003697	0.010613	0.005577	HSP90AA1,
BP	GO:00357€	endothelia	6/905	32/18866	0.00372	0.010667	0.005606	CXCL13/TN
BP	GO:00705;	protein kin	6/905	32/18866	0.00372	0.010667	0.005606	CD40/VEGI
BP	GO:00510!	regulation	27/905	323/18866	0.003734	0.0107	0.005623	F2R/F2RL1,
BP	GO:00305	intracellula	13/905	116/18866	0.003738	0.010708	0.005627	CALR/SRC/
BP	GO:00508(	positive re{	17/905	172/18866	0.003814	0.010918	0.005738	S100B/TNF
BP	GO:00345(	protein loc	24/905	277/18866	0.00387	0.011074	0.00582	CALR/HSP9
BP	GO:00610(	hepaticobil	15/905	144/18866	0.003899	0.011152	0.005861	TNFAIP3/IL
BP	GO:00308!	negative re	8/905	54/18866	0.003984	0.011389	0.005985	IFNG/MMF
BP	GO:00104;	regulation	7/905	43/18866	0.004059	0.011583	0.006087	ADIPOQ/IL
BP	GO:00400	positive re{	7/905	43/18866	0.004059	0.011583	0.006087	GDNF/IGF1
BP	GO:00457;	positive re{	7/905	43/18866	0.004059	0.011583	0.006087	CXCL12/VE
BP	GO:00459:	negative re	28/905	341/18866	0.004082	0.011642	0.006118	PSMB8/PSI
BP	GO:00456€	positive re{	9/905	66/18866	0.004103	0.011648	0.006121	LTF/IFITM1
BP	GO:00466;	regulation	9/905	66/18866	0.004103	0.011648	0.006121	IL1B/SOCS:
BP	GO:00468;	positive re{	9/905	66/18866	0.004103	0.011648	0.006121	HSP90AB1,

BP	GO:00327(	negative re	5/905	23/18866	0.004105	0.011648	0.006121	XCL1/TNFA
BP	GO:003545	cellular res	5/905	23/18866	0.004105	0.011648	0.006121	TLR3/STAT
BP	GO:004202	protein ref	5/905	23/18866	0.004105	0.011648	0.006121	B2M/HSPA
BP	GO:00461	pyrimidine	5/905	23/18866	0.004105	0.011648	0.006121	APOBEC3G
BP	GO:00510	regulation	5/905	23/18866	0.004105	0.011648	0.006121	IFNG/IL1B/
BP	GO:00512	cytoplasmi	5/905	23/18866	0.004105	0.011648	0.006121	PSMD10/T
BP	GO:00903	positive re	5/905	23/18866	0.004105	0.011648	0.006121	B2M/HLA-(
BP	GO:00033(	cardiac mu	12/905	104/18866	0.004131	0.011714	0.006156	PTK2/AGT/
BP	GO:00066	phospholip	35/905	455/18866	0.004161	0.011795	0.006199	TGFB1/FAE
BP	GO:00157	monocarbo	17/905	174/18866	0.00429	0.012152	0.006386	IL1B/FABP!
BP	GO:00219	olfactory l	6/905	33/18866	0.004365	0.012354	0.006492	WNT5A/SE
BP	GO:00350	positive re	6/905	33/18866	0.004365	0.012354	0.006492	F2R/F2RL1
BP	GO:00073	gastrulatio	18/905	189/18866	0.004394	0.012414	0.006524	MMP9/AD
BP	GO:00108	regulation	10/905	79/18866	0.004403	0.012414	0.006524	IL1B/FABP!
BP	GO:00310	pancreas d	10/905	79/18866	0.004403	0.012414	0.006524	WNT5A/GS
BP	GO:00422	tissue rege	10/905	79/18866	0.004403	0.012414	0.006524	APOD/FGF
BP	GO:00434(	negative re	10/905	79/18866	0.004403	0.012414	0.006524	IL1B/ADIP(
BP	GO:00703	negative re	10/905	79/18866	0.004403	0.012414	0.006524	ADIPOQ/AI
BP	GO:00903	cellular ser	10/905	79/18866	0.004403	0.012414	0.006524	B2M/CALR
BP	GO:00424	odontogen	14/905	132/18866	0.004426	0.012474	0.006556	SRC/TNFSF
BP	GO:00603	bone devel	19/905	204/18866	0.00445	0.012534	0.006587	LTF/SRC/TI
BP	GO:00350	regulation	8/905	55/18866	0.004471	0.012574	0.006608	IL1B/MAPK
BP	GO:00508	negative re	8/905	55/18866	0.004471	0.012574	0.006608	PROCR/PL
BP	GO:00973	mitochond	8/905	55/18866	0.004471	0.012574	0.006608	GSK3B/GZI
BP	GO:00310	positive re	11/905	92/18866	0.004505	0.012649	0.006647	IL1B/VEGF
BP	GO:19004(	regulation	11/905	92/18866	0.004505	0.012649	0.006647	TNF/IL10/N
BP	GO:19033	negative re	11/905	92/18866	0.004505	0.012649	0.006647	ISG15/TNF
BP	GO:00060	aminoglyca	9/905	67/18866	0.004543	0.012749	0.0067	PGLYRP1/P
BP	GO:00096	response t	22/905	250/18866	0.004609	0.012929	0.006795	LCN2/S100
BP	GO:00104	mesenchyr	7/905	44/18866	0.004631	0.012969	0.006816	STAT1/VEG
BP	GO:00330(	regulation	7/905	44/18866	0.004631	0.012969	0.006816	FCER1G/PL
BP	GO:19019	positive re	7/905	44/18866	0.004631	0.012969	0.006816	IL1B/MAPK
BP	GO:00359	response t	19/905	205/18866	0.004691	0.013045	0.006856	CALR/HSPA
BP	GO:00024	germinal c	4/905	15/18866	0.004696	0.013045	0.006856	CXCL13/TN
BP	GO:00028	regulation	4/905	15/18866	0.004696	0.013045	0.006856	FCER1G/C(
BP	GO:00200	hemoglobi	4/905	15/18866	0.004696	0.013045	0.006856	EPO/INHA/
BP	GO:00302	male sex d	4/905	15/18866	0.004696	0.013045	0.006856	PTGDR/GN
BP	GO:00357	T-helper 2	4/905	15/18866	0.004696	0.013045	0.006856	XCL1/RSAD
BP	GO:00423	fat-soluble	4/905	15/18866	0.004696	0.013045	0.006856	IFNG/IL1B/
BP	GO:00433	CD8-positi	4/905	15/18866	0.004696	0.013045	0.006856	SOCS1/IRF
BP	GO:00510	positive re	4/905	15/18866	0.004696	0.013045	0.006856	IFNG/IL1B/
BP	GO:00603	negative re	4/905	15/18866	0.004696	0.013045	0.006856	MMP12/AI
BP	GO:00718(	regulation	4/905	15/18866	0.004696	0.013045	0.006856	TNF/SRC/H
BP	GO:00901	regulation	4/905	15/18866	0.004696	0.013045	0.006856	ADIPOQ/PI
BP	GO:19015	regulation	4/905	15/18866	0.004696	0.013045	0.006856	IL1B/TNF/\
BP	GO:19031	regulation	4/905	15/18866	0.004696	0.013045	0.006856	IL1B/TNF/\
BP	GO:20010	positive re	4/905	15/18866	0.004696	0.013045	0.006856	TMSB4X/V
BP	GO:00071	establishm	20/905	220/18866	0.004697	0.013045	0.006856	HSP90AA1
BP	GO:00551	digestive s	15/905	147/18866	0.004731	0.013133	0.006902	CXCL8/TNF
BP	GO:00313	negative re	10/905	80/18866	0.004821	0.013367	0.007025	ISG15/TNF

BP	GO:190402	regulation	12/905	106/18866	0.004822	0.013367	0.007025	HSP90AB1/
BP	GO:190526	positive reg	12/905	106/18866	0.004822	0.013367	0.007025	IL1B/VEGFA
BP	GO:003051	regulation	11/905	93/18866	0.004893	0.013557	0.007125	HJV/WNT5
BP	GO:007124	cellular res	20/905	221/18866	0.004939	0.013675	0.007187	B2M/CALR
BP	GO:000693	muscle cor	29/905	362/18866	0.004962	0.013734	0.007218	F2R/DES/P
BP	GO:001052	regulation	5/905	24/18866	0.004984	0.013737	0.007219	APOBEC3G
BP	GO:001052	negative re	5/905	24/18866	0.004984	0.013737	0.007219	APOBEC3G
BP	GO:003233	negative re	5/905	24/18866	0.004984	0.013737	0.007219	GDF5/GREI
BP	GO:003414	regulation	5/905	24/18866	0.004984	0.013737	0.007219	LBP/LTF/BI
BP	GO:004595	positive reg	5/905	24/18866	0.004984	0.013737	0.007219	HLA-F/RAE
BP	GO:007084	inclusion b	5/905	24/18866	0.004984	0.013737	0.007219	HSPA2/MA
BP	GO:190342	regulation	5/905	24/18866	0.004984	0.013737	0.007219	PAEP/RAC3
BP	GO:200020	regulation	5/905	24/18866	0.004984	0.013737	0.007219	SRC/PTK2/
BP	GO:001081	negative re	9/905	68/18866	0.005018	0.013826	0.007266	MMP12/AI
BP	GO:003164	regulation	15/905	148/18866	0.005038	0.013865	0.007286	S100B/FAB
BP	GO:007190	negative re	15/905	148/18866	0.005038	0.013865	0.007286	IL1B/ADIPC
BP	GO:004599	regulation	14/905	134/18866	0.005061	0.013921	0.007316	ADIPOQ/W
BP	GO:001907	viral releas	6/905	34/18866	0.00509	0.013958	0.007336	PML/PPIA/
BP	GO:003043	sleep	6/905	34/18866	0.00509	0.013958	0.007336	PTGDS/PTC
BP	GO:003589	exit from h	6/905	34/18866	0.00509	0.013958	0.007336	PML/PPIA/
BP	GO:003589	exit from h	6/905	34/18866	0.00509	0.013958	0.007336	PML/PPIA/
BP	GO:190269	regulation	6/905	34/18866	0.00509	0.013958	0.007336	VEGFA/CX3
BP	GO:200035	negative re	6/905	34/18866	0.00509	0.013958	0.007336	ICAM1/TNI
BP	GO:001652	negative re	18/905	192/18866	0.005176	0.014188	0.007456	HLA-G/CXC
BP	GO:001489	striated mu	12/905	107/18866	0.005201	0.014241	0.007484	PTK2/AGT/
BP	GO:005501	cardiac mu	12/905	107/18866	0.005201	0.014241	0.007484	RBP4/AGT/
BP	GO:000752	myoblast fu	7/905	45/18866	0.005262	0.014357	0.007545	CXCL10/CX
BP	GO:003101	endocrine p	7/905	45/18866	0.005262	0.014357	0.007545	WNT5A/GS
BP	GO:003292	activin rece	7/905	45/18866	0.005262	0.014357	0.007545	HJV/FGF10
BP	GO:004277	intrinsic ap	7/905	45/18866	0.005262	0.014357	0.007545	PML/MIF/I
BP	GO:004591	positive reg	7/905	45/18866	0.005262	0.014357	0.007545	TGFB1/TFR
BP	GO:005502	negative re	7/905	45/18866	0.005262	0.014357	0.007545	RBP4/BMP
BP	GO:006041	ventricular	7/905	45/18866	0.005262	0.014357	0.007545	WNT5A/RC
BP	GO:006099	kidney mor	11/905	94/18866	0.005308	0.014477	0.007608	STAT1/VEG
BP	GO:000761	memory	13/905	121/18866	0.005361	0.014612	0.007679	S100B/MA
BP	GO:007124	cellular res	18/905	193/18866	0.005461	0.014877	0.007819	B2M/CALR
BP	GO:007123	cellular res	9/905	69/18866	0.005532	0.015056	0.007912	TNF/CYBB/
BP	GO:190331	regulation	9/905	69/18866	0.005532	0.015056	0.007912	TFR2/SRC/
BP	GO:001931	hexose me	22/905	254/18866	0.005549	0.015095	0.007933	RBP4/FABF
BP	GO:000931	response to	34/905	447/18866	0.005562	0.015124	0.007948	HSPA5/ICA
BP	GO:004685	phosphatic	8/905	57/18866	0.00558	0.015164	0.007969	SOCS3/SOC
BP	GO:004275	regulation	13/905	122/18866	0.005744	0.015602	0.0082	PTGDS/PM
BP	GO:200018	negative re	18/905	194/18866	0.005758	0.015632	0.008215	HLA-G/CXC
BP	GO:001648	peptide ho	6/905	35/18866	0.005899	0.015966	0.00839	CTSL/CTSG
BP	GO:003969	viral RNA g	6/905	35/18866	0.005899	0.015966	0.00839	CXCL8/APC
BP	GO:009700	energy hor	6/905	35/18866	0.005899	0.015966	0.00839	PIK3CA/BN
BP	GO:011011	negative re	6/905	35/18866	0.005899	0.015966	0.00839	TNF/STAT1
BP	GO:014044	signaling re	6/905	35/18866	0.005899	0.015966	0.00839	CTSL/CTSG
BP	GO:190001	negative re	6/905	35/18866	0.005899	0.015966	0.00839	APOD/NLR
BP	GO:001087	regulation	7/905	46/18866	0.005955	0.016102	0.008462	CETP/ADIP

BP	GO:00485	thymus de	7/905	46/18866	0.005955	0.016102	0.008462	MAPK3/FG
BP	GO:00075	sex determ	5/905	25/18866	0.005988	0.016118	0.008471	PTGDR/AM
BP	GO:00107	regulation	5/905	25/18866	0.005988	0.016118	0.008471	ADIPOQ/M
BP	GO:00433	positive re	5/905	25/18866	0.005988	0.016118	0.008471	FCER1G/HL
BP	GO:00506	negative re	5/905	25/18866	0.005988	0.016118	0.008471	MICB/NLRX
BP	GO:00602	regulation	5/905	25/18866	0.005988	0.016118	0.008471	QRFP/RETN
BP	GO:00606	embryonic	5/905	25/18866	0.005988	0.016118	0.008471	SOCS3/IL1
BP	GO:00612	regulation	5/905	25/18866	0.005988	0.016118	0.008471	VEGFA/AG
BP	GO:19055	positive re	5/905	25/18866	0.005988	0.016118	0.008471	AKT3/APLN
BP	GO:20010	regulation	5/905	25/18866	0.005988	0.016118	0.008471	CXCL13/TN
BP	GO:00600	heart cont	24/905	287/18866	0.006	0.016133	0.008479	S100A1/DE
BP	GO:00215	cell prolife	4/905	16/18866	0.006026	0.016133	0.008479	C5AR1/EGI
BP	GO:00425	negative re	4/905	16/18866	0.006026	0.016133	0.008479	SOCS3/SO
BP	GO:00429	cytoplasmi	4/905	16/18866	0.006026	0.016133	0.008479	PSMD10/T
BP	GO:00431	negative re	4/905	16/18866	0.006026	0.016133	0.008479	VEGFA/AD
BP	GO:00453	MHC class	4/905	16/18866	0.006026	0.016133	0.008479	IFNG/SLC1
BP	GO:00608	regulation	4/905	16/18866	0.006026	0.016133	0.008479	PPARG/RO
BP	GO:00614	trophoblas	4/905	16/18866	0.006026	0.016133	0.008479	CALR/VEG
BP	GO:00721	glomerular	4/905	16/18866	0.006026	0.016133	0.008479	PDGFRB/PI
BP	GO:00722	metaneph	4/905	16/18866	0.006026	0.016133	0.008479	ADIPOQ/PI
BP	GO:19011	regulation	4/905	16/18866	0.006026	0.016133	0.008479	CALR/VEG
BP	GO:00217	limbic syst	12/905	109/18866	0.006029	0.016134	0.008479	HDAC1/GS
BP	GO:00452	cell-cell ju	19/905	210/18866	0.006063	0.016215	0.008522	TGFB1/IL1
BP	GO:00068	cellular iro	9/905	70/18866	0.006085	0.016266	0.008549	IFNG/LCN2
BP	GO:00218	forebrain c	8/905	58/18866	0.006207	0.016561	0.008703	CXCL12/CX
BP	GO:19004	negative re	8/905	58/18866	0.006207	0.016561	0.008703	IL10/MET/
BP	GO:19032	negative re	8/905	58/18866	0.006207	0.016561	0.008703	IL10/MET/
BP	GO:19037	positive re	8/905	58/18866	0.006207	0.016561	0.008703	RAC2/GZM
BP	GO:00015	vasculoger	10/905	83/18866	0.006265	0.016707	0.00878	TGFB1/VE
BP	GO:00485	embryonic	34/905	451/18866	0.006353	0.016933	0.008899	CXCL8/TGF
BP	GO:19018	positive re	12/905	110/18866	0.006481	0.017257	0.009069	PSMC2/PS
BP	GO:19046	glucose tra	12/905	110/18866	0.006481	0.017257	0.009069	IL1B/FABP
BP	GO:00358	epithelial c	7/905	47/18866	0.006714	0.017843	0.009377	MMP9/AD
BP	GO:00433	mast cell d	7/905	47/18866	0.006714	0.017843	0.009377	FCER1G/BT
BP	GO:00457	negative re	7/905	47/18866	0.006714	0.017843	0.009377	ADIPOQ/N
BP	GO:00484	autonomic	7/905	47/18866	0.006714	0.017843	0.009377	SEMA3A/E
BP	GO:00364	cell death i	11/905	97/18866	0.006721	0.017853	0.009383	PML/IL10/
BP	GO:00015	branching i	6/905	36/18866	0.006797	0.018036	0.009479	VEGFA/DLL
BP	GO:00062	pyrimidine	6/905	36/18866	0.006797	0.018036	0.009479	APOBEC3G
BP	GO:00066	unsaturate	8/905	59/18866	0.006887	0.018265	0.009599	PTGDS/IL1
BP	GO:00511	positive re	12/905	111/18866	0.006959	0.018447	0.009695	CXCL9/TGF
BP	GO:00309	endoplasm	13/905	125/18866	0.007028	0.018613	0.009782	CALR/HSPA
BP	GO:00346	cellular res	13/905	125/18866	0.007028	0.018613	0.009782	HSP90AA1/
BP	GO:00020	glandular e	5/905	26/18866	0.007126	0.018798	0.009879	WNT5A/GS
BP	GO:00218	forebrain n	5/905	26/18866	0.007126	0.018798	0.009879	WNT5A/SE
BP	GO:00435	skeletal mu	5/905	26/18866	0.007126	0.018798	0.009879	IL15/ACTA
BP	GO:00607	prostate gl	5/905	26/18866	0.007126	0.018798	0.009879	WNT5A/FG
BP	GO:00722	cell differe	5/905	26/18866	0.007126	0.018798	0.009879	ADIPOQ/ST
BP	GO:01400	neuron prc	5/905	26/18866	0.007126	0.018798	0.009879	WNT5A/SE
BP	GO:19030	regulation	5/905	26/18866	0.007126	0.018798	0.009879	LTF/TNFSF

BP	GO:190475	positive reg	5/905	26/18866	0.007126	0.018798	0.009879	AGT/PDGFI
BP	GO:005080	regulation	20/905	229/18866	0.007269	0.019166	0.010072	TLR2/TNF/'
BP	GO:004683	lipid phosp	9/905	72/18866	0.007318	0.019285	0.010135	SOCS3/SOC
BP	GO:003476	regulation	36/905	489/18866	0.007324	0.019294	0.01014	HSPA2/IFN
BP	GO:000697	response to	10/905	85/18866	0.0074	0.019485	0.01024	HSP90AB1/
BP	GO:007265	establishm	14/905	140/18866	0.007415	0.019515	0.010256	HSP90AA1/
BP	GO:000227	mast cell a	7/905	48/18866	0.007543	0.019821	0.010417	FCER1G/BT
BP	GO:001079	regulation	7/905	48/18866	0.007543	0.019821	0.010417	TGFB1/WN
BP	GO:190403	negative re	7/905	48/18866	0.007543	0.019821	0.010417	ICAM1/TNI
BP	GO:000203	G protein-c	4/905	17/18866	0.007585	0.019865	0.01044	ARRB1/ADI
BP	GO:003094	positive reg	4/905	17/18866	0.007585	0.019865	0.01044	IL1B/FGF10
BP	GO:004244	progestero	4/905	17/18866	0.007585	0.019865	0.01044	ADM/LHB/
BP	GO:004562	negative re	4/905	17/18866	0.007585	0.019865	0.01044	TNFSF4/JAI
BP	GO:004853	anatomical	4/905	17/18866	0.007585	0.019865	0.01044	HSPA5/SEN
BP	GO:007024	thymocyte	4/905	17/18866	0.007585	0.019865	0.01044	WNT5A/HI
BP	GO:009706	cellular res	4/905	17/18866	0.007585	0.019865	0.01044	CTSB/CTSL
BP	GO:190288	negative re	8/905	60/18866	0.007621	0.019948	0.010483	IL10/MET/I
BP	GO:000751	muscle org	31/905	407/18866	0.00772	0.020197	0.010614	CXCL10/S1
BP	GO:003021	hyaluronar	6/905	37/18866	0.007791	0.020373	0.010707	TGFB1/IL1I
BP	GO:001038	regulation	18/905	200/18866	0.007831	0.020459	0.010752	HSPA2/HSI
BP	GO:001703	protein imp	18/905	200/18866	0.007831	0.020459	0.010752	HSP90AA1/
BP	GO:000170	formation	13/905	127/18866	0.008003	0.020899	0.010983	MMP9/ITG
BP	GO:003410	homotypic	10/905	86/18866	0.008025	0.020925	0.010997	CCL5/TNFS
BP	GO:003502	regulation	10/905	86/18866	0.008025	0.020925	0.010997	F2R/F2RL1
BP	GO:190186	positive reg	10/905	86/18866	0.008025	0.020925	0.010997	TGFB1/GRI
BP	GO:005117	import into	16/905	171/18866	0.008243	0.021484	0.011291	HSP90AB1/
BP	GO:000729	female gan	14/905	142/18866	0.00837	0.021803	0.011458	SRC/PTX3/
BP	GO:000202	response to	5/905	27/18866	0.008407	0.021808	0.011461	BMP8A/VG
BP	GO:000202	regulation	5/905	27/18866	0.008407	0.021808	0.011461	NOS1/ADN
BP	GO:000314	outflow tra	5/905	27/18866	0.008407	0.021808	0.011461	SEMA3C/R
BP	GO:007253	T-helper 17	5/905	27/18866	0.008407	0.021808	0.011461	IL23A/IL12
BP	GO:190012	regulation	5/905	27/18866	0.008407	0.021808	0.011461	B2M/MMP
BP	GO:190320	regulation	5/905	27/18866	0.008407	0.021808	0.011461	IL10/NR4A
BP	GO:000602	glycosamin	8/905	61/18866	0.008412	0.021808	0.011461	PGLYRP1/P
BP	GO:190211	positive reg	8/905	61/18866	0.008412	0.021808	0.011461	GSK3B/GZI
BP	GO:190390	positive reg	8/905	61/18866	0.008412	0.021808	0.011461	CD4/PPIA/
BP	GO:200075	regulation	8/905	61/18866	0.008412	0.021808	0.011461	IL1B/MAPK
BP	GO:003085	prostate gl	7/905	49/18866	0.008445	0.021872	0.011495	WNT5A/FG
BP	GO:012017	steroid hor	7/905	49/18866	0.008445	0.021872	0.011495	ADM/BMP
BP	GO:000864	hexose trai	12/905	114/18866	0.00856	0.022161	0.011646	IL1B/FABP5
BP	GO:003051	negative re	10/905	87/18866	0.008689	0.022472	0.01181	HSPA5/TGI
BP	GO:005507	iron ion ho	10/905	87/18866	0.008689	0.022472	0.01181	B2M/IFNG/
BP	GO:004863	positive reg	17/905	187/18866	0.008708	0.022511	0.01183	CXCL12/VE
BP	GO:002153	diencephal	9/905	74/18866	0.008732	0.022563	0.011858	WNT5A/FG
BP	GO:001491	negative re	6/905	38/18866	0.008884	0.022891	0.01203	ADIPOQ/SI
BP	GO:003288	regulation	6/905	38/18866	0.008884	0.022891	0.01203	TGFB1/GSK
BP	GO:004302	T cell home	6/905	38/18866	0.008884	0.022891	0.01203	IL2RA/JAK3
BP	GO:004577	positive reg	6/905	38/18866	0.008884	0.022891	0.01203	AGT/OXT/
BP	GO:005195	positive reg	6/905	38/18866	0.008884	0.022891	0.01203	CXCL12/AG
BP	GO:190002	positive reg	6/905	38/18866	0.008884	0.022891	0.01203	CALR/LIMS

BP	GO:190288	regulation	11/905	101/18866	0.009045	0.023292	0.012241	TNF/IL10/N
BP	GO:006041	heart grow	12/905	115/18866	0.009154	0.023561	0.012382	RBP4/AGT/
BP	GO:005178	regulation	17/905	188/18866	0.00916	0.023568	0.012386	CALR/IL1B/
BP	GO:005185	modulation	8/905	62/18866	0.009263	0.02381	0.012513	LTF/CCL5/C
BP	GO:007138	cellular res	8/905	62/18866	0.009263	0.02381	0.012513	ICAM1/STC
BP	GO:003265	regulation	4/905	18/18866	0.009388	0.024073	0.012651	IFNL1/TNF
BP	GO:004659	negative re	4/905	18/18866	0.009388	0.024073	0.012651	PTX3/IFITM
BP	GO:006003	anatomical	4/905	18/18866	0.009388	0.024073	0.012651	AMH/FLT1/
BP	GO:006097	coronary v	4/905	18/18866	0.009388	0.024073	0.012651	VEGFA/PD
BP	GO:190022	regulation	4/905	18/18866	0.009388	0.024073	0.012651	IFNG/TNF/
BP	GO:001021	response to	14/905	144/18866	0.009418	0.024073	0.012651	HSPA5/ICA
BP	GO:007058	protein loc	14/905	144/18866	0.009418	0.024073	0.012651	HSP90AA1/
BP	GO:001071	positive re	7/905	50/18866	0.009423	0.024073	0.012651	TGFB1/IL1
BP	GO:003019	negative re	7/905	50/18866	0.009423	0.024073	0.012651	PLAU/PRO
BP	GO:003164	protein de	7/905	50/18866	0.009423	0.024073	0.012651	SRC/BMP2/
BP	GO:004854	digestive tr	7/905	50/18866	0.009423	0.024073	0.012651	WNT5A/FG
BP	GO:005087	brown fat c	7/905	50/18866	0.009423	0.024073	0.012651	FABP3/ADI
BP	GO:190358	positive re	7/905	50/18866	0.009423	0.024073	0.012651	IFNG/TMSI
BP	GO:000170	mesoderm	9/905	75/18866	0.009513	0.024245	0.012742	WNT5A/DK
BP	GO:004362	response to	9/905	75/18866	0.009513	0.024245	0.012742	PDGFRB/PI
BP	GO:005080	negative re	9/905	75/18866	0.009513	0.024245	0.012742	IL1B/ADIPC
BP	GO:190007	regulation	9/905	75/18866	0.009513	0.024245	0.012742	IL1B/SOCS3
BP	GO:190198	regulation	9/905	75/18866	0.009513	0.024245	0.012742	IL1B/MAPK
BP	GO:000007	regulation	11/905	102/18866	0.009712	0.024729	0.012996	PSMD10/T
BP	GO:006084	artery deve	11/905	102/18866	0.009712	0.024729	0.012996	VEGFA/DLL
BP	GO:001574	monosacch	12/905	116/18866	0.009778	0.024874	0.013072	IL1B/FABP5
BP	GO:004691	cellular tra	12/905	116/18866	0.009778	0.024874	0.013072	IFNG/LCN2
BP	GO:000233	mature B c	5/905	28/18866	0.009838	0.024979	0.013128	PTK2B/CD1
BP	GO:000236	T cell linea	5/905	28/18866	0.009838	0.024979	0.013128	CTSL/IL23A
BP	GO:006051	prostate gl	5/905	28/18866	0.009838	0.024979	0.013128	WNT5A/FG
BP	GO:008011	DNA deme	5/905	28/18866	0.009838	0.024979	0.013128	APOBEC3G
BP	GO:190199	regulation	33/905	448/18866	0.009867	0.02504	0.013159	HSPA2/HSP
BP	GO:000820	steroid me	26/905	332/18866	0.009957	0.025258	0.013274	IFNG/NFYA
BP	GO:000719	activation c	6/905	39/18866	0.010084	0.025543	0.013424	EDNRA/AD
BP	GO:001045	regulation	6/905	39/18866	0.010084	0.025543	0.013424	WNT5A/DK
BP	GO:190222	regulation	6/905	39/18866	0.010084	0.025543	0.013424	CXCL12/TP
BP	GO:190384	negative re	10/905	89/18866	0.010142	0.025651	0.013481	HSPA5/TGI
BP	GO:190268	mitochond	8/905	63/18866	0.010177	0.025651	0.013481	GSK3B/GZI
BP	GO:000155	luteinizatio	3/905	10/18866	0.010245	0.025651	0.013481	INHBA/RET
BP	GO:000229	T cell activ	3/905	10/18866	0.010245	0.025651	0.013481	HLA-DMB/
BP	GO:000330	regulation	3/905	10/18866	0.010245	0.025651	0.013481	WNT5A/BN
BP	GO:001404	regulation	3/905	10/18866	0.010245	0.025651	0.013481	RAC3/EDNI
BP	GO:003281	regulation	3/905	10/18866	0.010245	0.025651	0.013481	IL15/FLT3L
BP	GO:003435	regulation	3/905	10/18866	0.010245	0.025651	0.013481	CCL2/PRKC
BP	GO:003558	sequesterii	3/905	10/18866	0.010245	0.025651	0.013481	LTBP1/GRE
BP	GO:004308	penile erec	3/905	10/18866	0.010245	0.025651	0.013481	EDNRB/OX
BP	GO:004571	low-density	3/905	10/18866	0.010245	0.025651	0.013481	ADIPOQ/IT
BP	GO:004659	positive re	3/905	10/18866	0.010245	0.025651	0.013481	CD4/LGALS
BP	GO:004826	determinat	3/905	10/18866	0.010245	0.025651	0.013481	GREM1/GF
BP	GO:005154	elastin met	3/905	10/18866	0.010245	0.025651	0.013481	CTSL/MMP

BP	GO:00700¢	regulation	3/905	10/18866	0.010245	0.025651	0.013481	CCL5/HIF1/
BP	GO:007047	regulation	3/905	10/18866	0.010245	0.025651	0.013481	OXT/GPER1
BP	GO:00709¢	neutrophil	3/905	10/18866	0.010245	0.025651	0.013481	CTSG/F2RL
BP	GO:00711(	response t	3/905	10/18866	0.010245	0.025651	0.013481	STAT1/IL2F
BP	GO:00716¢	regulation	3/905	10/18866	0.010245	0.025651	0.013481	ARG2/IL10,
BP	GO:007212	regulation	3/905	10/18866	0.010245	0.025651	0.013481	PDGFB/PD(
BP	GO:00722(	cell prolife	3/905	10/18866	0.010245	0.025651	0.013481	STAT1/PDG
BP	GO:00752¢	positive re	3/905	10/18866	0.010245	0.025651	0.013481	CD4/LGALS
BP	GO:190202	L-arginine	3/905	10/18866	0.010245	0.025651	0.013481	SLC11A1/A
BP	GO:190223	positive re	3/905	10/18866	0.010245	0.025651	0.013481	PMAIP1/PT
BP	GO:190433	regulation	3/905	10/18866	0.010245	0.025651	0.013481	GSK3B/DKI
BP	GO:00988€	cellular oxi	11/905	103/18866	0.010417	0.02607	0.013701	S100A9/DU
BP	GO:002187	forebrain n	7/905	51/18866	0.010483	0.026197	0.013767	B2M/WNT.
BP	GO:004591	negative re	7/905	51/18866	0.010483	0.026197	0.013767	TGFB1/ADI
BP	GO:19000¢	negative re	7/905	51/18866	0.010483	0.026197	0.013767	PLAU/PRO(
BP	GO:00900€	positive re	24/905	302/18866	0.010937	0.027319	0.014357	HSPA2/IL1I
BP	GO:00000E	G2/M trans	21/905	254/18866	0.010982	0.02742	0.01441	HSPA2/HSF
BP	GO:001591	sterol tran	12/905	118/18866	0.011127	0.027756	0.014587	CETP/ADIP
BP	GO:003421	carbohydra	12/905	118/18866	0.011127	0.027756	0.014587	IL1B/FABP!
BP	GO:00018E	endothelia	8/905	64/18866	0.011156	0.027778	0.014598	ICAM1/IL1I
BP	GO:00020€	columnar/	8/905	64/18866	0.011156	0.027778	0.014598	WNT5A/SR
BP	GO:003283	glomerulus	8/905	64/18866	0.011156	0.027778	0.014598	ADIPOQ/PI
BP	GO:00333¢	cholesterol	8/905	64/18866	0.011156	0.027778	0.014598	CETP/ADIP
BP	GO:00515¢	response t	14/905	147/18866	0.011182	0.027829	0.014625	HSPA5/S10
BP	GO:000742	peripheral	9/905	77/18866	0.011231	0.027901	0.014663	NDRG1/ED
BP	GO:004833	mesoderm	9/905	77/18866	0.011231	0.027901	0.014663	WNT5A/DK
BP	GO:007202	nephron m	9/905	77/18866	0.011231	0.027901	0.014663	STAT1/VEG
BP	GO:19032(	regulation	9/905	77/18866	0.011231	0.027901	0.014663	IL10/MET/I
BP	GO:00072E	activation	6/905	40/18866	0.011394	0.028115	0.014776	WNT5A/D/
BP	GO:00082(	C21-steroi	6/905	40/18866	0.011394	0.028115	0.014776	ADM/BMP
BP	GO:190342	negative re	6/905	40/18866	0.011394	0.028115	0.014776	IL10/FYN/H
BP	GO:00017E	B cell hom	5/905	29/18866	0.011429	0.028115	0.014776	TNFAIP3/M
BP	GO:00072€	nitric oxide	5/905	29/18866	0.011429	0.028115	0.014776	VEGFA/NO
BP	GO:00321¢	transpositi	5/905	29/18866	0.011429	0.028115	0.014776	APOBEC3G
BP	GO:003237	positive re	5/905	29/18866	0.011429	0.028115	0.014776	CETP/ADIP
BP	GO:003237	positive re	5/905	29/18866	0.011429	0.028115	0.014776	CETP/ADIP
BP	GO:00362¢	response t	5/905	29/18866	0.011429	0.028115	0.014776	PDGFRB/PI
BP	GO:004567	negative re	5/905	29/18866	0.011429	0.028115	0.014776	LTF/TLR3/(
BP	GO:00019¢	regulation	4/905	19/18866	0.011448	0.028115	0.014776	CTSG/F2R/
BP	GO:000292	positive re	4/905	19/18866	0.011448	0.028115	0.014776	LTA/IL1B/T
BP	GO:00031!	morphoge	4/905	19/18866	0.011448	0.028115	0.014776	CXCL10/DL
BP	GO:00359E	chondrocy	4/905	19/18866	0.011448	0.028115	0.014776	LTF/STC1/E
BP	GO:004337	CD4-positi	4/905	19/18866	0.011448	0.028115	0.014776	CTSL/IL23A
BP	GO:004432	cellular res	4/905	19/18866	0.011448	0.028115	0.014776	EBI3/LEPR/
BP	GO:004662	gamma-de	4/905	19/18866	0.011448	0.028115	0.014776	MICA/MICI
BP	GO:00484E	parasympa	4/905	19/18866	0.011448	0.028115	0.014776	SEMA3A/P
BP	GO:00515¢	keratinocy	4/905	19/18866	0.011448	0.028115	0.014776	MMP9/FGI
BP	GO:00602!	positive re	4/905	19/18866	0.011448	0.028115	0.014776	LTA/IL1B/T
BP	GO:00606(	branch elo	4/905	19/18866	0.011448	0.028115	0.014776	WNT5A/FG
BP	GO:00611!	endothelia	4/905	19/18866	0.011448	0.028115	0.014776	CXCL10/DL

BP	GO:00718(	podosome	4/905	19/18866	0.011448	0.028115	0.014776	TNF/SRC/H
BP	GO:190397	regulation	4/905	19/18866	0.011448	0.028115	0.014776	CCL3/CX3C
BP	GO:20006S	regulation	4/905	19/18866	0.011448	0.028115	0.014776	MMP9/AD
BP	GO:005507	sodium ion	7/905	52/18866	0.011626	0.028526	0.014991	EDNRB/AG
BP	GO:007213	mesenchyr	7/905	52/18866	0.011626	0.028526	0.014991	WNT5A/AC
BP	GO:00514S	regulation	10/905	91/18866	0.011774	0.028863	0.015168	NOX4/PIK3
BP	GO:006002	roof of mo	10/905	91/18866	0.011774	0.028863	0.015168	WNT5A/GD
BP	GO:00070E	regulation	15/905	163/18866	0.011906	0.029173	0.015332	IL1B/IL1A/I
BP	GO:00303(	cholesterol	11/905	105/18866	0.011944	0.029255	0.015375	CETP/ADIP
BP	GO:007233	intrinsic ap	9/905	78/18866	0.012173	0.029801	0.015662	PML/MIF/I
BP	GO:00357S	positive reg	8/905	65/18866	0.012203	0.029835	0.01568	GSK3B/GZI
BP	GO:00467E	regulation	8/905	65/18866	0.012203	0.029835	0.01568	CCL5/CCL4
BP	GO:190595	negative re	8/905	65/18866	0.012203	0.029835	0.01568	TNF/ITGAV
BP	GO:190305	regulation	19/905	225/18866	0.012243	0.029919	0.015724	HSP90AB1/
BP	GO:007115	regulation	11/905	106/18866	0.012769	0.031191	0.016392	CALR/HSP9
BP	GO:00425S	fear respor	6/905	41/18866	0.01282	0.031301	0.01645	MDK/UCN/
BP	GO:00456E	negative re	7/905	53/18866	0.012856	0.031332	0.016466	TNF/AREG/
BP	GO:00517(	intraspecie	7/905	53/18866	0.012856	0.031332	0.016466	LTF/BPIFA1
BP	GO:007132	cellular res	7/905	53/18866	0.012856	0.031332	0.016466	HSPA5/NO
BP	GO:190002	regulation	7/905	53/18866	0.012856	0.031332	0.016466	CALR/LIMS
BP	GO:190113	carbohydra	17/905	195/18866	0.012877	0.03137	0.016486	CTSL/MMP
BP	GO:00336E	regulation	5/905	30/18866	0.013186	0.032035	0.016835	LTF/ITGAV/
BP	GO:003647	neuron dea	5/905	30/18866	0.013186	0.032035	0.016835	IL10/NR4A
BP	GO:004814	negative re	5/905	30/18866	0.013186	0.032035	0.016835	LTA/PPARG
BP	GO:00714E	cellular res	5/905	30/18866	0.013186	0.032035	0.016835	HSPA5/NO
BP	GO:009031	positive reg	5/905	30/18866	0.013186	0.032035	0.016835	CHP1/ITGB
BP	GO:19021E	regulation	5/905	30/18866	0.013186	0.032035	0.016835	PML/TRIM
BP	GO:19021(	regulation	8/905	66/18866	0.013321	0.032333	0.016992	GSK3B/GZI
BP	GO:200037	negative re	8/905	66/18866	0.013321	0.032333	0.016992	IL10/FYN/H
BP	GO:000652	arginine ca	3/905	11/18866	0.013591	0.032421	0.017039	ARG2/NOS
BP	GO:001073	positive reg	3/905	11/18866	0.013591	0.032421	0.017039	ADIPOQ/M
BP	GO:00148E	muscle atr	3/905	11/18866	0.013591	0.032421	0.017039	IL15/PPAR
BP	GO:001937	cyclooxyge	3/905	11/18866	0.013591	0.032421	0.017039	PTGDS/PTC
BP	GO:00215E	facial nerve	3/905	11/18866	0.013591	0.032421	0.017039	SEMA3A/P
BP	GO:00216(	cranial ner	3/905	11/18866	0.013591	0.032421	0.017039	SEMA3A/P
BP	GO:002161	facial nerve	3/905	11/18866	0.013591	0.032421	0.017039	SEMA3A/P
BP	GO:003227	regulation	3/905	11/18866	0.013591	0.032421	0.017039	INHA/INHB
BP	GO:003314	positive reg	3/905	11/18866	0.013591	0.032421	0.017039	AR/PAK1/S
BP	GO:00334E	gas homeo	3/905	11/18866	0.013591	0.032421	0.017039	HIF1A/CAV
BP	GO:003411	negative re	3/905	11/18866	0.013591	0.032421	0.017039	ADIPOQ/IL
BP	GO:003545	cellular res	3/905	11/18866	0.013591	0.032421	0.017039	IFIT3/AXL/I
BP	GO:003953	negative re	3/905	11/18866	0.013591	0.032421	0.017039	NLRX1/DH
BP	GO:00450E	negative th	3/905	11/18866	0.013591	0.032421	0.017039	CCR7/ZAP7
BP	GO:004562	regulation	3/905	11/18866	0.013591	0.032421	0.017039	TNFSF4/CD
BP	GO:004664	negative re	3/905	11/18866	0.013591	0.032421	0.017039	XCL1/ARG2
BP	GO:00613(	cardiac neu	3/905	11/18866	0.013591	0.032421	0.017039	MAPK3/SE
BP	GO:00613(	cardiac neu	3/905	11/18866	0.013591	0.032421	0.017039	MAPK3/SE
BP	GO:007033	response t	3/905	11/18866	0.013591	0.032421	0.017039	TLR2/TLR6/
BP	GO:00705E	regulation	3/905	11/18866	0.013591	0.032421	0.017039	ADIPOQ/IL
BP	GO:007122	cellular res	3/905	11/18866	0.013591	0.032421	0.017039	TLR2/TLR6/

BP	GO:00712	cellular res	3/905	11/18866	0.013591	0.032421	0.017039	TLR2/TLR6,
BP	GO:00712	cellular res	3/905	11/18866	0.013591	0.032421	0.017039	B2M/NFYA
BP	GO:00716	chemokine	3/905	11/18866	0.013591	0.032421	0.017039	ARG2/IL10,
BP	GO:00718	positive re	3/905	11/18866	0.013591	0.032421	0.017039	TNF/SRC/C
BP	GO:00970	dendritic c	3/905	11/18866	0.013591	0.032421	0.017039	CXCL12/CC
BP	GO:00973	response t	3/905	11/18866	0.013591	0.032421	0.017039	IL17C/IL17
BP	GO:00973	cellular res	3/905	11/18866	0.013591	0.032421	0.017039	IL17C/IL17
BP	GO:00974	interleukin	3/905	11/18866	0.013591	0.032421	0.017039	IL17C/IL17
BP	GO:19024	positive re	3/905	11/18866	0.013591	0.032421	0.017039	MAPT/WN
BP	GO:19039	positive re	3/905	11/18866	0.013591	0.032421	0.017039	CCL3/LRRK
BP	GO:19043	response t	3/905	11/18866	0.013591	0.032421	0.017039	GNAI1/CFT
BP	GO:19043	cellular res	3/905	11/18866	0.013591	0.032421	0.017039	GNAI1/CFT
BP	GO:19059	regulation	3/905	11/18866	0.013591	0.032421	0.017039	DKK1/WNT
BP	GO:19904	positive re	3/905	11/18866	0.013591	0.032421	0.017039	HSPA5/CEE
BP	GO:20000	positive re	3/905	11/18866	0.013591	0.032421	0.017039	WNT5A/W
BP	GO:20003	positive re	3/905	11/18866	0.013591	0.032421	0.017039	IL23A/IL12
BP	GO:20005	regulation	3/905	11/18866	0.013591	0.032421	0.017039	XCL1/RSAD
BP	GO:20006	regulation	3/905	11/18866	0.013591	0.032421	0.017039	CXCL12/CC
BP	GO:00033	type B pan	4/905	20/18866	0.013777	0.032721	0.017196	WNT5A/GS
BP	GO:00100	response t	4/905	20/18866	0.013777	0.032721	0.017196	HSPA5/PTG
BP	GO:00108	positive re	4/905	20/18866	0.013777	0.032721	0.017196	ADIPOQ/AI
BP	GO:00312	retinal gan	4/905	20/18866	0.013777	0.032721	0.017196	VEGFA/SEM
BP	GO:00439	ossification	4/905	20/18866	0.013777	0.032721	0.017196	LTF/BMP2/
BP	GO:00450	negative re	4/905	20/18866	0.013777	0.032721	0.017196	IL10/KHSR
BP	GO:00600	pericardiun	4/905	20/18866	0.013777	0.032721	0.017196	WNT5A/DL
BP	GO:00710	regulation	4/905	20/18866	0.013777	0.032721	0.017196	FABP3/FPR
BP	GO:19017	positive re	4/905	20/18866	0.013777	0.032721	0.017196	CXCL9/FLT
BP	GO:19044	negative re	4/905	20/18866	0.013777	0.032721	0.017196	IL10/KHSR
BP	GO:00086	carbohydra	14/905	151/18866	0.013923	0.033054	0.017371	IL1B/FABP
BP	GO:19033	regulation	21/905	260/18866	0.01401	0.033245	0.017471	HSP90AA1,
BP	GO:00066	steroid bio	17/905	197/18866	0.014132	0.033521	0.017616	IFNG/NFYA
BP	GO:00486	response t	9/905	80/18866	0.01423	0.033724	0.017723	APOD/TNC
BP	GO:00550	regulation	9/905	80/18866	0.01423	0.033724	0.017723	RBP4/IGF1,
BP	GO:00002	mitochond	6/905	42/18866	0.014367	0.034017	0.017877	MX1/MAP
BP	GO:00466	negative re	6/905	42/18866	0.014367	0.034017	0.017877	RBP4/PTK2
BP	GO:20012	positive re	16/905	182/18866	0.014424	0.034138	0.017941	IL1B/PML/
BP	GO:00706	regulation	8/905	67/18866	0.014511	0.034313	0.018033	SRC/MYH9
BP	GO:19057	positive re	8/905	67/18866	0.014511	0.034313	0.018033	GSK3B/GZI
BP	GO:00488	sensory sys	29/905	394/18866	0.015026	0.035516	0.018665	RBP4/VEGI
BP	GO:00023	natural kill	5/905	31/18866	0.015116	0.035651	0.018736	HLA-F/PGL
BP	GO:00217	olfactory b	5/905	31/18866	0.015116	0.035651	0.018736	WNT5A/SE
BP	GO:00459	positive re	5/905	31/18866	0.015116	0.035651	0.018736	F2R/PTGS2
BP	GO:00609	heart form	5/905	31/18866	0.015116	0.035651	0.018736	WNT5A/RC
BP	GO:19001	negative re	5/905	31/18866	0.015116	0.035651	0.018736	APOD/CHP
BP	GO:00217	hippocamp	9/905	81/18866	0.015351	0.036188	0.019018	HDAC1/GS
BP	GO:00550	transition r	13/905	138/18866	0.015412	0.036318	0.019086	B2M/IFNG,
BP	GO:00423	regulation	7/905	55/18866	0.015593	0.036727	0.019302	IL1B/FABP
BP	GO:00613	renal tubul	10/905	95/18866	0.015621	0.036779	0.019328	STAT1/VEG
BP	GO:00903	negative re	6/905	43/18866	0.016038	0.037711	0.019818	APOD/ADI
BP	GO:19010	regulation	6/905	43/18866	0.016038	0.037711	0.019818	TNF/IL10/N

BP	GO:200064	positive re	6/905	43/18866	0.016038	0.037711	0.019818	VEGFA/CX3
BP	GO:003103	actomyosir	17/905	200/18866	0.016191	0.038054	0.019999	NOX4/SRC/
BP	GO:000236	alpha-beta	4/905	21/18866	0.016386	0.03823	0.020091	CTSL/IL23A
BP	GO:000317	pulmonary	4/905	21/18866	0.016386	0.03823	0.020091	ROBO2/JAG
BP	GO:000911	vitamin bic	4/905	21/18866	0.016386	0.03823	0.020091	IFNG/IL1B/
BP	GO:003022	platelet for	4/905	21/18866	0.016386	0.03823	0.020091	PTPN6/MY
BP	GO:003261	interleukin	4/905	21/18866	0.016386	0.03823	0.020091	IFNL1/TNF
BP	GO:003592	steroid hor	4/905	21/18866	0.016386	0.03823	0.020091	AGT/INHBA
BP	GO:004369	reverse ch	4/905	21/18866	0.016386	0.03823	0.020091	CETP/ABCA
BP	GO:004682	negative re	4/905	21/18866	0.016386	0.03823	0.020091	APOD/CHP
BP	GO:004848	sympathet	4/905	21/18866	0.016386	0.03823	0.020091	SEMA3A/G
BP	GO:006006	uterus dev	4/905	21/18866	0.016386	0.03823	0.020091	RBP4/WNT
BP	GO:006071	labyrinthin	4/905	21/18866	0.016386	0.03823	0.020091	SOCS3/IL10
BP	GO:190320	negative re	4/905	21/18866	0.016386	0.03823	0.020091	IL10/NR4A
BP	GO:190397	regulation	4/905	21/18866	0.016386	0.03823	0.020091	CCL3/SPHK
BP	GO:190488	beta-caten	4/905	21/18866	0.016386	0.03823	0.020091	GSK3B/WN
BP	GO:200004	regulation	4/905	21/18866	0.016386	0.03823	0.020091	VEGFA/WN
BP	GO:200038	regulation	4/905	21/18866	0.016386	0.03823	0.020091	DKK1/WNT
BP	GO:200073	positive re	4/905	21/18866	0.016386	0.03823	0.020091	SEMA3C/P
BP	GO:004648	phosphatic	16/905	185/18866	0.016617	0.038751	0.020365	SOCS3/SO
BP	GO:190274	regulation	18/905	217/18866	0.0171	0.03986	0.020948	HSPA2/HSP
BP	GO:003003	lamellipodi	8/905	69/18866	0.01712	0.03989	0.020964	VAV3/VAV
BP	GO:000726	Rho protei	13/905	140/18866	0.017191	0.040037	0.021041	F2R/F2RL1/
BP	GO:000916	nucleoside	5/905	32/18866	0.017227	0.040069	0.021058	APOBEC3G
BP	GO:005138	response t	5/905	32/18866	0.017227	0.040069	0.021058	CYBB/SRC/
BP	GO:007129	cellular res	5/905	32/18866	0.017227	0.040069	0.021058	IL15/PPAR
BP	GO:000698	ER overloa	3/905	12/18866	0.017484	0.040236	0.021146	HSPA5/GSK
BP	GO:001023	vascular tr	3/905	12/18866	0.017484	0.040236	0.021146	FABP5/CD3
BP	GO:003273	positive re	3/905	12/18866	0.017484	0.040236	0.021146	TNFSF4/IL3
BP	GO:003295	inositol tris	3/905	12/18866	0.017484	0.040236	0.021146	PTK2B/PLC
BP	GO:003369	positive re	3/905	12/18866	0.017484	0.040236	0.021146	LTF/ITGAV/
BP	GO:003573	CD4-positi	3/905	12/18866	0.017484	0.040236	0.021146	XCL1/ARG2
BP	GO:003600	response t	3/905	12/18866	0.017484	0.040236	0.021146	TLR2/PTPN
BP	GO:003600	cellular res	3/905	12/18866	0.017484	0.040236	0.021146	TLR2/PTPN
BP	GO:003646	TRAIL-activ	3/905	12/18866	0.017484	0.040236	0.021146	TNFRSF10B
BP	GO:004211	endothelia	3/905	12/18866	0.017484	0.040236	0.021146	CXCL10/TG
BP	GO:004330	negative re	3/905	12/18866	0.017484	0.040236	0.021146	HLA-F/CD3
BP	GO:004338	negative T	3/905	12/18866	0.017484	0.040236	0.021146	CCR7/ZAP7
BP	GO:004369	dedifferent	3/905	12/18866	0.017484	0.040236	0.021146	PDGFB/ESF
BP	GO:004369	cell dediffe	3/905	12/18866	0.017484	0.040236	0.021146	PDGFB/ESF
BP	GO:005154	positive re	3/905	12/18866	0.017484	0.040236	0.021146	MMP9/FGF
BP	GO:006045	regulation	3/905	12/18866	0.017484	0.040236	0.021146	OXT/UCN/
BP	GO:006067	branching i	3/905	12/18866	0.017484	0.040236	0.021146	SOCS3/IL10
BP	GO:007026	pyroptosis	3/905	12/18866	0.017484	0.040236	0.021146	AIM2/NLR
BP	GO:007058	cell-cell ad	3/905	12/18866	0.017484	0.040236	0.021146	ADIPOQ/IL
BP	GO:007258	caveolin-m	3/905	12/18866	0.017484	0.040236	0.021146	MAPK3/SR
BP	GO:190011	extracellula	3/905	12/18866	0.017484	0.040236	0.021146	LTBP1/GRE
BP	GO:190011	extracellula	3/905	12/18866	0.017484	0.040236	0.021146	LTBP1/GRE
BP	GO:190396	positive re	3/905	12/18866	0.017484	0.040236	0.021146	AGT/ITGB1
BP	GO:190495	Wnt signali	3/905	12/18866	0.017484	0.040236	0.021146	WNT5A/W

BP	GO:20005€	regulation	3/905	12/18866	0.017484	0.040236	0.021146	XCL1/ARG2
BP	GO:003167	cellular res	6/905	44/18866	0.017839	0.040984	0.021538	IL15/PPAR(
BP	GO:003288	regulation	6/905	44/18866	0.017839	0.040984	0.021538	TGFB1/GSK
BP	GO:004218	ketone bio:	6/905	44/18866	0.017839	0.040984	0.021538	ADM/BMP
BP	GO:19030£	regulation	6/905	44/18866	0.017839	0.040984	0.021538	TGFB1/AG'
BP	GO:199082	response t(	10/905	97/18866	0.017863	0.041003	0.021549	ICAM1/PM
BP	GO:19908:	cellular res	10/905	97/18866	0.017863	0.041003	0.021549	ICAM1/PM
BP	GO:003432	cell junctio	31/905	434/18866	0.017989	0.041274	0.021691	TLR2/IL1B/
BP	GO:00309(	hindbrain (	14/905	156/18866	0.018046	0.041389	0.021751	HSPA5/SEN
BP	GO:190198	regulation	34/905	486/18866	0.018133	0.041559	0.021841	HSPA2/HSP
BP	GO:000758	digestion	13/905	141/18866	0.018136	0.041559	0.021841	RBP4/CHIT
BP	GO:000602	aminoglyca	15/905	172/18866	0.018655	0.04273	0.022456	PGLYRP1/P
BP	GO:003052	intracellula	7/905	57/18866	0.018719	0.042823	0.022505	SRC/AR/ES
BP	GO:00610(	cell differe	7/905	57/18866	0.018719	0.042823	0.022505	MMP9/AD
BP	GO:00705:	protein K6:	7/905	57/18866	0.018719	0.042823	0.022505	TRAF3/TRI
BP	GO:005101	actin filam(	14/905	157/18866	0.018972	0.043383	0.022799	NOX4/SRC/
BP	GO:001701	regulation	12/905	127/18866	0.019041	0.043522	0.022872	HSPA5/HSP
BP	GO:00105€	positive re	4/905	22/18866	0.019285	0.043931	0.023087	IL15/CCR7/
BP	GO:00341(	negative re	4/905	22/18866	0.019285	0.043931	0.023087	TNFAIP3/A
BP	GO:003558	calcium-m(	4/905	22/18866	0.019285	0.043931	0.023087	DEFB1/CCL
BP	GO:003634	platelet mc	4/905	22/18866	0.019285	0.043931	0.023087	PTPN6/MY
BP	GO:00423£	vitamin D r	4/905	22/18866	0.019285	0.043931	0.023087	IFNG/IL1B/
BP	GO:00424£	ribonucleo	4/905	22/18866	0.019285	0.043931	0.023087	APOBEC3G
BP	GO:00433€	CD4-positi\	4/905	22/18866	0.019285	0.043931	0.023087	CTSL/IL23A
BP	GO:00456£	regulation	4/905	22/18866	0.019285	0.043931	0.023087	CD4/IRF7/I
BP	GO:00033:	metanephr	5/905	33/18866	0.019523	0.044343	0.023304	STAT1/PDC
BP	GO:00099£	proximal/d	5/905	33/18866	0.019523	0.044343	0.023304	FGF10/GRE
BP	GO:00100:	response t(	5/905	33/18866	0.019523	0.044343	0.023304	B2M/NFYA
BP	GO:00104€	regulation	5/905	33/18866	0.019523	0.044343	0.023304	STAT1/VEG
BP	GO:00109£	negative re	5/905	33/18866	0.019523	0.044343	0.023304	LRRK2/SER
BP	GO:004592	negative re	5/905	33/18866	0.019523	0.044343	0.023304	HLA-F/TRIN
BP	GO:190331	negative re	5/905	33/18866	0.019523	0.044343	0.023304	LRRK2/SER
BP	GO:00031¢	endocardia	6/905	45/18866	0.019774	0.044857	0.023574	ROBO2/BN
BP	GO:000677	fat-soluble	6/905	45/18866	0.019774	0.044857	0.023574	IFNG/RBP1
BP	GO:001067	negative re	6/905	45/18866	0.019774	0.044857	0.023574	ADIPOQ/G:
BP	GO:00302(	glycosamin	14/905	158/18866	0.019933	0.045198	0.023753	PGLYRP1/P
BP	GO:003237	regulation	8/905	71/18866	0.020051	0.045446	0.023884	CETP/ADIP
BP	GO:00353(	positive re	7/905	58/18866	0.020437	0.046302	0.024333	HSP90AB1/
BP	GO:00105(	negative re	9/905	85/18866	0.020485	0.046352	0.02436	IL10/PTK2/
BP	GO:004584	positive re	9/905	85/18866	0.020485	0.046352	0.02436	TGFB1/GRE
BP	GO:00486:	positive re	9/905	85/18866	0.020485	0.046352	0.02436	TGFB1/GRE
BP	GO:00550€	monovalen	14/905	159/18866	0.02093	0.047321	0.024869	MAPK3/SL(
BP	GO:20000£	regulation	14/905	159/18866	0.02093	0.047321	0.024869	HSP90AB1/
BP	GO:199074	cellular det	11/905	114/18866	0.021006	0.047471	0.024948	S100A9/DL
BP	GO:01500€	visual syste	28/905	388/18866	0.021018	0.047479	0.024952	RBP4/VEGI
BP	GO:190384	regulation	12/905	129/18866	0.021261	0.048009	0.02523	HSPA5/HSP
BP	GO:00107€	fibroblast r	6/905	46/18866	0.021847	0.049054	0.02578	TGFB1/PM
BP	GO:003164	regulation	6/905	46/18866	0.021847	0.049054	0.02578	S100B/TYN
BP	GO:006102	establishm	6/905	46/18866	0.021847	0.049054	0.02578	ICAM1/IL1I
BP	GO:00026:	negative re	3/905	13/18866	0.021933	0.049054	0.02578	CD22/IL33/

BP	GO:000267	negative re	3/905	13/18866	0.021933	0.049054	0.02578	PPARG/IL2
BP	GO:000292	negative re	3/905	13/18866	0.021933	0.049054	0.02578	SPINK5/PTI
BP	GO:000333	mesenchyr	3/905	13/18866	0.021933	0.049054	0.02578	STAT1/GDN
BP	GO:000749	midgut dev	3/905	13/18866	0.021933	0.049054	0.02578	WNT5A/ED
BP	GO:001064	negative re	3/905	13/18866	0.021933	0.049054	0.02578	APOD/ADII
BP	GO:001087	positive re	3/905	13/18866	0.021933	0.049054	0.02578	IFNG/HDA(
BP	GO:001580	arginine tra	3/905	13/18866	0.021933	0.049054	0.02578	SLC11A1/A
BP	GO:003166	negative re	3/905	13/18866	0.021933	0.049054	0.02578	LTF/TNFAIF
BP	GO:003195	negative re	3/905	13/18866	0.021933	0.049054	0.02578	ADIPOQ/CI
BP	GO:003242	regulation	3/905	13/18866	0.021933	0.049054	0.02578	AGTR1/EGI
BP	GO:003308	positive re	3/905	13/18866	0.021933	0.049054	0.02578	IL1B/IL7R/I
BP	GO:003314	positive re	3/905	13/18866	0.021933	0.049054	0.02578	AR/PAK1/S
BP	GO:004557	positive re	3/905	13/18866	0.021933	0.049054	0.02578	BTK/CD27/
BP	GO:004830	regulation	3/905	13/18866	0.021933	0.049054	0.02578	CD40/IL27I
BP	GO:004871	positive re	3/905	13/18866	0.021933	0.049054	0.02578	BMP2/CLCI
BP	GO:006031	regulation	3/905	13/18866	0.021933	0.049054	0.02578	TGFB1/FLT
BP	GO:006102	eyelid deve	3/905	13/18866	0.021933	0.049054	0.02578	HDAC1/INH
BP	GO:006146	regulation	3/905	13/18866	0.021933	0.049054	0.02578	NR4A1/NR
BP	GO:190499	negative re	3/905	13/18866	0.021933	0.049054	0.02578	CXCL12/CC
BP	GO:001076	regulation	5/905	34/18866	0.022012	0.049109	0.025808	TGFB1/PTK
BP	GO:001092	positive re	5/905	34/18866	0.022012	0.049109	0.025808	HSP90AB1/
BP	GO:003551	DNA dealky	5/905	34/18866	0.022012	0.049109	0.025808	APOBEC3G
BP	GO:003563	maintenan	5/905	34/18866	0.022012	0.049109	0.025808	VEGFA/PT(
BP	GO:005502	negative re	5/905	34/18866	0.022012	0.049109	0.025808	RBP4/PAK1
BP	GO:006111	negative re	5/905	34/18866	0.022012	0.049109	0.025808	RBP4/PAK1
BP	GO:000165	branching i	7/905	59/18866	0.022261	0.049583	0.026057	VEGFA/AG
BP	GO:004352	positive re	7/905	59/18866	0.022261	0.049583	0.026057	TNF/FASLG
BP	GO:004645	icosanoid b	7/905	59/18866	0.022261	0.049583	0.026057	PTGDS/IL1I
BP	GO:007138	cellular res	7/905	59/18866	0.022261	0.049583	0.026057	ICAM1/STC
BP	GO:000801	regulation	20/905	256/18866	0.022356	0.049774	0.026158	S100A1/DE
BP	GO:000205	positive re	4/905	23/18866	0.02248	0.049784	0.026164	VEGFA/CX3
BP	GO:000659	thyroid hor	4/905	23/18866	0.02248	0.049784	0.026164	CTSB/DUO
BP	GO:001022	response t	4/905	23/18866	0.02248	0.049784	0.026164	CALR/PTGS
BP	GO:003516	embryonic	4/905	23/18866	0.02248	0.049784	0.026164	VEGFA/FLT
BP	GO:005086	negative re	4/905	23/18866	0.02248	0.049784	0.026164	PTPN6/SH2
BP	GO:006014	negative re	4/905	23/18866	0.02248	0.049784	0.026164	TGFB1/TNF
BP	GO:006096	negative re	4/905	23/18866	0.02248	0.049784	0.026164	TGFB1/TNF
BP	GO:007097	bone matu	4/905	23/18866	0.02248	0.049784	0.026164	LTF/BMP2/
BP	GO:007130	cellular res	4/905	23/18866	0.02248	0.049784	0.026164	IL15/VDR/T
BP	GO:190103	negative re	4/905	23/18866	0.02248	0.049784	0.026164	IL10/MET/I
BP	GO:190152	positive re	4/905	23/18866	0.02248	0.049784	0.026164	NFYA/VEGI
BP	GO:190320	negative re	4/905	23/18866	0.02248	0.049784	0.026164	IL10/MET/I
BP	GO:200072	negative re	4/905	23/18866	0.02248	0.049784	0.026164	BMP2/DKK
BP	GO:004483	cell cycle G	21/905	273/18866	0.022825	0.050527	0.026554	HSPA2/HSI
BP	GO:006157	actin filam	14/905	161/18866	0.023037	0.050975	0.026789	NOX4/SRC/
BP	GO:005196	regulation	8/905	73/18866	0.023321	0.051583	0.027109	TNF/PTGS2
BP	GO:003243	positive re	9/905	87/18866	0.02347	0.051807	0.027226	PSMD10/G
BP	GO:004801	ephrin rece	9/905	87/18866	0.02347	0.051807	0.027226	MMP9/SR(
BP	GO:006029	long-term	9/905	87/18866	0.02347	0.051807	0.027226	S100B/AGE
BP	GO:006042	regulation	9/905	87/18866	0.02347	0.051807	0.027226	RBP4/IGF1

BP	GO:00975ɛ	lamellipodi	9/905	87/18866	0.02347	0.051807	0.027226	SRC/VAV3/
BP	GO:002241	cellular prc	27/905	375/18866	0.023729	0.052356	0.027515	HSPA2/DEI
BP	GO:003057	collagen ca	6/905	47/18866	0.024062	0.053005	0.027856	CTSB/CTSL,
BP	GO:004593	positive re	6/905	47/18866	0.024062	0.053005	0.027856	F2R/PTGS2
BP	GO:190473	vascular as	6/905	47/18866	0.024062	0.053005	0.027856	ADIPOQ/A(
BP	GO:190475	regulation	6/905	47/18866	0.024062	0.053005	0.027856	ADIPOQ/A(
BP	GO:001082	negative re	7/905	60/18866	0.024194	0.053253	0.027987	PSMD10/T
BP	GO:00359(	aorta deve	7/905	60/18866	0.024194	0.053253	0.027987	DLL4/PDGI
BP	GO:00226(	digestive s	10/905	102/18866	0.024493	0.053866	0.028308	RBP4/MUC
BP	GO:011002	regulation	10/905	102/18866	0.024493	0.053866	0.028308	NOX4/PIK3
BP	GO:200102	regulation	18/905	226/18866	0.024636	0.054159	0.028463	PSMD10/C
BP	GO:003026	apoptotic r	5/905	35/18866	0.024697	0.054162	0.028464	ACVR1C/GI
BP	GO:003094	regulation	5/905	35/18866	0.024697	0.054162	0.028464	IL1B/FGF1(
BP	GO:003368	osteoblast	5/905	35/18866	0.024697	0.054162	0.028464	LTF/ITGAV,
BP	GO:006025	regulation	5/905	35/18866	0.024697	0.054162	0.028464	LTA/IL1B/T
BP	GO:007088	regulation	5/905	35/18866	0.024697	0.054162	0.028464	TNF/CHP1/
BP	GO:190403	positive re	5/905	35/18866	0.024697	0.054162	0.028464	HSP90AB1,
BP	GO:003202	positive re	8/905	74/18866	0.02509	0.055	0.028904	RBP4/ARRI
BP	GO:000318	atrioventri	4/905	24/18866	0.02598	0.056813	0.029857	BMP2/ZFPI
BP	GO:004432	response t	4/905	24/18866	0.02598	0.056813	0.029857	EBI3/LEPR/
BP	GO:004582	positive re	4/905	24/18866	0.02598	0.056813	0.029857	IFNG/IGF1/
BP	GO:009874	cell aggreg	4/905	24/18866	0.02598	0.056813	0.029857	LTF/BPIFA1
BP	GO:190299	positive re	4/905	24/18866	0.02598	0.056813	0.029857	IFNG/TNF/
BP	GO:19033(	negative re	4/905	24/18866	0.02598	0.056813	0.029857	HLA-F/TRIN
BP	GO:000854	fibroblast g	11/905	118/18866	0.026349	0.057596	0.030269	MAPK3/WI
BP	GO:00456(	regulation	6/905	48/18866	0.026422	0.057686	0.030316	IL1B/TNF/V
BP	GO:005197	regulation	6/905	48/18866	0.026422	0.057686	0.030316	HSP90AA1,
BP	GO:005501	ventricular	6/905	48/18866	0.026422	0.057686	0.030316	TGFB1/DLL
BP	GO:00017(	neuron mi	14/905	164/18866	0.026489	0.057785	0.030368	CXCL12/VE
BP	GO:00461(	alcohol bio	14/905	164/18866	0.026489	0.057785	0.030368	IFNG/NFYA
BP	GO:00550(	cardiac cell	9/905	89/18866	0.026754	0.05825	0.030613	VEGFA/PD(
BP	GO:000177	immunolog	3/905	14/18866	0.026939	0.05825	0.030613	CCR7/PRF1
BP	GO:001087	regulation	3/905	14/18866	0.026939	0.05825	0.030613	AGT/AGTR
BP	GO:001487	response t	3/905	14/18866	0.026939	0.05825	0.030613	AGT/PPAR(
BP	GO:001989	axonal trar	3/905	14/18866	0.026939	0.05825	0.030613	MAPT/HIF1
BP	GO:003543	maintenan	3/905	14/18866	0.026939	0.05825	0.030613	HSPA5/PDI
BP	GO:003581	positive re	3/905	14/18866	0.026939	0.05825	0.030613	EDNRB/BT(
BP	GO:00381(	angiotensi	3/905	14/18866	0.026939	0.05825	0.030613	SRC/AGTR1
BP	GO:004505	positive th	3/905	14/18866	0.026939	0.05825	0.030613	ZAP70/CD3
BP	GO:004547	response t	3/905	14/18866	0.026939	0.05825	0.030613	OXT/CDK4/
BP	GO:00460(	negative re	3/905	14/18866	0.026939	0.05825	0.030613	ARG1/CD2
BP	GO:004632	positive re	3/905	14/18866	0.026939	0.05825	0.030613	PPARG/NR
BP	GO:00509(	detection c	3/905	14/18866	0.026939	0.05825	0.030613	CXCL12/CX
BP	GO:005154	regulation	3/905	14/18866	0.026939	0.05825	0.030613	MMP9/FGI
BP	GO:00713(	cellular res	3/905	14/18866	0.026939	0.05825	0.030613	CYBB/CCL7
BP	GO:009023	regulation	3/905	14/18866	0.026939	0.05825	0.030613	ARRB1/ML
BP	GO:190172	regulation	3/905	14/18866	0.026939	0.05825	0.030613	PDGFB/PD(
BP	GO:190289	negative re	3/905	14/18866	0.026939	0.05825	0.030613	PDGFB/PP/
BP	GO:200054	positive re	3/905	14/18866	0.026939	0.05825	0.030613	WNT3A/GJ
BP	GO:00159(	long-chain	8/905	75/18866	0.026949	0.05825	0.030613	FABP5/FAI

BP	GO:003241	lysosome l	8/905	75/18866	0.026949	0.05825	0.030613	FCER1G/BT
BP	GO:003314	regulation	8/905	75/18866	0.026949	0.05825	0.030613	CALR/SRC/
BP	GO:007208	nephron ep	8/905	75/18866	0.026949	0.05825	0.030613	STAT1/VEG
BP	GO:003105	regulation	13/905	149/18866	0.02717	0.058703	0.030851	IL1B/VEGF/
BP	GO:001054	regulation	5/905	36/18866	0.027584	0.059482	0.03126	FCER1G/PD
BP	GO:002195	central ner	5/905	36/18866	0.027584	0.059482	0.03126	HSP90AA1/
BP	GO:009031	regulation	5/905	36/18866	0.027584	0.059482	0.03126	CHP1/ITGB
BP	GO:010605	regulation	5/905	36/18866	0.027584	0.059482	0.03126	TNF/CHP1/
BP	GO:003223	regulation	10/905	104/18866	0.027585	0.059482	0.03126	NOX4/PIK3
BP	GO:000599	monosacch	22/905	296/18866	0.028353	0.061114	0.032118	RBP4/FABP
BP	GO:000611	regulation	6/905	49/18866	0.02893	0.062233	0.032706	ADIPOQ/FA
BP	GO:000759	lactation	6/905	49/18866	0.02893	0.062233	0.032706	VEGFA/VDI
BP	GO:004851	circadian b	6/905	49/18866	0.02893	0.062233	0.032706	PTGDS/CSF
BP	GO:006071	labyrinthin	6/905	49/18866	0.02893	0.062233	0.032706	SOCS3/IL10
BP	GO:009772	calcineurin	6/905	49/18866	0.02893	0.062233	0.032706	TNF/CHP1/
BP	GO:000762	locomotory	16/905	198/18866	0.029202	0.062793	0.033	CXCL12/IDO
BP	GO:002154	cerebellum	10/905	105/18866	0.029232	0.062807	0.033007	HSPA5/SEM
BP	GO:006201	negative re	10/905	105/18866	0.029232	0.062807	0.033007	TGFB1/ADI
BP	GO:000205	positive re	4/905	25/18866	0.029788	0.063799	0.033529	STAT1/VEG
BP	GO:003330	type B pan	4/905	25/18866	0.029788	0.063799	0.033529	WNT5A/GS
BP	GO:001482	vascular as	4/905	25/18866	0.029788	0.063799	0.033529	EDN3/EDN
BP	GO:003416	toll-like rec	4/905	25/18866	0.029788	0.063799	0.033529	TLR8/RSAD
BP	GO:005085	positive re	4/905	25/18866	0.029788	0.063799	0.033529	CCR7/CARD
BP	GO:006057	intestinal e	4/905	25/18866	0.029788	0.063799	0.033529	SRC/IL6ST/
BP	GO:006214	detection c	4/905	25/18866	0.029788	0.063799	0.033529	CXCL12/CX
BP	GO:200067	positive re	4/905	25/18866	0.029788	0.063799	0.033529	IFNG/TGFB
BP	GO:003090	midbrain d	9/905	91/18866	0.030348	0.064876	0.034095	HSPA5/S10
BP	GO:004247	odontogen	9/905	91/18866	0.030348	0.064876	0.034095	HDAC1/FG
BP	GO:004568	regulation	9/905	91/18866	0.030348	0.064876	0.034095	CTSL/TNF/
BP	GO:004847	oogenesis	9/905	91/18866	0.030348	0.064876	0.034095	SRC/PTX3/
BP	GO:004301	camera-typ	24/905	332/18866	0.03035	0.064876	0.034095	RBP4/VEGF
BP	GO:000692	cellular cor	5/905	37/18866	0.030677	0.065448	0.034396	ACVR1C/GI
BP	GO:003271	negative re	5/905	37/18866	0.030677	0.065448	0.034396	TMSB4X/A
BP	GO:004507	positive re	5/905	37/18866	0.030677	0.065448	0.034396	PPIA/CCL5/
BP	GO:005189	regulation	7/905	63/18866	0.030679	0.065448	0.034396	APOD/VEG
BP	GO:009010	regulation	7/905	63/18866	0.030679	0.065448	0.034396	APOD/VEG
BP	GO:000721	neuropepti	10/905	106/18866	0.030946	0.065907	0.034636	CYSLTR1/C
BP	GO:003241	positive re	10/905	106/18866	0.030946	0.065907	0.034636	HSPA2/IFN
BP	GO:004690	regulation	8/905	77/18866	0.030952	0.065907	0.034636	GSK3B/GZN
BP	GO:006133	renal tubul	8/905	77/18866	0.030952	0.065907	0.034636	VEGFA/AG
BP	GO:012025	olefinic cor	11/905	121/18866	0.030955	0.065907	0.034636	PTGDS/MA
BP	GO:000762	rhythmic b	6/905	50/18866	0.03159	0.067207	0.03532	PTGDS/CSF
BP	GO:004298	amyloid pr	6/905	50/18866	0.03159	0.067207	0.03532	IFNG/TNF/
BP	GO:000995	dorsal/ven	9/905	92/18866	0.032266	0.068575	0.036039	DLL4/GREN
BP	GO:001045	positive re	3/905	15/18866	0.0325	0.068575	0.036039	IL23A/IL12
BP	GO:002192	cell prolife	3/905	15/18866	0.0325	0.068575	0.036039	EGF/RORA/
BP	GO:002193	cerebellar	3/905	15/18866	0.0325	0.068575	0.036039	EGF/RORA/
BP	GO:003085	regulation	3/905	15/18866	0.0325	0.068575	0.036039	ADIPOQ/C1
BP	GO:003227	gonadotrop	3/905	15/18866	0.0325	0.068575	0.036039	INHA/INHB
BP	GO:003315	V(D)J recor	3/905	15/18866	0.0325	0.068575	0.036039	PRKDC/RA

BP	GO:003434	glial cell ap	3/905	15/18866	0.0325	0.068575	0.036039	CCL2/PRKC
BP	GO:004506	T-helper 2	3/905	15/18866	0.0325	0.068575	0.036039	TNFSF4/CD
BP	GO:004534	regulation	3/905	15/18866	0.0325	0.068575	0.036039	IFNG/IL10/
BP	GO:004547	locomotor	3/905	15/18866	0.0325	0.068575	0.036039	USP2/MTA
BP	GO:004583	negative re	3/905	15/18866	0.0325	0.068575	0.036039	MAPT/PTN
BP	GO:004848	enteric ner	3/905	15/18866	0.0325	0.068575	0.036039	EDNRA/ED
BP	GO:005086	positive re	3/905	15/18866	0.0325	0.068575	0.036039	CCR7/CARD
BP	GO:005505	neuroblast	3/905	15/18866	0.0325	0.068575	0.036039	WNT3A/AK
BP	GO:006034	heart trabe	3/905	15/18866	0.0325	0.068575	0.036039	RBP4/TEK/
BP	GO:006039	positive re	3/905	15/18866	0.0325	0.068575	0.036039	TGFB1/TGF
BP	GO:007256	chemokine	3/905	15/18866	0.0325	0.068575	0.036039	TNF/F2RL1
BP	GO:200034	regulation	3/905	15/18866	0.0325	0.068575	0.036039	TNF/F2RL1
BP	GO:200040	negative re	3/905	15/18866	0.0325	0.068575	0.036039	APOD/CCL2
BP	GO:200117	positive re	3/905	15/18866	0.0325	0.068575	0.036039	TMSB4X/PI
BP	GO:009723	cellular res	11/905	122/18866	0.03261	0.068781	0.036147	S100A9/DU
BP	GO:000001	regulation	10/905	107/18866	0.032731	0.068956	0.036239	TGFB1/TFR
BP	GO:004852	positive re	10/905	107/18866	0.032731	0.068956	0.036239	CD4/PPIA/
BP	GO:009917	regulation	10/905	107/18866	0.032731	0.068956	0.036239	WNT5A/PT
BP	GO:200004	regulation	15/905	185/18866	0.033099	0.069703	0.036632	PSME2/PM
BP	GO:000178	B cell apop	4/905	26/18866	0.033908	0.070938	0.03728	IL10/MIF/B
BP	GO:000222	innate imm	4/905	26/18866	0.033908	0.070938	0.03728	DEFB1/LTF
BP	GO:000317	atrioventri	4/905	26/18866	0.033908	0.070938	0.03728	BMP2/ZFPI
BP	GO:001943	removal of	4/905	26/18866	0.033908	0.070938	0.03728	TNF/MPO/
BP	GO:003081	positive re	4/905	26/18866	0.033908	0.070938	0.03728	TMSB4X/N
BP	GO:003631	cellular res	4/905	26/18866	0.033908	0.070938	0.03728	INHBA/ROI
BP	GO:004240	thyroid hor	4/905	26/18866	0.033908	0.070938	0.03728	CTSB/DUO
BP	GO:004356	regulation	4/905	26/18866	0.033908	0.070938	0.03728	BMP2/BMI
BP	GO:006034	trabecula f	4/905	26/18866	0.033908	0.070938	0.03728	RBP4/GREI
BP	GO:006039	regulation	4/905	26/18866	0.033908	0.070938	0.03728	TGFB1/GDI
BP	GO:006056	apoptotic p	4/905	26/18866	0.033908	0.070938	0.03728	VDR/JAG2/
BP	GO:007063	transepithe	4/905	26/18866	0.033908	0.070938	0.03728	EDNRB/CSI
BP	GO:007142	hematopoi	4/905	26/18866	0.033908	0.070938	0.03728	EIF2AK2/W
BP	GO:007227	metanephr	4/905	26/18866	0.033908	0.070938	0.03728	STAT1/PDG
BP	GO:009706	response t	4/905	26/18866	0.033908	0.070938	0.03728	CTSB/CTSL
BP	GO:190037	positive re	4/905	26/18866	0.033908	0.070938	0.03728	TMSB4X/N
BP	GO:190302	positive re	4/905	26/18866	0.033908	0.070938	0.03728	IL15/CCR7/
BP	GO:000329	physiologic	5/905	38/18866	0.033979	0.07095	0.037287	AGT/IGF1/
BP	GO:000330	physiologic	5/905	38/18866	0.033979	0.07095	0.037287	AGT/IGF1/
BP	GO:001943	triglyceride	5/905	38/18866	0.033979	0.07095	0.037287	FABP6/FAE
BP	GO:006104	cell growth	5/905	38/18866	0.033979	0.07095	0.037287	AGT/IGF1/
BP	GO:190589	positive re	5/905	38/18866	0.033979	0.07095	0.037287	PIK3R1/PM
BP	GO:007208	nephron tu	9/905	93/18866	0.034267	0.071523	0.037588	STAT1/VEG
BP	GO:000602	glycosamin	10/905	108/18866	0.034586	0.072162	0.037924	TGFB1/IL1I
BP	GO:000682	iron ion tra	8/905	79/18866	0.035343	0.073655	0.038708	B2M/IFNG
BP	GO:000749	endoderm	8/905	79/18866	0.035343	0.073655	0.038708	MMP9/ITG
BP	GO:003010	water hom	8/905	79/18866	0.035343	0.073655	0.038708	BPIFA1/CLI
BP	GO:000694	regulation	7/905	65/18866	0.035596	0.074124	0.038955	F2R/PTGS2
BP	GO:006067	ureteric bu	7/905	65/18866	0.035596	0.074124	0.038955	VEGFA/AG
BP	GO:001895	phenol-cor	10/905	109/18866	0.036514	0.076008	0.039945	CTSB/DUO
BP	GO:006042	lung morpl	6/905	52/18866	0.037376	0.077742	0.040856	TNF/MAPK

BP	GO:200067	regulation	6/905	52/18866	0.037376	0.077742	0.040856	IFNG/TMSE
BP	GO:000166	behavioral	5/905	39/18866	0.037493	0.077806	0.04089	MDK/UCN/
BP	GO:001073	protein kin	5/905	39/18866	0.037493	0.077806	0.04089	ADIPOQ/M
BP	GO:003314	regulation	5/905	39/18866	0.037493	0.077806	0.04089	SRC/AR/PA
BP	GO:003420	amyloid-be	5/905	39/18866	0.037493	0.077806	0.04089	IFNG/TNF/
BP	GO:007252	pyrimidine-	5/905	39/18866	0.037493	0.077806	0.04089	APOBEC3G
BP	GO:200027	negative re	5/905	39/18866	0.037493	0.077806	0.04089	ADIPOQ/SI
BP	GO:000758	respiratory	7/905	66/18866	0.038238	0.07912	0.04158	SFTPA2/CY
BP	GO:007217	mesonephr	7/905	66/18866	0.038238	0.07912	0.04158	VEGFA/AG
BP	GO:015011	regulation	7/905	66/18866	0.038238	0.07912	0.04158	APOD/VEG
BP	GO:000965	response to	4/905	27/18866	0.038343	0.07912	0.04158	HSP90AB1/
BP	GO:001099	response to	4/905	27/18866	0.038343	0.07912	0.04158	CXCL10/MI
BP	GO:003308	regulation	4/905	27/18866	0.038343	0.07912	0.04158	IL1B/IL7R/I
BP	GO:004250	serine pho:	4/905	27/18866	0.038343	0.07912	0.04158	IFNG/IFNE/
BP	GO:004642	negative re	4/905	27/18866	0.038343	0.07912	0.04158	SOCS3/SOC
BP	GO:004879	animal orga	4/905	27/18866	0.038343	0.07912	0.04158	LTF/BMP2/
BP	GO:009014	regulation	4/905	27/18866	0.038343	0.07912	0.04158	MAPT/PPA
BP	GO:190247	regulation	4/905	27/18866	0.038343	0.07912	0.04158	MAPT/WN
BP	GO:190331	positive reg	4/905	27/18866	0.038343	0.07912	0.04158	TFR2/SRC/
BP	GO:007200	nephron ep	10/905	110/18866	0.038515	0.07912	0.04158	ADIPOQ/ST
BP	GO:009719	execution p	9/905	95/18866	0.038521	0.07912	0.04158	ACVR1C/GI
BP	GO:000229	T-helper ce	3/905	16/18866	0.038609	0.07912	0.04158	IL23A/IL12
BP	GO:000322	ventricular	3/905	16/18866	0.038609	0.07912	0.04158	DLL4/NRG1
BP	GO:001091	regulation	3/905	16/18866	0.038609	0.07912	0.04158	PTK2B/ADC
BP	GO:001701	protein nitr	3/905	16/18866	0.038609	0.07912	0.04158	S100A9/S1
BP	GO:001811	peptidyl-cy	3/905	16/18866	0.038609	0.07912	0.04158	S100A9/S1
BP	GO:002154	dentate gy	3/905	16/18866	0.038609	0.07912	0.04158	MDK/NR2E
BP	GO:003222	regulation	3/905	16/18866	0.038609	0.07912	0.04158	PTGS2/GDI
BP	GO:003235	response to	3/905	16/18866	0.038609	0.07912	0.04158	INHBA/TGF
BP	GO:003586	response to	3/905	16/18866	0.038609	0.07912	0.04158	GNRH1/PP
BP	GO:005096	detection c	3/905	16/18866	0.038609	0.07912	0.04158	CXCL12/CX
BP	GO:005123	sequesterin	3/905	16/18866	0.038609	0.07912	0.04158	LCN2/S100
BP	GO:006057	intestinal e	3/905	16/18866	0.038609	0.07912	0.04158	SRC/IL6ST/
BP	GO:006154	ganglion de	3/905	16/18866	0.038609	0.07912	0.04158	SEMA3A/N
BP	GO:007088	positive reg	3/905	16/18866	0.038609	0.07912	0.04158	TNF/IGF1/I
BP	GO:007228	metanephr	3/905	16/18866	0.038609	0.07912	0.04158	STAT1/GDN
BP	GO:008610	G protein-c	3/905	16/18866	0.038609	0.07912	0.04158	SRC/NOS1/
BP	GO:010605	positive reg	3/905	16/18866	0.038609	0.07912	0.04158	TNF/IGF1/I
BP	GO:190012	negative re	3/905	16/18866	0.038609	0.07912	0.04158	B2M/ADIP
BP	GO:190372	regulation	3/905	16/18866	0.038609	0.07912	0.04158	S100A9/AF
BP	GO:000989	negative re	23/905	323/18866	0.038691	0.079258	0.041653	HSP90AB1/
BP	GO:009917	postsynaps	14/905	173/18866	0.039128	0.080124	0.042108	WNT5A/ND
BP	GO:004259	response to	16/905	206/18866	0.039838	0.081516	0.042839	HSPA5/EIF
BP	GO:190280	regulation	16/905	206/18866	0.039838	0.081516	0.042839	PSME2/PM
BP	GO:004394	regulation	6/905	53/18866	0.040506	0.082819	0.043525	GNAI1/NO
BP	GO:009917	presynapse	6/905	53/18866	0.040506	0.082819	0.043525	WNT5A/C5
BP	GO:190367	positive reg	7/905	67/18866	0.041007	0.083811	0.044046	S100A1/VE
BP	GO:000220	behavioral	5/905	40/18866	0.041222	0.08406	0.044177	MDK/UCN/
BP	GO:004390	positive reg	5/905	40/18866	0.041222	0.08406	0.044177	VEGFA/EDI
BP	GO:004574	positive reg	5/905	40/18866	0.041222	0.08406	0.044177	CACYBP/EC

BP	GO:00457	negative re	5/905	40/18866	0.041222	0.08406	0.044177	DLL4/ARRE
BP	GO:190274	apoptotic p	5/905	40/18866	0.041222	0.08406	0.044177	VDR/ROBO
BP	GO:190299	regulation	5/905	40/18866	0.041222	0.08406	0.044177	IFNG/TNF/
BP	GO:00324C	regulation	21/905	291/18866	0.041396	0.084384	0.044347	HSPA2/IFN
BP	GO:004358	ear develo	17/905	224/18866	0.042005	0.085593	0.044982	CXCL14/M/
BP	GO:190161	organic hyc	19/905	258/18866	0.042673	0.086921	0.04568	IFNG/NFYA
BP	GO:00218	cell prolifer	4/905	28/18866	0.043094	0.087514	0.045992	FABP7/WN
BP	GO:003295	inositol ph	4/905	28/18866	0.043094	0.087514	0.045992	PTK2B/PLC
BP	GO:003359	mammary	4/905	28/18866	0.043094	0.087514	0.045992	WNT5A/TN
BP	GO:003588	enteroend	4/905	28/18866	0.043094	0.087514	0.045992	WNT5A/GS
BP	GO:00443	cell-cell adl	4/905	28/18866	0.043094	0.087514	0.045992	VEGFA/WN
BP	GO:00457	positive re	4/905	28/18866	0.043094	0.087514	0.045992	NOS1/GRP
BP	GO:007145	cellular res	4/905	28/18866	0.043094	0.087514	0.045992	TNF/MPO/
BP	GO:007145	cellular res	4/905	28/18866	0.043094	0.087514	0.045992	TNF/MPO/
BP	GO:190301	regulation	6/905	54/18866	0.043797	0.08891	0.046725	IL15/PTX3/
BP	GO:004001	regulation	7/905	68/18866	0.043902	0.089089	0.046819	PIK3CA/GD
BP	GO:002289	regulation	20/905	276/18866	0.044104	0.089465	0.047017	HSPA2/IFN
BP	GO:190351	mucopolys	10/905	113/18866	0.044967	0.091182	0.04792	TGFB1/IL1
BP	GO:00031	pulmonary	3/905	17/18866	0.045257	0.09129	0.047976	ROBO2/JA
BP	GO:00092	mRNA tran	3/905	17/18866	0.045257	0.09129	0.047976	C5AR1/PP
BP	GO:001071	regulation	3/905	17/18866	0.045257	0.09129	0.047976	TGFB1/FG
BP	GO:00217	preganglio	3/905	17/18866	0.045257	0.09129	0.047976	SEMA3A/P
BP	GO:00307	sequesteri	3/905	17/18866	0.045257	0.09129	0.047976	IL1B/TNF/F
BP	GO:00336	cell-cell adl	3/905	17/18866	0.045257	0.09129	0.047976	CXCL13/CC
BP	GO:00457	positive re	3/905	17/18866	0.045257	0.09129	0.047976	PPARGC1A
BP	GO:005158	regulation	3/905	17/18866	0.045257	0.09129	0.047976	NOS1/GDN
BP	GO:00606	mammary	3/905	17/18866	0.045257	0.09129	0.047976	HIF1A/CEB
BP	GO:00611	3'-UTR-me	3/905	17/18866	0.045257	0.09129	0.047976	MOV10/DH
BP	GO:00702	renal abso	3/905	17/18866	0.045257	0.09129	0.047976	ADIPOQ/C
BP	GO:00901	negative re	3/905	17/18866	0.045257	0.09129	0.047976	MMP9/AD
BP	GO:19053	negative re	3/905	17/18866	0.045257	0.09129	0.047976	TNF/STAT1
BP	GO:20012	regulation	3/905	17/18866	0.045257	0.09129	0.047976	MMP9/PLA
BP	GO:00611	regulation	15/905	193/18866	0.045298	0.091332	0.047998	HSP90AB1,
BP	GO:00148	striated mu	8/905	83/18866	0.045345	0.091332	0.047998	RBP4/ANG
BP	GO:00480	focal adhes	8/905	83/18866	0.045345	0.091332	0.047998	APOD/VEG
BP	GO:20010	negative re	8/905	83/18866	0.045345	0.091332	0.047998	PSMD10/C
BP	GO:00447	DNA methy	9/905	98/18866	0.045552	0.091714	0.048199	APOBEC3G
BP	GO:000761	learning	12/905	145/18866	0.046385	0.093357	0.049062	TLR2/PTGS
BP	GO:00017	endoderm	6/905	55/18866	0.047251	0.094928	0.049888	MMP9/ITG
BP	GO:00032	ventricular	6/905	55/18866	0.047251	0.094928	0.049888	TGFB1/DLL
BP	GO:00083	glial cell mi	6/905	55/18866	0.047251	0.094928	0.049888	CCL2/CCL3
BP	GO:00220	telencepha	6/905	55/18866	0.047251	0.094928	0.049888	CXCL12/CX
BP	GO:00220	metenceph	10/905	114/18866	0.047271	0.094928	0.049888	HSPA5/SEN
BP	GO:003241	positive re	10/905	114/18866	0.047271	0.094928	0.049888	HSPA2/IFN
CC	GO:00098	external si	131/907	417/19559	2.88E-73	1.40E-70	1.13E-70	AZGP1/B2
CC	GO:00198	immunoglo	53/907	163/19559	1.09E-30	2.65E-28	2.14E-28	CD79A/IGH
CC	GO:00988	plasma me	66/907	307/19559	3.46E-26	5.62E-24	4.53E-24	B2M/CD4/
CC	GO:00425	immunoglo	34/907	76/19559	1.74E-25	2.12E-23	1.71E-23	IGHE/IGHG
CC	GO:00451	membrane	52/907	329/19559	7.15E-15	6.59E-13	5.31E-13	CD4/ICAM
CC	GO:00988	membrane	52/907	330/19559	8.12E-15	6.59E-13	5.31E-13	CD4/ICAM

CC	GO:00985€	membrane	52/907	343/19559	4.03E-14	2.81E-12	2.26E-12	CD4/ICAM:
CC	GO:00425€	specific gra	33/907	160/19559	3.53E-13	2.12E-11	1.71E-11	B2M/SLPI/
CC	GO:00319€	vesicle lum	49/907	328/19559	3.92E-13	2.12E-11	1.71E-11	B2M/HSP9
CC	GO:00602(	cytoplasmi	48/907	326/19559	1.18E-12	5.74E-11	4.63E-11	B2M/HSP9
CC	GO:00306€	secretory g	46/907	306/19559	1.66E-12	7.35E-11	5.93E-11	FCER1G/HL
CC	GO:00347	secretory g	47/907	322/19559	2.79E-12	1.13E-10	9.14E-11	B2M/HSP9
CC	GO:00421(	T cell recep	29/907	140/19559	8.50E-12	3.18E-10	2.57E-10	CD4/PTPN6
CC	GO:00708	tertiary gra	31/907	164/19559	2.01E-11	6.98E-10	5.63E-10	B2M/FCER
CC	GO:00725€	blood micr	29/907	148/19559	3.58E-11	1.16E-09	9.37E-10	HSPA2/TGI
CC	GO:00301	endocytic \	44/907	313/19559	4.98E-11	1.52E-09	1.22E-09	B2M/CALR
CC	GO:00620	collagen-cc	53/907	427/19559	6.49E-11	1.86E-09	1.50E-09	AZGP1/CAI
CC	GO:00226	proteasom	12/907	24/19559	1.49E-10	4.02E-09	3.24E-09	PSMC2/PSI
CC	GO:00453	phagocytic	27/907	140/19559	2.47E-10	6.33E-09	5.11E-09	B2M/CALR
CC	GO:00058	proteasom	11/907	21/19559	4.62E-10	1.13E-08	9.07E-09	PSMC2/PSI
CC	GO:00708	tertiary gra	19/907	73/19559	5.61E-10	1.30E-08	1.05E-08	FCER1G/PL
CC	GO:00426	MHC prote	11/907	25/19559	4.92E-09	1.09E-07	8.79E-08	B2M/HLA-/
CC	GO:00306	phagocytic	18/907	77/19559	1.02E-08	2.17E-07	1.75E-07	B2M/CALR
CC	GO:00057€	early endo:	44/907	377/19559	1.82E-08	3.70E-07	2.98E-07	B2M/CD1E
CC	GO:00306€	endocytic \	26/907	164/19559	3.87E-08	7.53E-07	6.07E-07	B2M/CALR
CC	GO:00355€	specific gra	15/907	62/19559	1.06E-07	1.98E-06	1.60E-06	B2M/SLPI/
CC	GO:00017	immunolog	12/907	39/19559	1.13E-07	2.03E-06	1.64E-06	ICAM1/VA\
CC	GO:00355	specific gra	18/907	91/19559	1.60E-07	2.79E-06	2.25E-06	PLAU/CYBE
CC	GO:00005(	proteasom	14/907	63/19559	8.61E-07	1.45E-05	1.17E-05	PSMB8/PSI
CC	GO:00716€	endocytic \	8/907	20/19559	1.59E-06	2.58E-05	2.08E-05	CALR/HSP9
CC	GO:00059	focal adhe:	42/907	415/19559	1.86E-06	2.92E-05	2.36E-05	B2M/CALR
CC	GO:00057€	endoplasm	34/907	308/19559	2.68E-06	4.06E-05	3.27E-05	B2M/CALR
CC	GO:19053€	endopeptic	14/907	69/19559	2.75E-06	4.06E-05	3.27E-05	PSMB8/PSI
CC	GO:00300	cell-substra	42/907	423/19559	3.03E-06	4.34E-05	3.50E-05	B2M/CALR
CC	GO:00021	semaphori	6/907	11/19559	3.70E-06	5.15E-05	4.15E-05	PLXNA1/PL
CC	GO:00059	phosphatic	9/907	29/19559	4.12E-06	5.28E-05	4.26E-05	SOCS3/SO(
CC	GO:00715	integral coı	9/907	29/19559	4.12E-06	5.28E-05	4.26E-05	CALR/HLA-
CC	GO:00985	lumenal sic	9/907	29/19559	4.12E-06	5.28E-05	4.26E-05	CALR/HLA-
CC	GO:00617(	inflammas	7/907	17/19559	5.82E-06	7.27E-05	5.86E-05	CASP1/DH)
CC	GO:00085	proteasom	6/907	12/19559	7.11E-06	8.65E-05	6.97E-05	PSMC2/PSI
CC	GO:00306€	coated ves	23/907	183/19559	1.37E-05	0.000163	0.000131	B2M/CD4/
CC	GO:00057	vacuolar lu	22/907	173/19559	1.74E-05	0.000201	0.000162	CD1E/CTSE
CC	GO:00301	coated ves	31/907	298/19559	2.39E-05	0.00027	0.000218	B2M/CD4/
CC	GO:00985	lumenal sic	9/907	36/19559	2.89E-05	0.00032	0.000258	CALR/HLA-
CC	GO:00310	platelet alp	12/907	67/19559	5.23E-05	0.000566	0.000456	TMSB4X/T(
CC	GO:00448	plasma me	16/907	113/19559	6.52E-05	0.000691	0.000557	F2R/MAPK
CC	GO:00550	recycling e	22/907	190/19559	7.39E-05	0.000765	0.000617	B2M/PDIA:
CC	GO:00356	CD40 rece	5/907	11/19559	7.75E-05	0.000784	0.000632	CD40/TRAI
CC	GO:00057	Golgi lume	15/907	103/19559	7.89E-05	0.000784	0.000632	DEFB1/TGI
CC	GO:19053€	peptidase (	14/907	92/19559	8.33E-05	0.000811	0.000654	PSMB8/PSI
CC	GO:00059(	caveola	13/907	82/19559	9.64E-05	0.00092	0.000742	F2R/MAPK
CC	GO:01010(	ficolin-1-ric	11/907	61/19559	9.99E-05	0.000936	0.000754	FCER1G/SL
CC	GO:00057	Golgi-asso	21/907	181/19559	0.000104	0.000953	0.000768	B2M/HLA-/
CC	GO:00306€	clathrin-co	16/907	118/19559	0.00011	0.000996	0.000803	CD4/HLA-D
CC	GO:00430	NADPH oxi	5/907	12/19559	0.000128	0.001132	0.000913	NOX4/DUC
CC	GO:00319(	early endo:	19/907	161/19559	0.000174	0.001515	0.001222	B2M/HLA-/

CC	GO:190472	tertiary gra	10/907	55/19559	0.000191	0.001635	0.001318	B2M/PGLY
CC	GO:009863	protein cor	8/907	36/19559	0.000197	0.001654	0.001333	ITGAV/ITGI
CC	GO:001989	extrinsic cc	29/907	306/19559	0.000223	0.001842	0.001485	DEFB1/S10
CC	GO:003013	clathrin-co	21/907	194/19559	0.000275	0.002229	0.001797	CD4/HLA-D
CC	GO:003109	platelet alp	13/907	91/19559	0.000282	0.002253	0.001816	TMSB4X/T(
CC	GO:004262	mast cell g	6/907	22/19559	0.000385	0.002975	0.002399	BTK/PIK3CI
CC	GO:009703	perinuclea	6/907	22/19559	0.000385	0.002975	0.002399	NOX4/CYBI
CC	GO:004320	lysosomal l	13/907	96/19559	0.00048	0.003654	0.002946	CD1E/CTSE
CC	GO:001250	ER to Golgi	10/907	62/19559	0.000525	0.003934	0.003171	B2M/HLA-/
CC	GO:005503	recycling e	12/907	85/19559	0.000534	0.003942	0.003178	B2M/PDIA3
CC	GO:003066	Golgi-assoc	14/907	112/19559	0.000675	0.004905	0.003954	B2M/HLA-/
CC	GO:003190	endosome	7/907	35/19559	0.000969	0.006943	0.005598	B2M/CTSB,
CC	GO:003125	cell leading	34/907	421/19559	0.001257	0.008873	0.007154	S100B/S10
CC	GO:003601	endolysosc	5/907	20/19559	0.001836	0.012774	0.010299	CTSB/CTSL,
CC	GO:000830	integrin co	6/907	31/19559	0.00266	0.018244	0.014709	ITGAV/ITGI
CC	GO:000172	ruffle	17/907	179/19559	0.004088	0.027652	0.022294	S100B/S10
CC	GO:010100	ficolin-1-ric	13/907	124/19559	0.004992	0.03285	0.026484	CTSB/HSP9
CC	GO:190481	ficolin-1-ric	13/907	124/19559	0.004992	0.03285	0.026484	CTSB/HSP9
CC	GO:007143	invadopodi	4/907	16/19559	0.005345	0.034344	0.027689	PLAUR/PAK
CC	GO:003013	transport v	31/907	410/19559	0.00536	0.034344	0.027689	B2M/HLA-/
CC	GO:001632	apical plasr	28/907	361/19559	0.005626	0.03558	0.028685	CTSB/CTSL,
CC	GO:000566	transcriptic	31/907	413/19559	0.005943	0.037107	0.029917	NFYA/RELB
CC	GO:009856	cytoplasmi	17/907	188/19559	0.00666	0.041054	0.033099	S100A6/GN
CC	GO:000577	multivesicu	8/907	62/19559	0.007628	0.045949	0.037045	CTSL/SFTP/
CC	GO:001660	PML body	11/907	102/19559	0.007642	0.045949	0.037045	PML/ISG20
CC	GO:009057	RNA polym	15/907	161/19559	0.008007	0.047553	0.038338	NFYA/PPAF
CC	GO:190255	serine/thre	10/907	89/19559	0.008104	0.047553	0.038338	IKBKG/ACV
CC	GO:003152	filopodium	4/907	18/19559	0.008348	0.0484	0.039021	HLA-G/ITG/
CC	GO:190291	protein kin	11/907	104/19559	0.008807	0.050456	0.040679	IKBKG/ACV
CC	GO:000564	nuclear env	3/907	10/19559	0.009335	0.052739	0.042519	CACYBP/IG
CC	GO:000989	cytoplasmi	15/907	164/19559	0.009422	0.052739	0.042519	S100A6/GN
CC	GO:000577	late endosc	22/907	278/19559	0.010533	0.058291	0.046996	CD1E/CTSL
MF	GO:004801	receptor lig	177/879	487/18352	2.42E-110	1.85E-107	1.37E-107	IFNG/LTA/(
MF	GO:003054	signaling re	177/879	492/18352	1.87E-109	7.13E-107	5.29E-107	IFNG/LTA/(
MF	GO:000512	cytokine re	110/879	271/18352	1.15E-73	2.94E-71	2.18E-71	IFNG/LTA/I
MF	GO:000512	cytokine ac	103/879	235/18352	5.02E-73	9.59E-71	7.11E-71	IFNG/LTA/(
MF	GO:000808	growth fac	68/879	162/18352	9.77E-47	1.49E-44	1.11E-44	CXCL12/TG
MF	GO:000382	antigen bir	69/879	168/18352	1.21E-46	1.54E-44	1.14E-44	CD1C/CD1I
MF	GO:001995	cytokine bi	55/879	135/18352	3.98E-37	3.80E-35	2.82E-35	CD4/CCR1(
MF	GO:014037	immune re	55/879	135/18352	3.98E-37	3.80E-35	2.82E-35	CD4/FCER1
MF	GO:000489	cytokine re	47/879	97/18352	3.58E-36	3.04E-34	2.25E-34	CD4/CCR1(
MF	GO:007085	growth fac	48/879	141/18352	2.48E-28	1.90E-26	1.41E-26	ERAP1/IL1I
MF	GO:000800	chemokine	28/879	49/18352	1.10E-24	7.65E-23	5.67E-23	CXCL14/CX
MF	GO:003498	immunoglc	34/879	80/18352	3.90E-24	2.48E-22	1.84E-22	IGHE/IGHG
MF	GO:004237	chemokine	32/879	70/18352	5.20E-24	3.06E-22	2.27E-22	CXCL14/CX
MF	GO:003281	tumor necr	26/879	48/18352	3.41E-22	1.86E-20	1.38E-20	LTA/TNF/ST
MF	GO:000516	tumor necr	21/879	31/18352	4.32E-21	2.20E-19	1.63E-19	LTA/TNF/ST
MF	GO:000166	G protein-c	58/879	293/18352	1.30E-20	6.21E-19	4.61E-19	CXCL14/CX
MF	GO:001983	growth fac	35/879	136/18352	1.23E-16	5.53E-15	4.10E-15	CXCL13/IT(
MF	GO:000852	G protein-c	36/879	148/18352	3.17E-16	1.35E-14	9.98E-15	CCR10/F2R

MF	GO:00016	peptide rec	36/879	152/18352	7.89E-16	3.17E-14	2.35E-14	CCR10/F2R
MF	GO:004802	CCR chemo	20/879	47/18352	9.35E-15	3.57E-13	2.65E-13	CXCL13/XC
MF	GO:000517	hormone a	31/879	122/18352	1.01E-14	3.67E-13	2.72E-13	ADIPOQ/C(
MF	GO:001995	C-C chemo	15/879	24/18352	1.24E-14	4.32E-13	3.20E-13	CCR10/CX(
MF	GO:001995	chemokine	17/879	33/18352	1.79E-14	5.94E-13	4.40E-13	CCR10/ITG
MF	GO:000163	G protein-c	15/879	26/18352	6.72E-14	2.05E-12	1.52E-12	CCR10/CX(
MF	GO:000495	chemokine	15/879	26/18352	6.72E-14	2.05E-12	1.52E-12	CCR10/CX(
MF	GO:000487	nuclear rec	20/879	52/18352	9.61E-14	2.72E-12	2.01E-12	PPARG/VD
MF	GO:009853	ligand-activ	20/879	52/18352	9.61E-14	2.72E-12	2.01E-12	PPARG/VD
MF	GO:001649	C-C chemo	14/879	23/18352	1.65E-13	4.50E-12	3.34E-12	CCR10/CX(
MF	GO:000471	protein tyr	30/879	135/18352	1.21E-12	3.18E-11	2.36E-11	EIF2AK2/T
MF	GO:004205	chemoattra	16/879	37/18352	3.35E-12	8.53E-11	6.33E-11	CXCL10/VE
MF	GO:004227	peptide bir	46/879	308/18352	6.17E-12	1.52E-10	1.13E-10	CALR/CD1(
MF	GO:001919	transmeml	20/879	80/18352	7.54E-10	1.80E-08	1.33E-08	HJV/PDGFI
MF	GO:199078	protein tyr	22/879	98/18352	9.95E-10	2.30E-08	1.71E-08	CD4/HSP9(
MF	GO:000372	double-stra	19/879	76/18352	2.00E-09	4.45E-08	3.30E-08	HSP90AB1/
MF	GO:004438	ubiquitin-li	42/879	316/18352	2.04E-09	4.45E-08	3.30E-08	CALR/HSPA
MF	GO:003321	amide binc	47/879	381/18352	2.67E-09	5.67E-08	4.20E-08	CALR/CD1(
MF	GO:000517	integrin bir	26/879	144/18352	4.51E-09	9.15E-08	6.78E-08	CALR/ICAN
MF	GO:000178	phosphoty	14/879	42/18352	4.55E-09	9.15E-08	6.78E-08	SOCS3/MA
MF	GO:000514	interleukin	9/879	16/18352	1.08E-08	2.07E-07	1.53E-07	ERAP1/IL1I
MF	GO:003162	ubiquitin p	39/879	297/18352	1.08E-08	2.07E-07	1.53E-07	CALR/HSPA
MF	GO:004530	protein ph	15/879	53/18352	1.61E-08	3.00E-07	2.23E-07	SOCS3/MA
MF	GO:000471	non-memb	14/879	46/18352	1.73E-08	3.14E-07	2.33E-07	EIF2AK2/T
MF	GO:004549	chemorepe	11/879	28/18352	2.90E-08	5.15E-07	3.82E-07	SEMA3A/S
MF	GO:003021	semaphori	10/879	23/18352	3.91E-08	6.64E-07	4.92E-07	SEMA3A/S
MF	GO:003818	pattern rec	10/879	23/18352	3.91E-08	6.64E-07	4.92E-07	PGLYRP1/P
MF	GO:004523	CXCR chen	9/879	18/18352	4.19E-08	6.96E-07	5.16E-07	CXCL8/CX(
MF	GO:004260	peptide an	11/879	31/18352	1.00E-07	1.63E-06	1.21E-06	HLA-A/HLA
MF	GO:000370	steroid hor	10/879	26/18352	1.59E-07	2.53E-06	1.88E-06	ESR2/ESRR
MF	GO:001502	coreceptor	13/879	48/18352	2.59E-07	4.04E-06	2.99E-06	CD4/HJV/I
MF	GO:000515	epidermal	11/879	34/18352	2.97E-07	4.53E-06	3.36E-06	PLSCR1/VA
MF	GO:001715	semaphori	7/879	12/18352	3.62E-07	5.42E-06	4.02E-06	PLXNA1/PL
MF	GO:004256	hormone b	17/879	84/18352	3.90E-07	5.73E-06	4.25E-06	CALR/PIK3I
MF	GO:005121	phosphopr	17/879	85/18352	4.66E-07	6.72E-06	4.98E-06	SOCS3/MA
MF	GO:001920	kinase regu	29/879	216/18352	5.15E-07	7.28E-06	5.40E-06	HSP90AB1/
MF	GO:003361	receptor se	10/879	29/18352	5.26E-07	7.31E-06	5.42E-06	SRC/BMP2
MF	GO:007070	BMP recep	7/879	13/18352	7.52E-07	1.01E-05	7.51E-06	SRC/BMP2
MF	GO:004354	phosphatic	10/879	30/18352	7.56E-07	1.01E-05	7.51E-06	PDGFRB/PI
MF	GO:000471	transmeml	14/879	61/18352	8.29E-07	1.09E-05	8.09E-06	PDGFRB/E(
MF	GO:007069	transmeml	9/879	24/18352	8.69E-07	1.12E-05	8.34E-06	SRC/BMP2
MF	GO:000161	virus recep	15/879	74/18352	1.83E-06	2.30E-05	1.70E-05	CD4/ICAM
MF	GO:014027	exogenous	15/879	74/18352	1.83E-06	2.30E-05	1.70E-05	CD4/ICAM
MF	GO:000153	lipopolysac	10/879	33/18352	2.04E-06	2.52E-05	1.87E-05	TLR2/LBP/I
MF	GO:000503	tumor necr	6/879	10/18352	2.11E-06	2.56E-05	1.90E-05	TNFRSF11/
MF	GO:004693	1-phospha	7/879	16/18352	4.42E-06	5.27E-05	3.91E-05	SOCS3/SO(
MF	GO:000553	glycosamin	28/879	232/18352	6.67E-06	7.80E-05	5.78E-05	CXCL10/CX
MF	GO:003097	receptor ty	14/879	72/18352	6.74E-06	7.80E-05	5.78E-05	BLNK/PIK3I
MF	GO:003802	cargo rece	14/879	73/18352	7.97E-06	9.09E-05	6.74E-05	TFRC/TFR2
MF	GO:000503	death rece	6/879	12/18352	8.55E-06	9.34E-05	6.92E-05	TNFRSF11/

MF	GO:000516	neurotroph	6/879	12/18352	8.55E-06	9.34E-05	6.92E-05	PIK3R1/NG
MF	GO:003532	Toll-like re	6/879	12/18352	8.55E-06	9.34E-05	6.92E-05	S100A9/S1
MF	GO:001617	superoxide	6/879	13/18352	1.52E-05	0.000164	0.000122	NOX4/DUC
MF	GO:000512	death rece	7/879	20/18352	2.53E-05	0.000264	0.000196	FASLG/NGI
MF	GO:003501	phosphatic	7/879	20/18352	2.53E-05	0.000264	0.000196	SOCS3/SOC
MF	GO:000517	vascular er	6/879	14/18352	2.56E-05	0.000264	0.000196	VEGFA/VEC
MF	GO:001988	protein kin	23/879	185/18352	2.70E-05	0.000275	0.000204	HSP90AB1,
MF	GO:000202	protease b	19/879	137/18352	2.92E-05	0.000293	0.000217	ADRM1/LC
MF	GO:000462	phospholip	8/879	28/18352	3.54E-05	0.000351	0.00026	PDIA3/CCL
MF	GO:005142	hormone r	22/879	177/18352	4.02E-05	0.000391	0.00029	CALR/OASL
MF	GO:000495	icosanoid r	6/879	15/18352	4.09E-05	0.000391	0.00029	PPARG/PTC
MF	GO:000516	platelet-de	6/879	15/18352	4.09E-05	0.000391	0.00029	VEGFA/PDC
MF	GO:000549	steroid bin	16/879	106/18352	4.31E-05	0.000407	0.000302	PDIA2/CET
MF	GO:000122	transcriptic	8/879	29/18352	4.69E-05	0.000437	0.000324	AR/NR4A3,
MF	GO:004830	calcium-de	14/879	85/18352	4.81E-05	0.000442	0.000328	S100A9/S1
MF	GO:007172	lipopeptide	5/879	10/18352	5.13E-05	0.000467	0.000346	CD1C/CD1I
MF	GO:000820	heparin bir	21/879	169/18352	6.00E-05	0.000539	0.0004	CXCL10/CX
MF	GO:000518	neuropepti	8/879	30/18352	6.12E-05	0.000539	0.0004	CORT/GRP,
MF	GO:000395	NAD+ nucl	6/879	16/18352	6.28E-05	0.000539	0.0004	TLR2/IL18F
MF	GO:005013	NAD(P)+ nu	6/879	16/18352	6.28E-05	0.000539	0.0004	TLR2/IL18F
MF	GO:006180	NAD+ nucl	6/879	16/18352	6.28E-05	0.000539	0.0004	TLR2/IL18F
MF	GO:000550	fatty acid b	9/879	39/18352	7.37E-05	0.000625	0.000463	S100A9/S1
MF	GO:001986	IgG binding	5/879	11/18352	9.04E-05	0.000759	0.000563	FCER1G/FC
MF	GO:000516	transformi	7/879	24/18352	9.54E-05	0.000784	0.000581	TGFB1/AM
MF	GO:001986	immunoglo	7/879	24/18352	9.54E-05	0.000784	0.000581	CD4/FCER1
MF	GO:000551	collagen bi	12/879	70/18352	0.000111	0.000899	0.000666	CTSB/CTSL,
MF	GO:002302	MHC prote	7/879	25/18352	0.000127	0.001022	0.000757	HLA-DMB/
MF	GO:001704	peptide ho	10/879	51/18352	0.000129	0.001025	0.00076	PIK3R1/ED
MF	GO:004283	peptidogly	6/879	18/18352	0.000134	0.001056	0.000783	PGLYRP1/P
MF	GO:008972	caspase bir	5/879	12/18352	0.000149	0.001161	0.00086	RIPK2/NLR
MF	GO:000443	phosphatic	7/879	26/18352	0.000167	0.001287	0.000954	CCL5/CCR5
MF	GO:000467	transmemb	6/879	19/18352	0.000188	0.001423	0.001055	HJV/LTBP1,
MF	GO:005066	oxidoreduc	6/879	19/18352	0.000188	0.001423	0.001055	NOX4/DUC
MF	GO:003970	co-recepto	5/879	13/18352	0.000232	0.001741	0.00129	TFR2/BMP
MF	GO:000154	amyloid-be	12/879	77/18352	0.000281	0.002065	0.001531	TLR2/MSR1
MF	GO:001990	protein ph	18/879	149/18352	0.000281	0.002065	0.001531	STAT1/MA
MF	GO:005084	extracellula	10/879	57/18352	0.000336	0.002444	0.001812	TINAGL1/V
MF	GO:005142	peptide ho	6/879	21/18352	0.000346	0.00248	0.001838	GNRH1/PT
MF	GO:004454	S100 prote	5/879	14/18352	0.000347	0.00248	0.001838	S100B/S10
MF	GO:003369	sialic acid b	6/879	22/18352	0.000457	0.003235	0.002398	ADIPOQ/CI
MF	GO:006162	RNA polym	26/879	267/18352	0.000492	0.003445	0.002553	CALR/OASL
MF	GO:000184	opsonin bir	5/879	15/18352	0.0005	0.003445	0.002553	CALR/ITGA
MF	GO:003604	long-chain	5/879	15/18352	0.0005	0.003445	0.002553	S100A9/S1
MF	GO:001650	peptidase a	8/879	40/18352	0.000526	0.003589	0.00266	PSME2/PSI
MF	GO:000515	insulin rece	6/879	23/18352	0.000594	0.003948	0.002926	SRC/PIK3R
MF	GO:005043	transformi	6/879	23/18352	0.000594	0.003948	0.002926	ITGAV/LTB
MF	GO:003329	monocarbo	11/879	72/18352	0.000594	0.003948	0.002926	S100A9/S1
MF	GO:004216	SH2 domai	8/879	41/18352	0.000627	0.004126	0.003059	SRC/PTK2/
MF	GO:000122	transcriptic	9/879	51/18352	0.000632	0.004126	0.003059	HDGF/AR/I
MF	GO:006113	peptidase r	23/879	229/18352	0.000664	0.004298	0.003186	PSME2/PSI

MF	GO:000515	insulin-like	5/879	16/18352	0.000699	0.004416	0.003273	SOCS1/ARI
MF	GO:000803	high-densit	5/879	16/18352	0.000699	0.004416	0.003273	ABCA1/SCA
MF	GO:005070	CARD dom	5/879	16/18352	0.000699	0.004416	0.003273	CARD11/CA
MF	GO:004228	MHC prote	8/879	42/18352	0.000742	0.004646	0.003444	CD4/KLRD1
MF	GO:007185	neuropepti	7/879	33/18352	0.000808	0.005018	0.00372	EDN3/APLN
MF	GO:000495	prostaglan	4/879	10/18352	0.00087	0.005362	0.003975	PPARG/PTG
MF	GO:003418	apolipopro	5/879	17/18352	0.000952	0.005819	0.004313	MAPT/ABC
MF	GO:007181	lipoprotein	7/879	34/18352	0.000976	0.005871	0.004352	MAPT/MSR
MF	GO:007181	protein-lipi	7/879	34/18352	0.000976	0.005871	0.004352	MAPT/MSR
MF	GO:001990	phosphatas	20/879	194/18352	0.001052	0.006281	0.004656	STAT1/MA
MF	GO:000203	p53 bindin	10/879	66/18352	0.001113	0.006593	0.004887	DAXX/HDA
MF	GO:000495	prostanoid	4/879	11/18352	0.001316	0.007617	0.005646	PPARG/PTG
MF	GO:003199	thioesteras	4/879	11/18352	0.001316	0.007617	0.005646	TRAF3/TRA
MF	GO:005078	RAGE recep	4/879	11/18352	0.001316	0.007617	0.005646	S100A9/S1
MF	GO:000196	fibronectin	6/879	27/18352	0.001478	0.008491	0.006294	CTSL/VEGF
MF	GO:003029	protein tyr	5/879	19/18352	0.001652	0.009418	0.006981	CCL5/EGF/
MF	GO:003503	histone ace	6/879	28/18352	0.001806	0.010221	0.007576	STAT1/NR4
MF	GO:000412	cytidine de	4/879	12/18352	0.0019	0.010593	0.007852	APOBEC3G
MF	GO:000487	compleme	4/879	12/18352	0.0019	0.010593	0.007852	C5AR1/CM
MF	GO:003024	carbohydra	24/879	267/18352	0.002363	0.01308	0.009696	CALR/KLRC
MF	GO:004317	alcohol bin	11/879	86/18352	0.002623	0.014307	0.010605	RBP1/RBP4
MF	GO:000502	transformi	4/879	13/18352	0.00264	0.014307	0.010605	LTBP1/BMI
MF	GO:003612	BMP bindir	4/879	13/18352	0.00264	0.014307	0.010605	HJV/GDF5/
MF	GO:000184	compleme	5/879	21/18352	0.00267	0.014363	0.010646	CALR/PTX3
MF	GO:004288	amide tran	8/879	51/18352	0.002739	0.014634	0.010847	AZGP1/TAF
MF	GO:190468	peptide tra	6/879	31/18352	0.003123	0.016572	0.012284	AZGP1/TAF
MF	GO:001692	nuclear rec	12/879	101/18352	0.003203	0.016875	0.012509	CALR/SRC/
MF	GO:000467	protein ser	34/879	435/18352	0.003573	0.018695	0.013857	HJV/EIF2AI
MF	GO:003525	nuclear hor	15/879	144/18352	0.003841	0.01994	0.01478	CALR/OASL
MF	GO:190168	sulfur com	23/879	262/18352	0.003863	0.01994	0.01478	CXCL10/CX
MF	GO:004302	cysteine-ty	7/879	43/18352	0.004023	0.02063	0.015292	ARRB1/BIR
MF	GO:004633	SMAD bind	10/879	79/18352	0.004353	0.022173	0.016436	PML/FOS/E
MF	GO:003525	glucocortic	4/879	15/18352	0.004669	0.02347	0.017397	NR4A1/NR
MF	GO:009715	cysteine-ty	4/879	15/18352	0.004669	0.02347	0.017397	CASP1/PYC
MF	GO:001679	hydrolase a	6/879	34/18352	0.005051	0.025089	0.018597	TLR2/IL18R
MF	GO:014029	DNA-bindir	28/879	347/18352	0.00506	0.025089	0.018597	CALR/OASL
MF	GO:006113	endopeptic	18/879	192/18352	0.00509	0.025089	0.018597	PSME2/PSI
MF	GO:003525	steroid hor	10/879	81/18352	0.005211	0.025523	0.018919	CALR/SRC/
MF	GO:003029	protein kin	10/879	82/18352	0.005687	0.027676	0.020515	LTF/CCL5/I
MF	GO:000510	fibroblast g	5/879	25/18352	0.005948	0.028763	0.02132	FGF10/FGF
MF	GO:002302	MHC class	4/879	16/18352	0.005993	0.028796	0.021345	HLA-DMB/
MF	GO:003140	carboxylic a	19/879	212/18352	0.006583	0.031435	0.023302	S100A9/S1
MF	GO:000504	scavenger	7/879	47/18352	0.006658	0.031593	0.023418	MSR1/DMI
MF	GO:000550	retinoid bir	6/879	36/18352	0.006746	0.031814	0.023582	PTGDS/RBF
MF	GO:003293	sterol bind	8/879	59/18352	0.006823	0.031979	0.023705	CETP/PMP
MF	GO:003016	low-densit	4/879	17/18352	0.007544	0.035143	0.02605	MSR1/SCA
MF	GO:001984	isoprenoid	6/879	37/18352	0.007733	0.035804	0.02654	PTGDS/RBF
MF	GO:000470	MAP kinas	4/879	18/18352	0.009337	0.042717	0.031664	MAPK3/PA
MF	GO:004696	retinoid X r	4/879	18/18352	0.009337	0.042717	0.031664	PPARG/VD
MF	GO:001920	kinase acti	10/879	89/18352	0.010036	0.045579	0.033786	LTF/CCL5/I

MF	GO:001712	SH3 domai	13/879	131/18352	0.010143	0.045579	0.033786	MAPT/PLS(
MF	GO:003631	phosphatic	3/879	10/18352	0.010202	0.045579	0.033786	PIK3R1/CB
MF	GO:00435(	insulin rece	3/879	10/18352	0.010202	0.045579	0.033786	PIK3R1/PIK
MF	GO:00081∠	drug bindir	11/879	104/18352	0.011037	0.049025	0.03634	HSP90AB1/
MF	GO:00432З	laminin bin	5/879	29/18352	0.011357	0.050153	0.037176	TINAGL1/IT
MF	GO:00431	organic aci	19/879	224/18352	0.011531	0.050629	0.037528	S100A9/S1
MF	GO:004698	protein het	25/879	321/18352	0.011862	0.051786	0.038386	CYBB/IKBK
MF	GO:00048(	protein kin	8/879	65/18352	0.012095	0.052505	0.03892	SOCS3/SO(
MF	GO:00484(	platelet-de	3/879	11/18352	0.013534	0.058417	0.043302	PDGFRB/PI
MF	GO:00316	adrenergic	4/879	20/18352	0.013704	0.058821	0.043601	ARRB1/AD

ID	Descriptior	GeneRatio	BgRatio	pvalue	p.adjust	qvalue	geneID	Count
hsa04060	Cytokine-c	144/643	295/8096	6.07E-83	1.54E-80	6.78E-81	CD4/IFNG/	144
hsa04061	Viral protei	58/643	100/8096	1.69E-38	2.15E-36	9.44E-37	LTA/CXCL1	58
hsa05169	Epstein-Ba	74/643	202/8096	1.51E-31	1.28E-29	5.63E-30	B2M/CALR	74
hsa04062	Chemokine	71/643	192/8096	1.39E-30	8.83E-29	3.88E-29	CXCL14/CX	71
hsa04650	Natural kill	55/643	131/8096	4.20E-27	2.14E-25	9.38E-26	FCER1G/HI	55
hsa04630	JAK-STAT s	59/643	162/8096	4.12E-25	1.74E-23	7.65E-24	IFNG/IFNL1	59
hsa05417	Lipid and a	66/643	215/8096	3.73E-23	1.35E-21	5.95E-22	HSPA2/HSI	66
hsa05323	Rheumatoi	40/643	93/8096	1.88E-20	5.96E-19	2.62E-19	CTSL/HLA-I	40
hsa04620	Toll-like re	42/643	104/8096	3.75E-20	1.06E-18	4.65E-19	CXCL8/CXC	42
hsa05163	Human cyt	63/643	225/8096	8.30E-20	2.11E-18	9.26E-19	B2M/CALR	63
hsa05164	Influenza A	54/643	172/8096	1.34E-19	2.87E-18	1.26E-18	HLA-DMB/	54
hsa04668	TNF signali	43/643	112/8096	1.35E-19	2.87E-18	1.26E-18	ICAM1/LTA	43
hsa04064	NF-kappa E	41/643	104/8096	3.09E-19	6.03E-18	2.65E-18	ICAM1/LTA	41
hsa05167	Kaposi sarc	57/643	194/8096	4.31E-19	7.82E-18	3.43E-18	HLA-A/HLA	57
hsa04380	Osteoclast	45/643	128/8096	1.12E-18	1.84E-17	8.07E-18	IFNG/RELB	45
hsa05162	Measles	47/643	139/8096	1.16E-18	1.84E-17	8.07E-18	HSPA2/TLR	47
hsa04621	NOD-like re	53/643	184/8096	2.12E-17	3.16E-16	1.39E-16	CTSB/HSP9	53
hsa04659	Th17 cell d	39/643	108/8096	8.43E-17	1.19E-15	5.23E-16	CD4/HLA-D	39
hsa04660	T cell recep	38/643	104/8096	1.34E-16	1.79E-15	7.87E-16	CD4/IFNG/	38
hsa04657	IL-17 signa	36/643	94/8096	1.49E-16	1.89E-15	8.30E-16	HSP90AA1/	36
hsa05170	Human imr	56/643	212/8096	1.89E-16	2.29E-15	1.00E-15	B2M/CALR	56
hsa04010	MAPK sign	66/643	294/8096	2.48E-15	2.86E-14	1.26E-14	HSPA2/REL	66
hsa05142	Chagas dis	35/643	102/8096	2.03E-14	2.24E-13	9.84E-14	CALR/IFNG	35
hsa04625	C-type lect	35/643	104/8096	3.98E-14	4.21E-13	1.85E-13	FCER1G/RE	35
hsa04612	Antigen pr	30/643	78/8096	4.63E-14	4.71E-13	2.07E-13	B2M/CALR	30
hsa05160	Hepatitis C	43/643	157/8096	1.76E-13	1.72E-12	7.55E-13	IFNG/CXCL	43
hsa05135	Yersinia inf	39/643	137/8096	6.34E-13	5.96E-12	2.62E-12	CD4/CXCL8	39
hsa04933	AGE-RAGE	32/643	100/8096	2.41E-12	2.19E-11	9.62E-12	ICAM1/CX(	32
hsa04015	Rap1 signa	49/643	210/8096	2.62E-12	2.29E-11	1.01E-11	VEGFA/F2F	49
hsa05171	Coronaviru	52/643	232/8096	2.89E-12	2.44E-11	1.07E-11	CXCL8/CXC	52
hsa05321	Inflammat	25/643	65/8096	6.24E-12	5.11E-11	2.25E-11	HLA-DMB/	25
hsa05133	Pertussis	27/643	76/8096	8.01E-12	6.09E-11	2.68E-11	CXCL8/CXC	27
hsa04151	PI3K-Akt si	67/643	354/8096	8.15E-12	6.09E-11	2.68E-11	HSP90AA1/	67
hsa05330	Allograft re	19/643	38/8096	8.15E-12	6.09E-11	2.68E-11	HLA-A/HLA	19
hsa05140	Leishmania	27/643	77/8096	1.14E-11	8.29E-11	3.64E-11	HLA-DMB/	27
hsa04940	Type I diab	20/643	43/8096	1.26E-11	8.89E-11	3.90E-11	HLA-A/HLA	20
hsa01521	EGFR tyros	27/643	79/8096	2.28E-11	1.56E-10	6.87E-11	VEGFA/MA	27
hsa05332	Graft-versı	19/643	42/8096	7.60E-11	5.08E-10	2.23E-10	HLA-A/HLA	19

hsa05166	Human T-c	48/643	222/8096	7.96E-11	5.19E-10	2.28E-10	B2M/CALR,	48
hsa05218	Melanoma	25/643	72/8096	8.56E-11	5.43E-10	2.39E-10	MAPK3/PD	25
hsa05340	Primary im	18/643	38/8096	9.30E-11	5.76E-10	2.53E-10	CD4/TAP1/	18
hsa04014	Ras signalir	49/643	232/8096	1.21E-10	7.30E-10	3.21E-10	VEGFA/MA	49
hsa04012	ErbB signal	27/643	85/8096	1.56E-10	9.19E-10	4.04E-10	MAPK3/SR	27
hsa05161	Hepatitis B	39/643	162/8096	1.79E-10	1.03E-09	4.53E-10	CXCL8/TGF	39
hsa04658	Th1 and Th	28/643	92/8096	2.19E-10	1.24E-09	5.44E-10	CD4/HLA-D	28
hsa04510	Focal adhe	44/643	201/8096	3.31E-10	1.83E-09	8.04E-10	VEGFA/ITG	44
hsa04662	B cell recep	26/643	82/8096	3.57E-10	1.93E-09	8.49E-10	MAPK3/CD	26
hsa05152	Tuberculos	41/643	180/8096	3.77E-10	1.99E-09	8.76E-10	FCER1G/HL	41
hsa05235	PD-L1 expr	27/643	89/8096	5.02E-10	2.60E-09	1.14E-09	CD4/IFNG/	27
hsa05418	Fluid shear	34/643	139/8096	1.73E-09	8.78E-09	3.86E-09	CTSL/HSP9	34
hsa04672	Intestinal i	19/643	49/8096	1.88E-09	9.35E-09	4.11E-09	HLA-DMB/	19
hsa04360	Axon guida	40/643	182/8096	1.91E-09	9.35E-09	4.11E-09	CXCL12/M	40
hsa05416	Viral myoc	21/643	60/8096	2.38E-09	1.14E-08	5.01E-09	HLA-A/HLA	21
hsa05144	Malaria	19/643	50/8096	2.82E-09	1.32E-08	5.82E-09	ICAM1/IFN	19
hsa04210	Apoptosis	33/643	136/8096	3.75E-09	1.73E-08	7.61E-09	CTSB/CTSL,	33
hsa05145	Toxoplasm	29/643	112/8096	6.88E-09	3.12E-08	1.37E-08	HLA-DMB/	29
hsa05214	Glioma	23/643	75/8096	7.76E-09	3.46E-08	1.52E-08	MAPK3/PD	23
hsa05215	Prostate ca	26/643	97/8096	1.92E-08	8.42E-08	3.70E-08	HSP90AA1,	26
hsa04145	Phagosome	34/643	152/8096	2.01E-08	8.66E-08	3.81E-08	CALR/CTSL,	34
hsa05205	Proteoglyc	41/643	205/8096	2.21E-08	9.34E-08	4.10E-08	CTSL/TGFB	41
hsa05224	Breast canc	33/643	147/8096	2.97E-08	1.23E-07	5.39E-08	MAPK3/WI	33
hsa04640	Hematopoi	26/643	99/8096	3.05E-08	1.23E-07	5.39E-08	CD1C/CD1I	26
hsa04664	Fc epsilon	21/643	68/8096	3.05E-08	1.23E-07	5.39E-08	FCER1G/TN	21
hsa04810	Regulation	42/643	218/8096	4.58E-08	1.82E-07	7.99E-08	CXCL12/TN	42
hsa04917	Prolactin si	21/643	70/8096	5.37E-08	2.10E-07	9.22E-08	STAT1/SOC	21
hsa04066	HIF-1 signa	27/643	109/8096	6.30E-08	2.42E-07	1.06E-07	IFNG/CYBB	27
hsa05223	Non-small	21/643	72/8096	9.25E-08	3.51E-07	1.54E-07	MAPK3/NR	21
hsa05134	Legionellos	18/643	57/8096	2.03E-07	7.60E-07	3.34E-07	HSPA2/CXC	18
hsa05211	Renal cell c	20/643	69/8096	2.10E-07	7.73E-07	3.39E-07	TGFB1/VEC	20
hsa04217	Necroptosi	33/643	159/8096	2.17E-07	7.87E-07	3.46E-07	HSP90AA1,	33
hsa05146	Amoebiasis	25/643	102/8096	2.42E-07	8.67E-07	3.81E-07	CD1C/CD1I	25
hsa05212	Pancreatic	21/643	76/8096	2.57E-07	9.05E-07	3.98E-07	TGFB1/STA	21
hsa05320	Autoimmu	17/643	53/8096	3.45E-07	1.20E-06	5.28E-07	HLA-A/HLA	17
hsa05165	Human pap	53/643	331/8096	4.89E-07	1.68E-06	7.37E-07	HLA-A/HLA	53
hsa05221	Acute mye	19/643	67/8096	6.16E-07	2.09E-06	9.16E-07	PML/MAPK	19
hsa04350	TGF-beta s	23/643	94/8096	7.58E-07	2.53E-06	1.11E-06	IFNG/TGFB	23
hsa04622	RIG-I-like r	19/643	70/8096	1.29E-06	4.25E-06	1.87E-06	CXCL8/CXC	19
hsa04072	Phospholip	30/643	148/8096	1.31E-06	4.27E-06	1.88E-06	FCER1G/CX	30
hsa01522	Endocrine	23/643	98/8096	1.66E-06	5.35E-06	2.35E-06	MMP9/MA	23
hsa04370	VEGF signa	17/643	59/8096	1.89E-06	5.99E-06	2.63E-06	VEGFA/MA	17
hsa05222	Small cell l	22/643	92/8096	2.00E-06	6.27E-06	2.75E-06	ITGAV/TRA	22
hsa04670	Leukocyte	25/643	114/8096	2.26E-06	6.99E-06	3.07E-06	ICAM1/CXC	25
hsa05203	Viral carcin	36/643	204/8096	3.75E-06	1.15E-05	5.04E-06	HLA-A/HLA	36
hsa04020	Calcium sig	40/643	240/8096	4.75E-06	1.44E-05	6.31E-06	VEGFA/F2F	40
hsa05220	Chronic my	19/643	76/8096	4.93E-06	1.47E-05	6.48E-06	TGFB1/MA	19
hsa04666	Fc gamma	22/643	97/8096	5.10E-06	1.50E-05	6.58E-06	MAPK3/HC	22
hsa04935	Growth ho	25/643	119/8096	5.13E-06	1.50E-05	6.58E-06	STAT1/SOC	25
hsa04929	GnRH secre	17/643	64/8096	6.43E-06	1.86E-05	8.15E-06	MAPK3/AR	17

hsa03050	Proteasom	14/643	46/8096	7.56E-06	2.14E-05	9.39E-06	IFNG/PSMI	14
hsa04610	Compleme	20/643	85/8096	7.57E-06	2.14E-05	9.39E-06	PROCR/PLA	20
hsa05210	Colorectal	20/643	86/8096	9.15E-06	2.55E-05	1.12E-05	TGFB1/MA	20
hsa04218	Cellular ser	29/643	156/8096	1.18E-05	3.26E-05	1.43E-05	HLA-A/HLA	29
hsa04514	Cell adhesi	28/643	149/8096	1.35E-05	3.69E-05	1.62E-05	CD4/HLA-A	28
hsa05202	Transcripti	33/643	192/8096	1.69E-05	4.56E-05	2.00E-05	CXCL8/MM	33
hsa05207	Chemical c	35/643	212/8096	2.30E-05	6.16E-05	2.71E-05	HSP90AA1,	35
hsa05226	Gastric can	27/643	149/8096	3.78E-05	0.0001	4.39E-05	TGFB1/MA	27
hsa05150	Staphyloco	20/643	96/8096	5.07E-05	0.000133	5.84E-05	HLA-DMB/	20
hsa05219	Bladder ca	12/643	41/8096	5.24E-05	0.000136	5.96E-05	CXCL8/MM	12
hsa04926	Relaxin sig	24/643	129/8096	6.54E-05	0.000168	7.37E-05	TGFB1/MM	24
hsa05231	Choline me	20/643	98/8096	6.91E-05	0.000175	7.70E-05	MAPK3/PD	20
hsa04613	Neutrophil	31/643	190/8096	8.44E-05	0.000212	9.33E-05	TLR2/CTSG	31
hsa04623	Cytosolic D	15/643	63/8096	8.96E-05	0.000223	9.80E-05	CXCL10/IL1	15
hsa05143	African try	11/643	37/8096	9.17E-05	0.000226	9.94E-05	ICAM1/IFN	11
hsa05225	Hepatocell	28/643	168/8096	0.000127	0.00031	0.000136	TGFB1/MA	28
hsa04722	Neurotropl	21/643	119/8096	0.000393	0.000952	0.000418	MAPK3/FA	21
hsa05310	Asthma	9/643	31/8096	0.000487	0.001168	0.000513	FCER1G/HL	9
hsa01524	Platinum d	15/643	73/8096	0.000507	0.001202	0.000528	MAPK3/FA	15
hsa05131	Shigellosis	35/643	247/8096	0.00052	0.001223	0.000537	CXCL8/IL1E	35
hsa04068	FoxO signa	22/643	131/8096	0.000588	0.00137	0.000602	TGFB1/TNF	22
hsa04611	Platelet act	21/643	124/8096	0.000694	0.001603	0.000704	FCER1G/F2	21
hsa04550	Signaling p	23/643	143/8096	0.000831	0.001868	0.000821	MAPK3/WI	23
hsa05017	Spinocereb	23/643	143/8096	0.000831	0.001868	0.000821	NFYA/PSM	23
hsa05130	Pathogenic	29/643	197/8096	0.000831	0.001868	0.000821	CXCL8/IL1E	29
hsa05020	Prion disea	37/643	273/8096	0.00086	0.001915	0.000841	HSPA2/HSF	37
hsa05322	Systemic lu	22/643	136/8096	0.000989	0.002184	0.000959	HLA-DMB/	22
hsa05120	Epithelial c	14/643	70/8096	0.001023	0.002241	0.000984	CXCL8/CXC	14
hsa04915	Estrogen si	22/643	138/8096	0.001205	0.002616	0.001149	HSPA2/HSF	22
hsa04919	Thyroid ho	20/643	121/8096	0.00126	0.002712	0.001191	STAT1/ITG/	20
hsa04540	Gap junctic	16/643	88/8096	0.001357	0.002896	0.001272	MAPK3/GN	16
hsa04080	Neuroactiv	43/643	342/8096	0.001596	0.003379	0.001484	CTSG/F2R/	43
hsa05168	Herpes sim	58/643	497/8096	0.001655	0.003468	0.001524	B2M/CALR,	58
hsa04750	Inflammato	17/643	98/8096	0.001666	0.003468	0.001524	IL1B/F2RL1	17
hsa04920	Adipocytok	13/643	69/8096	0.002689	0.005552	0.002439	TNF/ADIPC	13
hsa04390	Hippo signa	23/643	157/8096	0.002951	0.006046	0.002656	TGFB1/WN	23
hsa04024	cAMP signa	29/643	219/8096	0.004236	0.008608	0.003782	F2R/MAPK	29
hsa05132	Salmonella	32/643	249/8096	0.004363	0.008795	0.003863	HSP90AA1,	32
hsa05010	Alzheimer	45/643	384/8096	0.004956	0.009912	0.004354	PSMC2/PSI	45
hsa04071	Sphingolipi	18/643	119/8096	0.005751	0.011412	0.005013	FCER1G/TN	18
hsa05100	Bacterial in	13/643	77/8096	0.00712	0.014019	0.006158	SRC/PTK2/	13
hsa04960	Aldosteron	8/643	37/8096	0.007414	0.014486	0.006363	MAPK3/PIK	8
hsa05230	Central car	12/643	70/8096	0.008441	0.016367	0.00719	MAPK3/PD	12
hsa04213	Longevity r	11/643	62/8096	0.008853	0.017035	0.007483	HSPA2/HD/	11
hsa04930	Type II dial	9/643	46/8096	0.009125	0.017427	0.007655	TNF/ADIPC	9
hsa04211	Longevity r	14/643	89/8096	0.009993	0.018943	0.008321	ADIPOQ/PI	14
hsa04932	Non-alcoho	21/643	155/8096	0.01072	0.020021	0.008795	CXCL8/TGF	21
hsa04934	Cushing sy	21/643	155/8096	0.01072	0.020021	0.008795	MAPK3/GN	21
hsa04115	p53 signali	12/643	73/8096	0.01174	0.021766	0.009561	TNFRSF10E	12
hsa04923	Regulation	10/643	57/8096	0.013274	0.024432	0.010733	GNAI1/PTG	10

hsa05213	Endometri	10/643	58/8096	0.014937	0.027295	0.01199	MAPK3/NR	10
hsa04924	Renin secre	11/643	69/8096	0.019218	0.034868	0.015317	CTSB/GNAI	11
hsa04928	Parathyroi	15/643	106/8096	0.019807	0.035681	0.015674	MAPK3/GN	15
hsa04520	Adherens j	11/643	71/8096	0.023387	0.041834	0.018377	MAPK3/SR	11
hsa04371	Apelin sign	18/643	138/8096	0.024636	0.043759	0.019223	MAPK3/GN	18
hsa04152	AMPK sign	16/643	120/8096	0.027433	0.048389	0.021257	ADIPOQ/PI	16
hsa04725	Cholinergic	15/643	113/8096	0.033195	0.058149	0.025544	MAPK3/GN	15
hsa04215	Apoptosis -	6/643	32/8096	0.03755	0.065141	0.028616	BIRC5/BID/	6
hsa04150	mTOR sign	19/643	155/8096	0.0377	0.065141	0.028616	TNF/MAPK	19
hsa04730	Long-term	9/643	60/8096	0.045799	0.078601	0.034529	MAPK3/GN	9

Count
153
131
127
132
133
135
135
130
116
119
121
106
115
125
108
106
103
110
85
100
95
79
84
81
98
106
78
92
104
77
88
76
88
78
88
84
108
104
88
81
99
87
70
74
72
59
92
74
59

63
91
74
68
59
54
57
61
56
56
77
77
67
57
65
55
61
86
60
63
80
53
66
44
66
77
65
45
45
55
45
45
76
53
45
63
73
81
58
47
47
50
61
43
44
87
67
47
74
61

46
57
51
46
59
50
41
68
51
51
52
47
53
49
41
65
59
46
47
85
46
55
61
83
52
71
40
86
37
50
82
53
47
54
42
81
40
39
67
59
60
74
39
70
60
58
46
44
38
31

37
76
50
33
68
35
45
42
36
37
46
51
71
67
63
68
34
47
36
76
39
65
31
31
39
43
50
37
47
39
49
47
32
52
46
56
40
46
37
36
59
43
32
62
70
37
35
32
32
48

26
35
33
29
51
26
50
75
43
75
41
61
35
48
26
70
41
40
49
36
47
58
25
62
72
26
31
33
44
68
45
25
42
42
26
33
24
43
68
64
30
31
32
38
45
27
50
44
25
57

37
30
36
40
46
68
30
25
22
41
42
51
22
35
61
40
28
33
53
64
56
44
39
29
27
38
20
20
51
30
38
21
42
18
18
24
61
35
23
64
35
58
16
45
37
49
44
33
28
25

37
34
20
26
22
44
39
20
23
30
46
44
37
23
39
46
30
21
56
41
55
64
20
26
27
39
55
28
63
29
18
63
47
30
28
24
17
29
42
25
28
35
22
24
21
64
23
30
15
61

39
14
31
27
48
32
52
61
39
36
24
27
19
19
48
37
21
26
24
12
12
20
29
19
14
37
11
30
58
20
57
19
55
31
31
42
45
18
18
36
34
38
20
49
40
22
25
25
36
20

32
16
16
14
51
15
61
38
37
27
18
21
37
12
23
16
54
50
56
51
41
21
34
20
23
41
13
27
39
22
23
31
25
12
20
39
33
18
18
34
31
31
45
61
30
27
13
18
21
46

19
27
17
20
36
18
18
18
18
35
45
17
28
50
16
16
38
13
13
44
18
43
33
23
19
59
26
21
37
23
16
16
12
22
13
42
37
15
18
18
19
23
42
37
55
33
31
57
13
51

29
47
32
57
17
26
29
15
57
20
22
23
19
46
15
27
23
23
11
25
22
18
18
31
14
14
21
33
20
46
32
16
43
34
19
27
40
11
26
14
25
12
16
30
10
23
15
15
29
14

14
23
8
42
22
15
43
28
21
13
13
24
18
37
23
43
40
17
24
15
12
12
21
21
55
20
26
43
24
35
29
22
14
14
12
35
47
16
13
15
10
8
11
11
17
17
19
23
23
22

15
30
19
19
12
12
12
12
13
13
18
20
37
15
18
18
11
11
11
54
20
22
10
10
13
27
30
30
12
15
14
14
19
51
34
9
44
44
13
11
11
22
19
19
12
12
14
14
20
10

10
17
15
13
18
14
17
46
19
50
43
26
26
18
9
10
13
35
18
20
20
30
25
8
12
12
22
19
35
15
15
15
15
16
23
10
10
18
19
28
12
12
35
25
9
14
11
11
11
11

13
19
31
28
27
32
14
14
12
16
13
8
10
32
41
19
14
9
9
9
9
9
15
28
13
30
45
36
25
31
14
11
11
11
15
29
13
12
41
18
18
14
14
8
24
40
9
37
42
19

19
11
11
11
11
13
22
21
7
7
7
10
10
15
19
26
27
26
13
15
15
36
11
11
39
12
10
8
26
15
15
13
13
14
19
26
23
36
12
11
23
23
23
13
13
13
28
7
7
7

25
23
23
23
33
24
44
27
22
12
16
24
11
11
11
14
18
18
13
8
8
24
17
15
21
16
10
14
13
9
11
11
11
15
15
32
19
14
12
27
40
28
16
7
7
7
7
7
7
26

35
23
11
11
11
34
30
14
32
16
9
9
9
15
12
28
40
14
14
28
10
18
12
16
8
8
26
15
9
9
23
11
13
7
7
7
7
7
31
10
10
10
12
12
15
14
36
17
13
13

11
11
11
11
30
14
9
9
12
32
40
8
8
8
8
16
18
10
27
17
34
6
6
29
15
13
31
33
20
18
17
11
11
7
7
30
23
39
13
37
26
16
47
12
12
8
8
11
11
11

16
16
20
13
13
28
31
36
9
11
13
20
20
7
7
7
6
6
16
23
18
8
12
19
14
10
13
17
17
25
11
15
9
9
9
9
19
19
23
23
14
14
14
14
28
12
12
23
10
10

11
11
17
27
20
29
8
8
8
7
7
7
7
7
7
7
12
13
9
28
29
17
44
19
19
11
11
10
10
6
6
6
13
16
14
9
9
23
13
13
20
10
27
14
14
7
7
7
39
13

13
13
11
15
14
12
12
9
9
10
10
8
18
17
17
6
6
6
6
14
14
39
12
7
7
7
15
9
39
14
18
17
11
11
8
18
22
10
9
9
12
11
16
24
22
14
13
27
20
7

7
7
18
18
6
6
28
31
8
38
12
12
12
10
14
38
13
11
24
26
22
14
12
38
25
43
8
39
7
7
14
14
38
9
9
9
9
16
21
41
38
6
6
6
6
6
6
6
10
10

10
10
10
14
16
13
12
8
9
14
28
7
7
7
7
7
5
5
5
5
5
5
5
5
5
10
13
12
20
18
18
14
28
26
15
35
24
11
9
9
13
26
8
12
16
22
6
6
6
6

6
6
6
14
15
13
7
7
7
12
15
21
28
29
29
9
10
10
42
13
8
8
18
36
11
30
5
5
5
5
15
10
9
9
9
6
6
6
6
7
7
7
7
16
12
11
17
17
33
8

22
34
14
16
20
20
10
10
10
10
23
12
9
19
15
11
11
20
13
13
7
7
7
8
12
10
10
10
17
14
14
11
13
6
6
6
6
30
32
32
28
20
18
18
15
15
16
12
19
19

5
5
5
5
5
13
10
10
18
11
8
8
8
8
16
20
38
7
7
7
7
14
14
14
13
22
19
17
16
29
24
14
6
6
6
6
6
41
36
23
15
15
15
8
8
22
18
20
10
30

7
7
7
7
16
26
13
5
5
5
5
5
5
5
5
5
5
14
14
9
21
30
23
18
8
8
8
13
10
10
12
38
6
6
6
6
6
6
6
14
26
18
38
11
7
7
7
9
16
10

27
15
26
41
24
36
8
17
13
13
13
32
14
15
20
18
23
9
9
39
13
6
6
6
6
5
5
5
5
5
5
7
7
11
18
8
8
16
13
10
17
18
18
9
9
11
7
7
7
7

7
14
20
10
16
21
6
6
6
17
9
9
9
20
5
5
5
5
5
5
17
14
13
16
18
7
7
7
9
23
17
6
6
6
6
8
9
9
9
9
38
16
17
17
22
7
7
7
20
27

11
11
15
10
10
16
5
5
5
5
5
5
32
14
12
28
25
21
9
31
8
6
6
6
6
6
6
10
10
13
13
15
17
7
7
7
7
20
9
14
14
4
4
4
4
4
4
4
4
4

4
4
4
4
4
4
4
4
8
8
8
13
13
20
20
32
20
5
5
5
5
5
5
5
5
11
11
6
6
6
6
27
9
7
7
7
7
7
7
22
22
17
8
22
24
21
21
13
20
17

14
9
9
10
10
15
31
27
19
13
7
7
7
7
25
11
8
8
6
6
6
6
37
10
5
5
5
5
5
13
9
19
27
4
4
4
4
4
4
4
4
4
4
4
12
8
7
7
10
14

14
9
9
9
9
11
6
6
6
6
6
6
13
13
15
20
19
12
8
11
9
9
9
5
5
5
5
5
7
7
15
13
13
32
22
18
11
11
6
6
6
6
6
6
6
8
8
4
4
4

4
4
4
4
4
4
4
4
4
4
4
12
12
15
7
7
7
7
7
7
7
20
11
15
9
9
5
5
5
5
5
5
5
5
8
8
8
8
8
8
13
31
10
15
21
21
7
7
7
7

7
16
20
9
9
9
9
9
9
19
15
8
8
8
8
18
10
10
16
12
29
17
6
6
6
6
6
6
6
6
4
4
4
4
4
4
4
4
4
4
4
4
4
4
4
4
4
4
4
4

4
7
7
7
7
7
7
9
9
9
9
5
5
5
5
5
5
5
5
5
5
5
5
12
12
8
24
22
19
14
7
7
7
8
8
8
6
6
6
6
17
11
15
15
12
10
10
10
9
9

5
5
5
5
5
5
5
5
5
5
5
15
12
12
8
8
8
7
7
4
4
4
4
4
4
4
4
4
4
4
4
4
15
9
9
6
6
27
13
17
24
15
8
7
7
7
28
9
9
9

5
5
5
5
5
5
5
12
35
17
6
6
18
10
10
10
10
10
10
14
19
8
8
8
11
11
11
9
22
7
7
7
19
4
4
4
4
4
4
4
4
4
4
4
4
4
4
20
15
10

12
12
11
20
29
5
5
5
5
5
5
5
5
9
15
15
14
6
6
6
6
6
6
18
12
12
7
7
7
7
7
7
7
11
13
18
9
9
22
34
8
13
18
6
6
6
6
6
6
7

7
5
5
5
5
5
5
5
5
5
24
4
4
4
4
4
4
4
4
4
4
12
19
9
8
8
8
8
10
34
12
12
7
7
7
7
11
6
6
8
12
13
13
5
5
5
5
5
5
5

5
20
9
36
10
14
7
7
7
4
4
4
4
4
4
4
8
31
6
18
18
13
10
10
10
16
14
5
5
5
5
5
5
8
8
8
8
7
7
12
10
10
17
9
6
6
6
6
6
6

11
12
17
8
8
4
4
4
4
4
14
14
7
7
7
7
7
7
9
9
9
9
9
11
11
12
12
5
5
5
5
33
26
6
6
6
10
8
3
3
3
3
3
3
3
3
3
3
3
3

3
3
3
3
3
3
3
3
3
3
3
11
7
7
7
24
21
12
12
8
8
8
8
14
9
9
9
9
6
6
6
5
5
5
5
5
5
5
4
4
4
4
4
4
4
4
4
4
4
4

4
4
4
7
7
10
10
15
11
9
8
8
8
19
11
6
7
7
7
7
17
5
5
5
5
5
5
8
8
3
3
3
3
3
3
3
3
3
3
3
3
3
3
3
3
3
3
3
3
3

3
3
3
3
3
3
3
3
3
3
3
3
3
3
3
3
3
3
4
4
4
4
4
4
4
4
4
4
14
21
17
9
9
6
6
16
8
8
29
5
5
5
5
5
9
13
7
10
6
6

6
17
4
4
4
4
4
4
4
4
4
4
4
4
4
4
4
4
4
16
18
8
13
5
5
5
3
3
3
3
3
3
3
3
3
3
3
3
3
3
3
3
3
3
3
3
3
3
3
3

3
6
6
6
6
10
10
31
14
34
13
15
7
7
7
14
12
4
4
4
4
4
4
4
4
5
5
5
5
5
5
5
6
6
6
14
8
7
9
9
9
14
14
11
28
12
6
6
6
3

3
3
3
3
3
3
3
3
3
3
3
3
3
3
3
3
3
3
3
5
5
5
5
5
5
7
7
7
7
20
4
4
4
4
4
4
4
4
4
4
4
4
4
21
14
8
9
9
9
9

9
27
6
6
6
6
7
7
10
10
18
5
5
5
5
5
5
8
4
4
4
4
4
4
11
6
6
6
14
14
9
3
3
3
3
3
3
3
3
3
3
3
3
3
3
3
3
3
3
8

8
8
8
13
5
5
5
5
10
22
6
6
6
6
6
16
10
10
4
4
4
4
4
4
4
4
9
9
9
9
24
5
5
5
7
7
10
10
8
8
11
6
6
9
3
3
3
3
3
3

3
3
3
3
3
3
3
3
3
3
3
3
3
3
11
10
10
10
15
4
4
4
4
4
4
4
4
4
4
4
4
4
4
4
4
4
5
5
5
5
5
9
10
8
8
8
7
7
10
6

6
5
5
5
5
5
5
7
7
7
4
4
4
4
4
4
4
4
4
10
9
3
3
3
3
3
3
3
3
3
3
3
3
3
3
3
3
3
3
3
23
14
16
16
6
6
7
5
5
5

5
5
5
21
17
19
4
4
4
4
4
4
4
4
6
7
20
10
3
3
3
3
3
3
3
3
3
3
3
3
3
3
15
8
8
8
9
12
6
6
6
6
10
10
131
53
66
34
52
52

52
33
49
48
46
47
29
31
29
44
53
12
27
11
19
11
18
44
26
15
12
18
14
8
42
34
14
42
6
9
9
9
7
6
23
22
31
9
12
16
22
5
15
14
13
11
21
16
5
19

10
8
29
21
13
6
6
13
10
12
14
7
34
5
6
17
13
13
4
31
28
31
17
8
11
15
10
4
11
3
15
22
177
177
110
103
68
69
55
55
47
48
28
34
32
26
21
58
35
36

36
20
31
15
17
15
15
20
20
14
30
16
46
20
22
19
42
47
26
14
9
39
15
14
11
10
10
9
11
10
13
11
7
17
17
29
10
7
10
14
9
15
15
10
6
7
28
14
14
6

6
6
6
7
7
6
23
19
8
22
6
6
16
8
14
5
21
8
6
6
6
9
5
7
7
12
7
10
6
5
7
6
6
5
12
18
10
6
5
6
26
5
5
8
6
6
11
8
9
23

5
5
5
8
7
4
5
7
7
20
10
4
4
4
6
5
6
4
4
24
11
4
4
5
8
6
12
34
15
23
7
10
4
4
6
28
18
10
10
5
4
19
7
6
8
4
6
4
4
10

13
3
3
11
5
19
25
8
3
4
