# Supplementary material for: Building an Immune-Related Genes Model to Predict Treatment, Extracellular Matrix, and Prognosis of Head and Neck Squamous Cell Carcinoma
Source: Mediators Inflamm. 2023 Jul 11;2023:6680731. doi: 10.1155/2023/6680731 (PMC10353907; doi:10.1155/2023/6680731)
Supplement: Supplementary 6 — The detailed values of univariate and multivariate Cox regression analysis. [file 6680731.f6.pdf]

Univariate Cox regression analysis

id	HR	HR.95L	HR.95H	pvalue
Age	1.379535674	1.022716648	1.860846481	0.03511178
Gender	0.778807585	0.568422954	1.06705975	0.119713964
Grade	1.160543194	0.922834925	1.459481505	0.20293652
Stage	1.451799835	1.205963064	1.747750677	8.20E-05
IRGPI	1.850991524	1.581609661	2.166254864	1.68E-14

multivariate Cox regression analysis

id	HR	HR.95L	HR.95H	pvalue
Age	1.602395877	1.183779025	2.169047171	0.002272877
Stage	1.514006913	1.25774609	1.822479873	1.17E-05
IRGPI	2.014214902	1.691145497	2.399002143	4.15E-15
