# Supplementary material for: Building an Immune-Related Genes Model to Predict Treatment, Extracellular Matrix, and Prognosis of Head and Neck Squamous Cell Carcinoma
Source: Mediators Inflamm. 2023 Jul 11;2023:6680731. doi: 10.1155/2023/6680731 (PMC10353907; doi:10.1155/2023/6680731)
Supplement: Supplementary 7 — The GSEA in different groups. [file 6680731.f7.pdf]

ID	Descriptior	setSize	enrichmen	NES	pvalue	p.adjust	qvalues	rank
KEGG_HYP	KEGG_HYP	62	0.699758	2.497823	1.95E-09	3.52E-07	3.08E-07	914
KEGG_FOC	KEGG_FOC	170	0.484932	2.109414	1.50E-07	1.35E-05	1.18E-05	2002
KEGG_DILA	KEGG_DILA	65	0.623262	2.277318	1.52E-06	9.13E-05	8.01E-05	914
KEGG_ARR	KEGG_ARR	53	0.636119	2.206406	7.35E-06	0.000331	0.00029	914
KEGG_CAR	KEGG_CAR	57	0.603212	2.099505	1.43E-05	0.000515	0.000452	1367
KEGG_ECM	KEGG_ECM	64	0.556933	2.032462	4.56E-05	0.001369	0.001201	1615
KEGG_CELI	KEGG_CELI	107	-0.60069	-1.69033	0.000314	0.007349	0.006446	2088
KEGG_INTE	KEGG_INTE	32	-0.74052	-1.80642	0.000327	0.007349	0.006446	2703
KEGG_PRIN	KEGG_PRIN	29	-0.75457	-1.81098	0.000578	0.01156	0.010141	1769
KEGG_GLY(	KEGG_GLY(	19	0.717995	2.082828	0.000823	0.014815	0.012996	2447
KEGG_PRO	KEGG_PRO	42	0.527496	1.756057	0.003066	0.047627	0.041778	5417
KEGG_AUT	KEGG_AUT	26	-0.72837	-1.72415	0.003175	0.047627	0.041778	2484
KEGG_CHE	KEGG_CHE	160	-0.51688	-1.48452	0.003764	0.0507	0.044474	2191
KEGG_ALL(	KEGG_ALL(	27	-0.70939	-1.68708	0.003943	0.0507	0.044474	2484
KEGG_TYPI	KEGG_TYPI	32	-0.66729	-1.62777	0.004284	0.051405	0.045092	2484
KEGG_TAS	KEGG_TAS	13	-0.77972	-1.638	0.004818	0.054205	0.047548	2125
KEGG_DRU	KEGG_DRU	37	-0.65754	-1.64298	0.006592	0.069794	0.061223	1352
KEGG_ARA	KEGG_ARA	41	-0.64079	-1.61967	0.00807	0.080698	0.070788	2722
KEGG_LIN(	KEGG_LIN(	15	-0.76429	-1.6351	0.009317	0.088269	0.077429	3106
KEGG_ASTI	KEGG_ASTI	17	-0.74737	-1.64134	0.010431	0.093422	0.081949	2484
KEGG_RETI	KEGG_RETI	27	-0.66989	-1.59314	0.010899	0.093422	0.081949	1545
KEGG_ALPI	KEGG_ALPI	12	-0.73847	-1.51297	0.016113	0.127176	0.111558	3106
KEGG_REG	KEGG_REG	172	0.313253	1.372703	0.01625	0.127176	0.111558	1681
KEGG_T_CI	KEGG_T_CI	96	-0.51727	-1.44252	0.019156	0.143667	0.126023	2703
KEGG_CYT(	KEGG_CYT(	183	-0.45972	-1.32667	0.020301	0.146164	0.128214	2279
KEGG_B_C	KEGG_B_C	72	-0.53592	-1.45264	0.030735	0.212784	0.186653	3983
KEGG_SMA	KEGG_SMA	78	0.376804	1.376237	0.034135	0.227566	0.199619	1596
KEGG_O_G	KEGG_O_G	23	-0.63632	-1.47697	0.047014	0.292135	0.256259	1484
KEGG_GLY(	KEGG_GLY(	20	-0.6468	-1.46702	0.048408	0.292135	0.256259	1090
KEGG_GRA	KEGG_GRA	29	-0.60724	-1.45739	0.048689	0.292135	0.256259	2484

leading_ed	core_enrichment
tags=45%,	TTN/ACTC1/DES/CACNA1S/ITGA5/CACNG1/MYL2/ITGA3/CACNG6/ITGB6/ITGA6/CACNG4/
tags=35%,	LAMA3/ACTN2/LAMC2/FN1/VEGFC/CAV1/ITGA5/MYLPF/THBS1/FLNC/MYL2/ITGA3/PDGF
tags=40%,	TTN/ACTC1/DES/CACNA1S/ITGA5/CACNG1/MYL2/ITGA3/CACNG6/ITGB6/ITGA6/CACNG4/
tags=38%,	DES/ACTN2/CACNA1S/CDH2/ITGA5/CACNG1/ITGA3/CACNG6/ITGB6/ITGA6/CACNG4/SGC
tags=32%,	ACTC1/CACNA1S/CACNG1/MYL2/ATP1A2/CACNG6/COX6A2/CACNG4/TPM1/TNNC1/TPM
tags=45%,	LAMA3/LAMC2/FN1/ITGA5/THBS1/ITGA3/ITGB6/ITGA6/LAMB3/IBSP/COL4A6/ITGB1/TNC/
tags=48%,	CD34/HLA-DRB1/CLDN20/HLA-DQB1/CLDN23/HLA-G/PECAM1/MPZ/HLA-DRB5/HLA-DMA/
tags=72%,	MAP3K14/HLA-DRB1/HLA-DQB1/HLA-DRB5/TNFSF13/HLA-DMA/HLA-DPB1/HLA-DRA/HLA-
tags=59%,	CD4/ICOS/BTK/CD8B/CD8A/BLNK/CIITA/CD3E/CD3D/PTPRC/LCK/JAK3/ZAP70/IL2RG/TNFR
tags=63%,	CHPF/CHST11/DSE/CSGALNACT2/CHPF2/XYLT2/CHST3/B3GAT3/CHST15/CHSY1/CHSY3/B4
tags=83%,	PSMD2/POMP/PSMC1/PSMA7/PSMA1/PSMD7/PSMB9/PSMB7/PSMC6/PSMD13/PSMD4/
tags=69%,	HLA-DRB1/HLA-DQB1/HLA-G/GZMB/HLA-DRB5/HLA-DMA/HLA-DPB1/PRF1/HLA-DRA/HLA-
tags=29%,	CX3CL1/PPBP/ADCY4/GNG2/GNGT2/CCL21/FGR/PRKX/CCL17/PIK3R5/CCR8/PREX1/CXCL1
tags=67%,	HLA-DRB1/HLA-DQB1/HLA-G/GZMB/HLA-DRB5/HLA-DMA/HLA-DPB1/PRF1/HLA-DRA/HLA-
tags=66%,	HLA-DRB1/HLA-DQB1/HLA-G/GZMB/HLA-DRB5/HLA-DMA/HLA-DPB1/PRF1/HLA-DRA/HLA-
tags=62%,	ADCY4/PRKX/ADCY6/PLCB2/SCNN1G/TAS1R3/SCNN1B/SCNN1A
tags=35%,	ALDH3A1/UGT1A8/AOX1/GSTM1/ADH7/CYP3A5/GSTA1/CYP2E1/MAOB/CYP2C18/FMO2/
tags=59%,	CBR1/TBXAS1/PLA2G6/PLA2G2F/CYP2J2/ALOX15B/JMJD7-PLA2G4B/GGT1/ALOX15/ALOX
tags=80%,	PLA2G6/PLA2G2F/CYP2J2/JMJD7-PLA2G4B/ALOX15/AKR1B10/PLA2G4E/CYP3A5/PLA2G3/
tags=76%,	HLA-DRB1/HLA-DQB1/HLA-DRB5/HLA-DMA/HLA-DPB1/HLA-DRA/HLA-DPA1/HLA-DMB/HL
tags=37%,	RDH10/CYP26A1/UGT1A8/ADH7/ALDH1A1/DHRS9/RDH12/CYP3A5/CYP2C18/ADH1C
tags=75%,	PLA2G6/ACOX1/PLA2G2F/JMJD7-PLA2G4B/ACOX3/FADS2/PLA2G4E/PLA2G3/PLA2G2D
tags=24%,	ACTN2/FN1/ITGA5/FGF19/MYLPF/MYL2/ITGA3/PDGFA/ITGB6/MYLK2/ITGA6/PXN/ACTN1/
tags=31%,	MAP3K14/LCP2/NFATC2/IKBKB/NCK1/FOS/MAPK13/CD4/PIK3R5/VAV1/ICOS/IFNG/CTLA4
tags=32%,	IL7/TNFRSF9/CX3CL1/PPBP/ACVR2A/TNFRSF14/CCL21/KDR/TNFSF13/TNFRSF1B/CCL17/C
tags=40%,	NFATC4/KRAS/VAV3/CHP1/PTPN6/PIK3CB/PIK3AP1/CD72/RAF1/RASGRP3/PLCG2/LYN/NF
tags=23%,	LAMA3/LAMC2/FN1/PTGS2/ITGA3/BIRC2/ITGA6/LAMB3/CCND1/COL4A6/CDK6/ITGB1/LA
tags=30%,	GCNT3/GALNT12/GCNT4/GALNT5/GALNT17/ST6GALNAC1/B3GNT6
tags=25%,	ABO/FUT2/FUT7/FUT3/FUT6
tags=62%,	HLA-DRB1/HLA-DQB1/HLA-G/GZMB/HLA-DRB5/HLA-DMA/HLA-DPB1/PRF1/HLA-DRA/HLA-

SGCA/TPM1/SGCG/TNNC1/TPM2/SGCD/CACNB1/MYH7/IL6/ITGB1/MYL3/PRKAB2/PRKAA2/ITGB4/TNI
A/CAV3/BIRC2/ITGB6/MYLK2/ITGA6/LAMB3/CCND1/IBSP/PXN/COL4A6/CAV2/ACTN1/PDGFC/EGFR/VA
SGCA/TPM1/SGCG/TNNC1/TPM2/SGCD/CACNB1/MYH7/ITGB1/PLN/MYL3/ITGB4/TNNI3/ITGA7
A/SGCG/SGCD/CACNB1/ACTN1/ITGB1/DSG2/ITGB4/ITGA7
2/COX7B2/CACNB1/MYH7/MYL3/TNNI3/TNNT2/SLC9A1
/COL5A1/COL5A3/SDC4/ITGB4/AGRN/HMMR/ITGA7/COL5A2/SDC3/LAMC1/COL4A2/COL4A1/CD44/LA
/ITGB2/HLA-DPB1/HLA-DRA/HLA-DPA1/CD4/HLA-DMB/ITGA9/JAM2/SELPLG/ICOS/HLA-DQA1/HLA-DO
-DPA1/HLA-DMB/CXCL12/ICOS/HLA-DQA1/HLA-DOB/CCL28/HLA-DOA/HLA-DQA2/ITGB7/CXCR4/CD28/
SF13C/CD19/CD79A
GALT7
PSMA6/PSMF1/PSMC3/PSMD14/PSMB5/PSMB3/PSMB1/PSMD11/PSMB2/SEM1/PSMC2/PSMA2/PSMI
-DPA1/HLA-DMB/HLA-DQA1/HLA-DOB/CTLA4/HLA-DOA/HLA-DQA2/FASLG/CD28
2/VAV1/CCL2/CCL22/ADCY6/PLCB2/ARRB1/CCL18/TIAM1/CCL28/WAS/XCL2/CCL24/CCR5/RASGRP2/D
-DPA1/HLA-DMB/IFNG/HLA-DQA1/HLA-DOB/HLA-DOA/HLA-DQA2/FASLG/CD28
-DPA1/HLA-DMB/IFNG/HLA-DQA1/HLA-DOB/CPE/HLA-DOA/HLA-DQA2/FASLG/PTPRN2/CD28/ICA1

FMO3/ADH1C
5/CYP4F3/CBR3/PTGIS/HPGDS/PLA2G4E/EPHX2/ALOX12/GGT6/ALOX12B/PLA2G3/CYP2E1/PTGDS/CYP
CYP2E1/CYP2C18/PLA2G2D
A-DQA1/HLA-DOB/FCER1A/HLA-DOA/HLA-DQA2

/PDGFC/EGFR/RRAS2/VAV2/ITGB1/GNA12/PDGFB/PFN2/CFL2/ARPC1B/DIAPH3/ITGB4/FGFR4/ITGA7/N
/MAP3K8/PDCD1/CD8B/CD8A/TEC/CD3E/CD3D/PTPRC/LCK/PIK3CG/PIK3R3/ITK/CD28/CD247/CD3G/Z
CR8/CXCL12/IL3RA/CCL2/CCL22/IFNG/EDAR/CNTFR/CSF2RA/IL23A/CCL18/CCL28/TNFSF15/XCL2/IL10R
ATC2/IKBKB/SYK/FOS/PIK3R5/VAV1/FCGR2B/BTK/INPP5D/BLNK/PIK3CG/PIK3R3/PRKCB/CD79B/CD22/
MC1/PIK3CD/COL4A2/COL4A1/LAMB1/ITGAV

-DPA1/HLA-DMB/IFNG/HLA-DQA1/HLA-DOB/HLA-DOA/HLA-DQA2/FASLG/CD28

NI3/ITGA7
AV2/ITGB1/TNC/PDGFB/COL5A1/MET/PARVB/PRKCA/FLNA/COL5A3/ITGB4/PGF/ITGA7/COL5A2/BCAR

AMB1/ITGAV/SDC2
B/CTLA4/CLDN7/PDCD1/ICAM2/CD8B/CD8A/HLA-DOA/CLDN4/CADM1/NRCAM/HLA-DQA2/CD2/OCLN
/TNFRSF13C/TNFRSF17/PIGR

E4/PSMA3/PSMD3/PSMA4/PSME3/PSMB6/PSMA5/PSMD1/PSMB4/PSMD8/PSMD12/PSMB8

OCK2/CXCR6/CXCR4/JAK3/PIK3CG/PIK3R3/ITK/CXCL13/PRKCB/CX3CR1/NCF1/CCR7/GNG7/CCR2/CXCR

2C18/PLA2G2D

MSN/RRAS/BCAR1/BDKRB1/PIK3CD/CFL1/MYL9/MYH10/SLC9A1/ACTB/ITGAV/MRAS/PFN1/VCL
AP70/RASGRP1
A/CCL24/CCR5/TNFSF18/IL2RB/FASLG/LTB/IL20RA/TNFRSF11A/CXCR6/CXCR4/TNFSF4/TNFRSF4/CXCL
/CD19/CD79A

1/IGF1R/LAMC1/PIK3CD/SHC1/PARVA/MYL9/COL4A2/COL4A1/LAMB1/ACTB/ITGAV/VEGFA/VCL/RAP1

N/ITGB7/PTPRC/SPN/CD28/ITGAL/SELL/CD6/ICAM3/CLDN17/SELP/CLDN3/SELE/CLDN6/VCAM1/CD22/C

R3/CXCR2/CCR4/CCL19/ADCY5/GNG8

13/TNFRSF11B/IL21R/CX3CR1/CCR7/CCR2/IL2RG/CXCR3/CXCR2/TNFRSF19/CCR4/CSF2RB/CCL19/KIT/A

.B/COL6A2/PIK3CA/ILK/PPP1CA

CLDN10

AMH/TNFRSF13C/CD27/IL17RB/TNFRSF17/IL19
