# Supplementary material for: Building an Immune-Related Genes Model to Predict Treatment, Extracellular Matrix, and Prognosis of Head and Neck Squamous Cell Carcinoma
Source: Mediators Inflamm. 2023 Jul 11;2023:6680731. doi: 10.1155/2023/6680731 (PMC10353907; doi:10.1155/2023/6680731)
Supplement: Supplementary 8 — The relative proportion of various immune cells by CIBERSORT. [file 6680731.f8.pdf]

immune	cor	pvalue
B cell naive	-0.13712	0.002141
B cell mem	-0.09846	0.027853
B cell plasn	-0.13205	0.003123
T cell CD8+	-0.26457	1.94E-09
T cell CD4+	0.158416	0.000382
T cell follicu	-0.29986	7.94E-12
T cell regul	-0.16911	0.000147
NK cell rest	0.110945	0.013147
Monocyte	-0.09464	0.034554
Macrophag	0.284235	9.98E-11
Macrophag	-0.09458	0.034662
Myeloid de	-0.09569	0.032594
Mast cell a	-0.25002	1.50E-08
Mast cell re	0.203294	4.70E-06
Eosinophil	0.124214	0.00546
